# Two-year effects of the community-based overweight and obesity intervention program Gezond Onderweg! (GO!) in children and adolescents living in a low socioeconomic status and multi-ethnic district on Body Mass Index-Standard Deviation Score and quality of life

**DOI:** 10.1016/j.eclinm.2021.101217

**Published:** 2021-11-30

**Authors:** Dagna Lek, Annemien Haveman-Nies, Janine Bezem, Sonay Zainalabedin, Safina Schetters-Mouwen, Jenneke Saat, Gerrit Gort, Lian Roovers, Petra van Setten

**Affiliations:** a Wageningen University & Research, Consumption and Healthy Lifestyles, Wageningen, The Netherlands; b Public Health Services Gelderland-Midden, Department of Youth Health, Arnhem, The Netherlands; c Malburgen Medical Center, General Practice Malburgen Arnhem, The Netherlands; d Academic Collaborative Center AMPHI, Integrated Health Policy, Department of Primary and Community Care, ELG 117, Radboud University Medical Center, Nijmegen, The Netherlands; e HAN University of Applied Sciences, Academy of Paramedical Studies, Department of Nutrition and Dietetics, Nijmegen, The Netherlands; f Wageningen University, Biometris, Wageningen, The Netherlands; g Rijnstate Hospital, Clinical Research Department, Arnhem, The Netherlands; h Rijnstate Hospital, Department of Pediatrics, Arnhem, The Netherlands; i Department of Pediatric Endocrinology, Amalia Children's Hospital, Radboud Institute for Molecular Life Sciences, Radboud University Medical Center, Nijmegen, The Netherlands

**Keywords:** Childhood obesity, Collaborative community-based intervention, BMI-SDS, Health-related quality of life, Socioeconomic status, Multi-ethnicity

## Abstract

**Background:**

In most childhood obesity interventions, disadvantaged groups are underrepresented, and results are modest and not maintained. A long-term collaborative community-based approach is necessary to reach out to children from multi-ethnic backgrounds and achieve sustainable behavior change, resulting in sustained Body Mass Index-Standard Deviation Score (BMI-SDS) reductions. The objective is to determine the effects of GO! on BMI-SDS and Health-Related Quality of Life (HRQoL) for children and adolescents having overweight or obesity.

**Methods:**

A prospective, longitudinal cohort study was used to collect two-year follow-up data from November 2014 to July 2019. Children and adolescents (4-19 years old) from the low socioeconomic status and multi-ethnic district of Malburgen in the Dutch city of Arnhem were included. 178 children having overweight or obesity were recruited, with 155 children measured at baseline and after two years as a minimum, while 23 were lost to follow up. Participants attending the program for over six months were defined as completers (n=107) and participants attending the program for less than six months were defined as non-completers (n=48). The child health coach (CHC) acts as a central care provider in the collaborative community with healthcare providers from both medical and social fields. This coach coordinates, monitors and coaches healthy lifestyles, while increasing self-management for both children and parents. This is done in a customized and neighborhood-oriented manner and provided by all the stakeholders involved in GO!. The main outcomes are the change in BMI-SDS scores and HRQoL scores reported by participants.

**Findings:**

After 24 months, completers showed a decrease in BMI-SDS of -0·32 [95% CI: -0·42, -0·21], compared with -0·14 [95% CI: -0·29, 0·01] for non-completers (adjusted for gender and ethnicity; P=0.036). While 25% suffered from overweight and 75% from obesity at the start, following the intervention 5% showed normal weight, with 33% overweight and 62% with obesity. HRQoL reported by participants improved over time, showing no differences between completers and non-completers, gender and ethnicity after two years.

**Interpretation:**

Our results suggest that the GO! program might be effective in reaching out and reducing BMI-SDS for participants in a low socioeconomic status and multi-ethnic district over a two-year period. We noticed also trends to beneficial shifts in obesity grades. HRQoL improved regardless of the participation rate, gender and ethnic background. In light of the study limitations, further studies are needed to corroborate our observations.

**Funding:**

Dullerts-foundation, Nicolai Broederschap foundation, Burger en Nieuwe weeshuis foundation, Rijnkind foundation, Arnhems Achterstandswijken foundation, Menzis-foundation, the municipalities of Arnhem, Rheden, Overbetuwe and Lingewaard, the Association of Dutch municipalities, and Province of Gelderland.

**Research in Context**

**Evidence before this study**

A structured search showed that healthcare research and health services lately have increased interest in community coalitions, which are collaborations between various stakeholders in a community for joint action. However, despite progress in understanding what community coalitions are and how they work, little is known about the potential effectiveness and sustainability of this community-based approach for childhood obesity prevention interventions.

**Added value of this study**

Clinically relevant and accessible community-based interventions to prevent and treat childhood obesity involving low SES and multi-ethnicity minority groups are rare and clearly needed. To our knowledge, for the first time we have demonstrated that the novel, long-term collaborative community-based intervention program GO! is effective in significantly reducing BMI-SDS in children with overweight and obesity with a low SES living in a multiethnic district. Moreover, this reduction was accompanied by beneficial shifts in BMI grade.

**Implications of all the available evidence**

Our data show the potential and importance of the collaborative community-based intervention program GO! in combating and preventing childhood obesity particularly in areas with low SES and multi-ethnicity. Policy makers and intervention creators should consider the importance of a collaborative community focusing on 1) the individual level, 2) the community level, and 3) the neighborhood level when designing prevention programs. In light of the study limitations, further studies are needed to corroborate our observations.

Alt-text: Unlabelled box

## 1 Introduction

The childhood obesity epidemic is one of this century's most challenging public health issues [1]. To tackle this issue, a collaborative community-based approach covering both prevention and treatment is required [2,3]. Obesity is predominantly caused by an unhealthy lifestyle in which the increased intake of energy-dense food in combination with increased screen time, reduced physical activity and less sleep, play a pivotal role [4,5]. Besides an unhealthy lifestyle, demographic factors such as socioeconomic status (SES) and ethnicity are important factors in the etiology of childhood obesity [4,6,7].

A recent study in the Netherlands found that ethnic inequalities in obesity rates have widened since 2007, and these rates are generally higher among ethnic minority groups compared with Dutch children [8]. It is well known that these groups regularly face multiple problems [9]. As such, prevention and treatment programs should adapt to the situation of these disadvantaged groups [10]. Two systematic literature reviews demonstrate reductions in BMI-SDS, though generally small and measured over the short term [11,12]. Specifically, disadvantaged groups are underrepresented and consequently, programs do not reach their potential impact [13].

Healthcare research and health services have increased interest in community coalitions, which are collaborations between different stakeholders in a community for joint action. However, despite progress in understanding what community coalitions are and how they work, little is known about the potential effectiveness and sustainability of this community-based approach for childhood obesity prevention interventions. There is evidence demonstrating that creating collaborative, cohesive networks of (health) professionals can benefit the coordination of care and the quality of care. It has also been suggested that social support given by health professionals may have a limited effect, due to the nonreciprocal relationship between patients and professionals. However, the presence of a key player, like a health coach, who acts as a connector to transmit information, bridge groups and enables social and professional interaction is assumed to be vital. As such and in order to reach these groups, we developed the collaborative community-based program ‘Healthy on the Way!’ (‘Gezond Onderweg!’ (GO!)).

The GO! program identifies, recruits and treats children who are overweight or have obesity up to the age of 19 and their parents in the Netherlands. The three key items of GO! are 1) the collaborative community consisting of healthcare providers from both medical and social fields, 2) the child health coach (CHC), as the central care provider at the heart of this network, and 3) the neighborhood-oriented approach, involving all stakeholders in the direct environment of the participants and their parents. This research aimed to study the effect of the GO! program on long-term changes in BMI-SDS and HRQoL for children living in a low SES and multi-ethnic district.

## 2 Methods

### 2.1 Research design

This prospective, longitudinal cohort study was designed to analyze the effectiveness of the GO! program. Data were collected between November 2014 and July 2019. Anthropometric measurements of children who are overweight or have obesity were obtained within five different time periods: at baseline (T0), between 2 and 4.5 months (T3), 5 and 8 months (T6), 10 and 15 months (T12) and 20 and 28 months (T24) after baseline. Furthermore, children and parents were asked to complete the ‘Pediatric Quality of Life Inventory’ (PedsQL) about the children's HRQoL during these time periods [14].

### 2.2 Study population

#### 2.2.1 Eligibility

Children who are overweight or have obesity aged 4-19, living in the low SES and multi-ethnic district Malburgen in the Dutch city of Arnhem were allowed to join the GO! program. Inclusion criteria were children who are overweight or have obesity as defined by Cole et al. (2012), which is in accordance with the Dutch guidelines for Youth Health Care (YHC), general practitioners and pediatricians respectively. [15], [16], [17], [18] Participants who were measured at least at T0 and T24 were included in this study. Participants suffering from severe mental retardation (IQ less than or equal to 55), unknown endogenous problems where hypothalamic dysfunction was suspected, severe behavior problems and severe eating disorders requiring psychiatric treatment and those unable to understand the basics of the Dutch language were excluded.

#### 2.2.2 Population recruitment and sampling

**Figure 1:**
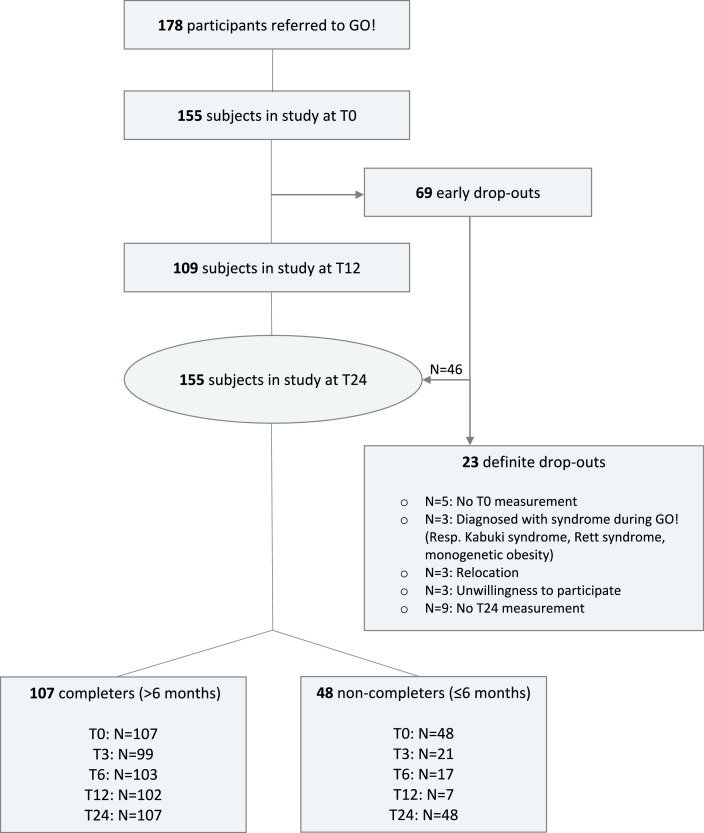
Flow chart GO! showing the follow-up of participants over two years of time. Two groups were formed ‘completers’ and ‘non-completers’.

178 children were recruited by referrals from local health professionals and self-referrals in the Malburgen district (Figure 1) [19]. 69 early drop-outs were recorded between T0 and T12. To complete this group's data, they were either invited to a T24 consultation or home visits were performed. 155 children (87.1%) were measured at T0 and T24, and 23 definite drop-outs (12.9%) were recorded because of multiple reasons (Figure 1). Participants attending the program for over six months with a mean of 15 consultations were defined as completers (n=107). Participants attending the program for only a limited period of ≤ six months (mean of three consultations), generally considered as inappropriate to result in health-related behavior changes, were defined as non-completers (n=48) [20]. In total, 113 children with obesity and 42 overweight children participated. It is estimated that 210 children with obesity live in the Malburgen district, which means GO! achieved an inclusion rate of almost 55% [21].

### 2.3 Description of GO! intervention

**Figure 2:**
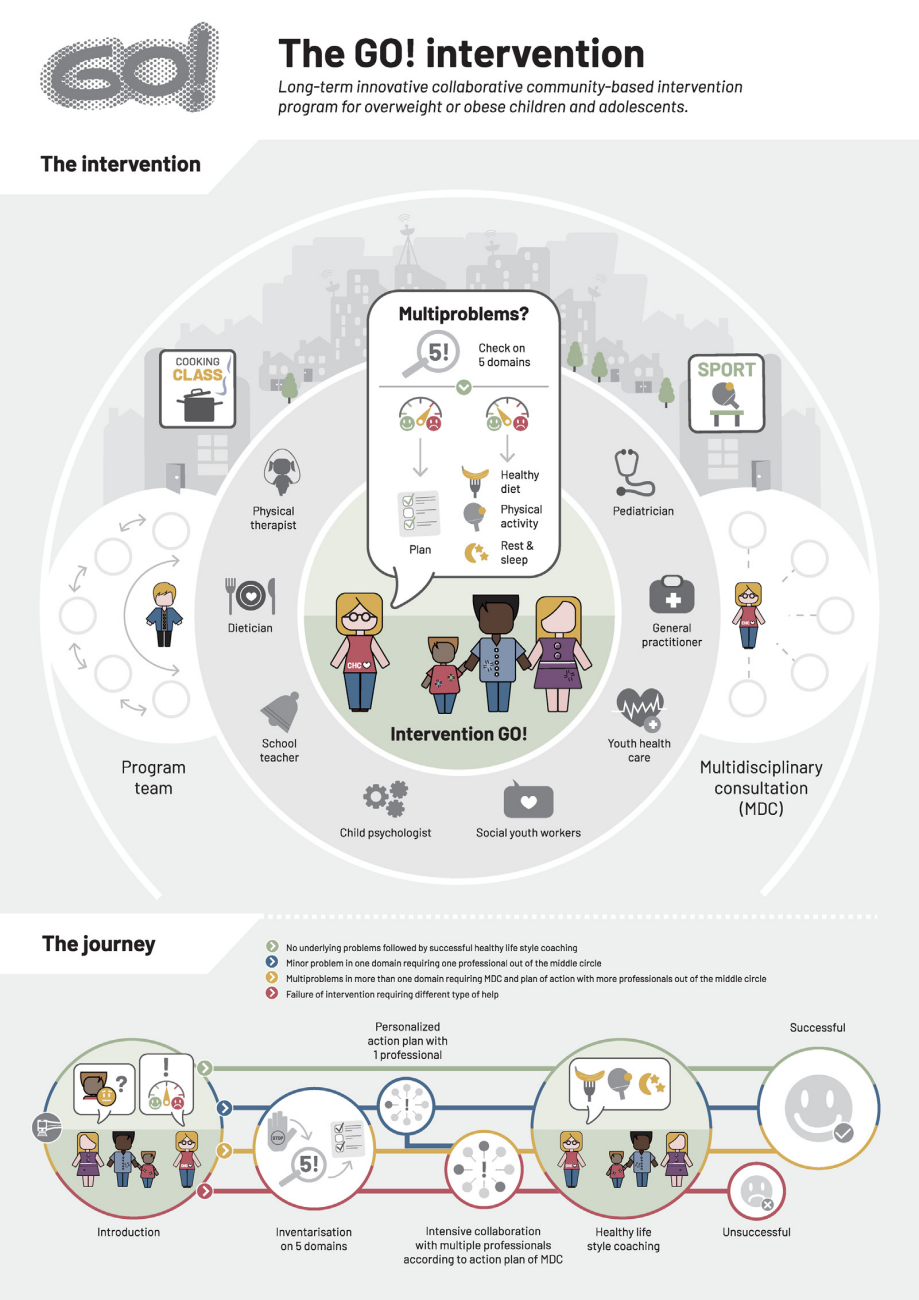
Description of the GO! intervention [53] *GO! schematically* The GO! approach focuses on three levels: a) participants and parents level (individual level): in which the child health coach (CHC) acts as a central care provider aiming to increase self-management of both participants and parents, b) community level: in which healthcare professionals from both the medical and social fields work closely together to offer “on demand” personalized help. The CHC coordinates this process, c) neighborhood level: in which stakeholders in the direct environment of the participants and their parents adapt and stimulate a healthy lifestyle and develop activities such as cooking workshops and physical activity programs aimed at improving healthy eating and physical activity. *Management and organization of GO!* GO! lasts for two years and is free of charge for all participants. The implementation of GO! in a new community starts with a stakeholder analysis in close collaboration with local policymakers to identify potential collaborators in the (para)medical and social fields, and locally embedded activities stimulating healthy behavior. Suitable professionals are selected and trained for the GO! program during three sessions: referral criteria, communication (“how to talk about obesity”) and treatment protocols of the different disciplines are discussed. Emphasis is laid on network orientation and collaboration. *Program team* In each community, a local ambassador, two local coordinators - preferably from the paramedical and social field - and a CHC are selected. Either a “child” mayor or public role model in the field of sport/cooking may fulfill the local ambassador role. The local coordinators are responsible for promoting GO! in the community and organizing program team meetings during which the professionals and community stakeholders discuss the progress and points for improvement. *Child health coaches* CHCs are specifically selected and trained for their communicative and coaching skills and are responsible for signaling and tackling underlying problems that are often associated with obesity and hinder the adoption of a healthy lifestyle. During the first consultation with the CHC, the CHC explains the program and interviews the participant and the parents extensively to identify multi-problems in five areas: child factors, parental factors, environmental factors, healthy lifestyle and coping strategies. Motivational interviewing techniques play a pivotal role in referring participants to personalized, desired help. Subsequently, the CHC develops an appropriate plan of action in collaboration with the participant and his/her parents and professionals in the middle circle, depending on what is needed. The CHC also monitors whenever the timing is right for healthy lifestyle coaching. *Lifestyle coaching* Itself is fully customized. The CHCs use the child friendly and appealing handbook [54] in which both the theoretical framework and practical tools for the topics healthy food, physical activity, rest and sleeping habits and cognitive behavior techniques are depicted. All consultations are held in the direct surrounding of the participants. At the start, the CHC meets face to face on a weekly basis, then every two weeks, then monthly and - if successful - quarterly. During the second year, the CHC monitors the maintenance of behavioral changes. *Multi-disciplinary consultation (MDC)* Complex cases that are unable to reach their healthy lifestyle goals, or where there is a suspicion of multi-problems, are discussed by the CHC according to a protocolled format during a multi-disciplinary consultation, preferably in the presence of the parents (four times a year). Underlying causal problems are mapped and a plan of action is made. After six months, evaluation takes place.

The description of the collaborative community-based program GO! is depicted in Figure 2. GO! is a collaborative community-based lifestyle intervention program for children and adolescents, in which (health)care providers from both the medical- and social field work closely together in order to coach children and their parents to a healthier lifestyle. These healthcare providers refer children who are overweight or have obesity to GO!. The CHC is the case manager and the midpoint of the GO! network and is responsible for signaling and tackling underlying problems that are often associated with obesity and hinder the adoption of a healthy lifestyle. Motivational interviewing techniques play a pivotal role in referring participants to personalized, desired help. Subsequently, the CHC develops an appropriate plan of action in collaboration with the participant and his/her parents and professionals, depending on what is needed. The CHC also monitors whenever the timing is right for healthy lifestyle coaching. The lifestyle coaching self is performed by the CHC and is fully customized. The CHCs use the child friendly and appealing handbook in which both the theoretical framework and practical tools for the topics healthy food, physical activity, rest and sleeping habits and cognitive behavior techniques are depicted. Since parents have a major influence on the lifestyle of their children, they will be included in the treatment program and are taught to become an exemplary role model. Cooking workshops and exercise activities are standard part of the program. A more extended explanation and information on organization of GO! as well as program team and multidisciplinary consultation can be found in the Figure Legends.

### 2.4 Outcome measures

#### 2.4.1 Socio-demographics and health characteristics

Socio-demographics and health characteristics of the child and parents were obtained by the CHC during the first consultation or with a telephone interview. The main characteristics of interest were the child's age, gender and ethnicity. Parents were asked to provide information about their weight, height and completed education level. Education level was classified in three categories based on the Dutch standard classification of education [22].

#### 2.4.2 Anthropometrics

Anthropometric parameters were determined by the CHC or the pediatrician. Weight was measured twice, while the subjects were wearing their clothes, but no shoes in kilograms (kg) to the nearest 0·1 kg using a class III-calibrated scale (Tanita BC 601). A stadiometer, calibrated in 0·1 cm intervals, was used to determine height, which was measured twice and averaged. BMI was calculated as the weight in kilograms divided by height in meters squared. BMI was adjusted for age and gender providing standardized BMI (BMI-SDS) scores using reference values from the Third Dutch National Growth Study [23,24. Overall, ≥ +1 BMI-SDS indicates overweight, and ≥ +2 BMI-SDS indicates obesity. Obesity grade 1,2 and 3 were defined as respectively adult equivalent BMI ≥ 30, 35 and 40 [25]. The change in BMI-SDS was defined as the primary outcome.

#### 2.4.3 Health-Related Quality of Life

Children and their parents were asked to fill in the PedsQL independently to assess HRQoL. This validated questionnaire consists of 23 questions about the physical- (eight items), emotional-, (five items) social- (five items), and school (five items) functioning of the child over the past month. These questions were answered on a five-point Likert scale ranging from ‘never’ to ‘almost always’ [14]. The overall psychosocial health score was the sum of the four domains. All items of the PedsQL were reverse-scored and linearly transformed to a 0-100 scale, in which a score of 100 refers to the highest possible HRQoL.

### 2.5 Statistical analysis

Data were digitally collected in a validated data management system called Research Manager. Statistical analysis was conducted using the statistical software SPSS (version 26·0) and SAS (version 9·4). Results were interpreted as significant when P<0.05 (two-sided). Descriptive statistics were used to describe the participants’ baseline characteristics. Mean differences of baseline characteristics between the completers and non-completers, and Western and Non-Western children were tested using an independent sample t-test for normally distributed data, a Mann-Whitney U test for not normally distributed data and a chi-squared test for categorical variables.

Differences in BMI-SDS changes over two years of time between the completers and non-completers were analyzed using a general linear regression model, with ethnicity and gender as covariates, followed by a t-test for comparisons of groups following ANOVA. BMI-SDS values at T3, T6, T12 and T24 were compared with T0 using a mixed linear model. In the completers group, time, gender and ethnicity were included with fixed main effects, as well as interaction of group with time and gender, and interaction of time with ethnicity, while participant-specific intercepts and slopes for time were included as random variables, and time-specific residual variances were included.

Differences in HRQoL changes over two years of time between the completers and non-completers were estimated by using independent t-tests. Reported HRQoL at T3, T6, T12 and T24 were compared with T0 using a generalized linear mixed model with overdispersed binomial distribution with logit link and participant-specific random intercepts. The fixed part of the model was identical to that of the model for BMI-SDS.

### 2.6 Ethical statement

Children and parents were informed about the study's goals and procedures, after which they were asked for informed consent to collect and analyze data. The procedures followed were in accordance with the ethical standards of the local feasibility committee, the Medical Research Involving Human Subjects Act (WMO) and the WMA Declaration of Helsinki (http://www.wma.net). The Medical Research Ethics Committee Arnhem-Nijmegen determined that this study did not fall within the remit of the Dutch “Medical Research Involving Human Subjects Act” (number 2014-1442).

### 2.7 Role of the funding source

The funders of the study had no role in study design, enrollment, data collection, data analysis, data interpretation or writing of the manuscript. DL, PvS, AH-N, LR and GG had full access to all data in the study. DL, PvS had final responsibility for the decision to submit for publication.

## 3 Results

### 3.1 Baseline characteristics

The mean age of the completers at baseline was 10.2 years old (SD 3·1) (Table 1). More girls (61·7%) and Non-Western participants (74·8%), predominantly Turkish and Moroccan, were represented in the completers group. The mean BMI-SDS was 3.0 (SD 0·7). At baseline, 26% of the completers were overweight; the majority were categorized as obesity grade 1 (39%), grade 2 (24%) or grade 3 (11%). Most fathers and mothers were overweight, with a respective BMI of 28·7 (SD 5·9) and 30.4 (SD 6·8) The completers and non-completers were comparable based on most parameters, except for gender and ethnicity.

**Table 1:** Baseline characteristics of GO! participants

	Completers (N=107)								Non-completers (N=48)							
Characteristics	N	Total		N	Western	N	Non-Western	P^a,d^	N	Total	N	Western	N	Non-Western	P^b,d^	P^c,d^
Age (years)	107		10.2 (3·1)	27	10·9 (3·4)	80	9·9 (3·0)	0·22	48	10·8 (4·2)	9	11·4 (3·9)	39	10·6 (4·3)	0·66	0·29
Gender, %	107			27				0·77	48		9				0·57	**0·01**
Female	66		61·7	16	59·3	50	62·5		27	56·3	6	66·7	21	53·8		
Male	41		38·3	11	40·7	30	37·5		21	43·8	3	33·3	18	46·2		
Ethnicity, %	107			27					48		9		39			**0·00**
Dutch	27		25·2	27	100	1			9	18·8	9	100				
Turkish	24		22·4			24	30·0		14	29·2			14	35·9		
Moroccan	17		15·9			17	21·3		6	12·5			6	15·4		
Different	39		36·4			39	48·8		19	39·6			19	48·7		
Number of consultations	107		14·9 (0·5)	27	14·9 (0·8)	80	14·9 (0·6)	0·22	48	3·0 (0·3)	9	3·2 (0·8)	39	3·0 (0·3)	0·66	0·29
BMI (kg/m^2^)	107		26·2 (4·6)	27	26·7 (4·4)	80	26·0 (4·7)	0·47	48	27·2 (6·2)	9	29·4 (7·3)	39	26·6 (5·9)	0·27	0·62
BMI-SDS	107		3·0 (0·7)	27	3·0 (0·6)	80	3·0 (0·8)	0·81	48	3·1 (0·7)	9	3·3 (0·8)	39	3·0 (0·7)	0·26	0·84
Obesity Class, %	107			27		80		0·73	48		9		39		0·11	0·77
Overweight	28		26·2	6	22·2	22	27·5		14	29·2	2	22·2	12	30·8		
Obesity 1	42		39·3	11	40·7	31	38·8		17	35·4	1	11·1	16	41·0		
Obesity 2	26		24·3	8	29·6	18	22·5		14	29·2	5	55·6	9	23·1		
Obesity 3	11		10·3	2	7·4	9	11·3		3	6·3	1	11·1	2	5·1		
BMI father (kg/m^2^)	89		28·7 (5·9)	21	31·6 (8·4)	68	27·8 (4·6)	0·06	35	28·1 (4·4)	7	31·8 (5·8)	28	27·2 (3·5)	**0·01**	0·55
BMI mother (kg/m^2^)	101		30·9 (6·8)	23	32·1 (9·2)	78	30·5 (5·9)	0·33	41	30·4 (6·6)	7	29·9 (6·4)	34	30·5 (6·8)	0·83	0·70
Education level mother, %								0·45							0·58	0·30
Low	23		28·4	4	19	19	31·7		6	20·7	1	16·7	5	21·7		
Medium	49		60·5	15	71·4	34	56·7		18	62·1	5	83·3	13	56·5		
High	9		11·1	2	9·5	7	11·7		5	17·2			5	21·7		
HRQoL - total, child	77		74·6 (12·8)	17	65·7 (12·8)	60	77·2 (11·8)	**0·00**	32	73·4 (16·2)	3	79·0 (9·2)	29	72·8 (16·8)	0·58	0·95
HRQoL - physical functioning, child	77		76·9 (14·7)	17	73·0 (14·7)	60	78·0 (14·6)	0·30	32	76·8 (16·5)	3	86·5 (12·6)	29	75·8 (16·7)	0·33	0·77
HRQoL - psychosocial functioning, child	77		73·4 (15·1)	17	61·7 (13·0)	60	76·7 (14·0)	**0·00**	32	71·6 (19·0)	3	75·0 (13·0)	29	71·2 (19·6)	0·90	0·99
HRQoL - total, parent	78		74·4 (13·6)	17	68·5 (13·5)	61	76·0 (13·3)	**0·05**	32	71·2 (16·9)	3	62·3 (10·1)	29	72·1 (17·3)	0·23	0·46
HRQoL - physical functioning, parent	78		74·0 (18·0)	17	71·9 (15·7)	61	74·6 (18·7)	0·50	32	75·7 (17·8)	3	74·0 (20·8)	29	75·9 (17·8)	0·90	0·65
HRQoL - psychosocial functioning, parent	78		74·5 (14·3)	17	66·7 (14·4)	61	76·7 (13·6)	**0·01**	32	68·7 (18·9)	3	56·1 (6·3)	29	70·0 (19·4)	0·21	0·22

a: P-value for the difference between Western and Non-Western children in the completers. b: P-value for the difference between Western and Non-Western children in the non-completers. c: P-value for the difference between the totals of the completers and non-completers. d: Independent t-test, Mann-Whitney U test, or Chi-squared test. Values have been reported as mean (SD), unless otherwise stated.

### 3.2 Differences in BMI-SDS after 24 months

After 24 months, children in the completer group showed a significant decrease in BMI-SDS of -0·23, compared with -0·05 for the non-completers group (p=0.049) (Table 2). Adjustment for gender and ethnicity resulted in a BMI-SDS of -0·32 in the completers, compared with -0·14 in the non-completers (p=0·036). After 24 months, a considerable shift in BMI grades was observed (Figure 3a + 3b). Whereas at start, no normal weight was observed in the completers and respectively ¼ and ¾ of the participants suffered from overweight and obesity, after the intervention 5% showed a normal weight, 33% overweight and 62% obesity. 41 children (38·3%) from the completers group reached a lower BMI-grade, 59 children (55·1%) remained stable in their BMI-grade and seven children (6·5%) reached a higher BMI-grade. In the non-completers group, nine children (18·8%) showed a decrease in BMI-grade, 28 children (58·3%) did not show any change, and 11 children (22·9%) showed an increase in BMI-grade.

**Table 2:** Changes in BMI-SDS of GO! participants in completers and non-completers from baseline to 24 months

	N	Completers	P^a,d^	N	Non-completers	P^b,d^	P^c,d^
BMI-SDS (unadjusted)	107	-0·23 [-0·33, -0·13]		48	-0·05 [-0·20, 0·09]		**0·049**
BMI-SDS adjusted for gender and ethnicity	107	-0·32 [-0·42, -0·21]		48	-0·14 [-0·29, 0·01]		**0·036**
BMI-SDS split by gender and adjusted for ethnicity			**0·01**			**0·01**	
Girls	66	-0·18 [-0·31, -0·05]		27	0·05 [-0·14, 0·24]		**0·04**
Boys	41	-0·45 [-0·61, -0·30]		21	-0·34 [-0·56, -0·12]		0·40
BMI-SDS split by ethnicity and adjusted for gender			**0·03**			0·34	
Western	27	-0·44 [-0·63, -0·26]		9	-0·21 [-0·53, 0·11]		0·22
Non-Western	80	-0·20 [-0·31, -0·09]		39	-0·04 [-0·19, 0·12]		0·08

Values have been reported as mean difference (95% CI).

a: P-value for the difference between the different genders and ethnicities of children in the completers.

b: P-value for the difference between the different genders and ethnicities of children in the non-completers.

c: P-value for the difference between the totals of the completers and non-completers.

d: General linear regression model with ethnicity and gender as covariates, followed by a t-test for comparisons of groups following ANOVA

**Figure 3a:**
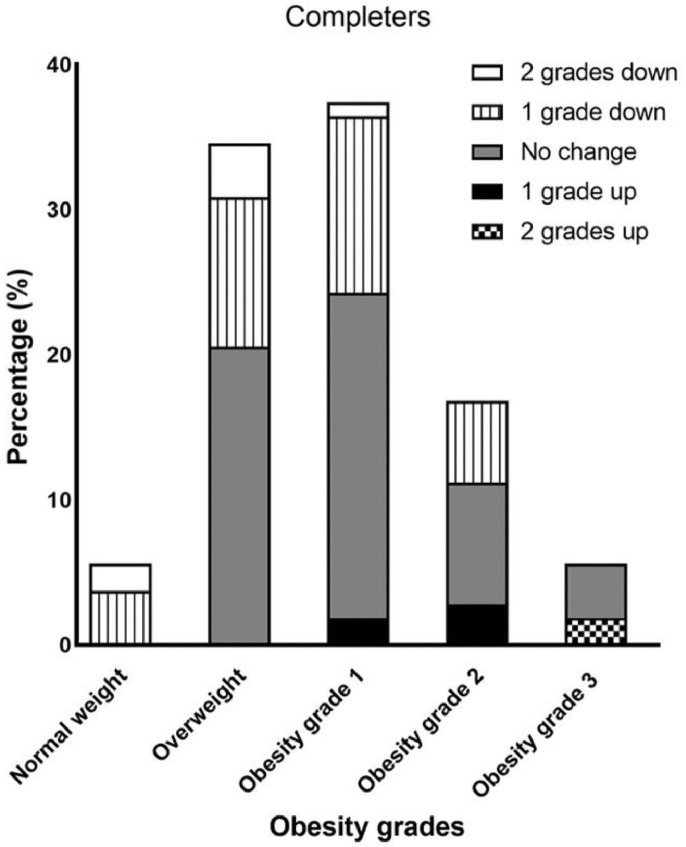
Shifts in obesity grades of the completers at T24, compared to T0. Overweight is defined as an adult equivalent BMI≥25 and obesity grade 1,2,3 as respectively adult equivalent BMI ≥ 30, 35 and 40. 1 grade up means for example a shift from overweight to grade 1 or a shift from grade 1 to obesity grade 2.

**Figure 3b:**
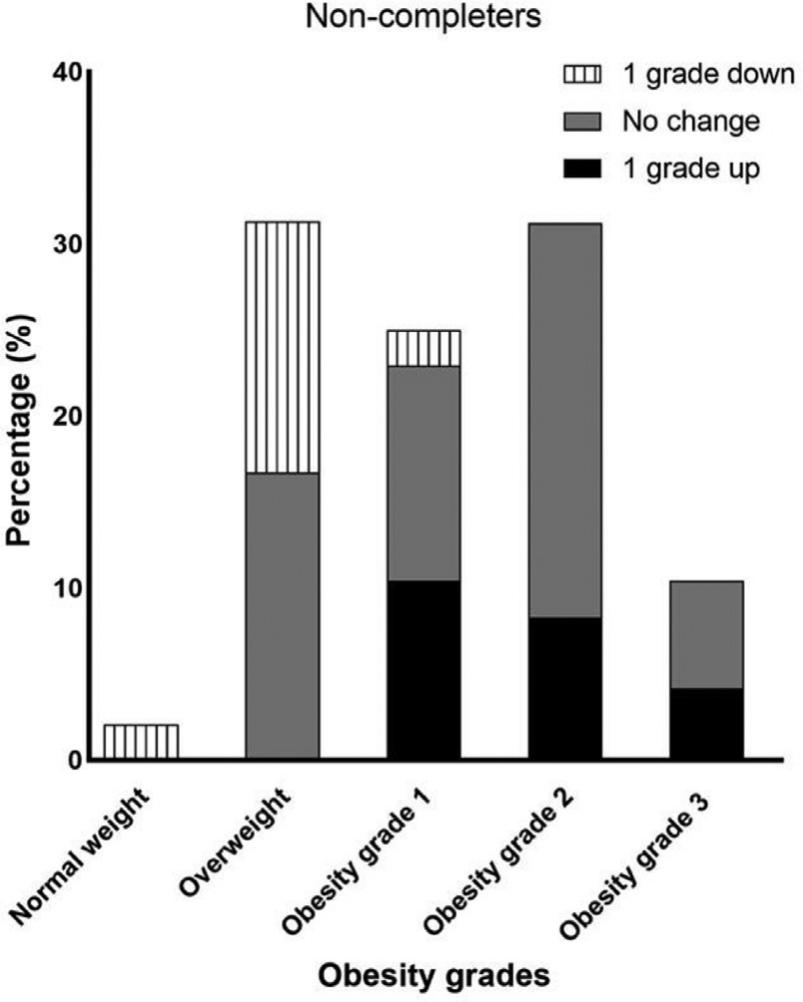
Shifts in obesity grades of the non-completers at T24, compared to T0. Overweight is defined as an adult equivalent BMI ≥25 and obesity grade 1,2,3 as respectively adult equivalent BMI ≥ 30, 35 and 40. 1 grade up means for example a shift from overweight to grade 1 or a shift from grade 1 to obesity grade 2.

Western completers showed a significantly larger reduction in BMI-SDS compared with Non-Western completers (respectively -0·44 and -0·20 BMI-SDS) (Table 2), while boys saw a significantly larger reduction in BMI-SDS than girls over two years (respectively -0·18 and -0·45 BMI-SDS). In Figure 4a predicted BMI-SDS means from the mixed linear model, together with their standard errors are presented. Results are shown for all five timepoints, split by gender and completion group. Per subgroup pairwise comparisons of timepoints with T0 resulted in significant differences for boys in the completers group (-0·34; P=0·001).

**Figure 4a:**
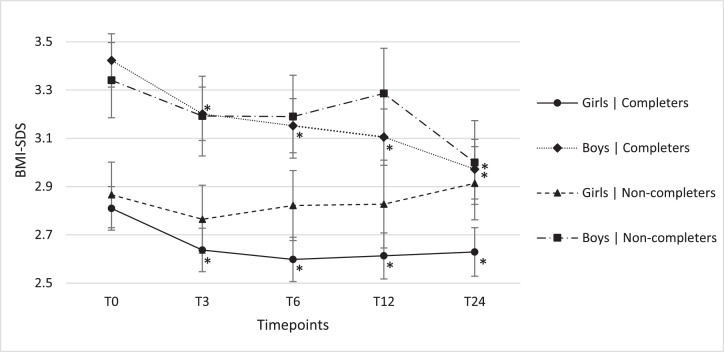
BMI-SDS values presented over time for girls and boys in the completers and non-completers, including the standard error. Results are presented as predicted means plus standard errors based upon the fitted mixed linear model for BMI-SDS. All values are adjusted for ethnicity. *Significantly different compared to the measurement at 0 months (T0) (p<0.05).

### 3.3 Differences in HRQoL after 24 months

Figure 4b shows predicted means for quality of life, based upon the genera1lized linear mixed model, together with their approximate standard errors. Results are shown for all 5 timepoints, split by gender and completion groups. Per subgroup pairwise comparisons of timepoints with T0 resulted in significant differences for boys and girls in the completers group at T6, T12 and T24. HRQoL reported by completers and non-completers both increased over time (Table 3, Figure 4b). HRQoL improvements did not differ significantly between boys and girls, or between Westerns and Non-Westerns, and completers and non-completers after two years.

**Figure 4b:**
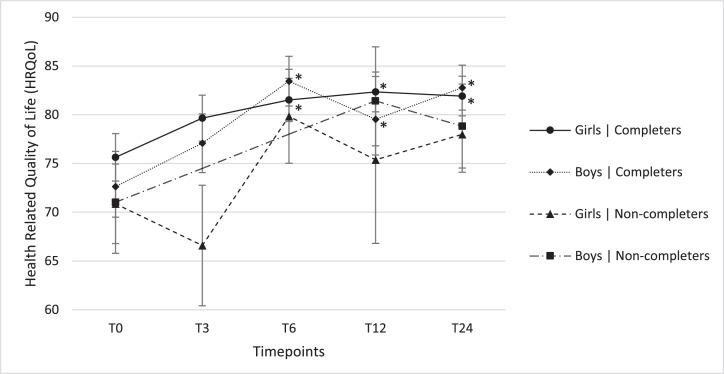
Reported total Health Related Quality of Life by girls and boys in the completers and non-completers at five different timepoints. Results are presented as predicted means plus standard errors based upon the fitted generalized linear mixed model for HRQoL. For boys in the non-completers group no data was available at T3 and T6. *Significantly different compared to the measurement at 0 months (T0) (p<0.05).

**Table 3:** Changes in reported health-related quality of life of Western and Non-Western boys and girls in the completers and the non-completers from baseline to 24 months

		Completers				Non-completers	
		Total(N=27|43)	Western(N=7|9)	Non-Western (N=20|34)	P^a,c^	Total (N=9|13)	P^b,c^
**Total score**	Boys	9·1 [4·8, 13·3]	10·6 [1·5, 19·7]	8·5 [3·3, 13·8]	0·67	10·9 [-7·2, 29·0]	0·75
	Girls	5·8 [2·9, 8·8]	5·7 [-5·7, 17·1]	5·9 [3·2, 8·6]	0·97	6·7 [-5·0, 18·4]	0·88
	Total	7·1 [4·7, 9·5]	7·8 [1·1, 14·6]	6·9 [4·4, 9·4]	0·74	8·4 [-0·8, 17·6]	0·78
**Physical functioning**	Boys	11·4 [7·0, 15·9]	11·1 [0·7, 21·5]	11·6 [6·2, 16·9]	0·93	5·9 [-10·0, 21·7]	0·31
	Girls	5·2 [1·0, 9·3]	3·1 [-6·9, 13·1]	5·7 [0·9, 10·5]	0·62	1·1 [-10·3, 12·5]	0·39
	Total	7·6 [4·5, 10·7]	6·6 [-0·1, 13·3]	7·9 [4·3, 11·5]	0·74	3·0 [-5·5, 11·6]	0·31
**Psychosocial functioning**	Boys	8·1 [3·1, 13·0]	11·0 [1·79, 20·11]	7·0 [0·8, 13·4]	0·49	13·4 [-6·9, 33·8]	0·42
	Girls	6·2 [2·9, 9·5]	7·0 [-5·4, 19·5]	6·0 [2·8, 9·1]	0·86	9·7 [-4·7, 24·0]	0·62
	Total	7·0 [4·2, 9·6]	8·8 [1·5, 16·0]	6·4 [3·4, 9·3]	0·47	11·2 [0·4, 22·0]	0·43

a: P-value for the difference between Western and Non-Western children of the completers.

b: P-value for the difference between the total completers and non-completers.

c: Independent sample t-test

Values are reported as mean difference [95% CI].

N= Boys|Girls

## 4 Discussion

Childhood obesity is associated with an increased prevalence of cardiometabolic risk factors [26]. Since obesity during childhood also increases the risk of prolonged obesity in adulthood, focus has shifted to not only prevention, but also treating obesity early in life [27]. The long-term results of combined lifestyle interventions for children and adolescents are generally disappointing. Adjusted analyses in our completers group showed a significant reduction in BMI-SDS of -0·32. This largely exceeded the widely used cut-off value of -0·25 BMI-SDS for effective and clinically relevant interventions [28], [29], [30]. It is also more beneficial than the average results of outpatient childhood obesity programs as documented by recent Cochrane systematic reviews reporting BMI-SDS reductions ranging from -0·03 to -0·20 [11,12. The study populations, design and duration of the interventions in these reviews are rather diverse, impeding a realistic comparison with our study. The study of Reinehr et al. (2007) however reported a BMI-SDS reduction of -0·41 two years from baseline [31]. This study included participants who had proven their motivation to take part in the program, whereas in GO! there were no motivation requirements. Van de Baan et al. (2014) investigated the effect of an inpatient treatment program and reported significant reductions in BMI-SDS of 0·5 and 0·29 after 12 and 18 months respectively [32]. Compared with GO!, these results could not be sustained over the long term.

Three critical pathways to effectiveness reviewed by Burchett et al. (2017) are fulfilled in the GO! program [33]. Key elements are the collaborative community with healthcare providers from both the medical- and social fields, the CHC as healthy lifestyle coach in the heart of this network, and the neighborhood-oriented approach. To our knowledge, for the first time we have demonstrated that a collaborative community-based intervention program is effective in significantly reducing BMI-SDS in a district with low SES and multi-ethnicity. Moreover, this reduction was accompanied by beneficial shifts in BMI grade, potentially reducing the risk of obesity in adulthood as well as comorbidities.

The difference in BMI-SDS reduction between the GO! completers and non-completers at two year-follow up was -0·18 BMI-SDS. Two Cochrane reviews reported a mean difference in BMI-SDS reduction between intervention and control group of -0.06 [95% CI: -0·10, -0·02] for children aged six to 11, and a mean difference in BMI-SDS reduction between intervention and control group of -0·13 [95% CI: -0·21, -0·05] for adolescents aged 12 to 17 [11,12. The reduction in BMI-SDS in our GO! program [-0.18] exceeds the mean difference values of these reviews, and indicates that the GO! approach is effective, particularly given our high baseline BMI-SDS and multi-ethnicity. The non-significant difference in BMI-SDS reduction between boys in the completers group and boys in the non-completers group is mainly due to the positive results of the non-completers. Reason for this is that a few non-completers interrupted the program because they were able to change their lifestyle with parental help. These boys flattered the data and are not representative for the whole group of non-completers.

In our study, Western (25%) and Non-Western (75%) participants both improved their BMI-SDS. However, Western participants achieved greater BMI-SDS reductions compared with Non-Westerns (-0·44 versus -0·20 for completers, -0·21 versus -0·04 for non-completers). Most interventions targeting children from multi-ethnic groups are unable to report a significant reduction in BMI-SDS [34], [35], [36], [37], [38]. A recent intervention among low socioeconomic status children was unable to reduce BMI gain [39]. Some interventions report maximum reductions of BMI-SDS in these groups of -0·16 (61·9% Non-Westerns), -0·18 (22·7% Non-Westerns) and -0·19 (100% Non-Westerns). [2,^40^,41]

Furthermore, we observed a substantial difference in BMI-SDS reduction between boys and girls in both the completers group (-0·27) and the non-completers group (-0·30). This is in accordance with the widely implemented MEND intervention, reporting a difference in reduction of -0·23 BMI-SDS between boys and girls at 2·4 years from baseline [42]. They assumed that the low physical activity level of girls was the cause of the BMI-SDS relapse in this group. This assumption is supported by studies showing that boys in general are more physically active and experience less difficulties in increasing their physical activity and decreasing sedentary time compared with girls [29,43,44]. We hypothesize that girls in the GO! program were less physically active, which may explain why their results lag behind those of boys. Although both boys and girls reach a significant BMI-SDS reduction over time, in the future a more gender-specific approach is advised. Whereas we did notice beneficial shifts in BMI grade, in accordance with literature we observed that children with a higher level of overweight (obesity grade 2 and 3) were more resistant [45].

Children and adolescents with obesity are at risk of developing significant impairments in quality of life. It is well described that baseline QoL scores are lower in these children [46]. A mean QoL score of 67 is reported in a cohort of children with obesity, compared with a score of 83 for healthy children. Our completers’ intermediate mean scores of 74·6 and 73·4 for Westerns and Non-Westerns were reported at baseline. During the intervention period, an average improvement in HRQoL of 7·1 points was found, exceeding the results of previous interventions, although lower than the improvement in HRQoL reported by Hoedjes et al. (2018) [47,48. In the latter study, the intervention consisted of an inpatient treatment period for participants with severe obesity, which might have influenced their results positively [49]. Non-completers showed similar improvements in HRQoL. This is remarkable since it was hypothesized that HRQoL improves when BMI-SDS reduces. Accordingly, the study of Finne et al. (2013) showed that improvements in HRQoL were not consistently bound to weight reduction [50]. This suggests that other factors are influencing the improvement in HRQoL for the non-completers. One hypothesis is that participants tend to change their behavior because they are the target of special interest in a study, also known as the Hawthorne effect [51]. Unfortunately, our findings cannot be compared with other studies, since studies measuring HRQoL in control groups are scarce.

Despite the equal improvements reported by the completers and non-completers, by Western and Non-Western participants, and by boys and girls, the significant improvements in HRQoL reported by children at six months from baseline were maintained during the next 1·5 years by the completers. It is well known that measures based on self-reporting have limitations, such as potential recall bias, reporting inaccuracies due to other factors such as feeling ashamed for not reaching set goals, poor adherence or wanting to answer in ways that are thought to be desired. This may have flattered the HRQoL scores. Overall, our results seem to confirm the tendency that the GO! program is able to improve HRQoL over the long term.

This study has several strengths. Firstly, the unique intervention design in which healthcare providers from both medical and social fields closely collaborate in the community, which enables a high inclusion rate for both Western and non-Western participants and low drop-out levels. Secondly, in our study, a fairly large group of 150 children and adolescents with an average BMI-SDS of 3·0 were longitudinally followed up for two years, providing long-term outcomes. Thirdly, the aspect of multi-ethnicity is well represented in our study and shows that the program is also potentially effective in Non-Westerns. Since the specific population in this study is usually less responsive compared to the general one, we hypothesize that applying the same treatment approach used in this study to the general population, should obtain even better results, making this study potentially generalizable [10].

This is even more relevant seen in the light of significantly increasing prevalence of morbid obesity in Dutch children over time. van Dommelen et al described this in their paper based on three national Dutch Growth Studies performed in 1980, 1997 and 2009 (n= 54,814) [52].

This study has several potential limitations. Firstly, neither BMI-SDS nor BMI are ideal outcome measures. However, the BMI-SDS was the best measure available and practical applicable for capturing changes in body fatness. Secondly, we did not randomize the children to either an intervention or control group and we included a surrogate control group. Stakeholders considered it ethically incorrect to assign half of the children with obesity to a control group. Alternatively, a comparison group was reconstructed consisting of non-completers. Although the non-completers cannot be considered as a group with similar characteristics to completers. In our opinion these groups give respectively a good representation of children that are able to persist and improve their lifestyle on the one hand, and on the other hand, children that were not able to proceed. The latter group of non-completers offering the best possible representation of a control group.

It is plausible that the GO! program is effective in reducing BMI-SDS for participants in a low SES and multi-ethnic district over a two-year period. We noticed beneficial shifts in obesity grades. HRQoL improved regardless of the participation rate, gender and ethnic background. A collaborative community approach makes it possible to reach out to a multi-ethnic population and achieve positive results. In light of the study limitations, further studies are needed to corroborate our observations. Future research should focus on a better understanding of the observed differences in Western versus Non-Western children, gender and different aspects of HRQoL in order to further optimize GO!

## 5 Contributors

PvS conceived of and designed the study. DL, GG and LR took responsibility for the integrity of the data and the accuracy of the statistical data analysis. DL produced all figures and tables. PvS, DL, SZ, and SS-M performed the data collection. DL, PvS and AH-N co-wrote the initial draft. DL, PvS, AH-N, LR and GG had full access to all data in the study. and have verified the data underlying the study. All authors edited the manuscript, provided feedback on the study, and approved the final manuscript.

## Declaration of Competing Interest

We declare no competing interests.

## Appendix Supplementary materials

Image, application 1

Image, application 2
