# Supplementary material for: Two-year effects of the community-based overweight and obesity intervention program Gezond Onderweg! (GO!) in children and adolescents living in a low socioeconomic status and multi-ethnic district on Body Mass Index-Standard Deviation Score and quality of life
Source: eClinicalMedicine. 2021 Nov 30;42:101217. doi: 10.1016/j.eclinm.2021.101217 (PMC8640234; doi:10.1016/j.eclinm.2021.101217)
Supplement: Supplementary file 1 [file mmc1.pdf]

GO! GEZOND ONDERWEG

Het GO! Handboek voor Kindergezondheidscoaches

Wegwijs in het gebruik van alle standaard consulten!

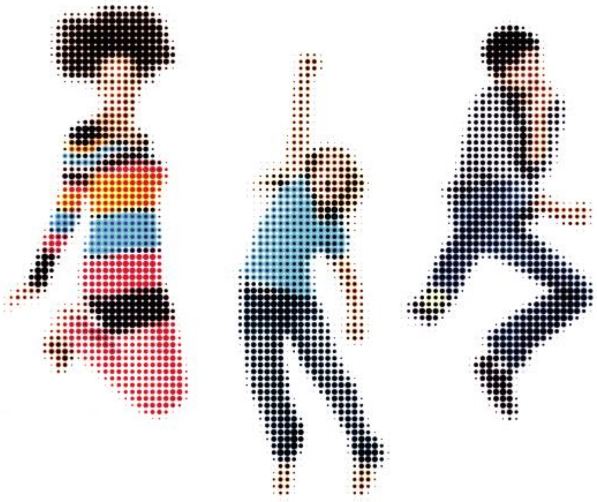

GO! Gezond Onderweg
2018

Marjolein Postma
Petra v Setten

Copyright © 2018
Auteurs Marjolein Postma, Petra van Setten

Uitgegeven door Stichting Rijnstate / GO! Gezond Onderweg
ISBN 978-90-9031404-4
NUR 100

Niets uit deze uitgave mag worden verveelvoudigd, door middel van druk, fotokopieën, geautomatiseerde gegevensbestanden of op welke andere wijze ook zonder voorafgaande schriftelijke toestemming van de auteur.

Inhoudsopgave

Inhoud

Inhoudsopgave 3
Inleiding 5
Aanmelding en het maken van de eerste afspraak. 7
Het intake-gesprek & Uitleg Bloedsuikerspiegel 9
Consult 1: BMI & Volkoren Producten 38
Consult 2: Groente en fruit 43
Consult 3: Het Ontbijt & Water 51
Consult 4: Het stoplicht 59
Consult 5: Een terugblik en waar staat het kind/jongere nu? 62
Consult 6: Motivatie voor leefstijlverandering en visualisatie 65
Consult 7: Sporten en bewegen 75
Consult 8 Het belang van voldoende slaap en ontspanning voor een gezonde leefstijl 84
Consult 9 Verdieping van de voedingsstoffen, bouwstoffen en brandstoffen. 89
Gezond eten=voeding 89
Consult 10: Etiketten lezen en E-nummers 97
Consult 11: Alles op een rijtje 101
Consult 12: Afsluiting GO! Gezond onderweg 103
Extra Consulten 105
Literatuurlijst 139
Materialen ter ondersteuning van consulten 140
Bijlage 1 Stroomschema voor verwijzing en ketensamenwerking, MDO en Bloeddruktabel 141
Bijlage 2 Gewichtsverloopkaart jongens 142
Bijlage 3 Gewichtsverloopkaart meisjes 143
Bijlage 4 Aanbevolen portiegroottes voor een kind ontbijt 144
Bijlage 5 Aanbevolen portiegroottes voor een kind lunch 145
Bijlage 6 Aanbevolen portiegroottes voor een kind warme maaltijd 146
Bijlage 7 Aanbevolen portiegroottes voor een kind tussendoor/toetjes 147
Bijlage 8 Aanbevolen portiegroottes voor een kind en volwassene warme maaltijd 148

Bijlage 9 Gezonde en minder gezonde dranken 149
Bijlage 10 Gezonde en minder gezonde dranken (suikerkaart) 150
Bijlage 11 Voorbeelden van kinderporties van niet-Schijf van Vijf producten 151
Bijlage 12 Beweegplanner 152
Bijlage 13 Beweegkaart 153
Bijlage 14 Schijf van vijf 154
Bijlage 15 BMI-tabel jongens 2 tot 18 jaar 155
Bijlage 16 BMI-tabel meisjes 2 tot 18 jaar 157
Bijlage 17 Voorbeeld groeidiagram jongens 1-21 NL 159
Bijlage 18 Uitleg Pedsql vragenlijsten 160
Bijlage 19 Terugkoppeling voortgang kind/jongere 161
Bijlage 20 Eetdagboek voor een week 162

Inleiding

Dit handboek is geschreven voor kindergezondheidscoaches, die gaan werken met kinderen en jongeren, die verwezen zijn naar het programma GO! Gezond Onderweg[1]. De doelgroep bestaat uit kinderen tussen de 0 en 19 jaar met overgewicht en obesitas graad I, II en III (met of zonder co morbiditeit of risicofactoren), inclusief de gezinsleden. Het ketenzorgprogramma GO! is een geprotocolleerd multidisciplinair en wijk[2] gericht behandelprogramma voor de signalering en behandeling van kinderen met overgewicht en obesitas. Er wordt in dit programma samengewerkt met alle zorgverleners, zorgaanbieders, leerkrachten en begeleiders rondom het kind/jongere en het gezin in zijn of haar wijk. De kindergezondheidscoach is de casemanager. Het behandelprogramma beslaat 2 jaar.

It Takes A Village to Raise a Child.

Het programma GO! heeft als visie om aan te sluiten bij de beleving en motivatie van het kind/jongere en zijn/haar omgeving. Hierdoor kan er zorg "op maat" verleend worden. Het programma GO! is een totale leefstijl begeleiding met als doel een blijvende gedragsverandering in de leefstijl. De leefstijl begeleiding bestaat uit drie pijlers: gezond & lekker eten, voldoende sport & beweging en genoeg rust & ontspanning. Het programma GO! gaat uit van de kracht van het kind, de motivatie van het kind/jongere en ouders/verzorgers is hierbij leidend. Als kindergezondheidscoach monitor je deze motivatie (en de daar aan grondslag liggende determinanten) gedurende het gehele programma. De werkwijze gaat uit van het bevorderen van de positieve gezondheid[3] door middel van de zes dimensies[4] van gezondheid. Er is een positieve benadering naar het kind/jongere en zijn/haar omgeving toe, waarbij GO! Gezond Onderweg gelooft dat kinderen vanuit hun eigen kracht gezonde keuzes kunnen maken – een leven lang.
Het programma GO! vindt het belangrijk dat alle kindergezondheidscoaches dezelfde aanpak hanteren waarbij er ruimte is voor een eigen professionele inbreng van de kindergezondheidscoach. Het handboek is hiervoor als leidraad ontwikkeld. De effectiviteit van het programma GO! wordt wetenschappelijk onderzocht door het ziekenhuis Rijnstate in samenwerking met Wageningen University & Research (WUR). Het programma is (gedeeltelijk) gestoeld op het A(ttitude) S(ociale invloed) E(eigen-effectiviteitsverwachting) model (De Vries, Dijkstra & Kuhlman, Kok, Mudde&Stretcher).

[1] Voor de leesbaarheid van dit boek zal GO! Gezond Onderweg afgekort worden tot GO!
[2] Voor de leesbaarheid van dit boek zal de term "wijk" gebruikt worden als het over het gebied gaat waar GO! actief is. Hiervoor kan ook gelezen worden kern, stad, dorp of gemeente.
[3] GO! sluit hierbij aan bij het gedachte goed van Machteld Huber.
[4] Zes dimensies: lichaamsfuncties, mentaal welbevinden, zingeving, kwaliteit van leven, sociaal maatschappelijke participatie en dagelijks functioneren.

Leeswijzer
In dit handboek is het programma GO! in 12 standaard consulten beschreven. Per consult is het doel vastgesteld. De werkwijze van het consult wordt beschreven, inclusief de uitwerking van de vragen die op de consulten staan. Tevens zijn de kaders van het programma GO! vastgesteld en beschreven. Het kind/jongere krijgt tijdens de intake een eigen werkboek. Hierin staan werkbladen die het kind/jongere tijdens het consult samen met de kindergezondheidscoach invult of thuis zelf invult. In de bijlagen zijn extra documenten toegevoegd die een hulpmiddel kunnen zijn bij de consulten. Daarnaast zijn er extra consulten ontwikkeld die te gebruiken zijn voor verdieping op de bestaande consulten of voor speciale momenten in het jaar zoals bijvoorbeeld kerstmis, sinterklaas, vakantietijd of de ramadan.
De leeswijze van het handboek is als volgt ingedeeld: op de linker pagina staat een werkbeschrijving van het consult voor de kindergezondheidscoach en op de rechterpagina staat het werkblad voor het kind/jongere en de ouder/verzorger.

Dit boek is mede mogelijk gemaakt door GO! Gezond Onderweg.
Kinderarts Petra van Setten heeft medisch inhoudelijk meegelezen. Zij is de founding father van GO! en medisch-inhoudelijk programmamanager van GO!

De consulten die de kindergezondheidscoach gebruikt zijn gebaseerd op de methode van Plan-Fit, welke is ontwikkeld door Marjolein Postma. Zij is Senior Kindergezondheidscoach, opleider en mede initiatiefneemster van GO!.

Aanmelding en het maken van de eerste afspraak.

Doel
Beoordelen of een verwijzing doorgezet kan worden naar een eerste afspraak.

Werkwijze
Er wordt gewerkt met een stroomdiagram (zie bijlage 1) voor de verwijzing van het kind/jongere naar GO!. Via dit stroomdiagram kun je als kindergezondheidscoach zien of het kind/jongere voldoet aan de criteria voor deelname aan GO!.
Een kind of jongere kan op de volgende manieren aangemeld worden bij het programma GO!:

- Verwijzing door jeugdarts, huisarts of kinderarts.
- Verwijzing door paramedicus (bijvoorbeeld fysiotherapeut, diëtist, wijkcoach/jeugdconsulent).
- Een kind/jongere meldt zichzelf aan. De kindergezondheidscoach controleert of het kind aan de juiste inclusiecriteria voldoet volgens het stroomdiagram.
  De inclusiecriteria zijn:
  1) Overgewicht, waarbij de jeugdarts het kind/jongere in het overbrugginsplan gezien heeft zoals beschreven in het stroomdiagram (bijlage 1).
  2) Er is met de BMI-tabel (bijlage 15 en 16) vastgesteld dat het kind/jongere obees is.

Wanneer het kind/jongere niet verwezen is door een arts dan moet het kind/jongere altijd nog één keer gezien worden door de eigen huisarts. De reden hiervoor is om eventuele medische problematiek te ondervangen. De kindergezondheidscoach overlegt met de ouder/verzorger dat deze afspraak, op korte termijn, gemaakt wordt met de huisarts.

Het maken van de eerste afspraak

Doel
Informeren van het kind/jongere en de ouder/verzorger over de inhoud, duur, deel 1 intake en wetenschappelijk onderzoek van GO!.
Afspraak maken voor het intakegesprek.
Verifiëren of de reden van verwijzing duidelijk is bij het kind/jongere en de ouder/verzorger.

Werkwijze
Wanneer een aanmelding binnenkomt maak je telefonisch contact met de ouder en/of kind/jongere.
Tijdens dit telefonische gesprek geef je informatie over het programma GO!. Je geeft uitleg over wat het programma inhoudt. Je vertelt dat de afspraken in principe met het kind/jongere en de ouder/verzorger samen zijn. Je legt het verloop van afspraken/consulten uit. De consulten zijn de eerste drie weken elke week. Vervolgens zijn de consulten twee weken om de twee weken. Als het goed gaat breid je uit naar één keer per maand. Dit doe je ongeveer twee á drie maanden. Als kindergezondheidscoach werk je dan al ongeveer een half jaar met het kind/jongere. Afhankelijk van hoe de begeleiding verloopt en of er positieve veranderingen optreden in de leefstijl van het kind/jongere, breid je na een half jaar de tijd tussen de consulten verder uit. Als kindergezondheidscoach ben je vrij om het

tempo zelf te bepalen, mits je de richtlijn van 14 consulten in 2 jaar aanhoudt. Deze richtlijn bevat 12 standaard consulten en twee herhalingsconsulten. Bij het kind/jongere waar (multi) -problematiek optreedt of aanwezig blijkt te zijn wordt met de verwijzer en de ketenzorg individueel gekeken naar het verloop en de intensiteit van de begeleiding.

De begeleiding van het kind/jongere is Op Maat gemaakt. Dit betekent dat er per kind/jongere en zijn/haar gezin gekeken wordt wat hij/zij individueel nodig heeft in zijn/haar begeleiding. Je past je consult aan naar de leeftijd van het kind/jongere. Zo kan het zijn dat je bij jongere kinderen (vijf jaar of jonger) voornamelijk met de ouder/verzorger spreekt en werkt en bij een ouder kind/jongere voornamelijk met hen zelf werkt. In beide gevallen blijf je rekening houden met de thuissituatie en doe je hier navraag naar. Je blijft contact houden met het hele gezin.

Je vraagt de adresgegevens/telefoonnummer/e mailadres van het kind/jongere en maakt een afspraak voor een intakegesprek. Je stuurt een bevestiging van de afspraak met datum, tijd en locatie. Op deze bevestiging staan ook de contactgegevens van jou als coach zodat het kind/jongere of ouder/verzorger weet hoe ze een afspraak kunnen verzetten of afzeggen. Geef deze gegevens tijdens het gesprek door.

Je vertelt de ouder/verzorger dat je het intakeformulier, deel 1 (pag. , naar ze opstuurt en vraagt of ze dit formulier ingevuld mee kunnen nemen naar de eerste afspraak. Je checkt bij de ouder/verzorger of dit mogelijk is, denkend hierbij aan taalbarrière en analfabetisme.

Je informeert de ouder/verzorger dat het programma GO! meedoet aan een onderzoek naar de Kwaliteit van leven, genaamd Pedsql. Dit onderzoek meet de kwaliteit van leven van het kind, voor, tijdens en na de begeleiding van het programma GO!
Je legt uit wat dit onderzoek behelst (zie bijlage 18) en vraagt of de ouder/verzorger daaraan mee wil doen. De vragenlijsten zijn te verkrijgen via de senior kindergezondheidscoach. Bij deelname stuur je de Pedsql vragenlijst op (samen met Deel 1 van de intakelijst). Je vraagt de ouder/verzorger om deze ingevuld mee te nemen naar de eerste afspraak. Je voorziet de Pedsql lijst van het inclusie nummer van het nieuwe kind/jongere, je geeft aan uit welke wijk het kind/jongere komt en voorziet de Pedsql lijst met de code T=0.
Blijf rekening houden met laaggeletterdheid en taalbarrières. Dit kan er namelijk voor zorgen dat mensen de afspraak niet goed zullen begrijpen en niet goed weten wat deze inhoudt. Vraag daarom altijd terug of de afspraak duidelijk is. Mocht je tijdens het telefonische gesprek constateren dat er een taalbarrière is, vraag de ouder/verzorger of er iemand uit de naaste omgeving mee kan komen naar het intakegesprek om als tolk op te treden. Tijdens het programma is deelname, steun, van de ouder/verzorger van groot belang. Vraag de ouder/verzorger daarom zoveel mogelijk aanwezig te zijn bij de consulten. Belangrijk is bij het kind/jongere die alleen komt dat je belangrijke informatie deelt met de ouder/verzorger. Verder geef je nogmaals aan dat deelname aan het programma in principe 2 jaar is. Dit omdat je door het doorlopen van het programma werkt aan een gedragsverandering op het gebied van een gezonde leefstijl.

Het intake-gesprek & Uitleg Bloedsuikerspiegel

Het intake-gesprek
Doel
Het kennismaken met het kind/jongere en de ouder/verzorger.
Het afnemen van de anamnese door middel van vragenlijst deel 2.
Het kind/jongere start met de eerste veranderingen in zijn/haar leefstijl om te komen tot een gezondere leefstijl bij het kind/jongere en de ouder/verzorger.

Werkwijze
Het eerste consult duurt één tot anderhalf uur. Je maakt kennis en stelt het kind/jongere en de ouder/verzorger op zijn/haar gemak.
De intake bestaat uit de volgende punten, die stapsgewijs doorlopen worden:

- Het in ontvangst nemen van het ingevulde deel 1 van het intake formulier en de ingevulde vragenlijst naar het onderzoek Kwaliteit van leven.
- Afname van deel 2 van het intake formulier. Belangrijk is het registreren van toestemming door ouders/verzorgers voor het gebruik van gegevens en delen van informatie in de ketenzorg.
- Registreren van het wel of niet meedoen aan wetenschappelijk onderzoek bijvoorbeeld, Kwaliteit van leven.
- Introductie werkboek
    - Uitleg afspraken annuleren dan wel verzetten. (zie pag. 24)
    - Het werkboek laten zien aan het kind/jongere.
- Analyse van de voedingsinname van 1 dag door de week (schooldag) en voedingsinname in het weekend. Deze voedingsanalyse wordt gedaan tijdens de intake en direct besproken. (zie pag. 29 en 30)
    - Uitleg en samen invullen van het eetschema
      Eetschema 1 (oudere kind/jongere) of eetschema 2 (jonge kind/taalbarrière/ analfabeet). (zie pag.32)
- Uitleg/invullen weeg- en meet kaart (zie pag. 26)
- Uitleg bloedsuikerspiegel (zie pag. 36)
- Benoemen en eventueel noteren in het werkboek van de gemaakte afspraken voor de komende periode en het maken van een vervolgafspraak.

Je verwerkt na het consult, alle verkregen gegevens en informatie in het dossier van het desbetreffende kind.

Intake-formulier deel 1

Doel

Algemene gegevens verkrijgen van het te includeren kind/jongere.

Werkwijze

De coach heeft telefonisch doorgegeven tijdens het maken van de eerste afspraak dat het intake formulier wordt toegestuurd en dat de ouder/verzorger deze ingevuld meeneemt naar het intakegesprek. De kindergezondheidscoach kijkt of deel 1 volledig is ingevuld en vult indien nodig gegevens aan. Deze gegevens worden bewaard in het dossier van het desbetreffende kind.

Intake-formulier (deel 1)

Graag ingevuld meenemen naar de eerste afspraak

Algemene Gegevens

Naam		
Leeftijd		
Geboortedatum		
Adres		
Telefoon		
Email		
Verzekering		
Polisnummer		
BSN		
School		
Groep/ klas		
Hoogst genoten opleiding ouders	Vader<br>◻ Geen afgemaakt<br>◻ Lagere school<br>◻ VMBO<br>◻ Havo<br>◻ VWO<br>◻ MBO<br>◻ HBO<br>◻ WO	Moeder<br>◻ Geen afgemaakt<br>◻ Lagere school<br>◻ VMBO<br>◻ Havo<br>◻ VWO<br>◻ MBO<br>◻ HBO<br>◻ WO

Medische Gegevens

Wie is je huisarts?	
Wie is eventueel je kinderarts?	

Ga je naar een specialist? (arts in ziekenhuis)	
Welke medicijnen gebruik je?	
Gebruik je vitaminepillen?	
Ben je ergens allergisch voor?	

Nb voor het jongere kind is de aanspreekvorm de *verzorger/ouder*

Intake-formulier deel 2
Doel
Medische en leefstijl gegevens verkrijgen van het kind/jongere en zijn/haar omgeving.
Inzicht verkrijgen in het bestaande voeding/eetschema van het kind/jongere en zijn/haar gezin.

Naast de verkregen informatie analyseer je de mogelijke belemmerende factoren en de motivatie van het kind en de ouder/verzorger. Belemmerende factoren kunnen onder andere zijn: financiële- en relationele problemen, eetproblemen, depressie en angststoornissen.

Werkwijze
Het kind/jongere en de ouder/verzorger zijn samen aanwezig bij het intakegesprek.
Je neemt een volledige anamnese en vragenlijst af over de leefstijl van het kind/jongere en zijn/haar omgeving.
Bij deelname aan het onderzoek Kwaliteit van leven neem je de ingevulde Pedsql vragenlijst in en stuur je die door. Het adres kun je navragen bij de senior kindergezondheidscoach.

Intake formulier deel 2

In te vullen door coach met kind/jongere en ouder/verzorger tijdens intake

Nb. lijst wordt afgenomen bij ouder/verzorger bij kinderen onder de zeven jaar

Locatie: …………………………………………………………

Kindergezondheidscoach: …………………………….

Datum intake: ……………………………………………….

Verwijzer: (aankruisen)

- ☐ Kinderarts, namelijk ……………………………
- ☐ Huisarts, namelijk ………………………………..
- ☐ Jeugdarts, namelijk ……………………………..
- ☐ Overig, namelijk …………………………………..

Wetenschappelijk onderzoek (Aliko): Ja……………..Nee………………

Studienummer: ……………………………………………….

Toestemming gegevens delen in ketenzorg: ja / nee

(LET OP: eerst algemene gegevens doornemen met kind/jongere en ouder/verzorger.)

Medische Gegevens

Geboortegegevens:<br>☐ Zwangerschapsduur<br>☐ Hoeveelste kind<br>☐ Geboortegewicht<br>☐ Geboortelengte<br>☐ Borstvoeding: ja/nee<br>☐ Duur flesvoeding<br>☐ Duur borstvoeding	
Ziektegeschiedenis kind:<br>Heeft het kind een syndroom of een lichamelijke beperking?<br><br>Heeft het kind een ATE <2j gehad?	

Zo ja, daardoor voedingsproblematiek gekregen?	
Heeft het kind een allergie?<br>Zo ja; welke?	
Eerdere interventies:<br>-Diëtiste/ leefstijlbegeleiding<br><br>-Fysiotherapie<br>(motorische problematiek)<br><br>-Beweegprogramma's<br><br>-Psycholoog/ coaching<br>Ja/nee en zo ja, hoe lang<br><br>-Zo ja, heeft deze interventie je geholpen?<br><br>Zijn er gedrag- en/of leerproblemen op school?	
Is er contact met het wijkteam? (wijkcoach, jeugdconsulent) (zie ook werkwijze)	
Extra ondersteuning?<br>□ Kind<br>□ Gezin<br>Zo ja, welke?	
Heb jij taken in het gezin? (inzicht in systeem gezin).<br>Zo ja, welke?	

Familiegegevens

Hoe is de gezinssamenstelling en zijn er huisdieren?	
Werkzaamheden ouders (werkt u?)	
Gaan kinderen naar de opvang?	

Familieanamnese:	Vader	Moeder
Oorspronkelijke afkomst van ouders<br><br>Komt er overgewicht voor in de families?<br>Zo ja, bij wie?<br><br>Komen er hart- en vaatziekten <60j voor in de familie?<br>Zo ja, bij wie?<br><br>Komt er hoog cholesterolgehalte voor in de familie?<br>Zo ja, bij wie?<br>Komt er hoge bloeddruk voor in de familie?<br>Zo ja, bij wie?<br><br>Komt er diabetes type 2 voor in de familie?<br>Zo ja, bij wie?<br><br>Heeft moeder zwangerschaps-DM ontwikkeld?		

Wat is de lengte en het gewicht van ouders?		

Lichamelijke Factoren

Had je de afgelopen maand:	JA	NEE	SOMS	NVT
Hoofdpijn				
Buikpijn				
Rugpijn				
Gewrichtsklachten				
Misselijk/Braken				
Concentratieproblemen				
Obstipatie (houd rekening met culturele achtergrond)				
Menstruatie (houd rekening met culturele achtergrond)				
Ongewenste haargroei op armen, benen en gezicht (houd rekening met culturele achtergrond)				
Huidverschijnselen (AN, striae)				
Snurken				
Overig:				

Sport en Activiteiten

Wat zijn je hobby's?	
Zit jij bij een vereniging of club? Zo ja, welke?	

Hoe vind jij het op je vereniging/club?	
Hoeveel beweeg je per dag?<br><br>Hoe vaak sport je per week?<br><br>Hoe lang sport je?	
Hoe vaak en hoe lang<br>speel jij buiten? (Jongere kind)<br><br>Hoe vind jij het bij jou in de buurt?	
Hoeveel tijd besteed je aan screen-time (tv, ipad etc.) per dag? Zijn er afspraken tussen kind/jongere en ouder/verzorger over beeldschermgebruik?	

Voedingsgewoonten van het kind/jongere

Zijn er een bepaalde voedingsgewoonten of een voedingsstructuren in het gezin? Bijvoorbeeld elke week is er een:<br>- avond broodmaaltijd<br>-patat dag<br>-vlees vrije-dag<br><br>Hoeveel frisdrank drinkt het kind/jongere per dag of week?<br><br>Moet het kind/jongere wel eens alleen eten?<br><br>Sport het kind/jongere rond etenstijd en wat eet of drinkt hij/zij?<br><br>Krijgt het kind/jongere een eet beloning na het sporten?	

Zijn er voedingsgewoonten ten aanzien van een geloofsovertuiging of bepaalde levensstijl? (varkensvlees vrij, vegetarisch, veganistisch)	

Slaap en Ontspanning

Hoe laat ga jij naar bed?	
Hoeveel slaap je gemiddeld per nacht?	
Kun jij makkelijk in slaap komen?	
Slaap je goed door?	

School/ Zelfbeeld

Hoe vind je het op school?	
Heb je vriendjes/ vriendinnetjes?	
Word jij weleens gepest?	

Algemene vragen

Wat vind je zelf van jouw gewicht?	
Heb je last van je gewicht?	Ja / nee
Ken je de oorzaken van overgewicht?	Ja / nee
Denk je dat je verzorger/ouder je kan en wil ondersteunen in het veranderen van je leefstijl?	

Verwachtingen

Wat is je motivatie om te starten met GO!?<br><br>Wat wil je bereiken met GO!?<br>-Wat denk je daarvoor nodig te hebben?<br><br>Zijn er op dit moment barrières waardoor je niet een gezonde leefstijl kan hebben?	
Wat verwacht je van de kindergezondheidscoach?	

Bijzonderheden/ aantekeningen:

………

………

………

………

Nb voor het jongere kind is de aanspreekvorm de *verzorger/ouder*

Introductie werkboek tijdens het intakegesprek
Na het afnemen van het intake formulier deel twee, introduceer je het werkboek voor het kind/jongere. Het werkboek bestaat uit 12 standaard consulten waarop het programma GO! is gebaseerd.

Afspraken verzetten en maken
Doel
Duidelijke informatie geven over het maken en verzetten van afspraken tussen de kindergezondheidscoach, kind/jongere en ouder/verzorger.

Werkwijze
Schrijf de gegevens (naam, telefoon en mailadres) van de coach op het formulier en leg de procedure voor het maken, verzetten en afzeggen van afspraken uit.

Naam Coach:

E-mail Coach:

Tel. Coach:

W www.go-nl.nl

Algemene afspraken

AFSPRAAK VERZETTEN

Mocht je een afspraak willen verzetten dan moet dit 24 uur van tevoren (probeer liever nog eerder) gebeuren.
Het is mogelijk een berichtje naar je coach te sturen of te mailen.
Vermeld daarin duidelijk je naam, je telefoonnummer, de datum en tijd van de afspraak die je wilt verzetten.

AFSPRAAK VERGETEN

Als je een afspraak vergeten bent, bel je coach dan zo snel mogelijk voor het maken van een nieuwe afspraak.

Als een afspraak vergeten wordt, heeft je coach onnodig tijd voor jou vrijgemaakt. Tijd die je coach ook aan een ander had kunnen geven.

Intake: Weeg- en meetkaart

Doel
Het verkrijgen van een overzicht van de weeggegevens en de metingen (bloeddruk, lengte, taille) van het kind/jongere per consult.

Werkwijze
Het kind/jongere wordt elk consult gewogen. De lengte wordt iedere maand gemeten. De weegschaal berekent automatisch de body Mass index (BMI) wanneer je de lengte invoert. De taille wordt ieder consult gemeten. Je noteert de gegevens op de meet/weegkaart in het werkboek van het kind/jongere. Je voert de gegevens in het digitale dossier in van het kind/jongere. Je meet de bloeddruk (RR) bij het kind/jongere. Wanneer de RR afwijkend is (zie bijlage 1) geef je dit door aan de huisarts of kinderarts. Je meet de RR bij het volgende consult weer.
Bij kopje opmerkingen, op de meet-weegkaart, worden bijzonderheden genoteerd op de van het kind/jongere. Wanneer er geen bijzonderheden zijn, vink je dit af met GB= Geen Bijzonderheden. Hoe je lengte, tailleomtrek en bloeddruk meet staat beschreven in de algemene introductie van het protocollenboek en wordt ook praktisch uitgelegd tijdens de opleiding.

Alle andere onderdelen die op de weeg-meetkaart staan zijn voor jongeren die uitgegroeid zijn. Indien je een consult hebt met een uitgegroeid kind/jongere dan vul je deze verkregen gegevens vanuit de weegschaal in op de weeg/meet kaart van de jongere.

-----Weeg en Meet Kaart ----

Naam: .... **Geslacht m/v** ....

St.nr: .... **Aliko ja/nee:** ....

	1	2	3	4	5	6	7	8	9	10
Datum										
Tijstip										
Gewicht										
Vet%										
Spiermassa										
Botmassa										
BMI										
Metab. Leeft.										
Vocht%										
Visceraal vet										
Taille										
Lengte										
Leeftijd										
BMI (streef)										
Bloeddruk										
Peds QL	T=0		T=3		T=6		T=12		T=24	

OPMERKINGEN

Intake: Eetschema

Doel
Inzicht verkrijgen in het bestaande voeding/eetschema van het kind/jongere en zijn/haar gezin.
Het bestaande voedings-/eetschema van het kind/jongere aanpassen naar de richtlijnen van het voedingscentrum *binnen* de mogelijkheden van het gezin.
Middels het voedings-/eetschema van het kind/jongere wil je dat het kind/jongere drie hoofdmaaltijden per dag nuttigt met tussendoor maximaal drie momenten van fruit en of groenten. Op een later moment zal tussendoor ook bewust gekozen kunnen worden voor een “snack/snoep”-moment.

Als coach stimuleer je dat de hoofdmaaltijden bestaan uit gevarieerde producten uit de schijf van vijf (zie schema in bijlage 14). Hierdoor probeer je zo optimaal mogelijk aan een volwaardige inname van voedingstoffen te komen. Daarnaast geeft het ritme van drie hoofdmaaltijden en maximaal drie gezonde tussendoortjes regelmaat aan het kind/jongere in het eetpatroon, waardoor de kans op het hongergevoel terug wordt gebracht.

Werkwijze
Je vult met het kind/jongere en ouder/verzorger een dagmenu in van een gemiddelde (school)dag. Je vraagt aan het kind/jongere en de ouder/verzorger of het dagmenu overeenkomt met een dag uit het weekend. Eventuele grote verschillen noteer je. Het invullen van het eet/drink dagboek helpt het kind/jongere, de ouder/verzorger en de kindergezondheidscoach om een beter inzicht te krijgen in de op dat moment bestaande eetgewoontes. Het is namelijk alleen mogelijk om iets te veranderen in je eetgedrag als je bewust bent van je eetpatronen. Om deze reden kan het wenselijk zijn om op een later moment gedurende de begeleiding een dagboekje voor langere periode bij te laten houden. (zie consult 5).

Er zijn twee soorten eetschema’s volgens de laatste richtlijnen van het voedingscentrum. Eetschema I is geschreven en bedoelt voor het oudere kind of jongere. Eetschema II is visueel en is bedoeld voor het jongere kind, of wanneer er een taalbarrière of analfabetisme speelt. Indien wenselijk kunnen de eetschema’s ook samen mee gegeven worden.
Het eetschema leg je uit aan het kind/jongere en zijn/haar ouder/verzorger en je vergelijkt het eetschema met het opgeschreven dagmenu van het kind/jongere. Wees er alert op dat aanpassingen in het eetschema niet in een keer allemaal veranderd kunnen worden. Je bepaalt samen met het kind/jongere en zijn/haar ouder/verzorger wat er als ontbijt/lunch/diner gegeten dan wel veranderd kan worden, denkend aan de schijf van vijf en wat past in de thuissituatie (cultureel, financieel en werkzaamheden ouders). Je adviseert ook om te stoppen met het drinken van suikerhoudende dranken. Stimuleer het kind/jongere hiervoor in de plaats water te gaan drinken. Eventueel kun je consult 3 (ontbijt &water) erbij pakken wanneer extra uitleg hierover op dit moment al wenselijk is. Je motiveert het kind/jongere zich aan dit eetschema te houden.

In week één krijgt het kind/jongere alleen wat er op het eetschema staat (aangepast op het kind/jongere en de thuissituatie).
Er wordt tussen de hoofdmaaltijden alleen groente en/of fruit gegeten, dit is het tussendoortje. Als tussendoortje wordt er dus geen snoep/snacks (koek, snoep, chips, ontbijtkoek, crackers, boterhammen) gegeten. Het kind/jongere wordt uitgedaagd om dit één week vol te houden. Probeer het kind/jongere en de ouder/verzorger hier positief te motiveren en te stimuleren.
Er wordt overlegd met de kindergezondheidscoach en het kind/jongere wanneer er blijkt dat er een bijzondere gebeurtenis(feest) plaatsvindt in deze week. Het eetschema zal dan worden aangepast.
Het toetje na het diner mag eventueel wel geruild worden met het tussendoortje in de middag.
Als de uitleg over de werking van de bloedsuikerspiegel geweest is, kun je nog beter uitleggen waarom er één week tussendoor alleen groente en fruit gegeten wordt.
Na één week krijgen de kinderen/jongeren één groot snack/snoepmoment per week. Het advies is dit in het weekend in te plannen. Optimaal is dat dit een gezamenlijk moment wordt in het gezin. Het is belangrijk dat het kind/jongere iets kiest wat hij/zij echt lekker vindt en naar uit kan kijken.

Na de tweede week krijgt het kind/jongere er een klein snack/snoepmoment bij. Ons advies is dit op de woensdag te plannen. Deze week heeft het kind/jongere dus één groot en één klein snack/snoepmoment.

Voorbeelden groot snack/snoepmoment:

> *stukje appeltaart, cup cake, chips, stukje chocola (bijv. Mars), ijsje. In plaats van een warme maaltijd mag er ook een keer friet gegeten worden (dit geldt als snackmoment). Wil het kind/jongere taart of een cup cake: stimuleer ze om deze zelf te bakken, want het idee is dat je het gezamenlijk met het gezin doet en je er een gezamenlijk moment van maakt.*

Voorbeelden klein snack/snoepmoment:

> *twee blokjes chocola, koekje, drie winegums/drop/pepermunt, stukje worst of kaas, etc.*

Na de derde week wordt door de coach bekeken of er uitbreiding met kleine snack/snoepmomenten mogelijk is bij het kind/jongere. Dit hangt af van hoe de ontwikkeling van de nieuwe leefstijl bij het kind/jongere en zijn/haar gezin verloopt. Breid de snoep/snackmomenten niet te snel uit tot het maximaal aantal per week. Dit voorkomt eventuele terugval naar het oude snoep patroon. De uitbreiding mag maximaal naar één groot snack/snoepmoment en drie kleine snack/snoepmomenten per week (rekening houdend met traktaties op school en verjaardagen/feesten).

Voedingsanamnese
Het eetdagboek helpt jou om een beter inzicht te geven in jouw eetgewoonten.

Eetdagboek door de week

**Eetdagboek van ……………….**	**Datum:...................**
**Ontbijt:**	
**Tussendoor:**	
**Lunch:**	
**Tussendoor:**	
**Avondeten:**	
**Tussendoor:**	

Eetdagboek in het weekend

Eetdagboek van ……………….. Datum:....................
**Ontbijt:**
**Tussendoor:**
**Lunch:**
**Tussendoor:**
**Avondeten:**
**Tussendoor:**

GO! GEZOND ONDERWEG

Eetschema I

**Eetschema van ………………..** **Datum:………………..**
**Ontbijt:**<br>……. volkoren boterhammen met dun laagje (room)boter/halvarine.<br>-Of halfvolle yoghurt (max.250 ml.) met bijvoorbeeld lijnzaad, muesli/havermout, noten, fruit.<br>-Of halfvolle melk met brinta of havermout.<br>Beleg: Alle soorten beleg mogen. Smeer of beleg het brood niet dubbeldik. Varieer zoveel mogelijk in beleg!<br>Drinken: 1 glas zuivel, thee (zonder suiker) of water
**Tussendoor:**<br>Fruit en/of groente of een klein handje ongezouten noten.<br>Drinken: water of (groene) thee zonder suiker, ranja zonder suiker.
**Lunch:**<br>……. Volkoren boterhammen met dun laagje (room)boter/halvarine<br>Beleg: Alle soorten beleg mogen. Smeer of beleg het brood niet dubbeldik. Varieer zoveel mogelijk in beleg!<br>Drinken: water of (groene) thee zonder suiker
**Tussendoor:**<br>Fruit en/of groente of een klein handje ongezouten noten.<br>Drinken: water of (groene) thee zonder suiker, ranja zonder suiker.
**Avondeten:**<br>Groente minimaal één opscheplepel. Eén portie: vlees of vis of kip of ei of een vleesvervanger.<br>Eén opscheplepel aardappel of bataat of zilvervliesrijst of couscous of volkorenpasta.<br>Eén uur na het eten een toetje: b.v. yoghurt, kwark of magere vla (zonder suiker).<br>Advies:<br>Eet minimaal 1 keer per week peulvruchten (vlees is niet nodig die dag) en vette vis. Voeg geen extra zout toe.<br>**Drink water 1,5 liter per dag!**

Eetschema II

Ontbijt:

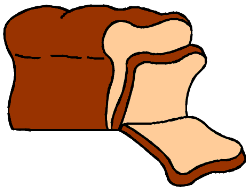 of 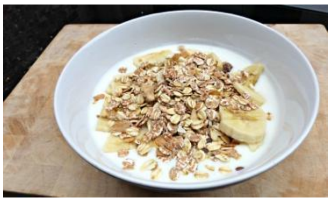 met 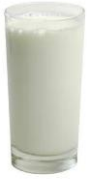 of 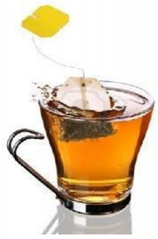

Tussendoor:

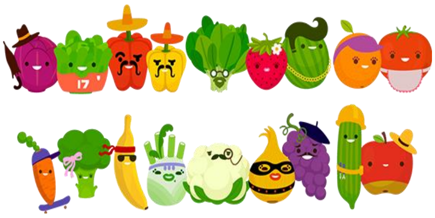 en 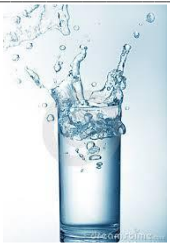 en 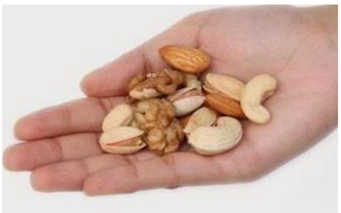

Lunch:

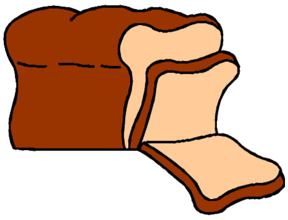 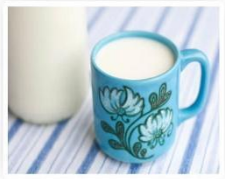 of 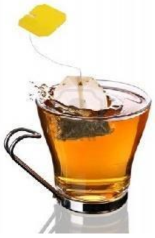

Tussendoor:

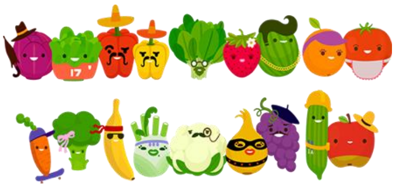 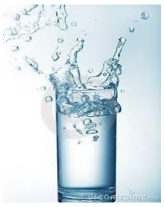

Diner:

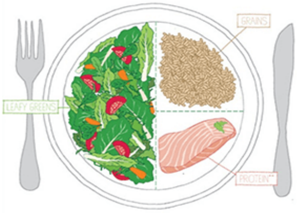

1 keer per week: 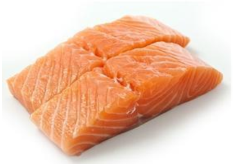 of 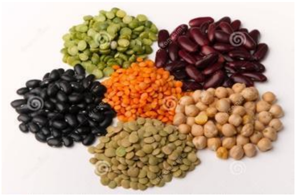

Tussendoor:

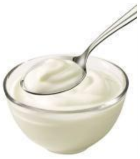 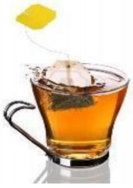 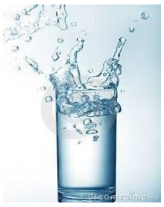 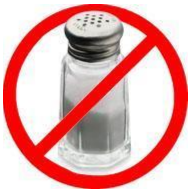

Intake: Uitleg Bloedsuikerspiegel

Doel

Het kind/jongere en ouder/verzorger krijgt inzicht in zijn/haar suikerverslaving.
Het kind/jongere en zijn/haar ouder/verzorger weet wat een bloedsuikerspiegel is.
Het kind/jongere en zijn/haar ouder/verzorger kent de verschillen tussen snelle en langzame suikers.

Werkwijze

Je neemt het afgenomen voeding-vocht dagboek van het kind/jongere erbij. Samen met het kind/jongere en de ouder/verzorger bekijk je wat er over die dag gegeten en gedronken is. Je analyseert volgens de richtlijnen van het voedingscentrum de inname van de voeding en vocht. Je vertelt aan het kind/jongere en de ouder/verzorger wat je geanalyseerd hebt. Hoeveel maaltijden werden er over de hele dag gegeten? Wat is de hoeveelheid limonade en snacks die gegeten zijn die dag? Was er voldoende inname van fruit, zuivel en goede vetten? Vaak zie je dat er over de hele dag heen (te) veel snelle suikers gegeten en gedronken zijn. Deze conclusie is een aanleiding om uit te leggen wat een suikerverslaving is. Je legt uit dat het woord suikers hetzelfde betekent als het woord koolhydraten.

Ons lichaam heeft net als een auto, brandstof nodig om te kunnen bewegen. Voor de mens zijn er drie soorten brandstof, namelijk koolhydraten(suiker), vetten en eiwitten (in hoofdstuk 9 beschrijven we dit uitgebreider).
Je vertelt dat in ons lichaam de brandstof suiker het makkelijkst om te zetten is in energie, deze energie heet glucose. Er zijn twee soorten suikers, namelijk snelle suikers (eenvoudige, geraffineerde) en langzame suikers (meervoudige), bijvoorbeeld zetmeel.
Je legt het verschil tussen snelle en langzame suikers in dit consult uit.

Snelle suikers worden makkelijk in je lichaam omgezet tot glucose (suiker) en daarna in je bloed opgenomen (zie grafiek 2). Bij inname van deze snelle suikers stijgt je bloedsuikerspiegel snel. Je krijgt hierdoor snel energie. Om ervoor te zorgen dat de bloedsuikerspiegel niet te hoog wordt en glucose de cel ingaat als energiebron maakt het lichaam als reactie insuline aan en zakt je bloedsuikerspiegel weer. De energie is hierdoor weer snel weg. Dit stijgen en dalen kan al binnen een half uur gebeuren. Die daling zorgt ervoor dat je weer zin hebt om iets te gaan eten of drinken. Je hebt op dat moment dan meestal niet zin in iets gezonds maar in een snelle snack.
Dit is geen hongergevoel maar trek! Als je nu weer iets met snelle suikers eet of drinkt dan blijft je bloedsuikerspiegel heen en weer schommelen (zie grafiek 1). Door deze schommeling werken snelle suikers verslavend.

Je vraagt aan het kind/jongere en ouder/verzorger of zij de volgende symptomen herkennen?
-Het kind/jongere is het ene moment druk, opgejaagd en hyper (bloedsuikerspiegel is hoog).
-Het kind/jongere is het andere moment vermoeid en minder geconcentreerd, gaat gapen en heeft hoofdpijn (bloedsuikerspiegel is laag).
Per kind/jongere kan dit verschillend worden ervaren.

Langzame suikers zitten moeilijker in elkaar. Het duurt daarom langer voor het lichaam om ze uit elkaar te halen en om te zetten in glucose, energie. De bloedsuikerspiegel stijgt en daalt hierdoor langzamer (zie grafiek 3). Bij opname in je lichaam hiervan krijg je langer een verzadigd/vol gevoel. Hierdoor heb je minder snel de behoefte aan een snelle snack of suikerhoudende drank. Langzame suikers bevatten meer vezels vitaminen en mineralen dan snelle suikers. Ze zijn daarom gezonder dan snelle suikers.

Ouders zullen je misschien vragen naar het gebruik van zoetstoffen. Er zijn veel onderzoeken gedaan naar de werking van zoetstoffen en eventuele gevolgen door veelvuldig of langdurig gebruik/inname. Veel onderzoeken spreken elkaar tegen. Het is daarom verstandig het kind/jongere en de ouder/verzorger te adviseren niet over de grens van maximaal 2 a 3 producten met zoetstoffen per dag, heen te gaan.
Je legt hierna uit dat zoetstoffen je hersenen kunnen laten denken dat je snelle suikers aan het eten of drinken bent. Gebruik de uitleg van de lichamelijke reacties door inname van snelle suikers van hierboven. De zoetstoffen zelf zorgen er niet voor dat je zwaarder wordt maar wel het eten van de snack erna. Het advies van de gezondheidsraad is maximaal 2 tot 3 producten met zoetstoffen per dag. (Check hierover regelmatig wat het meest recente advies is.)

Uitleg grafiek 1 Suikerverslaving
Eerder in het consult heb je de suikerverslaving uitgelegd. We gaan nu uitleggen hoe we deze suikerverslaving kunnen gaan doorbreken. Je neemt nu grafiek 1 erbij en geeft hierover nogmaals de uitleg. (Hierdoor kom je ook te weten of het kind/jongere en de ouder/verzorger je informatie en uitleg begrepen hebben).
De bovenkant van de piekjes in de grafiek kun je in een grote lijn omcirkelen. Daarbij kun je aangeven dat binnen deze cirkel een teveel aan suikers aanwezig is. Deze suikers zullen worden omgezet in vet. Bij kinderen gaat dit vet als eerste rondom de buik zitten. We noemen dit heel lief het “suikerbuikje”. Maar eigenlijk is dat “suikerbuikje” niet zo lief!

Je gaat de suikerverslaving met het kind/jongere beeldend stoppen door in grafiek 1 een kruis te zetten. Dit snelle schommelende suiker beeld wil je namelijk doorbreken bij het kind/jongere.
Om deze suikerverslaving te stoppen gaat het kind/jongere een uitdaging van één week aan.
De uitdaging is dat het kind/jongere één week tussendoor alleen groente en/of fruit eet.
Andere tussendoortjes mogen nu even niet, bijvoorbeeld ook geen crackers, ontbijtkoek of boterhammen tussendoor.
Qua drinken drinkt het kind/jongere alleen, water, thee zonder suiker, ranja zonder suiker of light frisdrank (maximaal 2 a 3 zoetstof houdende producten per dag).

Uitleg grafiek 2 Snelle suikers versus langzame suikers
In de grafiek geeft de rode lijn de stijging van snelle suikers in een witte boterham aan. De groene lijn geeft de stijging van langzame suikers in een volkoren boterham aan.

Uitleg grafiek 3 Gewenste bloedsuikerspiegel door de dag heen
In deze grafiek wordt getoond wat de gewenste bloedsuikerspiegel bij een mens over de hele dag heen is. Tussen de grote bogen zie je kleine bogen. Deze bogen verbeelden de suikerspiegels van de tussendoortjes op die dag.

Hoe werkt de bloedsuikerspiegel?

Suikerverslaving (grafiek 1)

Hoogte suiker in bloed

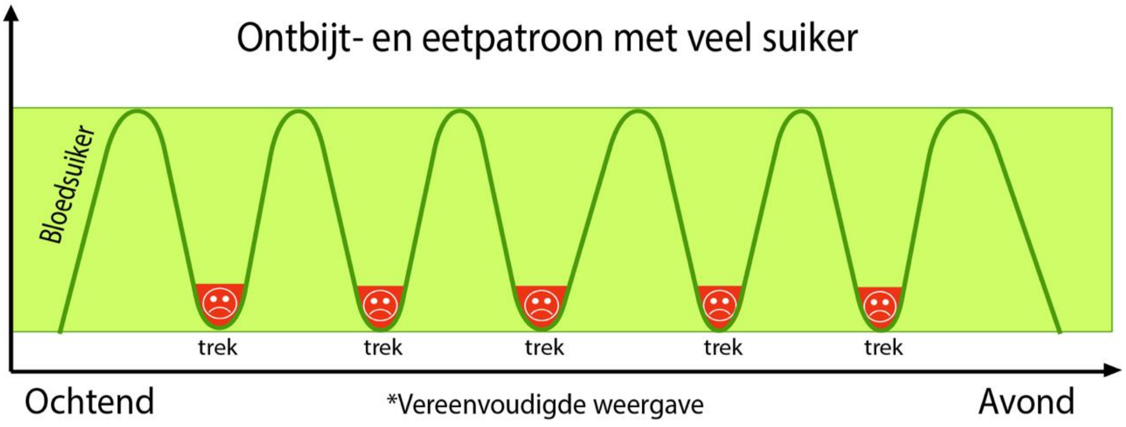

Snelle suikers versus langzame suikers (grafiek 2)

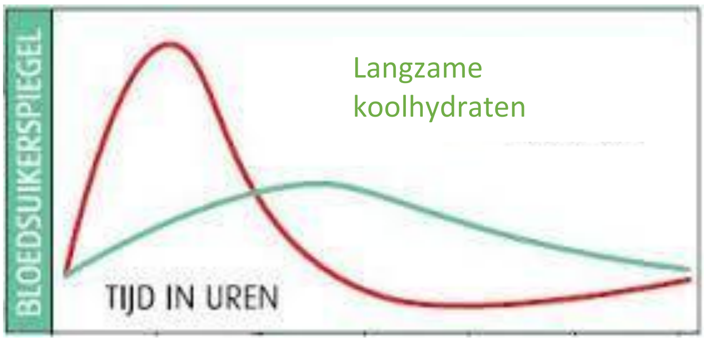

Volkoren boterham (langzame koolhydraten) of witte boterham (snelle koolhydraten)

Gewenste bloedsuikerspiegel over de dag heen (Grafiek 3)

Hoogte suiker in bloed

Op

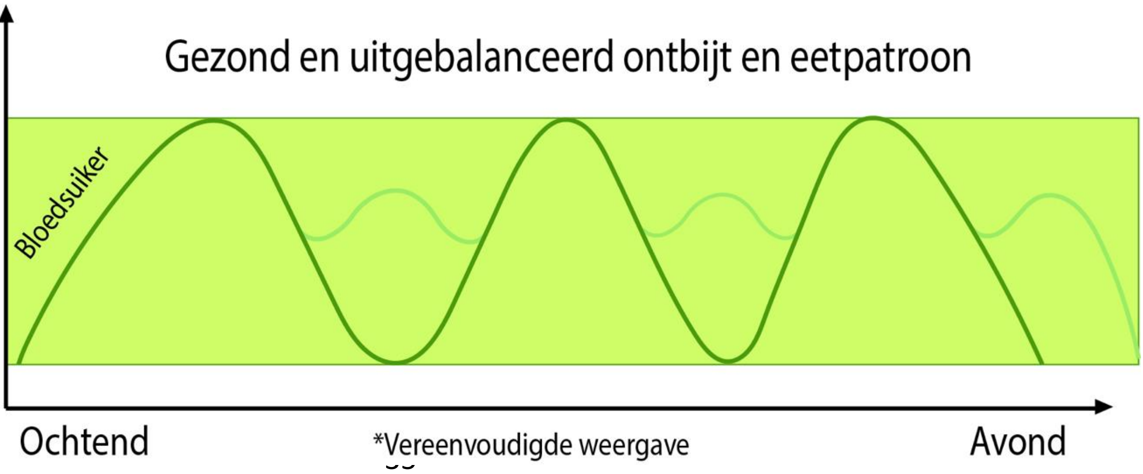

Consult intake:

Suikeropdracht
Doel
Het kind/jongere en ouder/verzorger leert hoe het kan uitrekenen hoeveel suikerklontjes er in een product zitten.
Het kind/jongere en ouder/verzorger is zich bewust van de hoeveelheden suiker in producten en kan een verantwoorde keuze hierin maken.

Werkwijze
Je vertelt het kind/jongere en de ouder/verzorger dat het thuis twee producten moet gaan uitkiezen. Bijvoorbeeld een pakje drinken of de pot chocopasta voor op brood. Je legt uit dat achterop de verpakking van het product staat aangegeven hoeveel vet, eiwit en koolhydraten, per 100 gram of 100 ml, in het product zitten. Voor het berekenen van de suikerklonten kijk je bij de koolhydraten. Onder de koolhydraten staat: *waarvan suikers*. Je kunt ervan uitgaan dat hiermee de snelle suikers worden bedoeld.
Je legt de rekensom voor het berekenen van de hoeveelheid suikerklonten als volgt uit:
Je vertelt dat één suikerklontje in totaal 5 gram is. Het kind/jongere leest op de verpakking hoeveel gram erachter *waarvan suikers* staat. Dit is bijvoorbeeld 20 gram. Het kind/jongere deelt nu 20 gram door 5 gram, de uitkomst is 4. Dit betekent dat er 4 suikerklontjes per 100 gram of 100 ml. in het product zit. Nu moet het kind/jongere en de ouder/verzorger de hoeveelheid suikerklontjes omrekenen naar de hoeveelheid die totaal in het product zit. Bijvoorbeeld een pakje drinken bevat in totaal 200 ml. Je rekent dan de totale hoeveelheid suikers uit door 4 (suikerklontjes) x 2 (200ml ipv 100ml) te doen. De uitkomst is hiervan 8; dat 8 suikerklontjes betekent. Het pakje drinken bevat 8 suikerklontjes.

Op deze manier kun je als kind/jongere en ouder/verzorger altijd uitrekenen of een product veel of weinig suikers bevat. Hierdoor maak je de aanwezige hoeveelheid suikerklonten in de voeding of vocht beeldend voor het kind/jongere. Dit is de eerste stap van bewustwording van de suikerinname van het kind/jongere.

Als extra opdracht kun je voorstellen aan het kind/jongere en de ouder/verzorger om een product uit te kiezen waarvan zij denken dat er geen suiker in zit. Vaak blijkt er dan toch suiker in te zitten. Bijvoorbeeld kipfilet voor op brood. Deze uitkomst kan helpen in de bewustwording van de aanwezigheid en hoeveelheid suikers in voeding en vocht.
Je kunt bij dit consult gebruik maken van de suikerklontjes lijst, zie bijlage 8 en 9.

Thuisopdracht 1 en 2:
Het kind/jongere schrijft met de ouder/verzorger de rekensom op van de twee uitgekozen producten.
Blokje 1: kies één product en bereken het aantal suikerklontjes dat er in het product zitten.
Blokje 2: kies één product en bereken het aantal suikerklontjes dat er in het product zitten.

Suiker opdracht

Thuisopdracht 1
Reken uit hoeveel suikerklontjes er zitten in …………………………………..

Thuisopdracht 2
Reken uit hoeveel suikerklontjes er zitten in …………………………………..

Consult 1: BMI & Volkoren Producten

Consult 1A: Body Mass Index, de BMI
Doel
Het kind/jongere en ouder/verzorger weet wat de Body Mass Index (BMI) betekent.
Het BMI van het kind/jongere wordt berekend en ingevuld op de weeg/meetkaart.

Werkwijze
Je legt uit wat de Body Mass Index betekent. Vervolgens meet je de lengte en weeg je het kind/jongere. Deze gegevens vul je in op de weeg/meet kaart. Met deze gegevens kan je de BMI van het kind/jongere berekenen. De weegschaal die de coach gebruikt berekent dit automatisch. Je kan het BMI ook zelf berekenen door het gewicht te delen door de lengte in meters keer lengte in meters. (1,72 x 1,72 = 2,958. 60 kg: 2,958 = 20,3 BMI).
Vervolgens kijk je in de BMI-tabel (zie bijlage 15 en 16.) in welke graad het kind/jongere zit.
Je legt aan het kind/jongere en zijn/haar ouder/verzorger uit wat deze BMI-waarde inhoud.

Je legt aan het kind/jongere uit dat het ondanks dat het niet afvalt, toch een betere BMI kan krijgen. Dit gebeurt als het gewicht hetzelfde blijft en de lengte toeneemt. Als het gewicht afneemt en de lengte blijft hetzelfde of groeit, neemt het BMI ook af.
Neemt het gewicht toe en de lengte blijft hetzelfde (lengte groeit niet snel genoeg mee conform het gewicht) dan stijgt het BMI. Dit is bij overgewicht/obesitas niet wenselijk.
Je kunt deze uitleg beeldend maken door gebruik te maken van een kneedgum/kauwgompje/elastiekje. (Als een kauwgompje niet groter/dikker wordt, maar wel uitrekt (doordat het groeit), wordt de kauwgom dunner).

Let op; het uitrekenen van het BMI is een belangrijk screen moment. Je analyseert waar het kind/jongere zit in het stroomschema (zie bijlage 1) en observeert of het kind/jongere in het juiste verwijstraject zit. Indien nodig, zorg je dat het kind/jongere door de juiste zorgprofessional(s) gezien wordt. Blijkt bijvoorbeeld dat het kind/jongere graad III obesitas heeft, dan moet er doorverwezen worden naar de kinderarts. Wanner blijkt dat het kind/jongere nog niet bekend is bij een kinderarts dan verwijs je het kind/jongere naar zijn/haar huisarts. Je informeert de huisarts over het kind/jongere wat contact zal gaan opnemen in verband obesitas graad III. De huisarts zal een verwijzing schrijven naar de kinderarts. Het screenen van de BMI gebeurt elk consult opnieuw. Hierdoor kun je als Kindergezondheidcoach op tijd stappen ondernemen.

B.M.I. Body Mass Index

Je bent net een kauwgompje……….

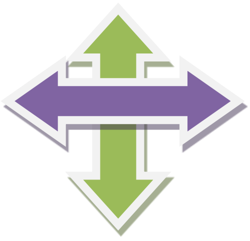

De BMI zegt iets over hoe lang je bent en hoe je gewicht daarover verdeeld is.
Het zegt niks over hoeveel vocht, spier- of vetmassa, je hebt.
We gebruiken de BMI-tabel om te kunnen zien of je een gezond gewicht hebt.

Wanneer je niet op gezond gewicht bent, is je BMI ook niet "gezond".
Je BMI verandert als je langer wordt (groeit) of als je gewicht meer of minder wordt.

Ben je nog niet uitgegroeid (tussen je 15de en 18de stopt je groei) dan kan je lichaam voor een beter gewicht gebruik maken van je groeispurt!!
Zie jezelf maar als een kauwgomballetje. Als je aan het kauwgomballetje trekt, rekt het uit. Het kauwgomballetje wordt dunner. Je hebt niks van het kauwgomballetje afgehaald! Zie de pijl figuren rechts op de bladzijde.

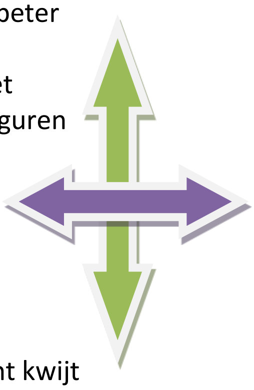

Blijft je gewicht hetzelfde en groei je, dan word je BMI lager.
Dit is een goede manier om naar je gezonde BMI te komen.

Ben je al uitgegroeid en is je BMI te hoog? Dan is het belangrijk om op de juiste manier gewicht kwijt te raken. Het heeft geen zin om heel weinig te gaan eten of extreem te gaan sporten. Dit is zelfs heel ongezond en gevaarlijk!!

Door *goed* en lekker te eten, veel te bewegen en te sporten, voldoende te slapen en te ontspannen, krijg je een gezond en energiek leven. Je BMI zal hierdoor verbeteren.

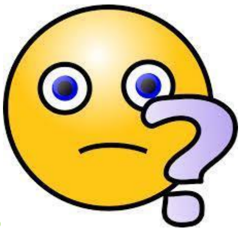

Lekker makkelijk gezegd….

Hiermee ga je nu samen met je coach aan de slag.

Consult 1B Volkoren producten en vezels

Doel

Het kind/jongere en ouder/verzorger weet wat volkoren producten en vezels zijn.
Het kind/jongere en ouder/verzorger weet waarom volkoren producten en vezels belangrijk zijn in een gezond voedingspatroon.

Werkwijze

Je legt uit dat volkoren graanproducten zijn gemaakt van de hele (vermalen) graankorrel. Het vliesje wat om de graankorrel zit wordt niet verwijderd. Denk aan volkorenbrood, volkorenpasta, zilvervliesrijst. Volkoren graanproducten bevatten veel langzame suikers, vezels, ijzer en B-vitamines. Deze zijn nodig om je lichaam gezond te houden en goed te laten werken.

Je legt uit wat het verschil is tussen bruin, volkorenbrood en wit brood (zie foto in consult). Bruin en wit brood zijn niet gemaakt van volkoren meel. Je leert het kind/jongere en ouder/verzorger dat hij/zij altijd moet nagaan of het brood van volkoren meel is gemaakt. Dit kan het kind/jongere en ouder/verzorger navragen aan de bakker of nalezen op het etiket van het brood.
Je legt in dit consult ook uit wat vezels zijn (zie foto in consult). Voedingsvezels (vezels) zijn belangrijke stoffen voor een gezonde spijsvertering. Ze dragen bij aan een verzadigd gevoel na het eten. De vezels zorgen ervoor dat de suikers (koolhydraten) niet snel opgenomen worden, waardoor de suikerspiegel langzaam stijgt en daalt. Vezels zijn er in verschillende soorten en maten. Per dag wordt geadviseerd zo'n 30 tot 40 gram vezels te eten. Volkorenproducten, groenten, fruit en peulvruchten bevatten veel vezels.
Je gebruikt de doppinda als voorbeeld. Om een doppinda zit een velletje net als bij een graankorrel. Dat velletje zijn de vezels om de graankorrel heen. Bij wit brood is het velletje/de vezels weggegooid, bij volkorenbrood is het mee gemalen in het meel

Leg uit dat er een verschil is tussen de opname van suikers bij het eten van fruit en het drinken van een fruitsap. Bij het pureren van fruit maak je de vezels van het fruit kapot waardoor je een snellere opname van suikers krijgt, je slaat namelijk de eerste stap van de vertering (kauwen in de mond) over. Ook wordt de schil vaak verwijderd van het fruit bij het maken van een smoothie of sapje. Juist in de schil zitten veel vezels.
Verder zit er in een smoothie of sapje minimaal 2 of 3 stuks fruit. Je drinkt dus al snel te veel fruit en krijgt hierdoor veel snelle suikers binnen.

Er is een extra consult 'brood'. Bij dit consult kun je nog meer uitleg geven over de verschillende soorten brood, indien wenselijk.

In het consult leg je het werkblad **Vraag en antwoord** uit.
Het kind/jongere mag dit werkblad thuis invullen. De uitwerking en antwoorden staan hieronder beschreven.

Je hebt vast weleens gehoord dat "bruin" brood gezonder is dan wit brood.
Heb jij een idee waarom dat zo is?
Antwoord: In bruinbrood zitten meer vezels.

Wat zijn vezels?
Antwoord: De uitleg doe je met behulp van een doppinda. Om een doppinda zit een velletje net als bij een graankorrel. Dat velletje zijn de vezels om de graankorrel heen. Bij wit brood is het velletje/de vezels weggegooid, bij volkorenbrood is het mee gemalen in het meel.

Volkorenbrood, pasta en zilvervliesrijst

Je hebt vast weleens gehoord dat "bruin" brood gezonder is dan wit brood.
Heb jij een idee waarom dat zo is?

Het gaat er niet om dat het brood **"bruin"** is, maar dat het **volkorenbrood** is. Dit geldt ook voor volkorenpasta of zilvervliesrijst.
Dit zijn allemaal volkoren graanproducten.

Volkoren graanproducten zijn beter voor onze gezondheid dan "witte" graanproducten.
Dit is omdat er in "volkoren eten" veel vezels, vitamines en mineralen zitten.
Vitaminen, mineralen en vezels zijn nodig om je lichaam gezond te houden en goed te laten werken.

Bij witte graanproducten zijn deze goede (voedings)stoffen voor het grootste gedeelte eruit gehaald. Er zitten dus veel minder voedingsstoffen in witte graanproducten waardoor je gezondheid er veel minder aan heeft. Hierdoor krijg je weer sneller honger en eet je er snel te veel van om toch een "vol"gevoel te krijgen.

Wat zijn **vezels?**

Wat doen **vezels?**
In volkoren producten zitten dus veel vezels. Vezels zitten ook in fruit, groente en peulvruchten. Vezels hebben wij nodig voor een goede werking van de darmen. Samen met genoeg drinken zorgen ze ervoor dat je onder andere goed naar het toilet kan gaan…

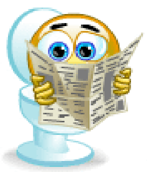

Vezels hebben nog meer leuke weetjes!

- Ze zorgen ervoor dat je tussen de maaltijden door minder snel honger hebt.
- Ze zorgen voor een verzadigd (vol) gevoel.
- Ze leveren bijna geen calorieën op.
- Ze beschermen tegen allerlei ziekten.
- Er zitten veel vitamines en mineralen in die allemaal goed voor ons lichaam zorgen.

Consult 2: Groente en fruit

Consult 2.1: Groente en fruit
Doel
Het kind/jongere en ouder/verzorger weet waarom groente en fruit gezond zijn.
Het kind/jongere en ouder/verzorger krijgt uitleg over vitamines en mineralen.

Werkwijze
Vitamines en mineralen zijn kleine stofjes die ervoor zorgen dat alles goed blijft werken in je lichaam. Ze zorgen ervoor dat je lichaam gezond blijft en goed kan groeien. Het is belangrijk om zoveel mogelijk gevarieerd te eten, want in elk soort groente en fruit zitten andere mineralen en vitamines.
Voorbeelden van de werking van vitamines en mineralen:
Vitamine C is goed voor je weerstand, je wordt daardoor bijvoorbeeld minder snel verkouden. Vitamine D is goed voor sterke botten. Vitamine K zorgt ervoor dat je wondje snel stopt met bloeden. Er zijn in totaal 13 soorten vitamines.
Mineralen zijn nog kleiner dan vitamines. Je lichaam kan niet zonder ze. Het mineraal ijzer is goed voor je bloed (genoeg rode bloedlichaampjes worden aangemaakt), het mineraal calcium is goed voor gezonde stevige botten en gezonde tanden.
Vezels hebben vitamines en mineralen in zich. In het vorige consult heb je het begrip vezels uitgelegd; hier kan je als coach naar terugverwijzen.
In hoofdstuk 9 leggen we meer uit over welke soorten vitamines en mineralen er zijn. In dit consult is het belangrijk dat je als coach het begrip mineralen en vitamines uitlegt (kennis en bewustwording).

Het is beter om fruit in zijn geheel te eten dan gepureerd (zie consult intake bloedsuikerspiegel). Behalve vitamine A, D, E en K zijn alle vitamines in water oplosbaar. Om tijdens de bereiding van groente en fruit zo min mogelijk vitamines verloren te laten gaan, kook je groente/fruit in weinig water en niet te lang. Je spoelt anders de vitamines zo door het gootsteenputje. Soep daarentegen is heel vitaminerijk. Je kunt ook het kookvocht gebruiken voor de rest van het te bereiden eten in plaats van dat je het weggooit.
Je motiveert het kind/jongere en ouder/verzorger een bewuste keuze te maken hoe fruit en groente te bereiden. Je geeft ze ook voorbeelden hoe dit te doen.

In het consult leg je het werkblad **Vraag en antwoord** uit.
Het kind/jongere mag dit werkblad thuis invullen. De uitwerking en antwoorden staan hieronder beschreven.
Waarom denk jij dat groenten en fruit gezond zijn?
Antwoord: Groente en fruit bevatten weinig calorieën. Ze zitten vol met vitamines, mineralen en vezels.

Weet jij hoeveel vitamines er zijn?
Antwoord: Er zijn 13 vitamines. Er zijn vitamines die in water oplossen en vitamines die in vet oplossen (hier komen we later op terug bij consult 9).

Waarom zijn groente en fruit belangrijk?

Die vraag is vast wel eens in je hoofd opgekomen. Waarom denk jij dat groenten en fruit gezond zijn?

Groente en **fruit** bevatten weinig calorieën. Ze zitten vol met vitamines, mineralen en vezels.

Vezels

Vezels geven je een vol gevoel (verzadigingsgevoel). Vezels hebben vitamines en mineralen in zich. Ze zitten in onder andere volkorenbrood, volkoren pasta en zilvervliesrijst. Vezels zorgen ervoor dat je makkelijk voor de grote boodschap naar het toilet kunt.

Mineralen

Mineralen zijn bijvoorbeeld, calcium, kalium, magnesium of ijzer. Dit zijn (voedings-)stoffen die ons lichaam nodig heeft om goed te kunnen werken (functioneren). Bijvoorbeeld calcium is goed voor gezonde stevige botten en gezonde tanden. Magnesium helpt onder andere om spierkrampen te voorkomen.

Vitamines

Vitamines ken je wel, bijvoorbeeld vitamine C of vitamine D. Vitamines zorgen ervoor dat er veel dingen goed blijven gaan in jouw lichaam. Vitamine C is goed voor je weerstand, je wordt daardoor bijvoorbeeld minder snel verkouden. Vitamine D zorgt samen met calcium voor sterke botten. Vitamine K zorgt ervoor dat als je een wondje hebt deze stopt met bloeden.

Weet jij hoeveel vitamines er zijn? **..........**

Kun je verschillende soorten vitamines opschrijven?

Hoeveel groente en fruit zou je elke dag moeten eten?
Antwoord: Minimaal 2 stuks fruit en 200 gram groente.

Er zijn nog twee vragen die je individueel met het kind afneemt.
Lukt het jou om dat elke dag te eten?
Wissel je vaak van groente en fruit?

In elk soort groente en fruit zitten verschillende soorten vitamines en mineralen. Doordat je veel verschillende soorten groente en fruit eet, krijg je dus ook al die verschillende stofjes binnen. Hierdoor blijft jouw lichaam goed werken!

Hoeveel groente en fruit zou je elke dag moeten eten?

Lukt het jou om dat elke dag te eten?

Wissel je vaak van groente en fruit?

Eet lekker veel groente en fruit!

Consult 2.2: Groente en fruit

Doel

Het kind/jongere weet waarom groenten en fruit gezond zijn.
Er vindt een bewustwording plaats bij kind/jongere en ouder/verzorger in het maken van een keuze in fruit en groente ten aanzien van een gezonde leefstijl.

Werkwijze

In dit consult ga je met het kind/jongere na hoeveel soorten groente en fruit hij/zij kent en eet. Je vult dit samen op het werkblad in. Wanneer blijkt dat dit minimaal is, dan leg je nog een keer uit waarom het eten van groente en fruit belangrijk is en welke soorten er zijn.
Je motiveert het kind/jongere en ouder/verzorger om deze week samen naar de winkel te gaan en één soort fruit en één soort groente uit te kiezen. De uitdaging is dat je het gekozen fruit en de groente drie keer gaat eten in deze week.
Je legt uit dat je smaken moet leren eten door te proeven. Proeven is goed kauwen en doorslikken. Je moet minimaal drie keer proeven voordat je een smaak lekker gaat vinden. Dit is belangrijk voor het kind/jongere om te weten.

Wanneer je groente en fruit minder lekker vindt.

Welke soorten fruit of groente eet jij?

Je weet nu waarom groente en fruit belangrijk zijn om gezond te blijven. We gaan nu proberen dat je meer groente en fruit gaat eten. We gaan ook proberen om meer soorten fruit en groente te eten. Dit hoef je natuurlijk niet alleen te doen! We gaan je hiermee helpen.

Deze week mag jij kiezen uit groente en fruit!
Kies in de winkel of bij de groenteboer één stuk fruit en één soort groente die je deze week minstens 3x gaat proberen. Nieuwe smaken moet je namelijk leren proeven! Het duurt soms even voor je ze lekker vindt. Hou daarom vol!

Als je het moeilijk vindt, denk dan vooral aan al die stofjes (vitamines, mineralen en vezels) die ervoor zorgen dat je je:

- **fit** voelt,
- **energie** hebt,
- **beter** kunt **sporten**,
- **minder honger** hebt
- **huid laat stralen**
- **haar laat glanzen.**

Kortom er zijn heel veel redenen om te genieten van groente en fruit!

Consult 2.3: Groente en fruit

Doel

Het kind/jongere en ouder/verzorger weet waarom groente en fruit gezond zijn.
Er vindt een bewustwording plaats bij het kind/jongere en ouder/verzorger in het maken van een keuze in fruit en groente ten aanzien van een gezonde leefstijl.
Het kind/jongere heeft geleerd meerdere soorten fruit en groente te eten.

Werkwijze

In dit consult probeer je het kind/jongere te motiveren om meer gevarieerd groente en fruit te eten in deze week.
Je pakt de fruitkalender in het werkboek erbij. Het kind mag in de vakken van de kalender de gekozen groente en fruit invullen. Ze kunnen aan de hand van smileys aangeven of ze het lekker of vies vonden. Als coach kun je een stickervel met smileys meegeven.
De kalender is gemaakt voor vier weken. Elke week kiest het kind/jongere een ander soort fruit of groente. De bedoeling hiervan is dat het kind/jongere zoveel mogelijk smaken gaat uit proberen.

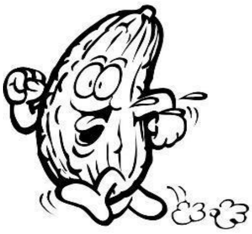
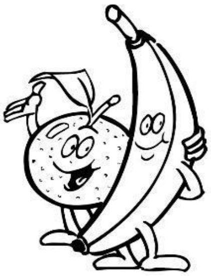
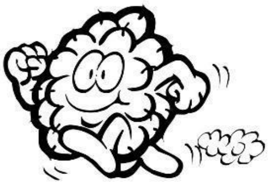
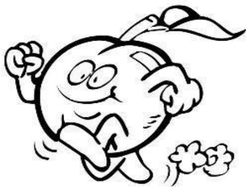

Fruitkalender

Naam:

Vul hieronder in welk soort fruit of groente je hebt geproefd én zet een krul door wat goed ging!

Week 1	Week 2	Week 3	Week 4

Opmerkingen of vragen:

……

……………………………………………………………………………………………………

Consult 3: Het Ontbijt & Water

Consult 3A: Het Ontbijt

Doel
Het kind/jongere en ouder/verzorger weet waarom ontbijten belangrijk is.

Werkwijze:
Je legt het kind/jongere in dit consult het belang van ontbijten uit.
Je hersenen kun je vergelijken met een computer. Je zet door te ontbijten je computer (je hersenen) aan. Van iedere hap eten die je doorslikt, gaat maar liefst 20% van de binnen gekregen energie naar onze hersenen.
Hersenen kunnen niet zoals spieren vet omzetten in energie.
Zij moeten regelmatig voeding krijgen om optimaal te kunnen functioneren.
Verder blijkt dat een gezond ontbijt honger/trek later op de dag voorkomt.
Je kunt het kind/jongere vertellen dat nog steeds een oud gezegde hierbij geldt, namelijk ontbijten als een koning, lunchen als een edelman en dineer als een bedelaar.

In het consult leg je het werkblad **Vraag en antwoord** uit.
Het kind/jongere mag dit werkblad thuis invullen. De uitwerking en antwoorden staan hieronder beschreven.
Wat kunnen hersenen niet en spieren wel?
Antwoord: Vet omzetten in energie.
Een ontbijt is nodig om je computer aan te zetten. Hierdoor zit je bijvoorbeeld “fit” in de klas en ben je minder moe.

Goed ontbijten!

"Een auto rijdt niet zonder benzine en een telefoon werkt niet met een lege batterij. Heel logisch, toch….? Je hersenen werken niet zonder ontbijt, net zo logisch"

Je hersenen zijn jouw computer en regelen alles. Als je hersenen goed werken kun jij bijvoorbeeld veilig op school aankomen en goed opletten op school. Die computer moet aan het begin van de dag natuurlijk wel aangezet worden.

Dat aanzetten van je hersenen doe je door een **Goed Ontbijt!**

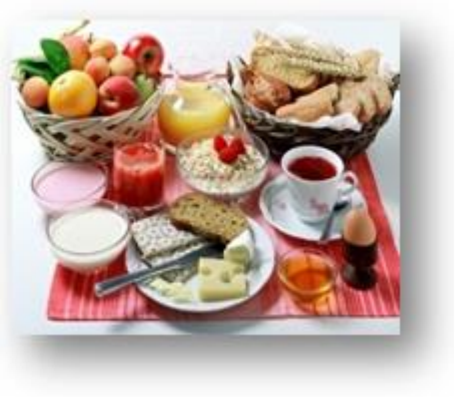

Hersenen bewaren geen energie zoals spieren en hebben na het opstaan dus energie nodig om te kunnen beginnen. In de ochtend willen hersenen vooral energie uit koolhydraten. Wist je dat je van iedere hap eten die je doorslikt, maar liefst 20% van die binnen gekregen energie naar onze hersenen gaat?

Wat kunnen hersenen niet en spieren wel?

Niet of laat ontbijten zorgt ook voor meer honger en trek later op de dag. Het zorgt ervoor dat je snel kiest voor ongezond eten en drinken.

Begin je dag met een goed en lekker ontbijt!

Consult 3A: Het Ontbijt

Doel
Het kind/jongere en ouder/verzorger weet waarom ontbijten belangrijk is.
Het kind/jongere heeft inzicht in zijn eigen ontbijt-patroon.
Het kind/jongere wordt gemotiveerd om iedere dag een gezond ontbijt te nemen.

Werkwijze
Je vraagt aan het kind/jongere wat hij/zij elke dag ontbijt. Ook ga je na wat er ontbeten wordt in het weekend. Het ontbijt in het weekend is vaak anders. Het kan zijn dat het ontbijt in het weekend wordt overgeslagen of een stuk minder gezond is. Leg uit dat dat niet erg is, maar maak het kind/jongere en ouder/verzorger hier wel bewust van. Dit doe je zodat ze erover nadenken en weten dat ze hierin keuzes kunnen (gaan) maken. Dit is een stap in de bewustwording van een gezonde leefstijl.

In het consult leg je het werkblad **Vraag en antwoord** uit.
Het kind/jongere mag dit werkblad thuis invullen. De uitwerking en antwoorden staan hieronder beschreven.
Wat ga je nu elke dag ontbijten?
Het kind/jongere en de ouder/verzorger bedenkt samen met de coach wat een goed ontbijt is en vult dat in.
Wat ga je in het weekend ontbijten?
Het kind/jongere en de ouder/verzorger bedenkt samen met de coach wat een goed ontbijt is voor in het weekend en vult dat in.

Het consult sluit je af met het nog een keer benoemen van wat de voordelen van het eten van een gezond ontbijt is en hoe je kunt variëren.

Het extra consult over brood kun je hier gebruiken ter verdieping.

Wat ga je nu elke dag ontbijten?

En wat ga je ontbijten in het weekend?

Start de dag met een goed ontbijt omdat:

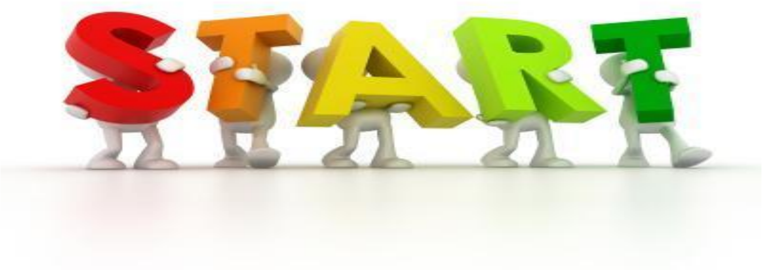

1) Ontbijten geeft je energie
2) Ontbijten helpt voor een goede stoelgang
3) Ontbijten levert voeding- en bouwstoffen op
4) Ontbijten helpt je om een goed gewicht te houden
5) Ontbijten is lekker en een gezellig begin van de dag

Lekkere ontbijt ideetjes:

- Geroosterde volkoren snee brood met boter en verschillend enkel beleg + een glas melk
- Een kom halfvolle yoghurt of kwark met fruit en muesli + kop thee
- Bord havermout of Brinta met warme melk en aardbeien + kop thee
- Glas verse sinaasappelsap met 2 volkoren crackers, boter en beleg
- Gekookt eitje op 1 volkoren beschuit en 1 volkoren beschuit met aardbeien + glas melk
- Quinoa of amandelmeel pannenkoekjes met banaan + thee of melk/karnemelk
- Roerei met een kop thee of karnemelk/melk

Consult 3B: Water

Doel
Het kind/jongere en de ouder/verzorger weet waarom water drinken belangrijk is voor de gezondheid.

Werkwijze
Je legt het kind/jongere uit voor hoeveel procent het lichaam uit water bestaat. Je moet water drinken om dit percentage van water in het lichaam op peil te houden. In veel dranken zit water, maar daarnaast ook veel suikers en andere ingrediënten. Het lichaam kan water het makkelijkst opnemen. Je verliest water uit het lichaam door te plassen, te zweten en te praten. Je bespreekt de zes punten op het gebied van water die beschreven staan op het werkblad. Je legt uit dat water ervoor zorgt dat alle goede stoffen uit je eten en drinken op de juiste plek in je lichaam terecht komen (transport).
Leg het kind/jongere en de ouder/verzorger uit dat het gezond is om ongeveer 1,5 liter water per dag te drinken. Wanneer er te weinig gedronken wordt (bijvoorbeeld een liter in plaats van anderhalve liter) kan het zijn dat je een hongergevoel krijgt, dit is dus geen dorstgevoel. Ook kan je zien aan de donkere kleur van de urine dat er te weinig vocht in het lichaam binnen is gekomen. Wanneer je een dorstgevoel krijgt, ben je eigenlijk *te laat* met drinken.
Verder zorgt water voor een goede stofwisseling. Zonder water kan dat niet gebeuren. Jouw lichaam gebruikt water onder andere om snel extra suikers in je bloed af te voeren. Je gaat je fit voelen door water te drinken.

Een leuk visueel voorbeeld om in te laten zien waarom water drinken goed is: Bloemen in een vaas zonder water gaan hangen. Wanneer je ze water geeft bloeien ze weer op.

In het consult leg je het werkblad **Vraag en antwoord** uit.
Het kind/jongere mag dit werkblad thuis invullen. De uitwerking en antwoorden staan hieronder beschreven:
Hoeveel water drink jij elke dag?
Het kind/jongere geeft aan wat het tot nu toe drinkt aan water per dag.
Hoeveel glazen/flesjes ga je vanaf nu elke dag drinken?
Het kind/jongere maakt samen met de coach een plan om anderhalve liter water per dag te drinken.

Water

Eerst water, de rest komt later!

1) Drinken (vocht) is nodig omdat je lichaam vocht verliest.
   Niet alleen door te plassen, maar ook door te ademen, te praten en natuurlijk door te zweten.
2) Wist je dat ons lichaam voor meer dan de helft uit water bestaat?
3) Ons lichaam kan weken zonder vast voedsel maar ongeveer vijf dagen zonder vocht. Dit komt omdat we geen water kunnen opslaan. Voeding kunnen we wel opslaan, denk maar aan suiker wat opgeslagen wordt in de vorm van vet.
4) Als we te weinig drinken kunnen de nieren geen 'plas' meer aanmaken, je raakt je afvalstoffen niet kwijt. Dit is heel ongezond voor je.
5) Je bloed wordt dikker door te weinig vocht en daardoor moet je hart harder werken om alles rond te pompen.
6) Door te weinig vocht voel je je moe, geprikkeld, minder geconcentreerd, krijg je hoofdpijn, spierpijn en slaap je onrustiger.

Bloemen in een vaas zonder water gaan hangen. Wanneer je ze water geeft bloeien ze weer op. Dit geldt ook voor jouw lijf.

Genoeg redenen om goed water te drinken!

Water geeft je energie: Water zorgt ervoor dat je niet uitdroogt. En water zorgt ervoor dat alle goede stoffen uit je eten en drinken op de juiste plek in je lichaam terecht komen (transport). Water in je lichaam zorgt ook voor een goede stofwisseling! Zonder water kan dat niet gebeuren. Jouw lichaam gebruikt water onder andere om snel extra suikers in je bloed af te voeren. Je gaat je fit voelen door water te drinken.

Water is Goedkoop: Een liter kraanwater kost in Nederland ongeveer 0,07 cent. Een liter cola kost ongeveer €1,50. Tel uit je winst. Bovendien bevat water geen calorieën, suikers of koolhydraten.

Drink 1,5 liter vocht op een dag en het liefst water!

Hoeveel water drink jij elke dag?

Hoeveel glazen/flesjes ga je vanaf nu elke dag drinken?

Consult 3.4: Water

Doel
Het kind/jongere en de ouder/verzorger zijn gemotiveerd om elke dag voldoende water te drinken.

Werkwijze
Je vraagt het kind/jongere of hij/zij het lastig vindt om voldoende water te drinken. Het kind/jongere weet immers nu waarom water drinken goed is. Toch kan het zijn dat kind/jongere moeite heeft om het doel voldoende water drinken te halen. Je gaat na wat de barrières hierin zijn voor het kind/jongere. Je pakt het werkblad erbij en laat tips om meer water te drinken zien. Met deze tips kun je al veel obstakels om weinig te drinken weghalen bij het kind/jongere. Hierdoor kan de motivatie omhooggaan om voldoende water te drinken.
Je stelt vervolgens de vraag aan het kind/jongere of hij/zij denkt dat ze nu helemaal geen suikerhoudende dranken (frisdrank/sapje) meer mogen drinken?
Je bespreekt met het kind/jongere dat dit wel mag maar het liefst in beperkte mate.
Door bovenstaande vragen te stellen en te bespreken kan je vaststellen of het kind/jongere in de afgelopen weken meer water is gaan drinken. Door dit onderwerp water bespreekbaar te maken kun je hierover (nieuwe) afspraken maken met het kind/jongere.

Onderaan het werkblad staat een schema waarin het kind/jongere bij kan houden hoeveel water hij/zij elke dag drinkt. Je kunt dit schema inzetten wanneer het kind/jongere nog niet voldoende water per dag drinkt. Het kind/jongere krijgt hierdoor inzicht in zijn/haar eigen water drinkgedrag. Tevens wordt het kind/jongere gemotiveerd om voldoende water te drinken. Bij jongere kinderen werkt een kleine beloning om voldoende water te drinken aan het einde van de week heel stimulerend. Dit kan in de vorm van een klein kadootje (nieuwe ((duurzame) waterfles) of samen een spelletje te spelen. Laat de ouder/verzorger geen voeding als beloning inzetten!

In het consult leg je het werkblad **Vraag en antwoord** uit.
Je bespreekt het werkblad met het kind/jongere in het consult. De uitwerking en antwoorden staan hieronder beschreven
Fris mag, maar niet zo vaak omdat:
Antwoord: Omdat er veel suiker in frisdrank zit en je hiermee de suikerverslaving stimuleert.

Wist je dat:

- Je door een schijfje citroen, sinaasappel, komkommer, basilicum of blaadje munt, water jouw eigen favoriete smaakje kunt geven?
- Dat er hele leuke milieuvriendelijke waterflesjes en zakjes te koop zijn?
- Water met ijsklontjes, van ranja zonder suiker, heel vrolijk en lekker zijn?
- Er water app's zijn die je helpen herinneren om water te drinken?
- Je een grote fles mooi kunt versieren met je naam erop, die je elke dag kunt gebruiken?
- Je voor 13.00 uur de helft op mag hebben?
- Het niet goed is veel water in één keer te drinken…..?

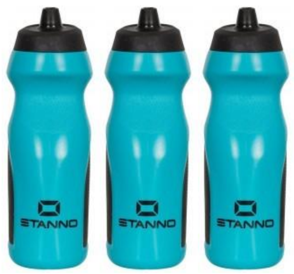

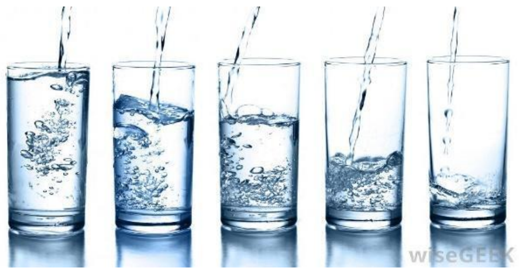

Een keer een glas fris mag natuurlijk best! Maar niet zo vaak omdat:

Hier kun je goed bijhouden of je elke dag wel genoeg **water** drinkt!

Maandag	Dinsdag	Woensdag	Donderdag	Vrijdag	Zaterdag	Zondag

Consult 4: Het stoplicht

Doel
Het kind/jongere en de ouder/verzorger leert wat het verzadigingsgevoel betekent.
Het kind/jongere en de ouder/verzorger herkent het verzadigingsgevoel bij zichzelf.
Het kind/jongere en de ouder/verzorger weet wat het verschil tussen honger en trek is.

Werkwijze
Je legt aan het kind/jongere en ouder/verzorger in dit consult het *verzadigingsgevoel* uit. Voor het kind/jongere kan je het ook een "vol gevoel" noemen. Dit verzadigingsgevoel wordt geregeld door een soort stoplicht in de hersenen. Dit stoplicht is een klein orgaantje in de hersenen dat officieel de hypothalamus heet.
Je legt de werking van het stoplicht aan het kind/jongere en de ouder/verzorger als volgt uit: Wanneer je begint aan een maaltijd staat het stoplicht" op groen. Op dat moment heb je een hongergevoel, want je hebt al een poosje niet gegeten. Tijdens de maaltijd verandert het stoplicht langzaam van groen naar oranje en uiteindelijk naar rood. Rood betekent dat je genoeg/voldoende hebt gegeten. Je hersenen krijgen na 20 minuten na het starten van de maaltijd het seintje dat je verzadigd/vol bent.
Wij eten vaak binnen 20 minuten onze maaltijd op. Zeker de lunch op school is vaak binnen 20 minuten genuttigd. Door dit snelle eten krijg je het signaal "rood" vanuit het stoplicht "te laat".
Eet je (te) snel? Dan eet je dus vaak (te) veel. Je eet niet bewust en weet daardoor niet *dat* en *wat* er gegeten is. Vaak heb je dan na een uur alweer honger (trek).

In Nederland weten veel mensen vaak niet goed meer wat een hongergevoel is. Doordat er altijd en overal eten voorhanden is, eten we vaak al voordat we echte "honger" krijgen.
Je legt het verschil tussen honger en trek uit.
Je begint met het stellen van volgende de vraag aan het kind/jongere: "Weet jij het verschil tussen honger en trek?"
Honger is lichamelijk, je hebt energie nodig om goed te kunnen blijven functioneren. Trek is tussen de oren, je verlangt naar iets lekkers (dikwijls iets zoets). Bij honger eet je "alles", bijvoorbeeld groente en fruit, het maakt niet zoveel uit wat het is.
Je stelt het kind/jongere en de ouder/verzorger gerust dat het kind/jongere bij trek altijd fruit of groente mag eten.
Je geeft het kind/jongere de opdracht mee naar huis om de komende week thuis te "voelen" wanneer hij/zij een hongergevoel heeft en wanneer hij/zij trek heeft.

De jonge kinderen kunnen thuis of tijdens het consult het stoplicht kleuren (dit is een stapje in de bewustwording hierin).

In het consult leg je het werkblad **Vraag en antwoord** uit.
Het kind/jongere mag dit werkblad thuis invullen. De uitwerking en antwoorden staan hieronder beschreven (denk aan de thuisopdracht; herkennen hongergevoel of trek.)
Wat betekent rood?
Antwoord: Rood betekent dat je te veel gegeten hebt (je broek zit te strak)
Wat betekent oranje?
Antwoord: Heb ik nog honger? Of heb ik trek? Ga ik nog wat eten of heb ik genoeg gehad?
Wat betekent groen?
Antwoord: Ik heb honger, ik ga eten (ander gevoel dan trek)

Honger of trek, weet jij het verschil?
Antwoord: Het verschil is dat je bij honger ook zin hebt in een appel of komkommer, het maakt eigenlijk niet uit als je maar wat kunt eten. Bij trek wil je vaak alleen maar iets 'lekkers' eten. Honger heb je als je al een poos niet gegeten hebt. Trek kan je hebben terwijl je net gegeten hebt.

Hoe lang duurt het voor het stoplicht van groen naar rood overgaat bij een maaltijd?
Antwoord: Het duurt twintig minuten voordat je hersenen een seintje krijgen dat je genoeg hebt gehad. Heb je dus binnen tien minuten je bord leeg, dan eet je nog tien minuten langer dan nodig is. Eet daarom rustig en kauw goed. Geniet!!
Een andere tip is eet samen aan tafel.

Het stoplicht,

is jouw verzadigingsgevoel…

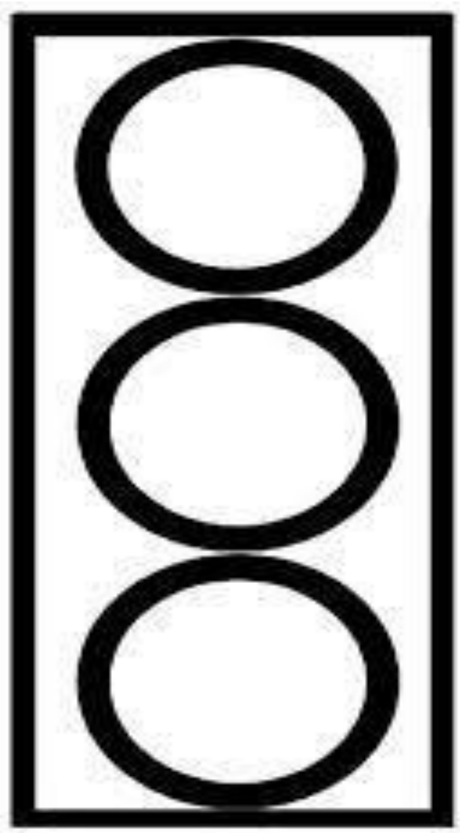

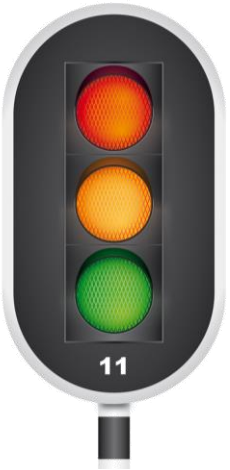

Wat betekent Rood?……………………………………………………………………

Wat betekent Oranje?…………………………………………………………………

Wat betekent Groen?…………………………………………………………………

Honger of trek, weet jij het verschil?

Hoe lang duurt het voor het stoplicht van groen naar rood overgaat bij een maaltijd?

Consult 5: Een terugblik en waar staat het kind/jongere nu?

Doel
Het controleren of de tot nu toe gegeven voorlichting over een gezondere leefstijl begrepen is bij het kind/jongere en ouder/verzorger.
Nagaan of het kind/jongere samen met de ouder/verzorger de verkregen kennis van de vorige consulten toe past in het dagelijks leven van het kind/jongere (gedragsverandering).
Het analyseren en benoemen van eventuele (multi-)problematiek bij het kind/jongere en de ouder/verzorger welke een barrière vormen om de leefstijl te kunnen veranderen.

Werkwijze
Per thema ga je na of de gegeven informatie en de voorlichting over het thema is begrepen en toegepast wordt thuis. Per thema vraag je na hoe het is gegaan en gaat en of ze nog ergens hulp bij nodig hebben (wat gaat makkelijk en wat gaat moeilijk). Je observeert of er eventuele barrières zijn die een gezonde leefstijl in de weg staan. Bijvoorbeeld; financiële problemen, onvoldoende eigen vaardigheden (analfabetisme, onzekerheid etc.), onvoldoende sociale steun etc.

In het consult leg je het werkblad **Vraag en antwoord** uit.
Het kind/jongere mag dit werkblad thuis invullen nadat hij/zij de vragen met de coach doorgenomen heeft. De uitwerking en antwoorden staan hieronder beschreven
Weet jij wat BMI betekent? Wat is jouw BMI?
Antwoord: het BMI vertelt iets over je lengte en gewicht. Verder vertelt het eigenlijk niets. Niet hoeveel spieren, vet of vocht je hebt. De dokters hebben een schema gemaakt wat een gezond BMI voor jouw leeftijd is. Daardoor weten wij of je wel of niet te zwaar bent. Het BMI is eigenlijk een soort kauwgompje (zie blad BMI).

Wat betekent het stoplicht?
Antwoord: het stoplicht is het verzadigingsgevoel. Het geeft aan wanneer je het gevoel hebt dat je genoeg gegeten hebt. Sommige mensen noemen dat ook wel 'vol zitten'. Het legt ook het verschil uit tussen honger en trek. Het stoplicht leert je (opnieuw) te voelen wanneer je genoeg gegeten hebt.

Waarom zijn volkorenbrood, volkorenpasta en zilvervliesrijst gezonder dan wit brood, pasta en rijst?
Antwoord: omdat hier vezels in zitten. Vezels zorgen ervoor dat je lichaam er langer over doet om het eten te verteren. De suiker in je bloed stijgt hierdoor langzaam. In vezels zitten ook vitamines en mineralen.

Waarom zijn een appel en wortel gezond?
Antwoord: dit zijn fruit en groenten. Hierin zitten vezels, mineralen en vitamines die goed zijn voor je lijf en je gezondheid.

Wat is suikerverslaving?
Antwoord: een suikerverslaving is dat je lijf/hersenen steeds weer om iets lekkers vraagt. Je hebt eigenlijk geen honger maar trek. Je hebt net wat gegeten of gedronken, maar toch wil je nog een snoepje, koekje of iets anders lekkers. Als coach leg je hierbij nogmaals het verhaal van de bloedsuikerspiegel uit (zie intake deel 1).

Waarom is het belangrijk dat je goed ontbijt?
Antwoord: Je hersenen kun je vergelijken met een computer. Je zet door te ontbijten je computer (je hersenen) aan. Van iedere hap eten die je doorslikt, gaat maar liefst 20% van de binnen gekregen energie naar onze hersenen. Door te ontbijten voorkom je later op de dag trek en voel je je fitter.

Daarnaast vul je de groeikaart voor het kind/jongere in met de gegevens uit de eerste meting en met de meting van die dag. Let hierbij op de verschillende kaarten per afkomst van het kind/jongere. Er zijn Marokkaanse, Nederlandse, Hindoestaanse en Turks groeikaarten. Ook is er een groeikaart voor kinderen met het syndroom van Down. Een voorbeeld van een groeikaart vind je in bijlage 17.

Wanneer je constateert dat er nog niet veel verandering heeft plaatsgevonden in het eetpatroon van het kind/jongere, kun je hier een extra consult toevoegen. Je kunt hier tevens gebruik maken van consult 6 (Motivatie deelname GO! Gezond Onderweg en visualisatie**)**. Dit consult gebruik je als het kind/jongere zich niet of onvoldoende bewust is van wat hij/zij allemaal eet en drinkt op een dag. Je vraagt het kind/jongere om voor een week een voedingsdagboekje (zie bijlage 20) bij te houden. Het kind/jongere en de ouder/verzorger krijgt hierdoor inzicht in wat hij/zij daadwerkelijk eet en drinkt op een dag.

Verder bespreek je de eventuele problemen/barrières die je observeert bij het kind/jongere aan de hand van de bijpassende consulten in het werkboek. Soms kun je constateren dat het opnieuw bespreken van het consult niet voldoende is. Het kan zijn dat het kind/jongere of de ouder/verzorger een probleem/barrière naar voren brengt wat de begeleiding door GO! in de weg staat. Het probleem of de barrière is dusdanig aanwezig dat een verandering in een gezondere leefstijl op dat moment niet of nauwelijks mogelijk is. Je bespreekt dit dan met het kind/jongere en ouder/verzorger en ook wat de vervolgstap zou kunnen zijn. Denk hierbij aan overleg met en doorverwijzing naar andere collega coaches en zorgverleners in de keten. Tevens beoordeel je of het kind/jongere aan de verwijzingscriteria voor een MDO voldoet. Wanneer dit zo is dan handel je volgens het protocol MDO uit het protocollenboek

Tevens is dit ook het moment dat er een terugkoppeling plaatsvindt naar de huisarts. Daarvoor gebruik je het voorbeeld document wat in de bijlage 19 zit en mailt deze via de beveiligde mail naar de huisarts.

Quizvragen

De afgelopen keren hebben we al veel over een gezonde leefstijl uitgelegd. Niet alles kun je zomaar in één keer onthouden en toepassen. Daarom kijk je samen met je coach naar de volgende vragen. Lukt het jou om de onderstaande vragen te beantwoorden?

Weet jij wat BMI betekent? Wat is jouw BMI?

Wat betekent het stoplicht?

Waarom zijn volkorenbrood, volkorenpasta en zilvervliesrijst gezonder dan wit brood, pasta en rijst?

Waarom zijn een appel en wortel gezond?

Wat is een suikerverslaving?

Waarom is goed ontbijten belangrijk?

Consult 6: Motivatie voor leefstijlverandering en visualisatie

Consult 6.1: Motivatie

Doel
Het kind/jongere en de ouder/verzorger kan benoemen wat zijn/haar motivatie was om mee te doen aan GO!
Het kind/jongere en de ouder/verzorger is voldoende gemotiveerd om het programma GO! te volgen.
Het kind/jongere en de ouder/verzorger weet wat visualiseren is.

Werkwijze
Je vraagt aan het kind/jongere wat de eerste reden (motivatie) was om met het programma GO! te starten. Je gaat verder na bij het kind/jongere of deze redenen op dit moment nog hetzelfde zijn of dat ze anders zijn geworden. Zo ja, wat is er veranderd in de motivatie? Door deze vraag te stellen kun je nagaan of de motivatie bij het kind/jongere voldoende is om door te gaan met het programma. Is de motivatie voldoende dan kun je doorgaan met de uitleg over visualisatie.
Lijkt er geen eigen of weinig motivatie te zijn, dan ga je verder door met vragen waarom dit zo is. Het kan zijn dat het kind/jongere met een andere reden is doorverwezen dan het kind/jongere en de ouder/verzorger zelf dacht. Hierdoor kan er geen eigen motivatie zijn voor deelname. Ga in overleg met het kind/jongere en de ouder/verzorger of ze zich kunnen vinden in de begeleiding door GO! en dat ze inzien waarom de verwijzing naar GO! heeft plaatsgevonden.
Het kan ook zijn dat je door het antwoord op deze vraag de begeleiding minder frequent laat zijn. Er blijft wel contact, maar de afspraken zijn met grotere tussenpozen. Het onderliggend probleem moet eerst aangepakt gaan worden voordat de begeleiding kans van slagen heeft. Wanneer je achterliggende problemen/barrières bij het kind/jongere observeert, koppel je dit terug naar de juiste hulpverlener of verwijzer uit de programmagroep. Mocht het niet goed gaan met het kind/jongere dan kan een kind/jongere besproken worden in een MDO (multidisciplinair overleg). Er kan sprake zijn van Multi problematiek bij het kind/jongere. Je gebruikt hiervoor het stroomdiagram dat in het protocollenboek staat om dit vast te stellen en ook om het kind/jongere goed te kunnen verwijzen naar het MDO.

Vooruitblik/visualisatie
Je gaat met het kind/jongere oefeningen doen op het gebied van visualisatie. Visualisatie is het vertalen van een gedachte naar een beeld, een uitdrukkingsvorm.
Je vraagt aan het kind/jongere wat het voor hem/haar betekent om op gezond gewicht te komen. Wat is er dan anders aan je? Je vraagt aan het kind/jongere er een plaatje in het hoofd van te maken (visualiseren). Door het kind/jongere hierover na te laten denken probeer je het kind/jongere te laten visualiseren hoe hij/zij er dan uit zou zien. Je helpt de visualisatie door de volgende vragen erbij te stellen: Als je een gezond gewicht hebt, wat voor kleren heb je dan aan? Waar ben je en met wie? Wat ben je aan het doen? Deze vragen staan beschreven op het werkblad van het kind. Een plaatje maken van een gedachte kan heel moeilijk zijn voor een kind/jongere om te doen.
Bij de gedachte komt dan niet direct een volledig beeld (plaatje). De volgende oefening kan helpen de visualisatie verder uit te leggen aan het kind/jongere (en ouder/verzorger).
Je vraagt het kind/jongere of hij/zij wil denken aan bijvoorbeeld de keuken in huis. Je vraagt aan het kind/jongere of ze de keuken in "hun hoofd" zien. Zie je ook het koffieapparaat en bijvoorbeeld de koelkast? Waarschijnlijk ziet het kind/jongere dit beeld en kan hij de keuken beschrijven aan je.

In het consult leg je het werkblad **Vraag en antwoord** uit.
Je vult samen met het kind/jongere de vraag in. De uitwerking en antwoorden staan hieronder beschreven
Waarom ben je hier naartoe gekomen?
Antwoord: het kind/jongere legt zijn motivatie uit.

Weet jij wat vooruitkijken (visualiseren) is?
Antwoord: het kind/jongere kan een plaatje in zijn hoofd beschrijven. Je kan als coach hier voorbeelden bij geven.

Motivatie en vooruitkijken

Motivatie….

Waarom ben je hier naartoe gekomen?

Dit is een reden waarom je aan het programma Go! meedoet en het zegt iets over jouw motivatie.

Vooruitkijken….

Weet jij wat vooruitkijken is?

Vooruitkijken is dat je kan bedenken wat er kan gaan gebeuren als je iets doet.

Voorbeelden van vooruitkijken:
Stel je eet elke dag koek, snoep, taart, ijs, chocolade en patat. Hoe zou je er dan na een tijdje uitzien?
Of je wekker gaat, maar je blijft liggen. Wat gebeurt er dan?

En stel nu dat je elke dag Goed & Lekker eet en drinkt, Beweegt & Sport en genoeg Slaap & Ontspanning hebt dan………

Dan heb jij een gezond lijf, fris hoofd en voel je je fit!

Gezondheid = Voeding + Bewegen + Ontspanning

Een Vooruitblik
Wat zou jij nu het allerliefst zien veranderen of zien gebeuren?
Wat betekent voor jou op gezond gewicht zijn?
Hoe zie je er dan uit?
Welke kleren heb je aan?
Waar ben je en met wie?
Wat ben je aan het doen?
KIJK vooruit naar jezelf………

Consult 6.2: Visualisatie leefstijl doel

Doel
Het kind/jongere kan een eigen leefstijl doel maken en beschrijven via een visualisatie oefening.

Werkwijze
Vanuit de eerste intentie/motivatie, de opgedane kennis van de afgelopen weken en wat het kind/jongere graag aan zichzelf graag zou willen veranderen, ga je in dit consult (meer) sturen op een gedragsverandering naar een gezondere leefstijl. Je laat het kind/jongere inzien dat een actie een gevolg heeft. Je gaat dit doen door hierover voorbeelden te geven aan het kind/jongere en ouder/verzorger. Als je het kind/jongere bijvoorbeeld vraagt wat er met zijn/haar lijf gebeurt als er elke dag ongezond gegeten wordt, dan begrijpen ze dat ze daar (te) dik van kunnen worden. Door dit voorbeeld leert het kind/jongere de gevolgen in te zien van wat ze doen. Zo kun je ook het voorbeeld geven dat wanneer ze elke dag gezond zouden eten, genoeg beweging en ontspanning zouden hebben, zij hierdoor een gezond gewicht en een gezonde leefstijl bereiken.
Het kind/jongere mag zijn/haar gevoel over de gevolgen van zijn/haar gezondere leefstijl beschrijven. Je laat het kind/jongere over de volgende hulpvragen nadenken:
Hoe denk ik dat ik er uit zie als ik fitter ben en een gezondere leefstijl heb? Welke kleren draag ik dan? Met wie ben ik? Waar ben ik?
Bijvoorbeeld: jongens tekenen vaak een voetballer, jonge meisjes denken eerder aan een prinsessenjurk die ze passen. Jongeren willen graag meedoen met de laatste trends.
Het kind/jongere gaat thuis hierover een tekening, woordweb, mindmap of moodboard maken.
Achterliggende gedachte in dit consult is dat je als coach het zelfbeeld en het zelfvertrouwen van het kind/jongere probeert te achterhalen.

In het consult leg je het werkblad **Vraag en antwoord** uit.
Het kind/jongere mag dit werkblad thuis invullen. De uitwerking en antwoorden staan hieronder beschreven:
Wat gaat er voor jou anders zijn als je op een gezond gewicht bent?
Antwoord: Het kind/jongere vult hier zijn eigen gedachtes over in.

Ruimte voor: een tekening, woordspin of moodboard. Bij deze (visualisatie)oefening kan je het kind/jongere helpen met de volgende vragen:
- Hoe ziet jouw slaapkamer eruit? Waar staat bijvoorbeeld je bed?
  Er komt nu een plaatje in je hoofd (dit is een visualisatie).
- Nu vraag ik je: denk niet aan een roze olifant. En toch zit hij nu in je hoofd.
- Denk nu aan jezelf op gezond gewicht…. Waarschijnlijk komt er geen plaatje in je hoofd. Dat komt omdat je nog niet geoefend hebt hierin. Je kunt dit gaan oefenen door bijvoorbeeld een tekening erover te maken of een woordspin. Door vaak aan deze tekening of woordspin te denken komt er wel een plaatje in je hoofd. Dit is visualiseren.

Wat gaat er voor jou anders zijn als je op een gezond gewicht bent?

Ruimte voor een tekening/moodboard:

Wat je boven als opdracht hebt gedaan is **visualiseren**.
Visualisatie is een beeld (plaatje) maken van wat je wilt, denkt of leest.

Consult 6.3a: Bewustwording van lastige momenten tijdens de gedragsverandering

Doel
Het kind/jongere en (ouder/verzorger) is zich bewust van eventuele terugval momenten tijdens de gedragsverandering naar een gezondere leefstijl.
Het kind/jongere kan beschrijven welke gedachtes hem/haar kunnen helpen op een lastig moment.

Werkwijze
In dit consult ga je werken aan de bewustwording en het voorkomen van lastige momenten die het kind/jongere kan gaan ervaren tijdens het veranderen van zijn/haar gedrag naar een gezondere leefstijl.
Je vraagt het kind/jongere en (ouder/verzorger) welke momenten hij/zij denkt lastig te gaan vinden om een gezonde leefstijl te gaan uitvoeren (zie vraag één op het werkblad).
Je vraagt vervolgens het kind/jongere (en ouder/verzorger) wat hen zou kunnen helpen tijdens deze lastige momenten. Je leert het kind/jongere te visualiseren hoe hiermee om te gaan (verhogen eigen vaardigheden).
Ook de oplossing kan je laten visualiseren/beschrijven zodoende een terugval te voorkomen.
Je gaat werken vanuit de kracht van het kind/jongere zelf! Je geeft voorbeelden van een terugval moment. Bijvoorbeeld het kind/jongere komt alleen thuis uit school na een niet zo'n leuke dag. Hij/zij ziet een zak chips liggen. Hoe voorkom je dat deze zak chips niet wordt leeggegeten? Je werkt dit uit in een visualisatie oefening en laat het kind dit beschrijven. Je kunt hier ook andere voorbeelden gebruiken zodat het kind/jongere weet wat de bedoeling is van deze vragen.
Je laat het werkblad met de vragen zien en legt uit dat deze thuis ingevuld moet gaan worden.
Belangrijk is dat het werkblad wordt ingevuld op een moment dat het kind/jongere goed in zijn/haar vel zit. Dit is om bij het kind/jongere tot goede positief helpende gedachten te komen.

In het consult leg je het werkblad **Vraag en antwoord** uit.
Het kind/jongere mag dit werkblad thuis invullen. De uitwerking en antwoorden staan hieronder beschreven
Welke momenten vind je lastig?
Antwoord: het is de bedoeling dat hier naar voren komt wat het kind/jongere lastig vindt in het uitvoeren van bijvoorbeeld gezond eten of bewegen. Sommige kinderen/jongeren vinden het bijvoorbeeld heel moeilijk om op een feestje niet te veel te snacken. Een ander voorbeeld is dat het kind/jongere het lastig vindt om uit school te gaan bewegen.

Wat zou je kunnen helpen tijdens deze lastige momenten?
Antwoord: het is de bedoeling dat het kind/jongere zelf nadenkt over wat hij/zij nodig heeft op dat moment om hem/haar te helpen. Het is belangrijk dat het kind/jongere deze momenten kan beschrijven en visualiseert.

Bedenk drie helpende gedachten die jou kunnen helpen tijdens een lastig moment.
Antwoord: bij de helpende gedachten beschrijft het kind/jongere welke gedachten (eventueel activiteiten) hem/haar kunnen helpen tijdens een lastig moment. Dit zijn dan een soort afspraken die het kind/jongere en de ouder/verzorger met zichzelf maakt om het lastige moment goed door te komen en de juiste keuzes voor zichzelf te maken.

Welke momenten vind je lastig?

Wat zou je kunnen helpen tijdens deze lastige momenten?

Bedenk drie helpende gedachten die jou zouden kunnen helpen tijdens een lastig moment!

Consult 6.3b: Strategie “nee” leren zeggen

Doel
Het kind/jongere leert “nee te zeggen” door middel van visualiseren en helpende gedachten hierbij.

Werkwijze
Dit werkblad kun je met het kind/jongere gaan bespreken als de vorige werkbladen (6.3a) door het kind/jongere thuis zijn ingevuld.
Meestal blijkt uit de werkbladen dat “nee” leren zeggen (tegen iets lekkers) voor het kind/jongere lastig is. Het kind/jongere moet vanuit zijn/haar eigen intrinsieke motivatie leren “nee” te zeggen.
Wanneer het kind/jongere bijvoorbeeld zegt: ”nee, dat mag ik niet”, praat het kind/jongere meestal niet vanuit zichzelf en kan het kind/jongere makkelijker overgehaald worden om het *wel* te doen. Het is daarom veel effectiever om te leren praten vanuit jezelf: “Nee, dat wil *ik* niet”.
Middels een visualisatie oefening oefen je “nee zeggen”. Bijvoorbeeld: het kind/jongere krijgt bij een vriendje een snoepje van de ouder aangeboden. Het kind/jongere wil “nee” zeggen. Dit doet het kind/jongere door te zeggen: ik hoef niet. De ouder zal dan waarschijnlijk zeggen: Waarom niet? Neem lekker een snoepje”. Het kind/jongere antwoordt: “dat mag ik niet van mijn mama”. Doordat het kind/jongere praat vanuit de ouder en niet vanuit zichzelf zal het sneller geneigd zijn om te gaan snoepen. Je leert het kind/jongere dat het antwoordt vanuit zichzelf: “nee, dank je, dat *wil ik* niet!”. Bij jongeren kun je benadrukken dat ze ook “nee” leren zeggen tegen hun vrienden. Je leert het kind/jongere aan om op deze momenten te denken aan zijn/haar mindmap, moodboard en woordspin. Hierop staat beschreven de motivatie waarom het kind/jongere zijn/haar gedrag in leefstijl wil veranderen.

In het consult leg je het werkblad **Vraag en antwoord** uit.
Je vult samen met het kind/jongere het werkblad in. De uitwerking en antwoorden staan hieronder beschreven
Wanneer zeg jij nu: “nee, dank je dat wil ik niet”?
Hier pak je het moodboard/mindmap of woordspin 6.2 van het kind/jongere en de helpende gedachten die het kind/jongere opgesteld heeft in hoofdstuk 6.3a er nog eens bij.
Je bespreekt nog een keer wat de helpende gedachten zijn en visualiseert dit met het kind/jongere.

Op welk moment zeg jij nu **“Nee, dank je dat wil *ik* niet”**?

Je mindmap/moodboard of woordspin en de helpende gedachten hierover helpen jou om te kiezen wanneer je “nee, dank je” zegt.
Door vaak te oefenen met vooruit te kijken, na te denken over jezelf komt dit “plaatje” in je hoofd te zitten. Nu visualiseer je.

Consult 7: Sporten en bewegen

Consult 7.1: Wat is het verschil tussen sporten en bewegen

Doel
Het kind/jongere en ouder/verzorger weet het verschil tussen sporten en bewegen.
Het kind/jongere en de ouder/verzorger kent het begrip “de energiebalans”.

Werkwijze
Je vertelt aan het kind/jongere en de ouder/verzorger dat bewegen en sporten net zo belangrijk zijn als goede voeding voor een gezondere leefstijl.
Je vraagt het kind/jongere of hij/zij het verschil weet tussen bewegen en sporten. Je legt dit aan het kind/jongere uit.
Bewegen doe je elke dag. Je beweegt al door binnen of buiten te spelen, trap te lopen, te schommelen, hinkelen of te gymmen. Maar ook door te fietsen of te lopen naar school. Bij bewegen kun je tussendoor praten. Bewegen kun je vaak heel lang volhouden.
Bij intensief bewegen voel je je hartslag sneller slaan, ben je soms buiten adem, kun je moeilijker praten tussendoor, ga je zweten of heb je na die tijd wel eens spierpijn. Dit is **sporten**.
Je legt de richtlijnen van de hoeveelheid bewegen en sporten uit.
Richtlijn bewegen: elke dag één uur. Sporten: 2-3 keer in de week. Van het sporten is 1 keer in de week krachttraining.

Nadat je verschil tussen bewegen en sporten verteld hebt leg je uit waarom sporten en bewegen net zo belangrijk is bij een gezonde leefstijl als goede voeding. Dit doe je door de energiebalans uit te leggen.
Als je meer energie binnenkrijgt dan je lichaam verbruikt, dan blijft er energie ‘over’. Dit teveel aan energie slaat je lichaam op als vet. Als je lange tijd achter elkaar meer energie binnenkrijgt dan je verbruikt, dan neemt je gewicht toe. Krijg je juist minder energie binnen dan je verbruikt, dan val je af.
Bijvoorbeeld: Een auto rijdt als er energie, benzine, in zit. Als er geen benzine in zit kan hij niet goed rijden. Dat geldt ook voor jou. Als je eet en drinkt krijg je energie binnen waardoor je kunt bewegen en sporten.
Wanneer je te veel benzine tankt, loopt de tank over, de auto heeft deze benzine (energie) niet nodig om goed te kunnen rijden. Dat geldt ook voor jou. Eet en drink je (te)veel en beweeg/sport je niet regelmatig dan word je zwaarder, want je lichaam slaat “de rest” op in vet.
Een ander voorbeeld is dat je het lichaam vergelijkt met een oplaadbare batterij. De batterij laadt op door voeding. Hij loopt weer leeg door de energie uit je eten te gebruiken.
Als je meer energie binnenkrijgt dan je lichaam verbruikt, dan blijft er energie ‘over’. Dit teveel aan energie slaat je lichaam op als vet. Als je lange tijd achter elkaar meer energie binnenkrijgt dan je verbruikt, dan neemt je gewicht toe. Krijg je juist minder energie binnen dan je verbruikt, dan val je af.

In het consult leg je het werkblad **Vraag en antwoord** uit.
Het kind/jongere mag dit werkblad thuis invullen. De uitwerking en antwoorden staan hieronder beschreven
Weet jij het verschil tussen bewegen en sporten?
Bewegen is minder intensief dan sporten. Dit verschil kun je zien doordat je bij sporten rode wangen krijgt, je zweet, je buiten adem bent, je je hart snel voelt kloppen en spierpijn kunt hebben de volgende dag. Bewegen doe je eigenlijk de hele dag door, bijvoorbeeld wiebelen op de stoel, lopen naar school. Met bewegen bedoelen wij dat je hele lichaam in beweging is en niet alleen maar je hand.

Waarom is bewegen en sporten net zo belangrijk als goed & lekker eten, voor een gezond en energiek leven?
Antwoord: Wat erin gaat, moet er ook weer uit. Door bewegen maak je het eten op. Dit noemen we met een mooi woord de energiebalans.

Onderaan de pagina van het werkblad staan nog enkele motiverende tips waarom bewegen/sporten echt goed voor je is.

Bewegen en Sporten

Weet jij het verschil tussen bewegen en sporten?

Bewegen doe je elke dag. Je beweegt al door binnen of buiten te spelen, trap te lopen, te schommelen, hinkelen of te gymmen maar ook door te fietsen of te lopen naar school. Bij bewegen kun je tussendoor praten. Bewegen kun je vaak heel lang volhouden.
Bij intensief bewegen voel je je hart sneller slaan, ben je soms buiten adem, kun je moeilijker praten tussendoor, ga je zweten of heb je na het intensief bewegen spierpijn. Dit noem je **sporten**.

Waarom is bewegen en sporten net zo belangrijk als goed & lekker eten,
voor een gezond en energiek leven?

De energiebalans

Bewegen en sporten zorgen ervoor dat je het te veel aan eten/drinken weer opmaakt. Zo voorkom je dat het te veel aan eten en drinken opgeslagen wordt als vet in je lichaam. Dit vet zit vooral op de buik, ook wel het suikerbuikje genoemd.

Voorbeeld: Een auto heeft brandstof (energie) nodig om te kunnen rijden. Als er geen brandstof in zit kan hij niet rijden. Dit geldt ook voor jou. Als je eet en drinkt krijg je brandstof binnen, waardoor je kunt bewegen en sporten.
Wanneer je te veel tankt, loopt de tank over. Dat geldt ook voor jou. Eet en drink (brandstof) je te veel en beweeg/sport je te weinig dan word je zwaarder.

Door elke dag flink te **bewegen** blijft je lijf fit en soepel! Je hebt meer energie én er is nog iets!! Als je regelmatig sport maakt je lichaam een stofje aan, waardoor je je **blijer en gelukkiger** voelt. Dit stofje heet 'Endorfine'. Van dit stofje ga je stralen, heb je minder snel trek en heb je steeds vaker zin om te gaan sporten.

Dus sporten en bewegen zorgen voor:

- Een trots gevoel!
- Een energiek en fit gevoel
- Een gezond lichaam
- Dat je harder kunt rennen
- Een strakker lijf door getrainde spieren
- Minder vet in je lichaam
- Meer spieren waardoor je een hogere verbranding krijgt
- Minder de behoefte aan snacken/snoepen!

Consult 7.2: Het beweegpatroon

Doel

Het kind/jongere en de ouder/verzorger heeft inzicht in zijn/haar eigen beweeg -en sportactiviteiten.
Het kind/jongere en de ouder/verzorger begrijpt dat er bewogen en gesport moet worden om energie te verbranden.
Het kind/jongere en ouder/verzorger weet dat we spieren nodig hebben om te kunnen bewegen.

Werkwijze

In dit consult geef je het kind/jongere en ouder/verzorger inzicht in zijn/haar beweegpatroon. Je vraagt na hoeveel het kind/jongere sport en beweegt. Samen met het kind/jongere ga je na of hun sport/beweegpatroon voldoende is ten opzichte van de richtlijnen op dit gebied. Mocht het sport/beweegpatroon niet voldoende zijn, dan motiveer je het kind/jongere hierin. De coach moet samen met het kind en gezin de sociale kaart ten aanzien van het bewegen en sporten in zijn/haar kern doornemen en het kind stimuleren een sport te kiezen. In bijlage 12 en 13 is een schema waarin je dit kan invullen.

Het werkblad begint met een paar vragen over de spieren in het lichaam. Samen met het kind/jongere en de ouder/verzorger kom je tot antwoorden. Duidelijk moet worden voor het kind/jongere en ouder/verzorger dat je spieren nodig hebt om te kunnen bewegen. Je legt vervolgens uit dat als je rugspier slap is je niet fijn kunt voetballen of gymmen. Ook geef je het voorbeeld van de hartspier. De hartspier heeft de beste conditie maar die moet wel in conditie gehouden worden. Leuk is het om aan het kind/jongere te vertellen dat de bilspier de grootste spier is in het lijf. En als je lacht zijn er meer dan 100 spieren aan het werk en daarom is lachen gezond. Omdat je dan dieper ademhaalt, krijg je ook meer zuurstof binnen! Ook door te lachen wordt endorfine aangemaakt. Endorfine is een pijnstillende stof die het lichaam zelf aanmaakt. Lachen is een mooie beweegoefening. Valt er niks te lachen? Trek je mondhoeken dan 3 minuten op.
Je kunt hier de dobbelsteen uitprinten, voor een extra beweeg spelletje.

In het consult leg je het werkblad **Vraag en antwoord** uit.
Het kind/jongere vult met jou samen het werkblad in. De uitwerking en antwoorden staan hieronder beschreven
Weet jij waar je grootste spieren zitten?
Antwoord: je bil.
Welke spier is het sterkst?
Antwoord: je rug.
Welke spier heeft de beste conditie?
Antwoord: je hart.

Hoe vaak beweeg en sport jij nu?
Je vraagt aan het kind/jongere hoe lang het op dit moment elke dag beweegt en hoeveel hij/zij per week sport.
Je vraagt dit om inzicht te krijgen in wat er op dit moment aan beweegactiviteiten door het kind/jongere gedaan wordt.

Wat zou jij kunnen doen om elke dag 1 uur te bewegen?
Schrijf samen met het kind/jongere op wat hij/zij zou kunnen doen in zijn omgeving, om elke dag een uur te bewegen. Je geeft voorbeelden en tips. Bijvoorbeeld: woon je in een flat? Ga de uitdaging aan om steeds meer verdiepingen met de trap te bereiken. Denk ook aan bewegen in huis (extra trap op en af lopen, touwtjespringen of krachtoefeningen via een app bijvoorbeeld).

En wat zou jij kunnen doen om 2 tot 3 x per week te sporten? (Waarvan 1x krachttraining)
Schrijf samen met het kind/jongere op wat en waar hij/zij zou kunnen gaan sporten.

N.B. Je bent als coach op de hoogte van sportverenigingen/sportclubs in de wijk/het dorp. Denk hierbij aan de beweegmix vanuit GO! zelf. Bijvoorbeeld: wat is er aan naschoolse activiteiten?

Weet jij waar je grootste spieren zitten?..
Welke spier is het sterkst?..
Welke spier heeft de beste conditie?..

Zonder spieren kun je niet bewegen of sporten! Dus trainen die spieren!
Hoe vaak sport of beweeg jij nu?
....
....

Wat zou jij kunnen doen om elke dag 1 uur te bewegen?

Wat zou jij kunnen doen om 2 tot 3x per week te sporten? (Waarvan 1x krachttraining)

Voorbeelden van verbranding van energie:
Om een blikje sinas te verbranden:
Hoeveelheid: 330 ml
Suikerklontjes: 7 (33g/ 135cal)
Wandelen: 54 min
Fietsen: 34 min

Fietsen
Je weegt 60 kilo en fietst 15 km per uur.
Je verbrandt dan 1 twix!

Go For it! Zet hem op!

en vergeet vooral niet te lachen

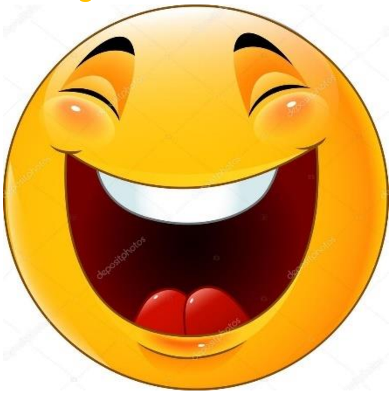

Consult 7.3: Voeding en vocht bij intensief sporten

Doel

Het kind/jongere weet het belang van goede voeding en voldoende vochtinname bij intensief sporten.

Werkwijze

Je vraagt aan het kind/jongere hoe vaak en hoe lang hij/zij sport. Als blijkt dat het kind/jongere intensief sport dan geef je een extra voorlichting over goede voeding en voldoende vochtinname hierbij.

Als een kind/jongere denkt dat hij/zij veel sport, maar het in werkelijkheid wel meevalt maak je het kind/jongere hiervan bewust. Duidelijk moet worden voor het kind/jongere dat het dan niet “extra” hoeft te eten.

Aan de hand van het werkblad geef je de voorlichting over goede voeding en voldoende vochtinname tijdens intensief sporten.

Op de laatste bladzijde van dit consult staat een beweegspelletjes die het kind/jongere zelf thuis kan maken. Je kunt de beweegdobbelsteen goed inzetten om binnen actief te zijn.

Voeding en vocht bij sporten
Bij bewegen en sporten heb je geen extra voeding nodig als je gezond eet en de tips hieronder gebruikt. Als je vaker, langer of heel intensief sport, heb je wel extra voeding nodig. Zeker als je in de groei bent. Heb je vragen hierover? Stel ze gerust aan jouw coach!

- Vocht/drinken is heel belangrijk als je sport, ook al heb je geen dorstgevoel. Niet alleen omdat je vocht verliest omdat je zweet, maar ook om oververhitting te voorkomen. Elk uur dat je sport drink je extra een halve liter, het liefst water. Bij warm weer of veel zweten kun je ook eventueel een dorstlesser (een isotone dorstlesser) drinken, die vult het verlies van zouten weer aan. (Geen energydrank, dit is niet nodig).
- Eet 3 uur voordat je gaat sporten een hoofdmaaltijd. Sport je rond etenstijd? Neem dan een uurtje van tevoren een kleine maaltijd, zoals de helft van je hoofdmaaltijd of bijvoorbeeld een boterham met jam, alvast je toetje of een banaan. Eet een half uur van tevoren niet meer. Je kan namelijk met een volle maag buikpijn krijgen of misselijk worden.
- Energydrank is niet nodig om te drinken! Dit is alleen nodig als je héél lang en zwaar sport. In een energydrank zitten heel veel suiker (soms wel 24 suikerklontjes per flesje). Voel je je slapjes/misselijk, neem dan een beetje ranja met suiker en daarna bijvoorbeeld yoghurt of de andere helft van je maaltijd.
- Drink vóór het sporten niet zoveel dat je buik ervan moet klotsen of dat je ineens moet plassen. Duurt de training langer dan 45 minuten, drink dan tussendoor wel.
- Na de training is magere zuivel met fruit een goede voeding. Zuivel bevat veel eiwitten die je spieren nodig hebben om te herstellen. Fruit is voor de zoete trek. Warme magere chocomelk is ook goed als het heel koud is. Dit alles is veel beter dan een koek, mueslireep of energydrank.

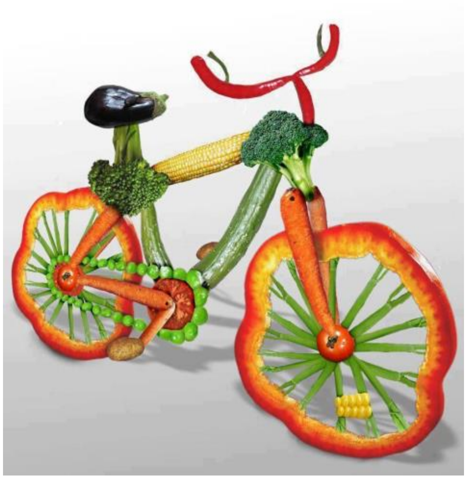

Beweeg dobbelsteen

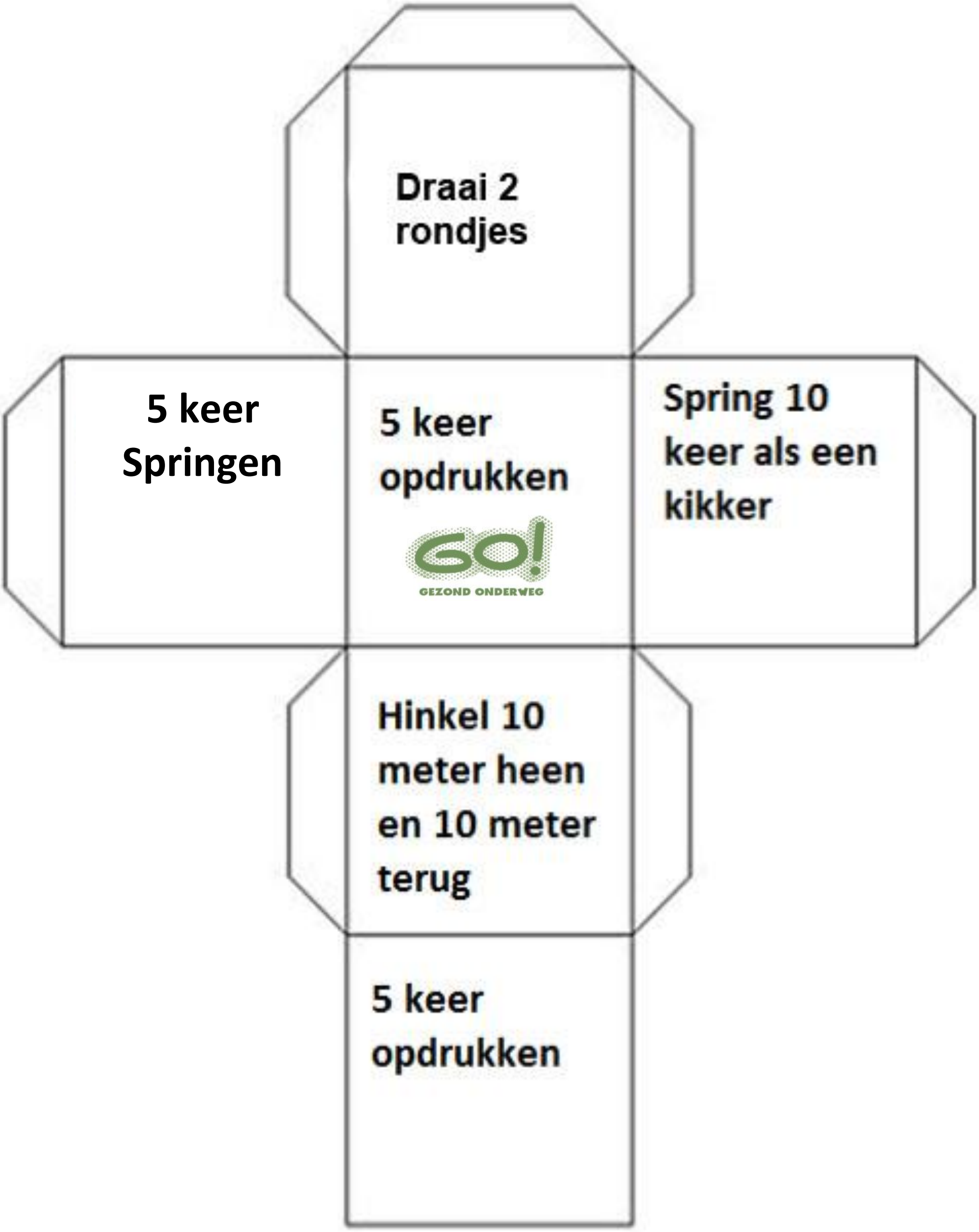

Consult 8 Het belang van voldoende slaap en ontspanning voor een gezonde leefstijl

Consult 8.1: Het belang van voldoende slaap

Doel
Het kind/jongere en de ouder/verzorger weet waarom voldoende slaap en ontspanning belangrijk is voor een gezonde leefstijl.

Werkwijze
Slecht slapen is een voorspeller van overgewicht bij kinderen. Je legt het kind/jongere en ouder/verzorger uit waarom. Door te weinig slaap krijg je minder energie. Wanneer je te weinig geslapen hebt, zul je sneller geneigd zijn om zoetigheid gaan eten. Als je een energietekort voelt wil je “energie” eten. Maar dit is eigenlijk niet waar ons lijf op dat moment om vraagt. Ons lijf vraagt om rust. Ons lichaam doorgaat een hormoonverandering wanneer we te weinig slapen. We maken meer cortisol (stresshormoon) en ghreline aan, dit veroorzaakt een hongergevoel. De productie van leptine neemt af. Leptine regelt de vetafbraak. Doordat er minder leptine gemaakt wordt, is er minder vetafbraak. Daarnaast zorgt leptine ervoor dat je hersenen een seintje krijgen dat je genoeg hebt gegeten.

De richtlijn voor een goede slaaphygiëne is tussen de 9 en 12 uur slaap. Vooral kinderen in de puberteit hebben veel slaap nodig in verband met alle ontwikkelingen die in het lichaam gaande zijn. Slaap is ook belangrijk voor de herstelwerkzaamheden in je lichaam. Verder heeft slaap effect op je humeur.
Te veel slapen is ook niet goed. In de tijd dat je slaapt, beweeg je nauwelijks.

Je geeft het kind adviezen om ontspannen te gaan slapen:
- 2 uur van tevoren geen screens (dus geen iPad/telefoon/spelcomputer). Tv kan wel omdat het verder weg staat)
- douchen voor het slapen gaan werkt ontspannend
- eventueel de dag doorspreken met je ouder/verzorger
- bij piekeren: piekerschriftje/-doosje (hierin schrijf je op wat er allemaal in je gedachten zit, maar waar je nu niets aan kunt doen).

Rust voor het lichaam betekent voldoende slaapuren maken.
Ontspanning is de bezigheid waarvan je uitrust en rustig wordt. Je gedachten kunnen ontspannen. Je hoeft dus niet per se te slapen om te ontspannen. Ontspanning is je relaxed voelen en kunnen zitten zonder iets “te moeten”.

Ontspannen kun je op verschillende manieren doen. Bijvoorbeeld op de bank een boekje lezen, een bad nemen, wandelen in het bos of luisteren naar muziek. Ontspanning kun je ook krijgen door lichamelijke inspanning. Denk hierbij aan een stuk fietsen of rennen/hardlopen. Je denkt dan even nergens anders aan dan waar je op dat moment mee bezig bent. Tijdens ontspanning neemt het stresshormoon cortisol in je lichaam af. Hierdoor neemt ook het hongergevoel af (denk hierbij aan het stoplicht).
Screens (mobiel, televisie- en computerschermen) werken niet ontspannend voor je hersenen, omdat deze veel prikkels afgeven qua beeld/geluid/gebeurtenissen.

Je geeft het kind/jongere tips om te ontspannen: wandelen, in de natuur zijn, douchen/bad, lezen, yoga, mindfulness, (bord)spelletje doen, kleuren, etc.

Als coach ben je alert op eventuele onderliggende problemen voor een goede slaaphygiëne. Dit kan heel eenvoudig op te lossen zijn zoals, minder licht in de kamer, raampje open, niet meer eten voor het slapen. Er kunnen ook onderliggende problemen zijn zoals nachtmerries, moeilijk in slaap komen, veel wakker gedurende de nacht. Je bespreekt dit dan met het kind/jongere en ouder/verzorger en ook wat de vervolgstap zou kunnen zijn. Denk hierbij aan overleg met en doorverwijzing naar andere collega coaches en zorgverleners in de keten.

In het consult leg je het werkblad **Vraag en antwoord** uit.
Het kind/jongere mag dit werkblad thuis invullen. De uitwerking en antwoorden staan hieronder beschreven.
Slaaptekort zorgt voor meer zin in (slecht) eten, omdat je lijf de vermoeidheid voelt als tekort aan energie. Voelen we een energietekort dan willen we energie ETEN!! Maar dat is eigenlijk niet waar ons lijf om vraagt….
Waar vraagt het wel om?
Antwoord: het vraagt om rust. Je moet slapen.

Wanneer doe jij dat?
Antwoord: Het kind/jongere vult hier zelf in wat het doet aan ontspanning.

Slaap en ontspanning

Slaap en rust zijn goed voor je lijf en je hoofd.
Als je genoeg slaapt en ontspanning hebt, kun je beter opletten, voel je je rustig, heb je een beter humeur, heb je voldoende energie en ga zo maar door…
Weet jij er nog één? ……………………………………………………………………………

Slaap heeft ook met een gezonde leefstijl te maken. Door te weinig slaap kun je zelfs zwaarder worden.
Slaaptekort zorgt voor meer zin in (slecht) eten, omdat je lijf de vermoeidheid voelt als tekort aan energie. Voelen we een energietekort dan willen we energie ETEN!! Maar dat is eigenlijk niet waar ons lijf om vraagt….

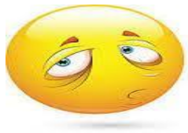

Waar vraagt het wel om?

Ons lichaam doorgaat een hormoonverandering wanneer we te weinig slaap hebben. Dit komt omdat je lichaam - door slaapgebrek- de productie van het hormoon, dat het hongergevoel veroorzaakt, verhoogt. Dit hormoon noemen we ghreline. Hierdoor krijg je meer behoefte aan voedsel. Bovendien neemt de productie van het hormoon (leptine), dat voor vetafbraak zorgt, af. Daarnaast zorgt leptine ervoor dat je hersenen een seintje krijgen dat je genoeg hebt gegeten. Deze effecten zorgen ervoor dat je erg gemakkelijk aankomt door meer en slecht te gaan eten.

Tussen de 9 en 12 uur slaap per nacht is het beste. Maar de voldoende slaap hangt ook van je leeftijd af. Te veel slaap is ook niet goed voor je gewicht, want je beweegt nauwelijks.

Ontspanning en rust
Ontspanning door inspanning kan ook heel goed werken:
Even je hoofd leeg maken door te fietsen, wandelen of hard te lopen.
Bewegen kan ook het effect hebben dat je alles op een rijtje krijgt in je hoofd en je minder piekert.

Bewust ontspannen is wat anders.
We vinden dat we van alles moeten, er wordt veel van ons verwacht door onder andere social media. De mobiel of i-pad wordt veel gebruikt voor filmpjes. Het leven is vaak druk en hectisch. Hierdoor kan het stresshormoon cortisol hoger worden in je lichaam. Het hormoon cortisol zorgt onder andere voor een hongergevoel. Door meer stress ga je vaak meer eten. Bewust ontspannen is relaxen, chillen, ontspannen, even lekker niks….

Wanneer doe jij dat?
……

Consult 8.2: Het belang van ontspanning

Doel

Het kind/jongere weet het belang van ontspanning en kan dit in de praktijk toepassen.

Werkwijze

Je legt het kind/jongere het belang van ontspanning uit. Door te ontspannen krijgen je hersenen minder prikkels binnen en daardoor rust. Ontspannen is niet alleen belangrijk voor je hersenen, maar ook voor de rest van het lichaam. Je lichaam kan herstellen van alle arbeid die het gedaan heeft, denk hierbij aan sporten, huiswerk maken, buiten spelen. Verder groeit je lijf beter bij voldoende rust. Door genoeg slaap en ontspanning zul je minder behoefte hebben aan eten/snacken. Zie 8.1 nogmaals.
Als coach geef je ontspanningsoefeningen. Deze staan op het werkblad.

In het consult leg je het werkblad **Vraag en antwoord** uit.
De uitwerking en antwoorden staan hieronder beschreven. Je vult samen met het kind/jongere en de ouder/verzorger het werkblad in. Je kunt deze vragen ook meegeven naar huis om verder uit te werken.
Welke prikkels komen er bij jou binnen?
Antwoord: vanuit het kind/jongere nadenken wat de redenen zijn dat hij/zij misschien minder goed kan slapen of ontspannen.

Wat zijn oplossingen om minder prikkels te krijgen?
Antwoord: vanuit de eigen gedachten van het kind/jongere oplossingen bedenken om minder prikkels binnen te krijgen.

Ontspannen is goed voor je hersenen.
Je geeft je hersenen rust, ze hoeven even niks. Alle prikkels die je in de loop van de dag binnenkrijgt, kun je even *opruimen*...........

Welke prikkels komen er bij jou binnen?

Wat zijn oplossingen om minder prikkels binnen te krijgen?

Ontspannen is ook goed voor de rest van je lichaam.
Je lichaam kan herstellen van alle arbeid die het gedaan heeft, denk hierbij aan sporten, huiswerk maken en buiten spelen. Verder groeit je lijf beter bij voldoende rust. Door genoeg slaap en ontspanning zul je dus minder behoefte hebben aan eten/snacken

Door meer ontspanning, krijg je meer rust, zal je beter slapen, heb je minder "trek" en is je leefstijl beter in balans!

Tips:
1x per dag 5 diepe ademhalingen doen, voel je buik bij de inademing naar buiten gaan en bij de uitademing naar binnen gaan (leg je hand hierbij op je buik)
1x per dag je kopje thee of melk bewust proeven....
1x per dag naar de lucht kijken....
1x per dag luisteren of de wind waait....
Allen 1 minuut... dat is 4 minuten per dag, 4 minuten gezondheid.

Oefening:
Sta stevig maar ontspannen op beide voeten op heupbreedte met gebogen knieën.
Sluit je ogen....
Haal rustig adem....
Luister....
Voel....
Ruik....

Consult 9 Verdieping van de voedingsstoffen, bouwstoffen en brandstoffen. Gezond eten=voeding

Consult 9.1: Gezond eten = Voeding. Uitleg over de verschillende voedingsstoffen, bouwstoffen en brandstoffen

Doel:

Het kind/jongere en ouder/verzorger weet waarom je bepaalde producten "gezond" noemt.
Het kind/jongere en ouder/verzorger weet enkele voedingsstoffen te benoemen en kan vertellen wat hun werking is.
Het kind/jongere

Werkwijze:

Je legt het kind/jongere uit wat gezond eten inhoudt. Gezond eten is eten waar stoffen in zitten die goed zijn voor je lichaam. Je lichaam heeft er iets aan, deze stoffen zorgen voor een goed werkend lichaam en heldere geest.
Door gezond eten heb je meer energie en voel je je fitter. Je lichaam heeft energie nodig voor onder andere je ademhaling, je lichaamstemperatuur, om goed te kunnen groeien en om bijvoorbeeld goed te kunnen bewegen.
Je vertelt dat we eten en drinken met deze "goede" stoffen erin, voeding noemen. Het voedt je lichaam en geest.
Wat je lichaam nodig heeft, op welk moment van de dag, verschilt elke keer. Hierdoor is het belangrijk om gevarieerd te eten zodat je zoveel mogelijk alle soorten voedingsstoffen binnen krijgt.
Het is belangrijk als coach dat je de voeding afstemt op de leefstijl van het kind/jongere en de ouder/verzorger. Zo creëer je cohesie in het bereiken van een gezonde voedingsinname in het gezin en kunnen eventuele barrières vroegtijdig gesignaleerd worden en besproken.
Je vertelt het kind/jongere dat er verschillende soorten voedingsstoffen zijn. In dit hoofdstuk bespreek je de voedingsstoffen mineralen, vitamines, koolhydraten, eiwitten en vetten. Daarnaast benoem je het belang van water nog eens en de beperkte inname van zout.

Mineralen

Je legt uit wat mineralen zijn. Mineralen zijn zouten. Mineralen zijn hele kleine stofjes in de voeding, die nodig zijn om het lichaam goed te laten werken. Mineralen kunnen niet door het lichaam zelf aangemaakt worden en moeten dus via voeding en drank opgenomen worden door het lichaam. Voorbeelden van mineralen zijn: calcium, kalium of ijzer. IJzer zorgt voor de rode kleur in het bloed dat hemoglobine heet. Hemaglobine vervoert zuurstof van de longen naar de rest van het lichaam. IJzer zit in vlees, groente, fruit volkorenbrood. Calcium is kalk (krijtje) in onze voeding en zorgt ervoor dat botten en tanden stevig blijven. Calcium is ook nodig voor het goed werken van de zenuwen en spieren. Calcium zit in zuivel, groente, noten en peulvruchten. Kalium zorgt ervoor dat de bloeddruk laag blijft. Dit is gezond voor je bloedvaten. Kalium zit in zuivel, groente en fruit.

Vitamines
Je legt uit wat vitamines zijn. Vitamines zijn hele kleine stofjes in de voeding, die nodig zijn om het lichaam goed te laten werken. Vitamines kunnen niet allemaal door het lichaam zelf aangemaakt worden, daarom moeten sommige vitamines door het lichaam via voeding of drank worden opgenomen.
Vitamine C is goed voor je weerstand (een goede weerstand beschermt je tegen virusinfecties.) Vitamine C zit onder andere in rode paprika, kiwi en broccoli. Vitamine K helpt bij het stollen van het bloed als je een wondje hebt. Vitamine K zit in groene bladgroente en olie. Vitamine D wordt door je eigen lichaam aangemaakt als je genoeg zonlicht krijgt. Vitamine D zit verder in vette vis (makreel, zalm haring) en ei.
Verder is het belangrijk om te weten dat Vitamine A, D, E en K vet oplosbare vitamines zijn. De vet oplosbare vitamines worden alleen opgenomen in het lichaam als er goede vetten aanwezig zijn in het lichaam. De andere vitamines zijn in wateroplosbaar. Dit is belangrijk om te weten voor de bereiding van de voeding. Tip: kook daarom groente in weinig water en kook niet te lang.

De voedingsstoffen koolhydraten, eiwitten en vetten staan ook wel bekend als bouw - en brandstoffen. In hoofdstuk 1 heb je uitgelegd dat ons lichaam net als een autobrandstof (benzine) nodig heeft om te kunnen bewegen. Naast de brandstof (benzine) heeft de auto ook andere stoffen (bijvoorbeeld olie) nodig om te goed blijven rijden. Dit geldt ook voor het lichaam. Naast brandstoffen en voedingsstoffen heeft het lichaam ook bouwstoffen nodig. Brandstoffen zijn koolhydraten, eiwitten, vetten en alcohol (NIX18). Bouwstoffen zijn eiwitten vetten en enkele mineralen.

Koolhydraten
Koolhydraten zijn een belangrijke bron van energie voor het lichaam. Er zijn twee soorten koolhydraten. Zie uitleg in intake deel 1 bloedsuikerspiegel. Indien nodig herhaal je deze stof met het kind/jongere en ouder/verzorger. Koolhydraten zitten onder andere in brood, pasta, aardappels, haver, peulvruchten, fruit en melk. Maar ook in gebak, chips, koek en frisdrank (snelle koolhydraten.) Het is belangrijk dat dit verschil van snelle en langzame koolhydraten bekend is bij het kind/jongere en ouder/verzorger om een goede keuze te maken in hun voeding.

Eiwitten
Eiwitten zijn belangrijke bouwstenen voor je lichaam. Eiwitten zorgen voor de ontwikkeling van spieren, botten, organen en klieren. Er bestaan plantaardige en dierlijke eiwitten. Plantaardige eiwitten zitten in peulvruchten, noten bepaalde granen en groente zoals champignons, avocado’s en olijven. Dierlijke eiwitten zitten in vlees,vis, eieren, melk, yoghurt en kaas. Eiwitten zijn goed om te in te nemen na een sportactiviteit om de spieren te helpen herstellen.

Voedsel/voeding

Als je beweegt dan verbruik je energie.

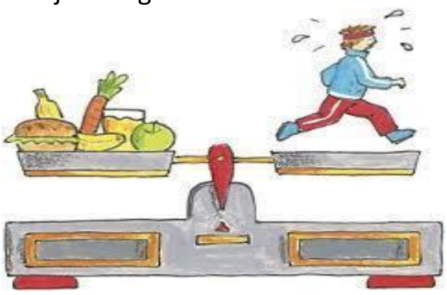

De Energiebalans……

Je lichaam haalt energie uit…..voedsel, eten en drinken. Er is een verschil tussen soorten eten en drinken. Er is gezond eten en drinken en ongezond eten en drinken.

Gezond eten noemen we ook wel *voeding*. Gezond eten is eten waar stoffen in zitten die goed zijn voor je lichaam. Je gaat je namelijk door gezond eten minder moe voelen en je krijgt er energie van. In elk voedingsmiddel zitten weer andere voedingsstoffen. Wat je lichaam nodig heeft op welk moment van de dag verschilt elke keer. Hierdoor is het belangrijk om gevarieerd te eten zodat je elke soort voedingsstof binnen krijgt.

Dit zijn de **voedingsstoffen** die ervoor zorgen dat ons lichaam goed werkt:
1) Mineralen worden ook wel zouten genoemd. Mineralen zijn hele kleine stofjes in de voeding die nodig zijn om het lichaam goed te laten werken. Voorbeelden zijn: calcium, kalium, of ijzer. IJzer is een van de bekendste en zorgt ervoor dat er genoeg rode bloedlichaampjes in je bloed blijven (die zijn belangrijk om zuurstof door je bloed te vervoeren). Calcium is kalk, een krijtje. Calcium zorgt ervoor dat botten en tanden stevig blijven. Calcium zit veel in zuivel.

2) Vitamines ken je wel. Ken jij een voorbeeld van een vitamine?
Vitamine ..of Vitamine ..
Vitamine C is goed voor je weerstand (een goede weerstand beschermt je tegen virusinfecties.) Vitamine C zit onder andere in rode paprika, kiwi en broccoli. Vitamines zorgen ervoor dat veel dingen goed blijven gaan in jouw lichaam. Bijvoorbeeld vitamine D zorgt samen met calcium voor sterke botten. Vitamine K helpt bij het stollen van het bloed als je een wondje hebt. Vitamines zit veel in groenten en fruit, en ook in andere voedingsmiddelen.

3) Koolhydraten zijn een belangrijke bron van energie voor het lichaam. Er zijn snelle en langzame koolhydraten. Weet je nog de uitleg van de snelle koolhydraten in de bloedsuikerspiegel? Langzame koolhydraten zitten o.a. in brood, pasta, aardappels, haver, peulvruchten.

4) Eiwitten zijn belangrijke bouwstenen voor je lichaam. Eiwitten zorgen voor de ontwikkeling van spieren, botten, organen en klieren. Er bestaan plantaardige en dierlijke eiwitten. Plantaardige eiwitten zitten in peulvruchten, noten bepaalde granen en groente zoals champignons, avocado's en olijven. Dierlijke eiwitten zitten in vlees, vis, eieren, melk, yoghurt en kaas. Eiwitten zijn goed om te in te nemen na een sportactiviteit om de spieren te helpen herstellen.

Vetten
Vet levert twee keer zoveel energie als koolhydraten of eiwit. Er zijn twee soorten vetten: verzadigde vetten en onverzadigde vetten. Verzadigde vetten zijn slecht voor je lichaam en daar moet je zo min mogelijk van eten of drinken (bijvoorbeeld geraffineerde en geharde vetten en olie) Dit zit in onder andere in chips, taart, koekjes, chocola en frituurproducten. In veel van deze producten zit naast verzadigde vetten ook transvetten. Deze transvetten zijn ook ongezond voor het lichaam. Bij hoge verhitting van bepaalde olie ontstaat deze transvet. Verzadigd vet blijft hard al je het buiten de koelkast bewaard.
Onverzadigde vetten zijn goede vetten voor het lichaam. Deze vetten zijn nodig om vitamines die in vet oplosbaar zijn op te nemen in het lichaam. Deze vitamines zijn A, D, E en K. Onverzadigde vetten zorgen er ook voor dat je minder kans hebt op hart-en vaatziekten, ze zijn goed voor de werking van de spieren en en functioneren van je hersenen. Onverzadigde vetten komen voor in van nature vette vis zoals zalm, plantaardige oliën, noten en avocado.

Vezels en water horen ook bij een gezonde voeding. In consult 1B is de voedingsstof vezels uitgelegd en consult 3 is water uitgelegd. Belangrijk is dat je als coach deze nog een keer herhaalt/benoemt.

Zout
Zout is het mineraal natrium. Je legt uit aan het kind/jongere dat je 1 á 2 gram zout per dag nodig hebt om gezond te blijven. Gezonde voeding bevat voldoende zout. Het is dus niet nodig dat je aan tafel nog zout toevoegt. Te veel zout kan een hoge bloeddruk veroorzaken. Verder veroorzaakt een te hoge inname van zout dat je vocht vasthoudt waardoor je zwaarder wordt. Zout is een smaakversterker en is daarom veel toegevoegd aan kant en klaar producten en geraffineerde producten. Om je eten smaak te geven kan je (verse) kruiden of (verse) knoflook gebruiken in plaats van zout.

Werkwijze
In het consult leg je het werkblad **Vraag en antwoord** uit.
Het kind/jongere mag dit werkblad thuis invullen. De uitwerking en antwoorden staan hieronder beschreven:
Kan jij een voorbeeld noemen van voeding waar goede vetten inzitten?
Antwoord: Goede vetten: van nature vette vis zoals zalm, plantaardige oliën en avocado.

*Kan jij een voorbeeld noemen van voeding waar slechte vetten inzitten**?***
Antwoord: Slechte vetten: chips, taart, koekjes, chocola en frituurproducten.

5) **Vetten** geven twee keer zoveel energie als koolhydraten of eiwit. Er zijn twee soorten vetten: verzadigde vetten en onverzadigde vetten. Verzadigde vetten zijn slecht voor je lichaam en daar moet je zo min mogelijk van eten of drinken. Onverzadigde vetten zijn goede vetten voor het lichaam. Deze vetten zijn nodig om vitamine A, D, E en K op de goede plek in je lichaam te krijgen.

Kan jij een voorbeeld noemen van voeding waar goede vetten in zitten?

Kun jij een voorbeeld noemen van voeding waar slechte vetten in zitten**?**

6) Water Wist je dat je hele lichaam voor meer dan 70% bestaat uit water?
Een groot deel van je bloed bestaat uit water.

7) Zout wordt, net als suiker, aan bijna al ons eten toegevoegd. Zelfs aan koekjes wordt zout toegevoegd. Gezonde voeding bevat voldoende zout. Het is dus niet nodig dat je aan tafel nog zout toevoegt.
Breng je eten daarom op smaak met kruiden!

Door gevarieerd te eten, krijg je veel verschillende stofjes binnen!
Hierdoor kan jouw lichaam goed werken!

Je eet gezond door elke dag te kiezen uit:

1. **Groente en fruit.** Eet elke dag minimaal 2 stuks fruit, 200 gram groente en elke week een keer peulvruchten in plaats van vlees
2. **Brood of ontbijtgranen, aardappels, rijst, pasta of peulvruchten en vitamines**. Daar zitten koolhydraten, eiwitten, mineralen en vezels in.
3. **Zuivel, vlees(waren), vis, ei of vleesvervangers**. Daar zitten eiwitten, mineralen en (gezonde) vetten in.
4. **Vetten of olie.** Eet daarom 1 handje ongezouten noten per dag.
5. **Vocht.** Het liefst 1.5 liter water per dag.
6. **Minder zout.**

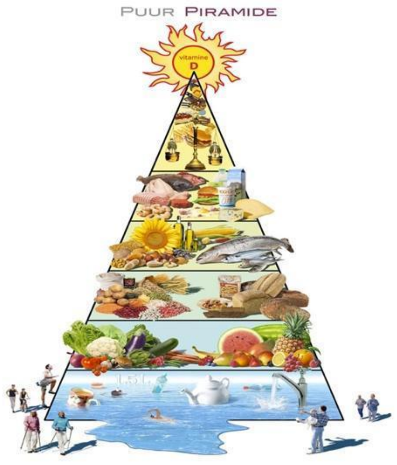

Werkwijze
In het consult leg je het werkblad **Vraag en antwoord** uit.
Het kind/jongere mag dit werkblad thuis invullen. De uitwerking en antwoorden staan hieronder beschreven:
Opdracht invullen Quiz: Wat is gezond eten en wat is ongezond eten?
Antwoord: Kleur het goede antwoord in het desbetreffende balletje.
Het kind/jongere mag deze tabel thuis invullen.

Kan je voorbeelden geven van gezonde voeding?
Antwoord: groente, fruit, vette vis, zuivel, noten, graanproducten.

Kan je ook uitleggen waarom het gezonde voeding is?
Antwoord: Gezond eten is eten waar stoffen in zitten die goed zijn voor je lichaam. Je gaat je namelijk door gezond eten minder moe voelen en je krijgt er energie van. Je lichaam heeft energie nodig voor onder andere je ademhaling, je lichaamstemperatuur en om goed te kunnen bewegen.

Je legt verder uit dat we eten en drinken met deze "goede" stoffen erin, voeding noemen. Wat je lichaam nodig heeft op welk moment verschilt elke keer. Hierdoor is het belangrijk om gevarieerd te eten zodat je elke soort voedingsstof binnen krijgt.

Op het einde van dit consult is de Puur Pyramide toegevoegd. Deze Pyramide doorloop je van onder naar boven. Dit geeft de verhouding tussen voedingsmiddelen aan.

Quiz: Wat is gezond eten, wat is ongezond eten? Kleur het goede antwoord in:

Gezond		Ongezond
O	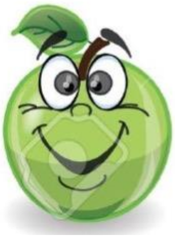	O
O	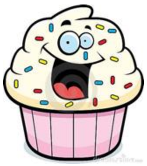	O
O	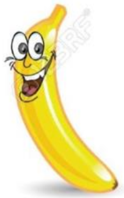	O
O	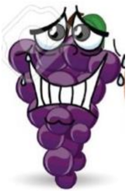	O
O	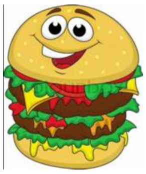	O

Gezond		Ongezond
O	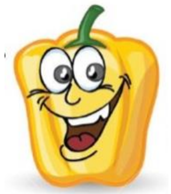	O
O	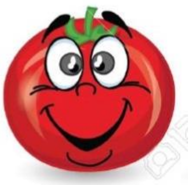	O
O	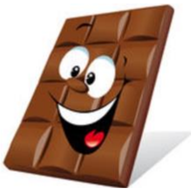	O
O	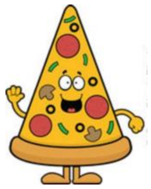	O
O	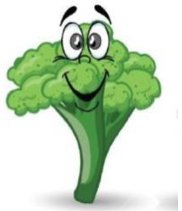	O

Kan je voorbeelden geven van gezonde voeding en waarom het gezond is?

Deze Puur Pyramide doorloop je van onder naar boven. Dit geeft de verhouding tussen voedingsmiddelen aan.

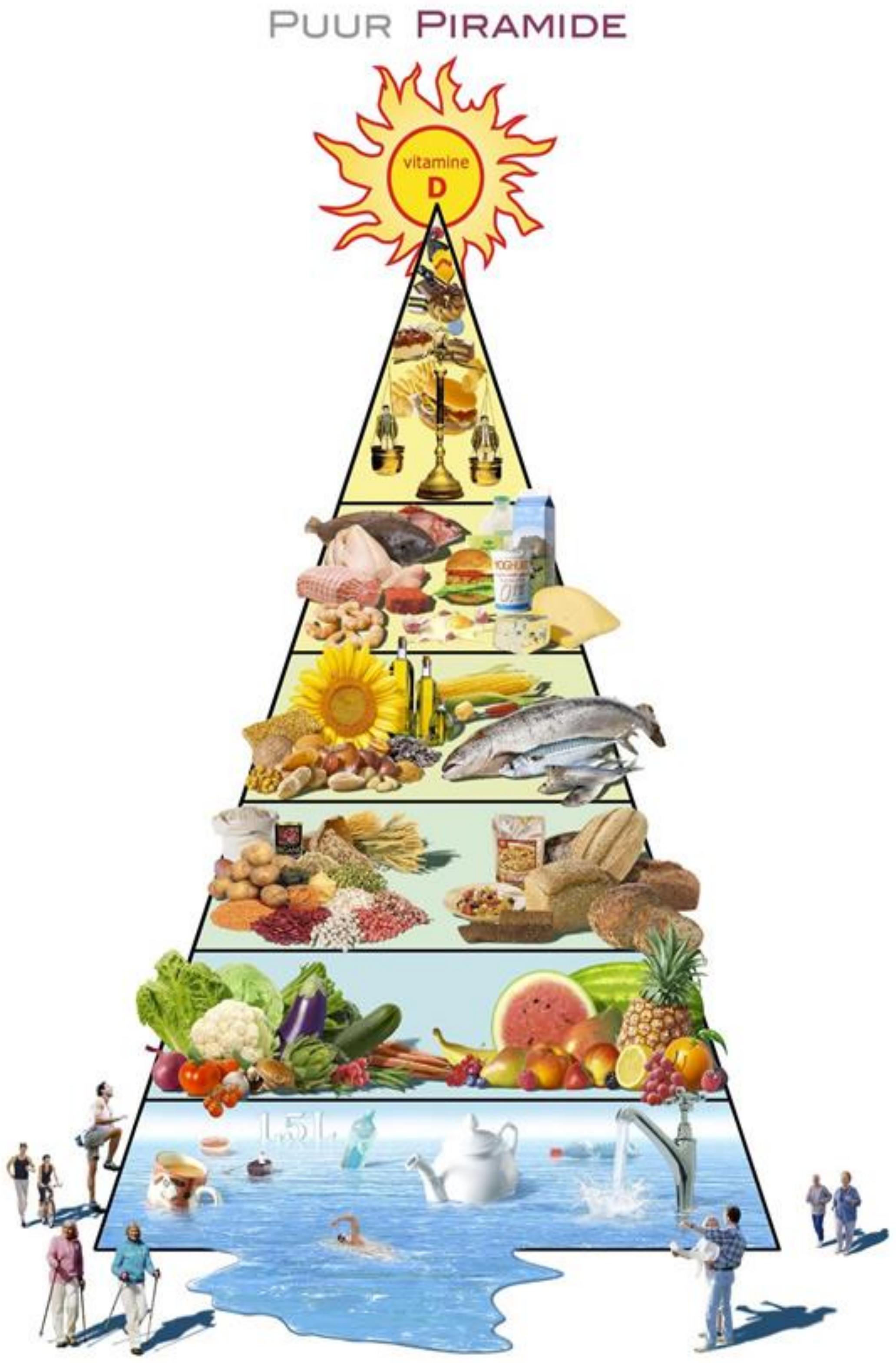

Consult 10: Etiketten lezen en E-nummers

Consult 10.1: Etiketten lezen
Doel
Het kind/jongere en de ouder/verzorger weet wat het belang is van het lezen van etiketten op voedingsmiddelen en kan dit in de praktijk toepassen.

Werkwijze
Je legt uit aan het kind/jongere en de ouder/verzorger hoe het een etiket kan aflezen. Wat er in het product zit aan ingrediënten staat altijd in aflopende hoeveelheid op het etiket. Dus wat als eerste stof op het etiket genoemd wordt zit het meeste in het product. Daarnaast staat er een voedingswaardedeclaratie op het etiket met de hoeveelheden energie, voedingsstoffen, vitamines, mineralen en toevoegingen. Je gaat met het kind/jongere en ouder/verzorger oefenen hoe je etiketten kan aflezen zodoende dat het kind/jongere en de ouder/verzorger dit thuis (en in de winkel) kunnen toepassen.

In het consult leg je het werkblad **Vraag en antwoord** uit.
Het kind/jongere mag dit werkblad thuis invullen. De uitwerking en antwoorden staan hieronder beschreven.
Kijk eens hoe vaak je suiker als een van de eerste ingrediënten tegenkomt in producten.
Antwoord: Het kind/jongere zal ontdekken dat dit heel vaak is. Dit bespreek je vervolgens met het kind/jongere.

Etiketten lezen en E-nummers

Achterop verpakkingen van eten en drinken staan etiketten. Op etiketten staan de stoffen die er in je eten en drinken zitten.
Etiketten lezen, hoe doe je dat?
Om te weten of iets meer of minder gezond is, kijk je achterop de verpakking op het etiket. Bij het lezen van het etiket let je op de volgende punten:

1. Hoeveel calorieën zitten er totaal in het product
2. Zitten er zo min mogelijk snelle suikers in het product
3. Zitten er zo min mogelijk verzadigde vetten in het product
4. Zit er zo min mogelijk zout in het product
5. Zitten er zo veel mogelijk vezels in het product
6. Zitten er zo veel mogelijk vitamines in het product
7. Zitten er zo min mogelijk ingrediënten in het product
8. Zitten er zo min mogelijk E-nummers in het product

Soms word je door het lezen van de etiketten voor de gek gehouden. Bijvoorbeeld: er staat fruit afgebeeld, maar als je het etiket op de achterkant leest, kom je niet het woord fruit tegen maar bijvoorbeeld vruchtensap.
De stof die het meest voorkomt in het eten of drinken staat bovenaan op het etiket. De stof die het minste voorkomt staat als laatste op het etiket.

Opdracht:
Kijk eens hoe vaak je suiker als een van de eerste ingrediënten tegenkomt in producten.

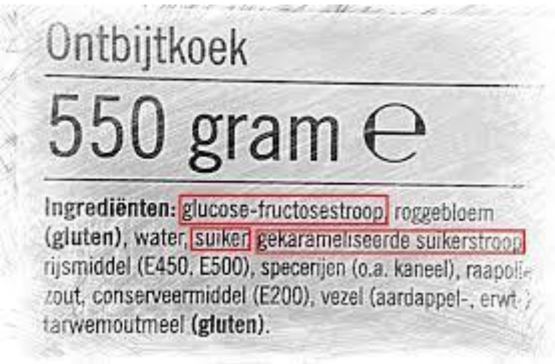

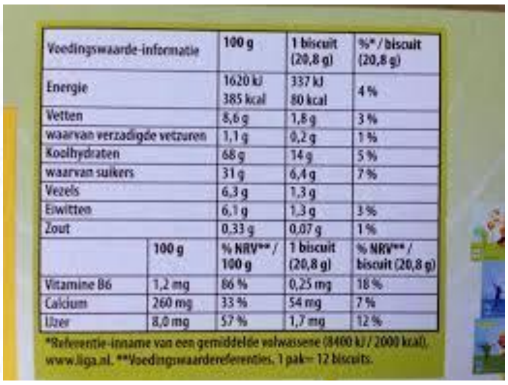

Consult 10.2: Wat zijn E-nummers
Doel
Het kind/jongere en de ouder/verzorger weet wat E-nummers zijn en kent de smaakversterker E621.

Werkwijze
Je legt nogmaals aan het kind/jongere uit dat achterop de verpakking van voedingsmiddelen een etiket zit waarop staat beschreven welke stoffen er in het product zitten. Er staan veel stoffen op die vaak onbekend zijn voor het kind/jongere en ouder/verzorger. Van deze stoffen gaan we er één behandelen genaamd: Umami/mononatriumglutamaat/E621.
Het stofje E621 is een smaakversterker. Dit stofje zorgt ervoor dat wat je eet je heel smakelijk en lekker vindt. Het geeft echter niet aan dat je genoeg hebt gehad.
Dit stofje zit in heel veel producten, maar voornamelijk in kant en klaar maaltijden en bewerkte producten zoals in een pot kant en klare spaghettisaus en noodle soepen.
Wanneer je deze bewerkte producten gaat eten, zul je meer eten dan eigenlijk goed voor je is. Het stofje zorgt er namelijk voor dat je blijft dooreten. Kun je deze E-nummers vermijden in je voeding? Helaas niet, maar door vooral verse onbewerkte en natuurlijke producten te gebruiken tijdens het koken voorkom je dat je te veel van deze stof binnen krijgt. Let ook op dat de tussendoortjes voornamelijk verse onbewerkte producten zijn. Er zullen altijd momenten zijn dat er bewerkte producten gegeten worden. Vertel het kind/jongere dat dat niet erg is maar maak het kind/jongere bewust dat tijdens het eten van bewerkte producten het zijn/haar stoplicht niet voelt.

E 620 t/m 635, E640 en E650 zijn allemaal smaakversterkers.

Je vertelt waar de stof umami (E621) vandaan komt en legt samen uit aan met het kind/jongere en de ouder/verzorger de andere smaken uit.

In het consult leg je het werkblad **Vraag en antwoord** uit.
Samen met het kind/jongere vul je het werkblad in. De uitwerking en antwoorden staan hieronder beschreven:
Weet jij welke 4 andere smaken je kunt proeven?
Antwoord: zout, zoet, bitter en zuur.

Je weet nu dat E621 vaak in je voeding zit. Door je er bewust van te zijn, kun jij er mee omgaan. Jij bent sterker dan E621!
Schrijf hierop hoe?
Antwoord: Doordat je bewust bent van het feit dat je een product eet waar E621 in zit kun je met jezelf de afspraak maken hoeveel je er maximaal van gaat eten. Zo voorkom je dat je teveel eet omdat je je stopknop niet voelt.

Heb jij dit weleens meegemaakt?

- Ik eet soms zoveel dat ik mijn riem een gaatje wijder moet zetten.
- Als we chinees gaan eten, kan ik er geen genoeg van krijgen.
- Je kunt gewoon niet stoppen tot de zak chips helemaal leeg is, whooooaaa!!!

Dit overkomt je ook met sommige koekjes, (light-)chips, pizza, sauzen, pakjes kruiden, soep van poeder en veel kant-en-klaar maaltijden. Dit eten en drinken smaakt ook allemaal zo lekker! Als je achterop de verpakking kijkt bij de ingrediënten zie je een nummer staan, E621.

***…E621 is een smaakversterker*….**

Op de verpakking kan je ook bijvoorbeeld mononatriumglutamaat of glutaminezuur (E620 t/m 625) zien staan.
Allemaal hele moeilijke woorden die dezelfde werking als de smaakversterker E621 hebben. Deze stoffen zorgen ervoor dat jij meer van het product gaat eten!
Je kunt niet meer voelen dat je genoeg hebt gehad. Je stoplicht blijft op oranje staan.

Waar komt het vandaan?
Natrium glutamaat is ook bekend als het Chinese ve-tsin. Het is lang geleden ontdekt in Japan waar het uit zeewier wordt gehaald. Het wordt gebruikt om eten te kruiden. Het heeft de smaak Umami, dit is onze 5de smaak op de tong. Weet jij welke 4 andere smaken je kunt proeven?

Natrium glutamaat, E620 t/m E625, schakelt je verzadigingsgevoel uit. Je kunt eenvoudigweg niet stoppen met eten. Toch zou het jammer én heel moeilijk zijn als je nooit meer die lekkere snacks zou kunnen eten of nooit meer soep of saus uit een pakje kan nemen. Doordat je nu bewust bent van het feit dat je iets eet waar E621 in zit, kun je met jezelf hierover een afspraak maken. Zo voorkom je dat je teveel eet omdat je je stoplicht niet voelt. Jij bent sterker dan E621! Schrijf hierop welke afspraak je maakt met jezelf.

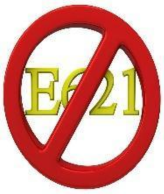

Consult 11: Alles op een rijtje

Doel
Het kind/jongere is met de verkregen kennis van de vorige consulten aan de slag gegaan en past dit toe in zijn eet-drink- slaap en beweegpatroon.

Werkwijze
Je vraagt het kind/jongere en de ouder/verzorger hoe hij/zij de afgelopen periode heeft ervaren. De volgende vragen gebruik je hiervoor:

- Zie je een verandering in je leefstijl sinds je bent begonnen met het programma GO!?
- Zijn de eventuele (onderliggende) problemen opgepakt?
- Is er een positieve verandering in de gezonde leefstijl?
- Klopt het in consult 6 gemaakte doel nog steeds? Wil je hier aanpassingen in maken?
- Zijn er thema's die nog je steeds lastig vindt? Bijvoorbeeld "nee" zeggen?

Je evalueert samen met het kind/jongere en de ouder/verzorger de deelname aan GO! in de afgelopen maanden. Met het kind/jongere en de ouder/verzorger loop je de werkbladen door.
Verder bespreek je de eventuele problemen/barrières die je observeert bij het kind/jongere aan de hand van de bijpassende consulten in het werkboek. Je loopt het desbetreffende consult nog een keer met het kind/jongere door. Soms kun je constateren dat het opnieuw bespreken van het consult niet voldoende is. Je bespreekt dit dan met het kind/jongere en ouder/verzorger en ook wat de vervolgstap zou kunnen zijn. Denk hierbij aan overleg met en doorverwijzing naar andere collega coaches en zorgverleners in de keten. Ook denk je hier weer aan verwijzingscriteria voor een MDO.
Je pakt vervolgens de groeicurves erbij in het werkboek van het kind/jongere.
Je vult de laatste meetgegevens van het kind/jongere in (BMI, lengte en gewicht). Nu kan je het kind/jongere laten zien waar hij/zij nu in de groeicurve is. Dit kan confronterend of motiverend zijn!
Let hierbij op de verschillende groeicurves per afkomst van het kind/jongere.

ALLES OP EEN RIJTJE

- Water, de rest komt later…………flesjes!
- Slaap voldoende en neem je rust.
- Beweeg elke dag, ook als het regent.
- Eet goed én lekker.
- Voel je verzadigingsgevoel (stoplicht).
- Groente en/of fruit tussendoor mag altijd.
- Ontbijt elke ochtend.
- Eet aan tafel, rustig, goed kauwen, *weet* dat je eet.
- Ga niet naar de winkel met honger of trek.
- Zorg dat je een suikervrij kauwgompje bij je hebt voor moeilijke momenten.
- Neem geen geld mee naar school.
- Plan je snack-momenten, kijk vooruit.
- Sport 2 a 3x per week.

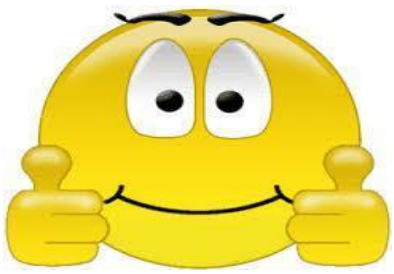

Consult 12: Afsluiting GO! Gezond onderweg

Doel
Het kind/jongere en de ouder/verzorger hebben geen begeleiding meer nodig van GO! en kunnen zelfstandig verder met hun nieuwe gezonde leefstijl.
Het kind/jongere is zich bewust van zijn nieuwe gezonde leefstijl.

Werkwijze
Als kindergezondheidscoach sluit je de begeleiding met het kind/jongere en ouder/verzorger af.
Je evalueert hoe ze de begeleiding hebben ervaren en wat het hen gebracht heeft.
Je sluit af met het doornemen van het allerlaatste werkblad.

In het consult leg je het werkblad **Vraag en antwoord** uit.
Het kind/jongere mag dit werkblad thuis invullen. De uitwerking en antwoorden staan hieronder beschreven
Weet jij nog waarom je hiernaar toe gekomen bent? Terugdenken aan de beginsituatie en aan wat er veranderd is.

Kun jij de 5 beste dingen, die voor jou zijn veranderd, opschrijven?
……………………….

Hoe kun jij ervoor zorgen dat deze positieve veranderingen blijven?

5 veranderingen: dit zijn vaak dingen die ze nog moeilijk vinden.
5 oplossingen: hier geven ze aan hoe ze de veranderingen in stand kunnen houden, vanuit hun eigen kracht.

Belangrijk: aangeven dat de jongeren na dit consult altijd nog mogen bellen/mailen bij vragen of problemen.

Wat kan jij nu allemaal?

Een poosje geleden ben jij begonnen bij GO! vandaag is de laatste keer.

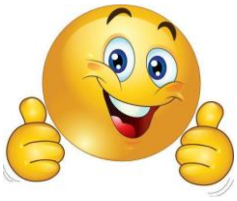

Weet jij nog waarom je hier naartoe gekomen bent?

Kun jij de 5 beste dingen, die voor jou zijn veranderd, opschrijven?
1)..
2)..
3)..
4)..
5)..

Hoe kun jij ervoor zorgen dat deze positieve veranderingen blijven?
1)..
2)..
3)..
4)..
5)..

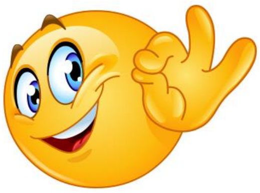

Nu heb jij je eigen steuntje in de rug gemaakt.

Je mag super trots zijn op wat je allemaal hebt bereikt!
Dit heb je zelf gedaan!
Natuurlijk ook met een beetje hulp en steun van de mensen om jou heen ging jij gezond onderweg.
Jij weet nu *hoe* je moet kiezen en *dat* je kunt kiezen en daardoor sta je sterk.
Blijf dit doen voor jezelf, want jij bent een gezond en energiek leven meer dan waard!

Extra Consulten

Extra consult: Brood

Doel:
Het kind/jongere krijgt uitleg over graansoorten en broodsoorten.
Er vindt een bewustwording plaats bij kind/jongere en ouder/verzorger in het maken van een keuze in brood ten aanzien van een gezonde leefstijl.

Werkwijze:
Dit consult is een aanvulling op het consult 2.1 volkoren producten en vezels en het consult 3.1 ontbijt**.** Je legt als kindergezondheidscoach het kind/jongere uit dat er verschillende soorten granen zijn waarvan meel wordt gemaakt.
Er zijn diverse soorten graan zoals bijvoorbeeld tarwe, gerst, rogge en spelt. Van elke graansoort kan meel voor brood worden gemaakt. De meest voorkomende meelsoort is die van tarwe.
Graankorrel:

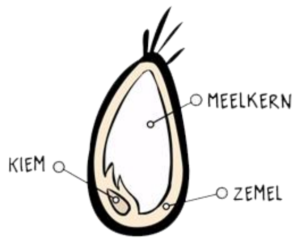

De meeste mensen eten brood dat gemaakt is van tarwebloem, meel of volkorenmeel. Waar je brood van is gemaakt kan je lezen op het etiket of kan je vragen aan de bakker.
Vervolgens leg je als kindergezondheidscoach de verschillende meelsoorten uit.
Bloem wordt gemaakt van uitsluitend het binnenste deel van de graankorrel; de meelkern. Alle overige onderdelen zoals de zemelen en de kiem worden er na het malen uit gezeefd waardoor er een wit graan poeder overblijft. Bloem wordt gebruikt voor de bereiding van witbrood.
Volkorenmeel bevat alle onderdelen van de graankorrel, dus de meelkern, de kiem en de zemel in de natuurlijke verhouding. In volkorenmeel zijn de zemelen met het blote oog zichtbaar. Volkorenmeel wordt gebruikt bij de bereiding van volkorenbrood.
Meel is een combinatie van bloem en volkorenmeel. In meel zijn de zemelen met het blote oog zichtbaar. Meel wordt gebruikt bij het bereiden van bruinbrood. Bruinbrood adviseren wij liever niet aan het kind/jongere.

Volkoren is belangrijk in ons eten omdat de graankorrel van nature waardevolle *voedings*stoffen (stoffen die iets goed doen voor je lichaam en helpen jou gezond te blijven) bevat.
Dit komt omdat de graankorrel eigenlijk het zaadje is waaruit een nieuwe plant kan groeien. Dit zaadje heeft alle stoffen in zich die nodig zijn voor het jonge plantje. Deze stoffen zijn ook goed voor de mens. Dan snap jij nu dat het slim is om de hele graankorrel te eten!

Voor volkorenbrood wordt de hele tarwekorrel gebruikt, in wit en bruinbrood niet.
Volkorenbrood heeft de meeste voedingsvezels, vitamines en mineralen. De definitie voor

volkoren staat vastgelegd in de warenwet: de hele korrel moet verwerkt zijn om het volkoren te mogen noemen. Je weet dus zeker dat als er volkoren op het etiket staat dat het *echt* volkorenbrood is.

- 1 snee volkorenbrood bevat gemiddeld 2,3 gram voedingsvezels
- witbrood bevat gemiddeld 0,8 gram voedingsvezels per snee
- bruinbrood bevat gemiddeld 1,8 gram voedingsvezels per snee.

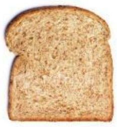

Meergranenbrood is niet per definitie volkorenbrood. Dit brood kan van vol[illegible]el gemaakt zijn, maar ook van bloem voor wit brood. In dat geval zal het brood minder voedingsstoffen en dus ook minder vezels bevatten dan volkorenbrood. Aan het meergranenbrood zijn gebroken of geplette graankorrels en zaden toegevoegd. Door deze granen en zaden lijkt het brood gezonder maar het bevat nog steeds minder voedingsstoffen dan volkorenbrood.

Moutmeel is gemaakt van gekiemde tarwe- en gerstekorrels. Deze granen laat men kiemen in een vochtige omgeving. Tijdens het kiemen wordt het zetmeel omgezet in maltose. Maltose is een suiker en zorgt voor een zoete smaak, maar het zorgt er ook voor dat het brood beter kan rijzen en dat het mals blijft. Daarom voegt men soms mout toe aan brood. Resultaat: moutmeel, dat brood op een natuurlijke manier een donkere kleur en een lichtzoete smaak geeft. Deze donkere kleur zegt niks over hoe gezond het brood is of dat het volkoren is.

Spelt is een oud tarwe-ras. Spelt levert een eiwitrijk meel met een hoog glutengehalte. Dit hoge gehalte aan gluten zorgt ervoor, dat spelt niet geschikt is voor mensen met coeliakie (gluten-intolerantie), maar wel bijzonder geschikt is voor mensen met een tarwe allergie.

In het consult leg je het werkblad **Vraag en antwoord** uit.
Het kind/jongere mag dit werkblad thuis invullen. De uitwerking en antwoorden staan hieronder beschreven. De uitwerking en antwoorden staan hieronder beschreven:

Welk soort brood eet jij op dit moment vaak?
Antwoord: Het kind/jongere vult het antwoord thuis in.
Weet jij wat het verschil is tussen wit, bruin en volkorenbrood?
Antwoord: Wit brood is gemaakt van bloem en bevat nauwelijks voedingsstoffen en vezels. Bruinbrood is gemaakt van volkorenmeel en bloem. Dit brood bevat iets meer voedingsstoffen en vezels dan wit brood. Volkorenbrood is gemaakt van volkorenmeel en bevat veel voedingsstoffen en vezels.
Nu je dit allemaal weet over brood, welke broodsoort kies je en ga je vaak eten?
Antwoord: Het kind/jongere vult het antwoord thuis in.

BROOD

In Nederland eten we veel brood. We ontbijten ermee en eten tussen de middag als lunch ook vaak brood. Soms eten we ook bij het avondeten brood.
Maar wat is nu eigenlijk brood? Wat zit erin? Waarom is het goed voor jou?
Dat gaan we uitleggen!

Welk soort brood eet jij op dit moment vaak?

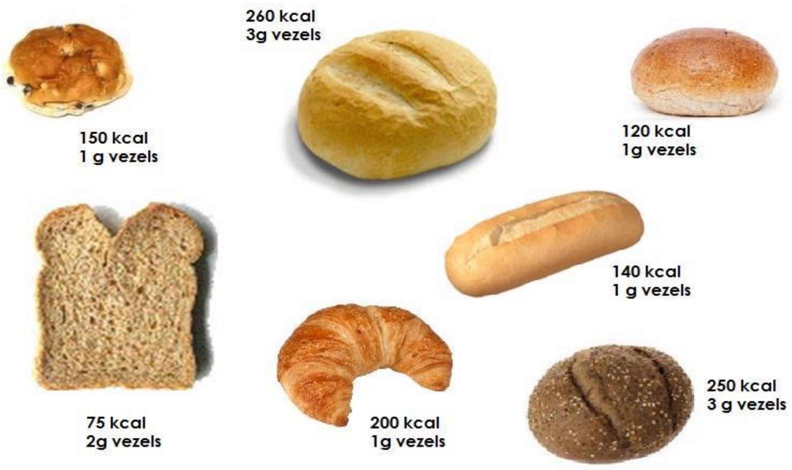

Brood is gemaakt van graan.
Er zijn diverse soorten graan zoals bijvoorbeeld tarwe, gerst, rogge en spelt. Van elke graansoort kan meel voor brood worden gemaakt. De meest voorkomende meelsoort is die van tarwe.

Graankorrel:

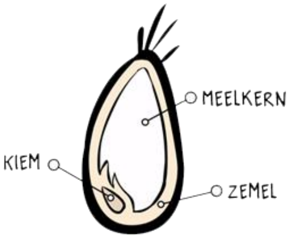

De meeste mensen eten brood dat gemaakt is van bloem, volkorenmeel of meel. Waar je brood van is gemaakt kan je lezen op het etiket of vragen aan de bakker.
Weet jij wat het verschil is tussen wit, bruin en volkorenbrood?

Bloem wordt gemaakt van uitsluitend het binnenste deel van de graankorrel; de meelkern. Alle overige onderdelen zoals de zemelen en de kiem worden er na het malen uit gezeefd waardoor er een wit graanpoeder overblijft. Bloem wordt gebruikt voor de bereiding van witbrood.
Volkorenmeel bevat alle onderdelen van de graankorrel, dus de meelkern, de kiem en de zemel in de natuurlijke verhouding. In volkorenmeel zijn de zemelen met het blote oog zichtbaar. Volkorenmeel wordt gebruikt bij de bereiding van volkorenbrood.
Meel is een combinatie van bloem en volkorenmeel. In meel zijn de zemelen met het blote oog zichtbaar. Meel wordt gebruikt bij het bereiden van bruinbrood.

Volkoren is belangrijk in ons eten omdat de graankorrel van nature waardevolle *voedings*stoffen (stoffen die iets goed doen voor je lichaam en helpen jou gezond te blijven) bevat.
Dit komt omdat de graankorrel eigenlijk het zaadje is waaruit een nieuwe plant kan groeien. Dit zaadje heeft alle stoffen in zich die nodig zijn voor het jonge plantje. Deze stoffen zijn ook goed voor de mens. Dan snap jij nu dat het slim is om de hele graankorrel te eten!

Voor volkorenbrood wordt de hele tarwekorrel gebruikt, in wit en bruinbrood niet. Volkorenbrood heeft de meeste voedingsvezels, vitamines en mineralen. De definitie voor volkoren staat vastgelegd in de warenwet: de hele korrel moet verwerkt zijn om het volkoren te mogen noemen. Je weet dus zeker dat het dan echt volkorenbrood is.

- 1 snee volkorenbrood bevat gemiddeld 2,3 gram voedingsvezels
- witbrood bevat gemiddeld 0,8 gram voedingsvezels per snee
- bruinbrood bevat gemiddeld 1,8 gram voedingsvezels per snee.

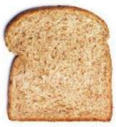

Meergranen brood is niet altijd volkorenbrood. Dit brood kan van volkorenmeel gemaakt zijn, maar ook van bloem voor wit brood. In dat geval zal het brood minder voedingsstoffen en dus ook minder vezels bevatten dan volkorenbrood.

Moutmeel is gemaakt van gekiemde tarwe- en gerstekorrels. Deze granen laat men kiemen in een vochtige omgeving. Tijdens het kiemen wordt het zetmeel omgezet in maltose. Maltose is een suiker en zorgt voor een zoete smaak, maar het zorgt er ook voor dat het brood beter kan rijzen en dat het mals blijft. Daarom voegt men soms mout toe aan brood. Resultaat: moutmeel, dat brood op een natuurlijke manier een donkere kleur en een lichtzoete smaak geeft.

Spelt is een oud tarwe-ras. Spelt levert een eiwitrijk meel met een hoog glutengehalte. Dit hoge gehalte aan gluten zorgt ervoor, dat spelt niet geschikt is voor mensen met coeliakie (gluten-intolerantie), maar wel bijzonder geschikt is voor mensen met een tarwe allergie.

Nu je dit allemaal weet over brood, welke broodsoort kies je en ga je vaak eten?

BROOD, DAAR ZIT WAT IN

Extra consult: Zomer en ijsjes

Doel:

Het kind/jongere is zich bewust van de hoeveelheid suikers en vetten in de verschillende ijssoorten.

Het kind/jongere kan een keuze maken wanneer het een ijsje in een snackmoment plant.

Werkwijze:

Je begint dit consult met de vraag of ze vaker ijsjes eten omdat het nu warmer weer is.

Je gaat hier met het kind/jongere gelijk het werkblad bespreken.

In de tabel over de calorieën per ijsje (werkblad) leg je het kind/jongere uit dat hij /zij de volgende ijsjes als klein snackmoment mag eten: raket, sorbetijs (1 bolletje), peren ijsje en de split.

De andere ijsjes in de tabel kunnen gegeten worden tijdens een groot snackmoment.

Let op; de drie onderste ijsjes bevatten enorm veel suikers én vetten. Stimuleer het kind/jongere deze ijsjes zoveel mogelijk te laten staan.

In het consult leg je het werkblad **Vraag en antwoord** uit.

Samen met het kind/jongere vul je het werkblad in. De uitwerking en antwoorden staan hieronder beschreven:
Weet jij in welk ijsje de meeste suikers zitten? En zitten er ook veel vetten in ijs?
Antwoord: Dat is het hoorntje met 3 bolletjes. Ja er zitten veel vetten in ijs. Bijvoorbeeld door chocolade maar ook omdat ijs van room gemaakt is.

Wat is een slimme oplossing om toch een ijsje te kunnen eten, naast je snackmoment?

Antwoord: Maak zelf ijsje van gepureerd fruit of limonade zonder suiker en vries dit in.

Kijk vooruit! Wanneer neem jij je ijsje (snackmoment)? Wat helpt om niet elke dag een ijsje te eten?

Antwoord: Hier maakt het kind/jongere afspraken met zichzelf over wanneer het een ijsje gaat eten. Door afspraken te maken weet het kind/jongere wanneer het wel een ijsje kan nemen zonder zich schuldig te voelen.

IJSJES!

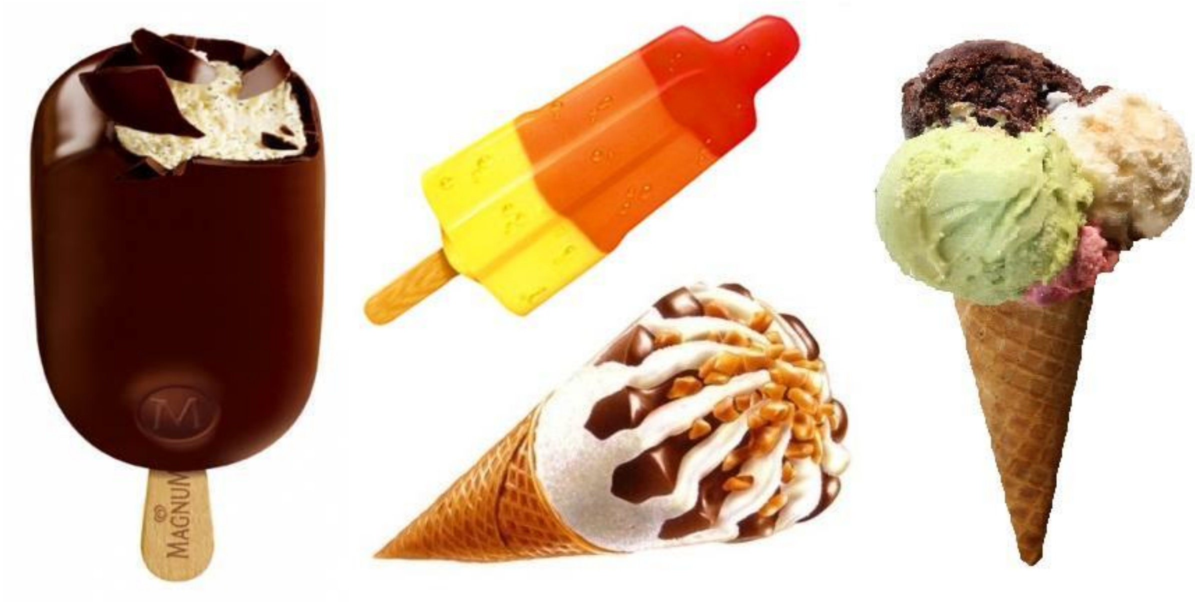

Weet jij in welk ijsje de meeste suikers zitten? En zitten er ook veel vetten in ijs?

Wat is een slimme oplossing om toch een ijsje te kunnen eten, naast je snackmoment?

Kijk vooruit! Wanneer neem jij je ijsje (snackmoment)? Wat helpt om niet elke dag een ijsje te eten?

Recept voor zelf ijsjes maken:
Fruit naar smaak
Pureren met de staafmixer
Verdelen over de ijshouders
Invriezen
Smullen maar!!!!!

Tabel Calorieën ijsjes

Ijsje	Calorieën (kcal)	Suiker (g)	Verzadigd vet (g)
Raket	40	9	0
Sorbetijs (1 bolletje)	45	10	0
Perenijsje	50	10	<0,5
Split	65	11	1,5
Solero	94	17	1
Calippo	100	20	<0,5
Schepijs (1 bolletje)	110	10	4
Vienetta (plakje)	120	10	6
Softijsje	155	13	5
Cornetto	190	14	8
Magnum	260	23	12
Sundae caramel	285	49	4
Mc. Flurry	375	49	10

Je ziet het ene soort ijs is de andere niet!
Een Mc Flurry bevat bijna 10x zoveel calorieën als een raketje.
Een coupe met ijs zit nog hoger dan een Mc Flurry (natuurlijk afhankelijk van de grootte van de coupe).
Een raketje of ander waterijsje op een snack-moment is dus geen enkel probleem.
Vergelijk bijvoorbeeld een raketje met een klein glas frisdrank, deze bevat 88kcal en 22g suiker. Of met een biscuitje, die bevat 60kcal, 8g suiker en 1,5g verzadigd vet.

Frambozen yoghurt ijslolly's

Ingrediënten (voor 6-8 ijsjes):

400 gram dikke yoghurt (bijvoorbeeld uitgelekte yoghurt of kwark)

350 gram frambozen. Roer voorzichtig de frambozen er doorheen voor als je mooie hele frambozen in je ijsje wilt zien zitten. Als je de frambozen pureert dan krijg je roze ijsjes.

Verdeel de yoghurt over de ijsvormpjes

Zet ze minimaal 6 uur in de vriezer.

Extra consult: Sporten en bewegen; uitleg MET-waarde

Doel
Het kind/jongere en ouder/verzorger heeft kennis van de MET-waarde en kan dit toepassen in eigen situatie.

Werkwijze
Dit extra consult bij sport en bewegen is voornamelijk informatief en verdiepend. De MET-waarde (Metabolic Equivalent of Task) is een meeteenheid voor de hoeveelheid energie die een bepaalde fysieke inspanning kost, vergeleken met de hoeveelheid benodigde energie in rust. De MET-waarde wordt uitgedrukt als de hoeveelheid verbrande kilocalorieën, per lichaamsgewicht in kilo per aantal uur.
Wanneer het kind/jongere en de ouder/verzorger interesse hebben over de MET-waarde, kun je hierover uitleg geven. Het is interessant om te laten zien hoeveel energie verbruikt wordt met de verschillende soorten inspanning. Tevens kun je de informatie over de MET-waarde gebruiken om het kind/jongere en de ouder/verzorger extra inzicht te geven over het belang van bewegen. Bron:
http://www.voedingscentrum.nl/encyclopedie/bewegen.aspx

Hoe lang moet je sporten om voeding te verbranden?

Gewicht<br><br>70 kilo	Milka chocolade 1 reep (100 g)	Friet met mayonaise	Pizza	Zak chips (200 g)
**Hond uitlaten**	2:30 uur	5:12 uur	3:54 uur	5:00 uur
**Rustig wandelen**	2:36 uur	5:24 uur	4:06 uur	5:06 uur
**Voetballen**	1:06 uur	2:24 uur	1:48 uur	2:18 uur
**Hard zwemmen**	0:42 uur	1:24 uur	1:00 uur	1:18 uur
**Paardrijden**	1:54 uur	3:54 uur	2:54 uur	3:42 uur
90 kilo				
**Hond uitlaten**	2:00 uur	4:06 uur	3:00 uur	3:54 uur
**Rustig wandelen**	2:00 uur	4:12 uur	3:06 uur	4:00 uur
**Voetballen**	0:54 uur	1:54 uur	1:24 uur	1:48 uur
**Hard zwemmen**	0:30 uur	1:06 uur	0:48 uur	1:00 uur
**Paardrijden**	1:30 uur	3:06 uur	2:18 uur	2:54 uur

Berekend met behulp van de MET-waarden van het voedingscentrum (zie tabel hieronder).

Activiteit	MET-waarde 4-17 jaar	MET-waarde 18 en ouder
**Hond uitlaten**	3	3
**Rustig wandelen**	2,9	3,5
**Voetballen**	6,6	7
**Hard zwemmen**	11,6	9,8
**Paardrijden**	4	5,5

Extra consult: Pasen

Doel:
Het kind/jongere is zich bewust van de extra lekkernijen die de Pasen met zich meebrengt en weet hiermee om te gaan.

Werkwijze:
Rond Pasen wordt er bij veel gezinnen extra chocolade gegeten Dit brengt uitdagingen met zich mee voor het kind/jongere. Je maakt het kind/jongere hiervan bewust. Je vraagt het kind/jongere wat het gaat doen met de paasdagen. Je anticipeert op het antwoord van het kind/jongere en gebruikt de kennis van consult 6.

De volgende adviezen neem je door met het kind/jongere:
-Voel dat je genoeg hebt gegeten!!
-Eet niet om het eten maar alleen wat jij ***echt*** lekker vindt.
-Spreek af hoeveel chocolade eitjes je eet op je snack-moment.
-Rust goed uit.
-Eet op de andere dagen volgens je schema.
-Drink voldoende water of thee zonder suiker.
-Beweeg elke dag!! Ook als het regent!
-En de allerlaatste maar wel de belangrijkste…**Geniet!**

Vertel het kind/jongere dat er ook recepten staan met minder calorieën als idee voor de paasbrunch.

In het consult leg je het werkblad **Vraag en antwoord** uit.
Antwoord: Samen met het kind/jongere vul je het werkblad in. De uitwerking en antwoorden staan hieronder beschreven:

Wanneer is jouw Choco-Ei moment?
Antwoord: Je gaat met het kind/jongere vooruitkijken en afspreken wanneer dit is.

PASEN

Pasen brengt net als de kerst, het suikerfeest of sinterklaas een heleboel lekkernijen met zich mee!
Vooral de chocolade-eitjes zijn een grote verleiding….

Neem een eitje op een afgesproken snack-momentje. Voor de paasdagen of paasvakantie bedenk je van tevoren hoe je het aan gaat pakken.

Je weet wel…. De Vooruitblik!

Wanneer ga jij jouw Choco-Ei moment plannen?

Een eitje van de kip bevat ongeveer 75 kcal. Je mag er bij het Paas-ontbijt gerust 1 of 2 van!

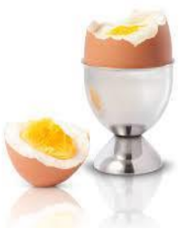

Chocolade eitjes zijn zo verleidelijk dat bijna iedereen er wel meer dan 1 lust.
Chocolade eitjes bevatten wel 1400kcal per 250gram!!!!
Het zijn echte caloriebommetjes……

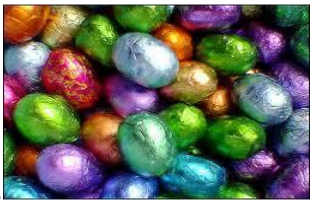

Van 1 eitje proeven komt toch echt vaak meerdere eitjes proeven…

Kleine paaseitjes (8 gram/stuk) Paaseitje witte chocolade - 45 kcal
Paaseitje melkchocolade - 45 kcal
Paaseitje pure chocolade - 40 kcal
Paaseitje gevuld (lichte vulling, likeur) - 37 kcal
Paaseitje gevuld (zware vulling, noot/praline) - 52 kcal

Grote paaseieren (50 gram/stuk)
Paaseieren witte chocolade - 270 kcal
Paaseieren melkchocolade - 270 kcal
Paaseieren pure chocolade - 260 kcal
Paaseieren gevuld (lichte vulling, likeur) - 232 kcal
Paaseieren gevuld (zware vulling, noot/praline) - 325 kcal

Paasfiguren en diverse
Middelgroot figuur melkchocolade (150 gram) - 800 kcal
Groot figuur melkchocolade (200 gram) - 1100 kcal

Paasbrood
Palmpaashaantjes (50 gram) - 148 kcal
Paasstol (1 snee, 55 gram) - 167 kcal

Eieren
Ei gebakken (1 stuk, 50 gram) - 110 kcal
Ei gekookt (1 stuk, 50 gram) - 75 kcal
Eierdooier gekookt (1 stuk, 15 gram) - 55 kcal

Paas tips op een rijtje
Voel dat je genoeg hebt gegeten!!
Eet niet om het eten maar alleen wat jij ***echt*** lekker vindt.
Spreek af hoeveel chocolade eitjes je eet op je snack-moment.
Rust goed uit.
Eet op de andere dagen volgens je schema.
Drink voldoende water of thee zonder suiker.
Beweeg elke dag!! Ook als het regent!
En de allerlaatste maar wel de belangrijkste…**Geniet!!!!**

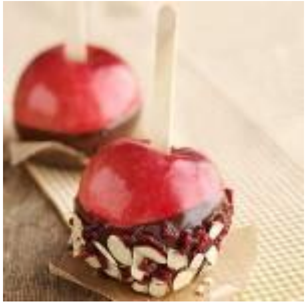

Gezonde chocolade tip voor Pasen

In stukjes 70% pure chocolade die gesmolten worden in een pannetje op laag vuur en een schepje Stevia erdoor is het ook leuk om een appel te dopen. Voor de chocola hard wordt kun je de appel in amandelsnippertjes en stukjes cranberry dippen.

Pure chocola, zonder suiker maar met de heerlijk zoete stevia om een gezonde appel.

Tips voor de slanke Paasbrunch:
Koop een pak met luxe mini-bolletjes, deze kun je beleggen met de volgende belegsoorten:
- Hüttenkäse met garnalen en dille: Per stuk 116 calorieën
- Omelet met tuinkruiden: (2 eieren voor 4 broodjes) Per stuk 142 calorieën
- Light roomkaas met aardbeien: Per stuk 118 calorieën
- Light paté met rucola: Per stuk 122 calorieën

Gouden-kiwi smoothie: per glas 105 kcal
Ingrediënten voor 4 personen:
200 gram yoghurt
wat stevia, honing of kokosbloesemsuiker naar smaak toevoegen
½ banaan
4 'gouden' kiwi's

1. Schil en pel het fruit en snijd het in kleine stukjes. Doe de stukjes in de blender.
2. Doe er zoveel yoghurt bij tot het fruit erin is verdwenen.
3. Voeg de stevia (of andere keuze) toe en blender het geheel 30 seconden.

Kortom lekkere ideetjes voor een slanke Paasbrunch…

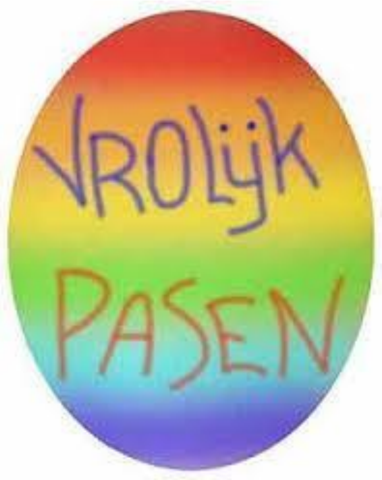

EET SMAKELIJK!!!!!

Extra consult: Sinterklaas

Doel:
Het kind/jongere is zich bewust van de extra lekkernijen die de sinterklaas periode met zich meebrengt en weet hiermee om te gaan.
Het kind motiveer je meer te bewegen door middel van pieten spelletjes.
De jongere maak je bewust van het belang van extra bewegen tijdens de sinterklaas periode.

Werkwijze:
Je bespreekt met het kind/jongere dat er in de periode voor, tijdens en na het sinterklaasfeest veel zoetigheid wordt aangeboden. Het is belangrijk dat het kind/jongere leert hier “Nee” tegen te zeggen. Zie consult 6.
In deze periode is bewegen van groot belang omdat je ervan uit kunt gaan dat het kind/jongere meer zoetigheid binnen krijgt. Door veel te bewegen compenseer je de inname van zoetigheid in deze periode.
Het jongere kind stimuleer je om de pieten spelletjes te doen zoals uitgelegd op het werkblad. Deze spelletjes kan het kind eventueel meenemen naar school en aan de juf/meester voorleggen om te gaan doen met de klas.
De jongere laat je zelf bedenken wat het kan gaan doen om meer te gaan bewegen. Je maakt samen een planning hiervoor.

In het consult leg je het werkblad **Vraag en antwoord** uit.
Samen met het kind/jongere vul je het werkblad in. De uitwerking en antwoorden staan hieronder beschreven:

Schrijf hieronder op hoe jij al het lekkers van Sinterklaas gaat weerstaan.
Antwoord: Door een planning te maken wanneer je een sinterklaas snack momentje neemt. Door een planning te maken wanneer je gaat bewegen. Hoe je bewust “nee” gaat zeggen tegen sinterklaas verleidingen.

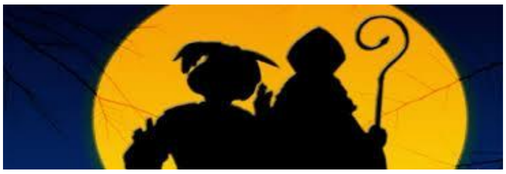

SINTERKLAAS en PIET zijn weer in ons land!!!!

Wat een feest wat een feest, wie zoet is krijgt lekkers….

Aai…. Dat is heel moeilijk! **Overal** zie je pepernoten, marsepein, chocoladeletters, gevulde speculaas. Hoe doe je dat, nu je net zo goed bezig bent met jouw gezonde leefstijl?

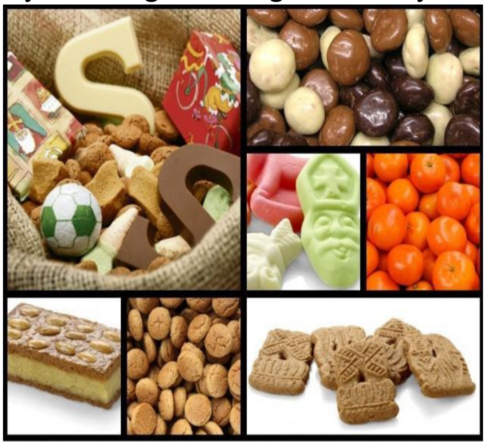

Lekkers onder de 100 calorieën:

1. 10 chocoladepepernoten (100 kcal)
2. 10 pepernoten (76 kcal)
3. Speculaasje (1 stuk = 51 kcal)
4. Schuimpjes (1 stuk = 10 kcal)
5. Warme chocolademelk (Optimel, 200 ml = 86 kcal)
6. Marsepein (20 gr = 94 kcal)
7. Taaitaai popje (55 kcal)
8. Gevulde speculaas (20 gr = 98 kcal)
9. Mandarijn (25 kcal)
10. Banketstaaf (20 gr = 93 kcal)

Maar deze zijn wel een beetje veel….

1 kleine Chocoladeletter: 350 kilocalorieën

1 grote Chocoladeletter: 730 kilocalorieën

Hoe ga je het inpakken? Uh…. Aanpakken

Schrijf hieronder op hoe jij al het lekkers van Sinterklaas gaat weerstaan.

PIETENSPORT!!!!

Sportspel 1

Pakjes in de schoorsteen mikken

Zet verschillende dingen neer om in te gooien: korf, bak, emmer, hoepel. Ga achter een lijn staan en probeer een pakje in een schoorsteen te gooien. Wanneer je goed gegooid hebt haal je je eigen pakje weer op en gooi je opnieuw.

Je kunt ook zelf van een kartonnen doos een schoorsteen knutselen waar je in kunt mikken.

Sportspel 2

Pakjes overgooien

Pieten moeten op de daken goed samen kunnen werken. Soms gooien ze een pakje van het ene dak naar het andere dak. Ga een eindje uit elkaar staan en gooi de pakjes over (maar met een bal lukt het ook hoor……). Máár... laat ze niet vallen!

Sportspel 3

Klimmen op de daken

Heb je een broertje of een zusje? Heb jij even geluk! Je broertje of zusje is een zak vol met kadootjes en die neem jij op je rug. YEAH… Met de zak vol kadootjes (maar eigenlijk dus je broertje of zusje) loop jij rondjes om de tafel. Hoeveel rondjes kun jij lopen?

Sportspel 4

Pakjes naar beneden mikken

Zet een doos en 10 pakjes hééééél ver bij de bank vandaan (ballen mogen ook……). Klim 1 voor 1 met 10 pakjes op de bank (schoenen uit!) en gooi de pakjes 1 voor 1 in de doos. Heb je ze alle 10 in de doos gegooid? Applausje voor jezelf !!

Sportspel 5

Pietendans

Zet een leuk muziekje op en dans een Zwarte Pietendans!

Elke ochtend om half 6
krijgen alle pieten les.
Ze doen dan ochtendgymnastiek
op de maat van de muziek.
Sinterklaas zegt: luister goed
ik zal zeggen hoe het moet:
voor en achter, laag en hoog
en maak nu een mooie boog.
Ga eens zitten en weer staan
draai eens rond en het is gedaan.

Sportspel 6

Springen op de daken

Leg een eindje uit elkaar kranten op de vloerbedekking of maak met plakband een gladde vloer. Dit zijn de daken. Spring van krant naar krant zonder "naar beneden te vallen".

Pietenspelletjes

- Hoe snel kun je fluiten na het eten van pepernoten of taaitaai?
- Hoe lang kun je op je hurken zitten met je handen omhoog?
- Wie kan het langst een pepernoot op zijn neus laten liggen? Als het lang duurt, mag degene die af is opdrachten geven, zoals "ga staan", "klim op je stoel".
- Zaklopen
- Cadeau gooien. Mik een cadeau van een afstandje in een krat.
- Verstop pepernoten. Wie vindt er de meeste?
- Beeld een sinterklaasliedje uit. Wie raadt welk liedje het is? Zing het liedje samen.
- Pepernotendans. Dans rondom een tafel waar pepernoten op liggen. Als de muziek stopt, pakt iedereen zo snel mogelijk een pepernoot (mandarijntjes kunnen ook…. …..) Wie geen pepernoot heeft, is af en mag de muziek stopzetten.
- Stop een wasknijper in je mond. Doe er een chocolade munt tussen. De ander heeft ook een wasknijper in de mond. Geef de munt door, zonder dat hij valt!

Sinterklaas kleurplaat

De Gezond en Energieke leefstijl van AMERICO!!!

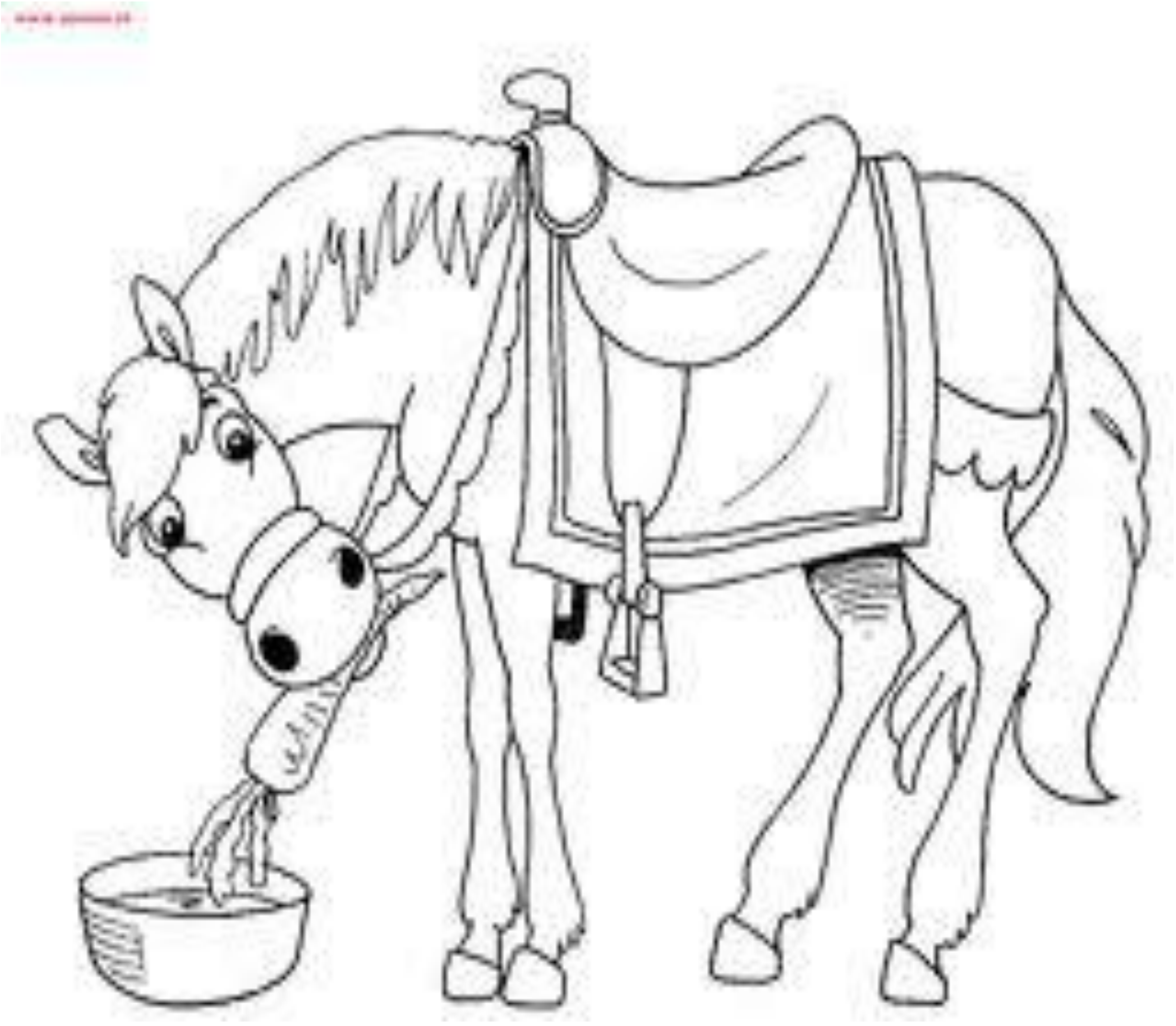

Waarom eet Americo een wortel en niet pepernoten?

..

Waarom drinkt Americo geen chocolademelk maar water?

...

Americo staat klaar om te gaan rijden waarom gaat hij niet in zijn stal liggen?

..

Jij bent net zo goed bezig als AMERICO!!!

Extra consult: Kerstmis

Doel:
Het kind/jongere is zich bewust welke uitdagingen op het gebied van voeding en beweging de periode rond Kerst en Oud & Nieuw met zich meebrengt.
Het kind/jongere kan een planning maken op het gebied van voeding en beweging van de feestdagen en de vakantie dagen.
Het kind/jongere weet hoe het zijn/haar kerstvakantie gezond en energiek door kan brengen.

Werkwijze:
Je vertelt het kind/jongere dat deze feestdagen nog meer dan andere feestdagen vooral gaan om lekker (en veel) eten. Maak het kind/jongere bewust van de uitdaging van de kerstvakantie. Deze uitdaging ligt niet alleen op het gebied van lekker en veel eten tijdens de feestdagen. Ook de periode van het jaar zorgt er vaak voor dat je veel binnen zit en daardoor minder beweegt.
Om deze dagen goed door te komen is het belangrijk dat het kind/jongere zich beseft dat het voor deze dagen afspraken met zichzelf moet gaan maken op het gebied van een gezonde leefstijl. Je gaat het kind/jongere helpen met het maken van een planning voor de feestdagen en de vakantiedagen. Hierbij gebruik je onder andere de kennis van consult 6.
Verder kan je het kind/jongere de volgende adviezen geven:
-Voel dat je genoeg hebt gegeten!!
-Eet niet om het eten maar alleen dat wat jij ***echt*** lekker vindt.
-Spreek af met jezelf hoeveel oliebollen en/of appelflappen je gaat eten.
-Rust goed uit na Oudejaarsnacht.
-Eet op de andere dagen volgens je schema.
-Neem en plan geen groot snack- moment deze weken.
-Drink voldoende water of thee zonder suiker.
-Beweeg elke dag!! Ook als het regent of sneeuwt!
-En de allerlaatste maar wel de belangrijkste…**Geniet!!!!**

In het consult leg je het werkblad **Vraag en antwoord** uit.
Samen met het kind/jongere vul je het werkblad in. De uitwerking en antwoorden staan hieronder beschreven:
Wat is er anders aan de kerstvakantie dan aan een zomervakantie?
Antwoord: Het is winter. Er zitten 3 (4) feestdagen in deze vakantie.

Hoe plan je het eten en drinken op 1ste en 2de kerstdag?
Antwoord: Je maakt samen het kind/jongere afspraken en een planning voor de feestdagen.

Wat ga je doen in de dagen vóór de kerst en in de dagen ná de kerst, in de vakantie dus?
Antwoord: Deze dagen staan in het teken van een gezonde leefstijl. Geen snack momentjes tussendoor eten of drinken en voldoende bewegen. Het is heel belangrijk dat het kind/jongere zich hier bewust van is en gemotiveerd is deze afspraken na te komen.

Hoe plan je het bewegen en eten en drinken vóór de kerst en de dagen ná de kerst, in de vakantie dus?
Antwoord: Je gebruikt de kerstvakantie kalender uit de bijlagen en vult deze samen in.

Hoe ga jij Oud en Nieuw vieren?
Antwoord: Met wie ben je die avond?
Vanaf hoe laat gaan jullie de avond vieren. Begint deze avond met het avondeten of daarna?
Blijf je de hele avond op of ga je even naar bed?

Hoeveel oliebollen of appelflappen denk jij te gaan eten?
Antwoord: Je spreekt af met het kind/jongere hoeveel oliebollen of hapjes hij/zij maximaal gaat eten die avond.

Hoe overleef ik……

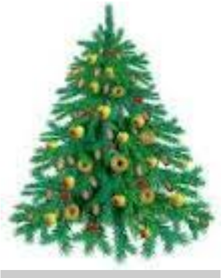

De Kerstdagen, de Kerstvakantie en Oud en Nieuw!!!

De Kerstvakantie

De kerstvakantie is een bijzondere vakantie. Het is midden in de winter, het is snel donker, soms is het koud of ligt er zelfs sneeuw en ijs. We maken onze huizen gezellig met een kerstboom en hangen overal lampjes op en steken kaarsjes aan. Nog veel meer dan andere feestdagen is eten een heel belangrijk onderwerp in deze dagen. Het is heel lastig om op deze dagen geen extra koekjes, chocolaatjes, chipjes en andere lekker dingen te nemen. Maar hoe ga je daar nou mee om als je gezond en energiek wil leven??

Neem een kopje thee (water of suikervrije limonade mag natuurlijk ook). Ga er eens lekker voor zitten en denk na over de volgende vragen. Vul samen met bijvoorbeeld je moeder, vader of vriend/vriendin de vragen in. **Zo ga je goed voorbereid en zeker van jezelf de kerstvakantie tegemoet!!**

Happy Holidays!!!!

Wat is er anders aan de kerstvakantie dan aan een zomervakantie?

De Kerstdagen…Ho ho ho…Merry Christmas!

Hoe plan je het eten en drinken op 1ste en 2de kerstdag?

Wat ga je doen in de dagen vóór de kerst en in de dagen ná de kerst, in de vakantie dus...? Hoe plan je het bewegen en eten en drinken vóór de kerst en de dagen ná de kerst, in de vakantie dus?

Bewegen

Eten en **Drinken**

Oud en Nieuw!!!!!!

Hoe ga jij Oud en Nieuw vieren?
Met wie? Vanaf hoe laat? Blijf je de hele avond op?

Hoeveel oliebollen of appelflappen denk jij te gaan eten?...........................

Kerstvakantietips op een rijtje:
Voel dat je genoeg hebt gegeten!!
Eet niet om het eten maar alleen dat wat jij ***echt*** lekker vindt.
Spreek af met jezelf hoeveel oliebollen en/of appelflappen je gaat eten.
Rust goed uit na Oudjaarsnacht.
Eet op de andere dagen volgens je schema.
Neem en plan geen groot snack- moment deze weken.
Drink voldoende water of thee zonder suiker.
Beweeg elke dag!! Ook als het regent of sneeuwt!
En de allerlaatste maar wel de belangrijkste.... **Geniet!!!!**

Mooie Kerstdagen en een Gezond en Energiek Nieuwjaar!!

GO! GEZOND ONDERWEG

Extra consult: Ramadan

Doel:
Het kind/jongere is zich bewust van het belang van een gezond eetpatroon tijdens de Ramadan tijd en kan dit toepassen.
Het kind/jongere is zich bewust van de uitdagingen die zich tijdens de ramadan kunnen voordoen op het gebied van eten, drinken en bewegen.
Het kind/jongere weet een gezond beweegpatroon tijdens de Ramadan tijd toe te passen.

Werkwijze:
Als kindergezondheidscoach ga je na of het kind/jongere meedoet aan de Ramadan. De Ramadan is de maand van inkeer, waarin tussen fajr, de dageraad ruim vóór zonsopkomst, en maghrib, bij zonsondergang, gevast wordt. Dit betekent dat er in deze periode een ander eet- drink en beweegpatroon is voor het kind/jongere.
Je bespreekt in dit consult met het kind/jongere hoe ze de Ramadan-tijd met zijn andere eet/drink- en beweegpatroon kan gaan inpassen in de geleerde doelen van het programma GO! Gezond Onderweg. (Eten, drinken, bewegen en rust zijn de belangrijke punten waar we in GO! Gezond Onderweg met het kind/jongere mee bezig zijn).
Je gaat met het kind/jongere na wat de uitdagingen zijn op dit gebied.
Dit doe je onder andere middels de vragen op het werkblad, die je samen tijdens het consult invult.
In dit consult geef je verder adviezen voor het eten, drinken en bewegen.
Adviezen:
-Eet niet te snel en daardoor te veel! Hier bespreek je het stoplicht met het kind/jongere nogmaals.
-Doorbreek het vasten door te starten met een paar dadels/gedroogde vijgen. Deze zorgen voor “gezondere” suikers dan de suikers in de baklava en is goed voor je spijsvertering. Vaak zit je na een paar happen baklava “vol” en eet je niet veel meer hierna. Hierdoor mis je andere belangrijke voedingsstoffen die je binnenkrijgt door het andere eten.
-Een lekkere gezonde oplossing bij weinig honger:
Maak een fruit shake met avocado, banaan, lijnzaad, beetje melk en dadels. Natuurlijk mag je ook ander fruit toevoegen.
-Een andere tip is om tijdens het avondeten eerst een lichte soep te eten en dan pas een glas water.
-Sporten kan het kind/jongere nu beter niet intensief doen, omdat je dan veel vocht verliest en dat kan je nu niet direct weer aanvullen. Hierdoor kan je dan hoofdpijn krijgen of je niet lekker voelen. Sport op een rustige manier. Niet gaan rennen maar bijvoorbeeld oefeningen doen die je spieren versterken, yogaoefeningen om te ontspannen. Blijf proberen om voldoende te bewegen zoals rustig wandelen en fietsen
-Neem ook overdag een extra rust momentje. Het is niet nodig om echt te slapen maar even uitrusten kan zorgen voor minder hongergevoel. De nachten zijn onrustig tijdens de Ramadan omdat je wakker gemaakt wordt om te kunnen eten en drinken voor zonsopgang. Hierdoor mis je slaap.

-Wanneer je volkorenbrood, zilvervliesrijst, couscous, groente en fruit eet zorgen de aanwezige vezels voor een “vol” gevoel na de maaltijd. Hierdoor krijg je minder snel honger. Dat is wel zo fijn voor overdag als je niet mag eten en drinken.
-Door veel verschillende gezonde dingen te eten krijg je meer verschillende voedingsstoffen binnen!
-Voordat je begint met vasten wordt er in de nacht gegeten. Eet hier zoals je normaal je ontbijt samenstelt. Bijvoorbeeld: Geroosterd volkorenbrood met komkommer/plakje kaas en thee met munt en nog een bakje yoghurt met aardbeien.
-Drink ’s morgens voor zonsopgang een paar extra glazen water!
-Drink niet te snel en soms kun je ook wat bouillon nemen als het erg warm is.
-In de zomer krijg je sneller dorst als je lange tijd niets drinkt. Het is vaak (erg) warm, hierdoor zweet je meer en raak je meer vocht kwijt. Daarom is het goed om in de tijd dat je mag eten en drinken ruim voldoende water, thee en eventueel glaasje bouillon te drinken.

In het consult leg je het werkblad **Vraag en antwoord** uit.
Samen met het kind/jongere vul je het werkblad in. De uitwerking en antwoorden staan hieronder beschreven:

Doe jij mee aan de Ramadan?
Antwoord: Zo ja; ga door met de volgende vragen.
Hoe zorg je er bijvoorbeeld voor dat je voldoende voedingsstoffen binnen krijgt?
Antwoord: Gevarieerd eten en drinken, vooral producten waar vitamines en mineralen inzitten. Eet minder vet en suikerrijke voeding om te voorkomen dat je dan te weinig gezonde producten eet.
Hoe voorkom je buikpijn?
Antwoord: Door niet te veel en te snel te eten achter elkaar.
Wat kan je doen om minder snel trek te krijgen overdag?
Antwoord: Gezonde vezelrijke producten te eten en te drinken op de momenten dat het mag.
Hoe blijf je bewegen en sporten zonder eten en drinken?
Antwoord: Door laag intensief te bewegen, bijvoorbeeld yoga, wandelen, rustig fietsen etc.
Hoe voorkom je hongergevoel door te weinig slapen?
Antwoord: Door op de momenten dat je mag eten en drinken gezonde en vezelrijke voeding te nemen. Daarnaast bijvoorbeeld in de middag een poosje slapen om je slaaptekort te verminderen.
Zou jij nog meer moeilijkheden kunnen bedenken?
Antwoord: Kind/jongere geeft hier zelf een antwoord op.
Weet je nog wat vezels zijn?
Antwoord: Zie consult 2.1
Kun jij verschillende soorten voeding opschrijven die allemaal goede stofjes voor jouw lichaam bevatten?
Antwoord: Bijvoorbeeld volkorenbrood, aardappels, zuivel, fruit en groente.
Waarom is Baklava of gefrituurde hapjes niet zo fijn voor jou om te eten?
Antwoord: Het is te calorierijk met vooral suiker en vet, hierdoor raak je snel verzadigd en kan de baklava ervoor zorgen dat je te weinig gezonde producten gaat eten.

Tips voor een goed en gezond doorkomen van de Ramadan

De Ramadan is begonnen.
Met het Suikerfeest vier je het einde van de Ramadan.

Doe jij mee aan de Ramadan?............................

Nu jij bezig bent bij GO! en je ook meedoet aan de Ramadan dan vraag je je misschien af hoe deze uitdaging aan gaat?

Hoe zorg je er bijvoorbeeld voor dat je voldoende voedingsstoffen binnen krijgt?
Hoe voorkom je buikpijn?
Wat kan je doen om minder snel trek te krijgen overdag?
Hoe blijf je bewegen en sporten zonder eten en drinken?
Hoe voorkom je hongergevoel door te weinig slapen?

Zou jij nog meer uitdagingen kunnen bedenken?

Voordat je begint met vasten wordt er in de nacht gegeten. Eet hier zoals je normaal je ontbijt samenstelt. Bijvoorbeeld: Geroosterd volkorenbrood met komkommer/plakje kaas en thee met munt en nog een bakje yoghurt met aardbeien.

Drink 's morgens voor zonsopgang een paar extra glazen water!
Drink niet te snel en soms kun je ook wat bouillon nemen als het erg warm is.

In de zomer krijg je sneller dorst als je lange tijd niets drinkt. Het is vaak (erg) warm, hierdoor zweet je meer en raak je meer vocht kwijt. Daarom is het goed om in de tijd dat je mag eten en drinken ruim voldoende water, thee en eventueel glaasje bouillon te drinken.

Eet vezelrijk om trek te verminderen!

Je hebt geleerd veel vezelrijke voeding te eten (volkoren producten, fruit, groenten).
Weet je nog wat vezels zijn?

Wanneer je volkorenbrood, zilvervliesrijst, couscous, groente en fruit eet zorgen de aanwezige vezels voor een “vol” gevoel na de maaltijd. Hierdoor krijg je minder snel honger. Dat is wel zo fijn voor overdag als je niet mag eten en drinken.

Door veel verschillende gezonde dingen te eten krijg je meer verschillende voedingsstoffen binnen!

Kun jij verschillende soorten voeding opschrijven die allemaal goede stofjes voor jouw lichaam bevatten?

Waarom is Baklava of gefrituurde hapjes niet zo fijn voor jou om te eten?

Ja! Heel goed! Omdat ze veel snelle suikers en verkeerde vetten bevatten. Hierdoor kun je last krijgen van piekjes en dipjes.

Tips voor het eten tijdens de ramadan:
Eet niet te snel en daardoor te veel!
Je hebt maar een paar uurtjes de tijd om tussen zonsondergang en zonsopgang te eten. Doorbreek het vasten door te starten met een paar dadels/gedroogde vijgen. Deze zorgen voor “gezondere” suikers dan de suikers in de baklava en is goed voor je spijsvertering. Vaak zit je na een paar happen baklava “vol” en eet je niet veel meer hierna. Hierdoor mis je andere belangrijke voedingsstoffen die je binnenkrijgt door het andere eten.

Een lekkere gezonde oplossing bij weinig honger:
Maak een fruit shake met avocado, banaan, lijnzaad, beetje melk en dadels. Natuurlijk mag je ook ander fruit toevoegen.
Een andere tip is om tijdens het avondeten eerst een lichte soep te eten en dan pas een glas water.

Ga niet haasten om te eten.
Ken je het stoplicht nog?
Neem de tijd om rustig te eten. Voel dat je genoeg gehad hebt.
Als je dan kleine hapjes eet en goed kauwt kan je lijf weer aan het eten wennen en krijg je minder snel buikpijn. En dat is wel fijn toch?

Beweging, hoe zit het daar mee?
Sporten kan je nu beter niet intensief doen, omdat je dan veel vocht verliest en dat kan je niet direct weer aanvullen. Je kan dan hoofdpijn krijgen of je niet lekker voelen. Sport op een rustige manier. Niet gaan rennen maar bijvoorbeeld oefeningen doen die je spieren versterken, yogaoefeningen om te ontspannen. Blijf proberen om voldoende te bewegen zoals rustig wandelen en fietsen

Neem ook overdag een extra rust momentje. Het is niet nodig om echt te slapen maar even uitrusten kan zorgen voor minder hongergevoel. De nachten zijn onrustig tijdens de Ramadan omdat je wakker gemaakt wordt om te kunnen eten en drinken voor zonsopgang. Hierdoor mis je slaap.
Dan is het einde van de Ramadan, je hebt het volgehouden en je bent trots! Samen met al jouw familie vier je dat jullie een maand van bezinning gehad hebben.
Nu is het feest, het Suikerfeest!
Natuurlijk doe jij mee aan dit feest!
Wanneer jij die ene dag meedoet is er niets aan de hand……
Vergeet vooral niet om ervan te genieten!
Na het Suikerfeest ga je weer verder met jouw gezonde en energieke leefstijl.
Je kiest weer uit wanneer je je snackmomenten hebt, je pakt je ritme van eten en drinken weer op. Ook het sporten kun je nu weer lekker intensief gaan doen. Is het lastig om weer in het ritme te komen? Mail, sms of bel dan jouw coach!

Succes en zet hem op!

Gezond recept Harira.

In het harira recept zitten veel snelle koolhydraten. Dit is mede door de bloem en de vermicelli die erin zit. Bloem wordt gebruikt om de soep te binden, dit kun je vervangen door extra verse groente toe te voegen. Hierdoor maak je de soep extra lekker....

Wat heb je nodig?

- Scheutje olijfolie
- 1 el Keltisch zeezout (ook beetje op gevoel)
- 500 gram mager lamsvlees (mag ook runder)
- 2 uien
- 1 bleekselderij
- 4 grote tomaten
- 4 grote wortels
- 200 gram tomatenblokjes (voorkeur naar biologisch)
- 200 gram kikkererwten
- 200 gram linzen
- Klein bosje koriander
- 1 tl-gemberpoeder
- 1 tl-paprikapoeder
- 1 tl zwarte peper (gemalen)
- 1 tl-kurkumapoeder

Bereidingswijze

1. Snij het vlees in blokjes
2. Bak het vlees in de olijfolie en laat dit even gaar bakken.
3. Doe de ui, bleekselderij en wortels in een blender en blend dit heel fijn en bak dit mee met het vlees.
4. Doe dit hetzelfde met de tomaten en voeg ook de tomaatblokjes toe.
5. Na ongeveer 10 minuten voeg je de koriander toe samen met alle kruiden en keltisch zeezout. Kook ondertussen 1.5 liter water.
6. Giet het inmiddels gekookte water erbij, en laat dit goed sudderen voor een klein uurtje
7. Blijf de soep wel in de gaten houden door af en toe te roeren.
8. Na een uurtje voeg je de linzen en kikkererwten bij, dit laat je nog eens 15 tot 20 minuten meekoken.

Extra consult: Vakantie kalender

Doel:
Het kind/jongere voor uit laten denken en een planning laten maken.
Het kind/jongere inzicht geven in zijn/haar vakantie met alle verleidingen.

Werkwijze:
Je pakt de kalender van de juiste vakantie erbij. (Er zijn kalenders voor de zomer, herfst, kerst en voorjaarsvakantie. Tevens is er een blanco, zelf in te vullen kalender).
Je kijkt samen met het kind/jongere naar de lengte van de vakantie. Je vult samen in welke bijzonderheden het kind al weet te gaan doen in de vakantie.
Bij de kerstvakantie vul je de feestdagen in.
Je maakt afspraken met het kind/jongere wanneer er snack momenten zijn. Ook vul je in wanneer en hoeveel er bewogen kan worden. Je denkt mee met mogelijkheden tot bewegen en oplossingen voor eventuele voorziene moeilijkheden in de keuze van voeding en vocht.

HERFSTKLEURPLAAT

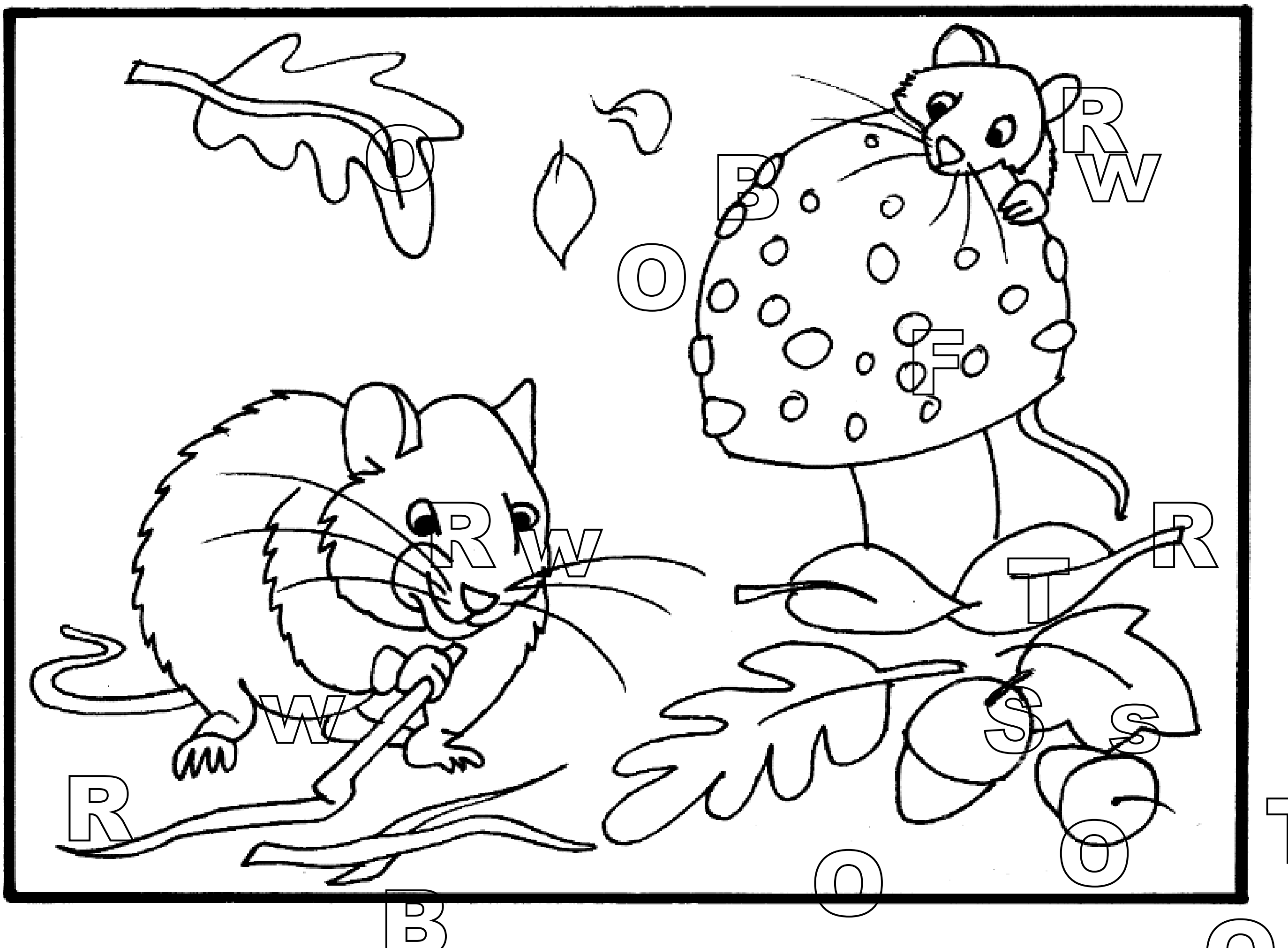

paintingfun.nl

In elke zin wordt een woord met een kleur aangegeven. Zoek de letter in de kleurplaat en kleur in!

W = Drink elke dag 1,5 liter, het liefst Water of thee zonder suiker.

O= Eet elke dag voor je naar school gaat een **ontbijt.** Een ontbijt geeft energie om de dag te starten en zorgt ervoor dat je lichaam goed wakker wordt.

R = Ga op tijd slapen (zonder je telefoon!) zodat je genoeg Rust krijgt en energie hebt voor de nieuwe dag.

F = **Fruit en Groente**, dat mag altijd!

B = Elke dag een uurtje Bewegen en twee a drie keer in de week Sporten.

S = *Voel* dat je genoeg hebt gegeten en stop dan! Dit is je Stoplicht, het verzadigingsgevoel.

T = Suikers en verkeerde vetten, zitten in veel Tussendoortjes. Eet ze daarom niet elke dag. Kies uit wanneer je iets gaat snacken en GENIET ervan! Op andere momenten eet je groenten of fruit.

(Voorbeeld: W = grijs, O=bruin,..........)

Bedacht door Sari, 8 jaar.

Extra consult: Beloning

Doel:
Het kind motiveren en stimuleren om zijn/haar doel of afspraak te behalen.

Werkwijze:
Je zet deze beloningskaart in wanneer je merkt dat het kind afspraken of doelen niet nakomt of behaalt. De reden hiervoor kan zijn dat de motivatie hiervoor van het kind niet voldoende is, of een steuntje in de rug nodig heeft. De ervaring leert dat kinderen een hogere motivatie krijgen om met een afspraak of doel bezig te zijn wanneer er een beloning tegenover staat. De beloningskaart kan je op een laagdrempelige manier inzetten.
Je kunt als kindergezondheidscoach een velletje stickers meegeven aan het kind en de ouder/verzorger. Het kind kan in totaal 5 stickers sparen en plakken op zijn beloningskaart. Je bespreekt met de ouder/verzorger welke beloning het kind krijgt als zij/hij de beloningskaart vol heeft. Voorbeelden van een beloning zijn een klein kadootje of samen een spelletje spelen. De beloning mag geen voeding of drank zijn.

Beloningskaart

Naam:________________________________Datum:__________________________

Ik krijg een beloning als ik .. keer heb laten zien dat ik:

____

____

____

____

____

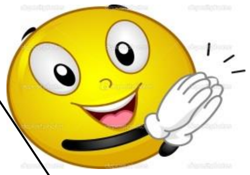

1

2

3

4

5

Mijn beloning

____

____

____

Literatuurlijst

-Brug, J., Schaalma, H., Kok, G., Meerstens, R.M., van der Molen, H.T. (2000). *Gezondheidsvoorlichting en gedragsverandering, een planmatige aanpak.* Van Gorcum & Comp. B.V. Assen.
-Dr. Deckers-Kocken, J.M. (2014). *Kinderbuik.*
-Mill van, E., Struik, A., (2015) *Overgewicht en obesitas bij kinderen. (Boom).*
-Breat, C., Mels, S., Joossens, L., Swets, E., (2010). *Kinderen en jongeren met overgewicht. Werkboek voor kinderen.* Garant
-Breat, C., Tanghe, A., Swets, E., (2010). *Kinderen en jongeren met overgewicht. Werkboek voor adolescenten.* Garant.
-Breat, C., Mels, S., Joossens, L., Swets, E., Tanghe, A., (2010). *Kinderen en jongeren met overgewicht. Werkboek voor ouders.* Garant.
-Breat, C., Mels, S., Joossens, L., Swets, E., Moens, E., Tanghe, A., (2010). *Kinderen en jongeren met overgewicht. Handleiding voor begeleiders.* Garant.
-Breat, C., (2010). *Kinderen met overgewicht.* Hogrefe.
-Vroemen, S., (2016). *Eten voor je leven.* Akasha.
-Dewulf, D., (2010). *Mindful Gelukkig*. Lannoo.
-Seidell, J., Halberstadt, J., (2018). *Jongleren met voeding.* Atlas contact.
-Kortink, J., Noordenbos, G., (2011). *Uit de Ban van emotie-eten*. Servire
-Heyster, H., Verheijen, H., (1994). *Psychologie voor kinderverpleegkundigen*. Lemma
-Majoor, P.W.F.M., Leyten, M., Poorthuis-Bisschop, A.A.M, Hiemstra, I. (1993). *Kinderverpleegkunde*. Elsevier/Bunge.
-Prof.Dr. Hirasing, R., Gouwerok, M. (2007). *Kinderen met overgewicht.* Rean.
- Talma, H., Schonbeck, Y., Bakker, B., Hirasing, R.A., Buuren van S. (2011) *Groeidiagrammen 2010.* TNO Inovation for life.
-PON-partnerschap overgewicht nederland. (2010). *Zorgstandaard Obesitas*.
-Seidell, J., Halberstadt, J. (2018), *Jongleren met voeding*. Atlas contact.
- Hoenderdos, K. (2011). *Puur gezond. Slank in 8 stappen*. Thoeris.
-Kharbouch, N., Kharbouch, R. (2017). *Healthy Sisters.* Xander.
-Haisma, I. *Met verstand gezond.* MAM.
-Steenhuis, I., Poelman, M., Overtoom, W.(2016). *Smart Size Me*. Scriptum.
-Furman, B., (2004). *Kids'Skills.* Boom/Nelissen.
-Beumer-peeters, C. (2010). *Mission Possible*. Boom/Nelissen.
-Wolters, C. (2011). *Oplossingsgericht aan het werk met kinderen en jongeren.* Pica.
-Diekstra, R.F.W. (2012). *Ik kan denken/voelen wat ik wil.* Pearson.
-Houweling, B., Kleefstra, N., Berk, K. (2016). *Etenstrijd*. Langerhaus.
-Tol, M., Zerouali, N.(2012). *Arabia bij je thuis*. Kosmos.
Torenvliet, M. (2017). *Herken je honger*. S2uitgevers.
-Drs. Leth de, R., Dorenbos, J. (2013). *OERsterk koken.* de Leth.
-Bakker-Dam van, M., Witteman, L. (2010). *Voedingspiramide*. Colofon.
-Steenhuis, I., Overtoom, W. (2015). *Terugval en verleiding*. SWP.
-Berkenpas, M., Herweijer, G., Klamer, J., Smit, L. (2016). *Eet als een expert*. Kosmos.
-Hoenderbos, K., Pijl, H. (2016). *Diabetes type 2?*. Fontaine.

Materialen ter ondersteuning van consulten

Er zijn veel materialen ontwikkeld over een gezonde leefstijl. Denk hierbij aan folders, infographics of posters. Tijdens je werkzaamheden als kindergezondheidscoach kan het van meerwaarde zijn om deze, vaak beeldende, materialen in te zetten tijdens het geven van een consult. Je gebruikt als kindergezondheidscoach alleen onderbouwde materialen die ontwikkeld zijn met kennis en eventueel wetenschap.
Deze materialen zijn meestal gratis te verkrijgen. Je schaft de materialen als kindergezondheidscoach zelf aan. Wanneer er vragen hierover zijn kun je terecht bij de senior kindergezondheidscoach.

De volgende materialen adviseren wij hiervoor:
Folders, kaarten en een blok beweegkaarten ontwikkeld door Friesland campina. Via www.frieslandcampina.com
Map voor de jeugdverpleegkundige van het voedingscentrum. Via www.voedingscentrum.nl

Daarnaast zijn de volgende materialen toepasbaar tijdens de consulten:

- Kneedgum ter verduidelijking hoe het BMI veranderd ten opzichte van gewicht en lengte.
- Een paar vliespinda's ter verduidelijking van de uitleg van vezels.
- Een set kaarten met gezonde en minder gezonde producten en activiteiten. Dit is om te kijken wat het kind/jongere al weet. Daarnaast breng je hiermee een spelelement in het consult.
- Een set kaarten met een gedachte of spreuk. Je gebruikt deze om tot een dieper gesprek te komen of ter positieve ondersteuning. Bijvoorbeeld: Helpende gedachten van Adinda de Vreede. Story cubes, www.storycubes.com. Praatprikkels van www.filosofiejuf.nl. Voor jezelf als coach kan ondersteunend zijn de hulpwaaier coachen van kinderen, van Corry Wolters. Ook de waaier van Tea Adema, oplossingsgericht aanpakken is een tip.
- Bloknoot met kleurplaten en een set kleurpotloden of stiften (uitwasbaar) voor het jonge kind. Zie ook inventarisatielijst
- Stickervelletjes ter beloning (beloningskaart), als compliment of motivatie. Zie ook inventarisatielijst.

Bijlage 1 Stroomschema voor verwijzing en ketensamenwerking, MDO en Bloeddruktabel

Stroomdiagram voor verwijzing en ketensamenwerking.

Zie de dropbox van jouw kern voor het verwijsschema.

Stroomschema verwijzing naar MDO

Zie de dropbox van jouw kern voor het protocol en de werkwijze van een MDO.

Bloeddruktabel voor kinderen

leeftijd (jaren)	systole (mmHg)	diastole (mmHg)	leeftijd (jaren)	systole (mmHg)	diastole (mmHg)
**5**	112	72	**12**	123	81
**6**	114	74	**13**	126	81
**7**	115	76	**14**	128	82
**8**	116	78	**15**	131	83
**9**	118	79	**16**	132	84
**10**	119	79	**17**	135	85
**11**	121	80	**18**	135	85

Je meet de bloeddruk bij de intake. Wanneer de bloeddruk afwijkt van de aangegeven waarde in de tabel hierboven dan geef je dit door aan kinderarts, jeugdarts of huisarts.

Bijlage 2 Gewichtsverloopkaart jongens

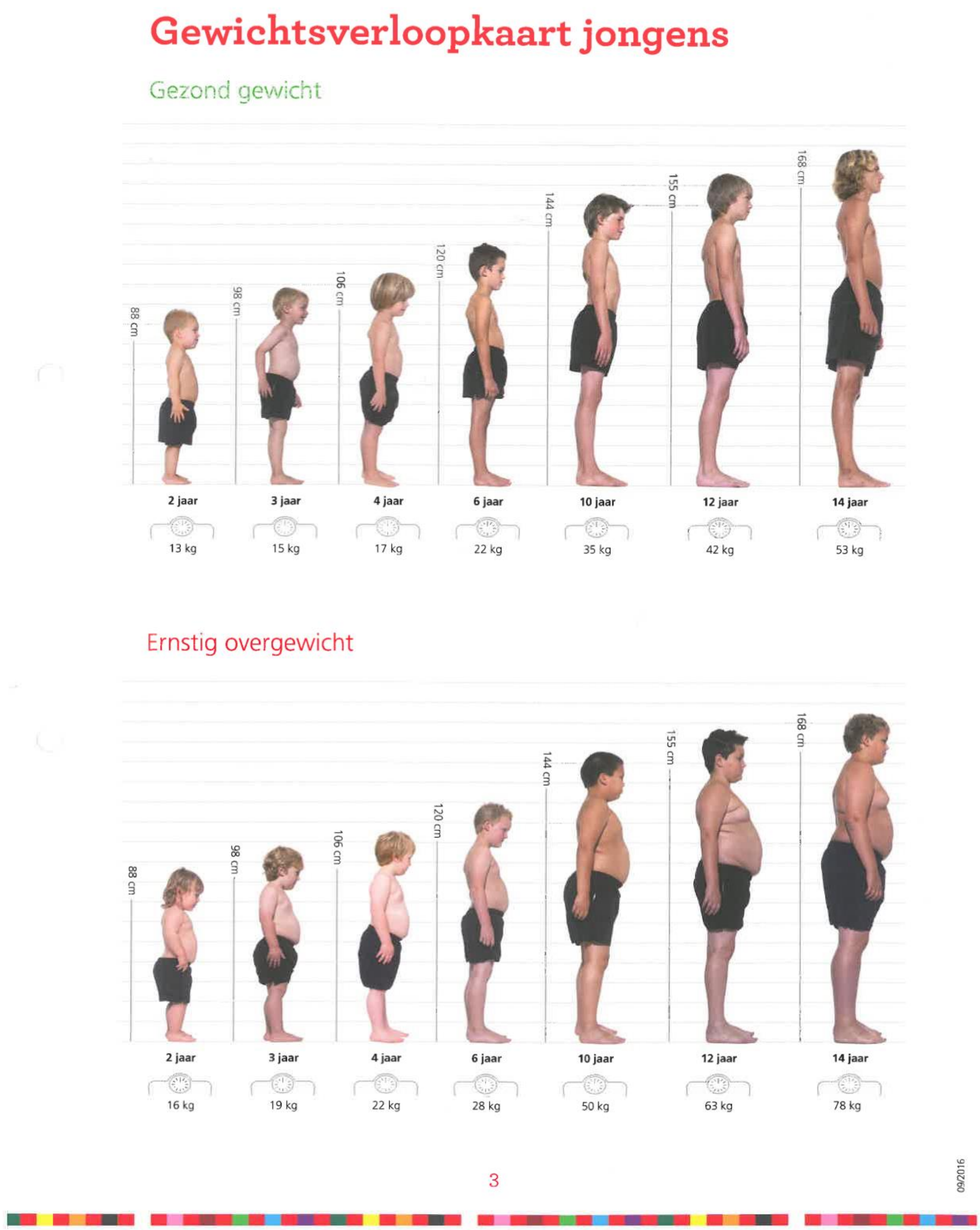

Bron: Voedingscentrum

Bijlage 3 Gewichtsverloopkaart meisjes

Gewichtsverloopkaart meisjes

Gezond gewicht

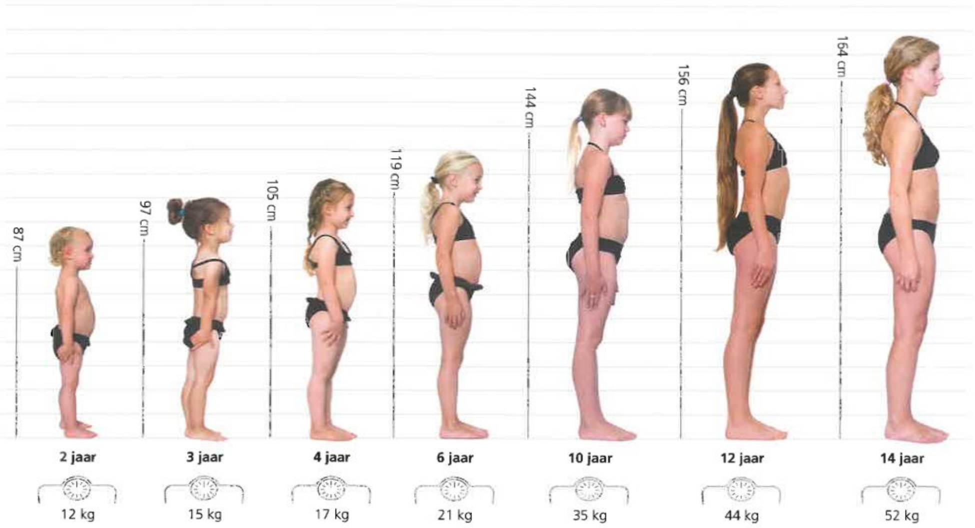

Ernstig overgewicht

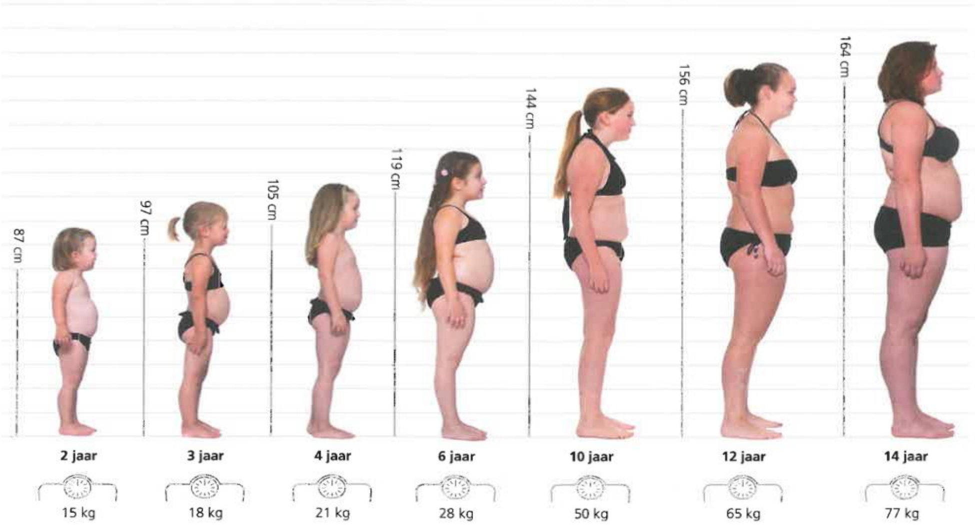

4

09/2016

Bron: Voedingscentrum

Bijlage 4 Aanbevolen portiegroottes voor een kind ontbijt

Aanbevolen portiegroottes voor een kind

Ontbijt

Kind 1 - 3 jaar
- 1 belegde boterham
- glas melk (150 ml)

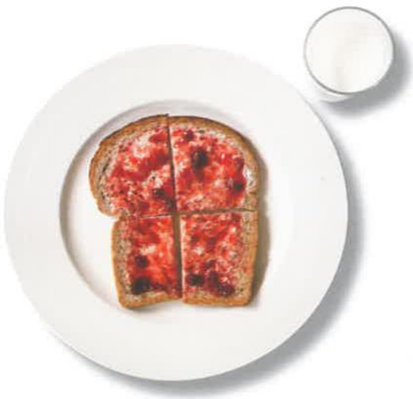

Kind 4 - 8 jaar
- 1-2 belegde boterhammen
- glas melk (150 ml)

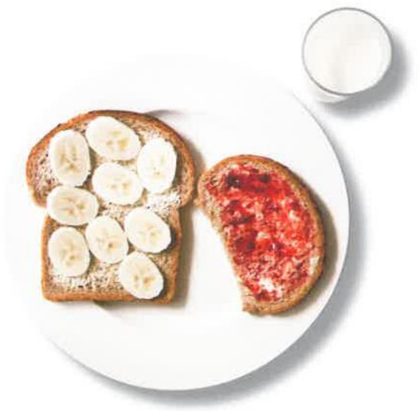

Kind 1 - 3 jaar
- 1 puntje bruin Turks brood (30 gram) met olijfolie
- ½ gekookt ei
- 2 zwarte olijven*
- glas lauwe lichte thee

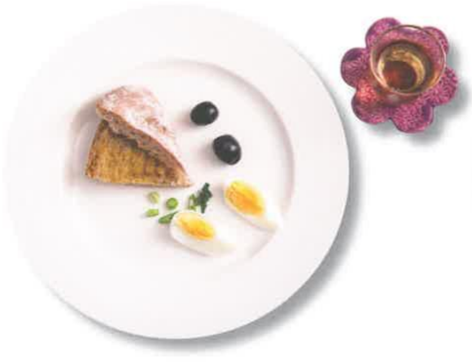

Kind 4 - 8 jaar
- 2 puntjes bruin Turks brood (60 gram) met olijfolie
- 1 gekookt ei
- 3 zwarte olijven*
- glas lauwe lichte thee

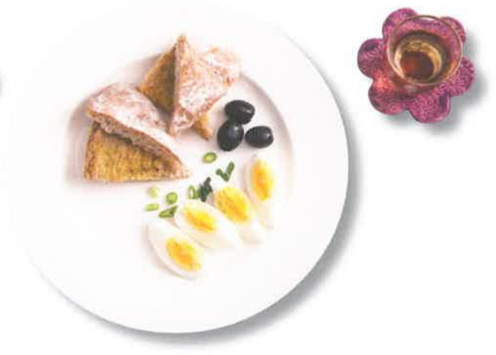

* Olijven kunnen veel zout bevatten, maar kunnen wel onderdeel zijn van een maaltijd. Het is belangrijk dat het een kleine hoeveelheid is.

9

09/2016

Bron: Voedingscentrum

Bijlage 5 Aanbevolen portiegroottes voor een kind lunch

Aanbevolen portiegroottes voor een kind

Lunch

Kind 1 - 3 jaar
- 1-2 belegde boterhammen
- glas water
- mandarijntje

Kind 4 - 8 jaar
- 2-3 belegde boterhammen
- glas water
- mandarijntje

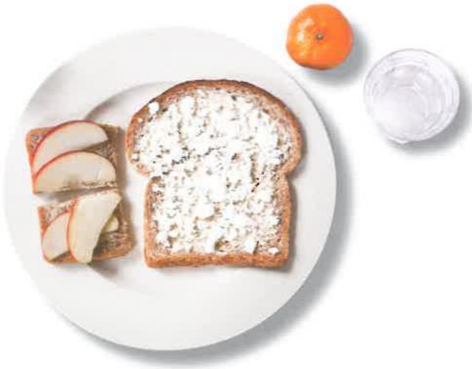

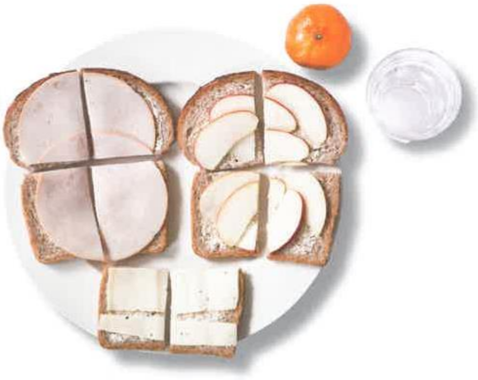

Kind 1 - 3 jaar
- 1-2 puntjes bruin Turks brood (30 gram)
- humus met reepjes gegrilde paprika
- glas water

Kind 4 - 8 jaar
- 2 puntjes bruin Turks brood (60 gram)
- 1 met humus met reepjes gegrilde paprika
- 1 met verkruimelde witte kaas en paar plakjes komkommer
- glas water

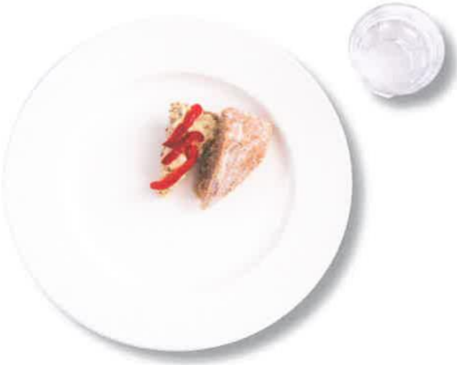

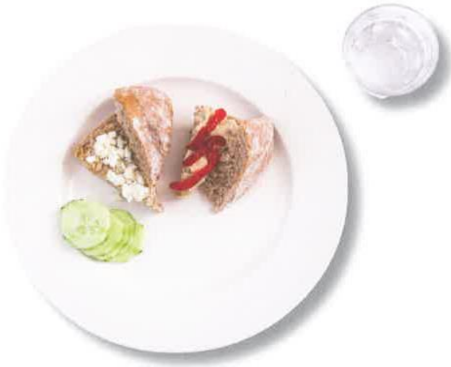

10

09/2016

Bron: Voedingscentrum

Bijlage 6 Aanbevolen portiegroottes voor een kind warme maaltijd

Aanbevolen portiegroottes voor een kind

Warme maaltijd

Kind 1 - 3 jaar
- 1-2 opscheplepels (50-100 gram) volkorenpasta
- 1-2 opscheplepels (50-100 gram) groente
- 50 gram kipfilet
- glas water

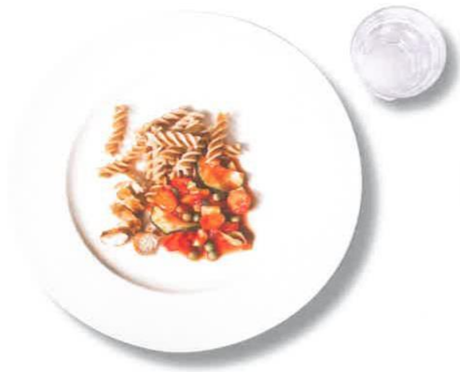

Kind 4 - 8 jaar
- 2 opscheplepels (100 gram) volkorenpasta
- 2-3 opscheplepels (100-150 gram) groente
- 50-60 gram kipfilet
- glas water

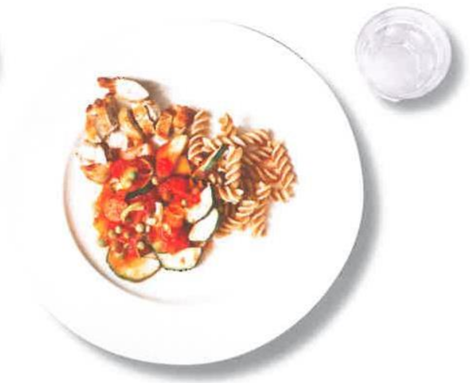

Kind 1 - 3 jaar
- 1 opscheplepel (volkoren) couscous (50 gram) gemengd met peterselie en lente-ui
- 1-2 opscheplepels groente (75 gram totaal)
- 25 gram gebakken kabeljauw
- glas water

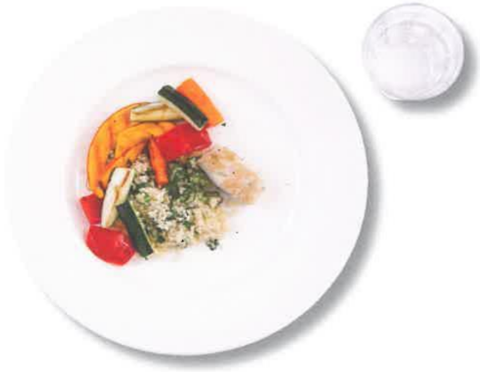

Kind 4 - 8 jaar
- 2 opscheplepels (volkoren) couscous (100 gram) gemengd met peterselie en lente-ui
- 2-3 opscheplepels groente (120 gram totaal)
- 50 gram gebakken kabeljauw
- glas water

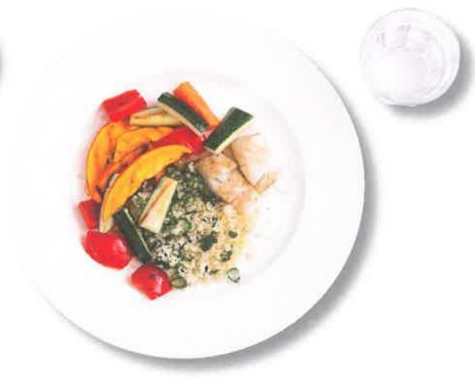

11

09/2016

Bron: Voedingscentrum

Bijlage 7 Aanbevolen portiegroottes voor een kind tussendoor/toetjes

Aanbevolen portiegroottes voor een kind

Tussendoor / toetje

Kind 1 - 3 jaar
- glas lauwe lichte thee
- ½ sinaasappel

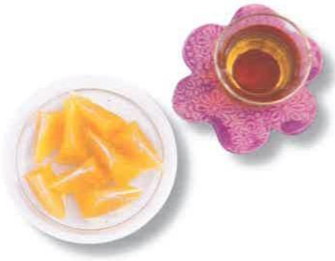

Kind 4 - 8 jaar
- glas lauwe lichte thee
- 1 sinaasappel

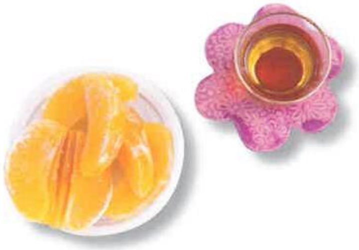

Kind 1 - 3 jaar
- magere yoghurt (150 g)
- fruit

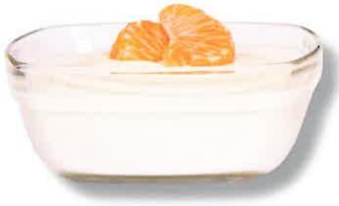

Kind 4 - 8 jaar
- magere yoghurt (150 g)
- fruit

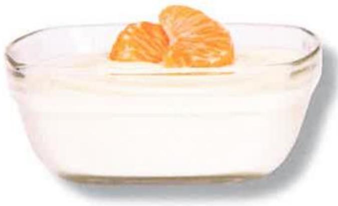

09/2016

Bron: Voedingscentrum

Bijlage 8 Aanbevolen portiegroottes voor een kind en volwassene warme maaltijd

Aanbevolen portiegroottes voor een kind en een volwassene

Warme maaltijd

Kind 1 - 3 jaar
- 1,5 opscheplepel (75 gram) zilvervliesrijst
- 1,5 opscheplepel (75 gram) courgette
- 50 gram kipfilet

Volwassene
- 4-5 opscheplepels (225 gram) zilvervliesrijst
- 4 opscheplepels (200 gram) courgette
- 100 gram kipfilet

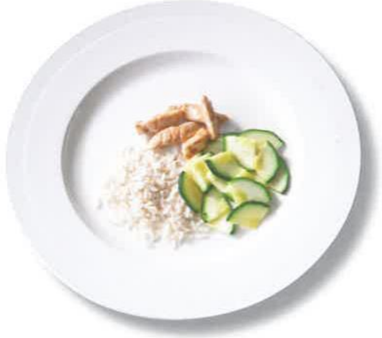

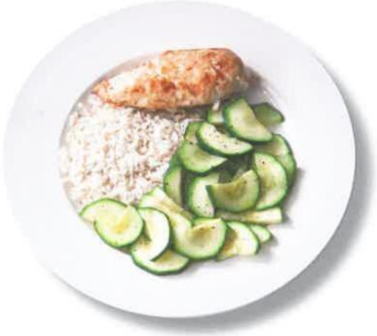

Warme maaltijd

Kind 1 - 3 jaar
- 1,5 opscheplepel (75 gram) volkoren pasta
- 1,5 opscheplepel (75 gram) groente
- 50 gram kipfilet

Volwassene
- 4-5 opscheplepels (225 gram) volkoren pasta
- 4 opscheplepels (200 gram) groente
- 100 gram kipfilet

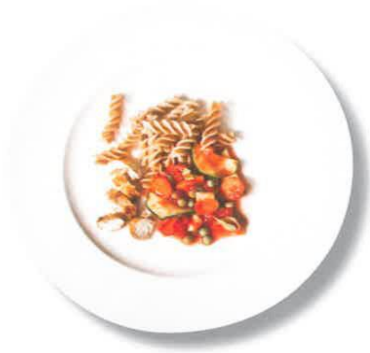

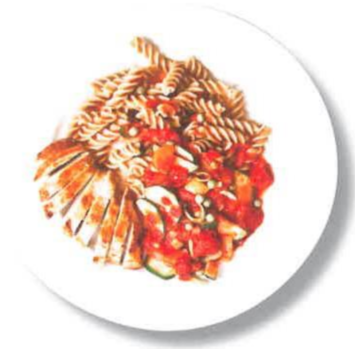

13

09/2016

Bron: Voedingscentrum

Bijlage 9 Gezonde en minder gezonde dranken

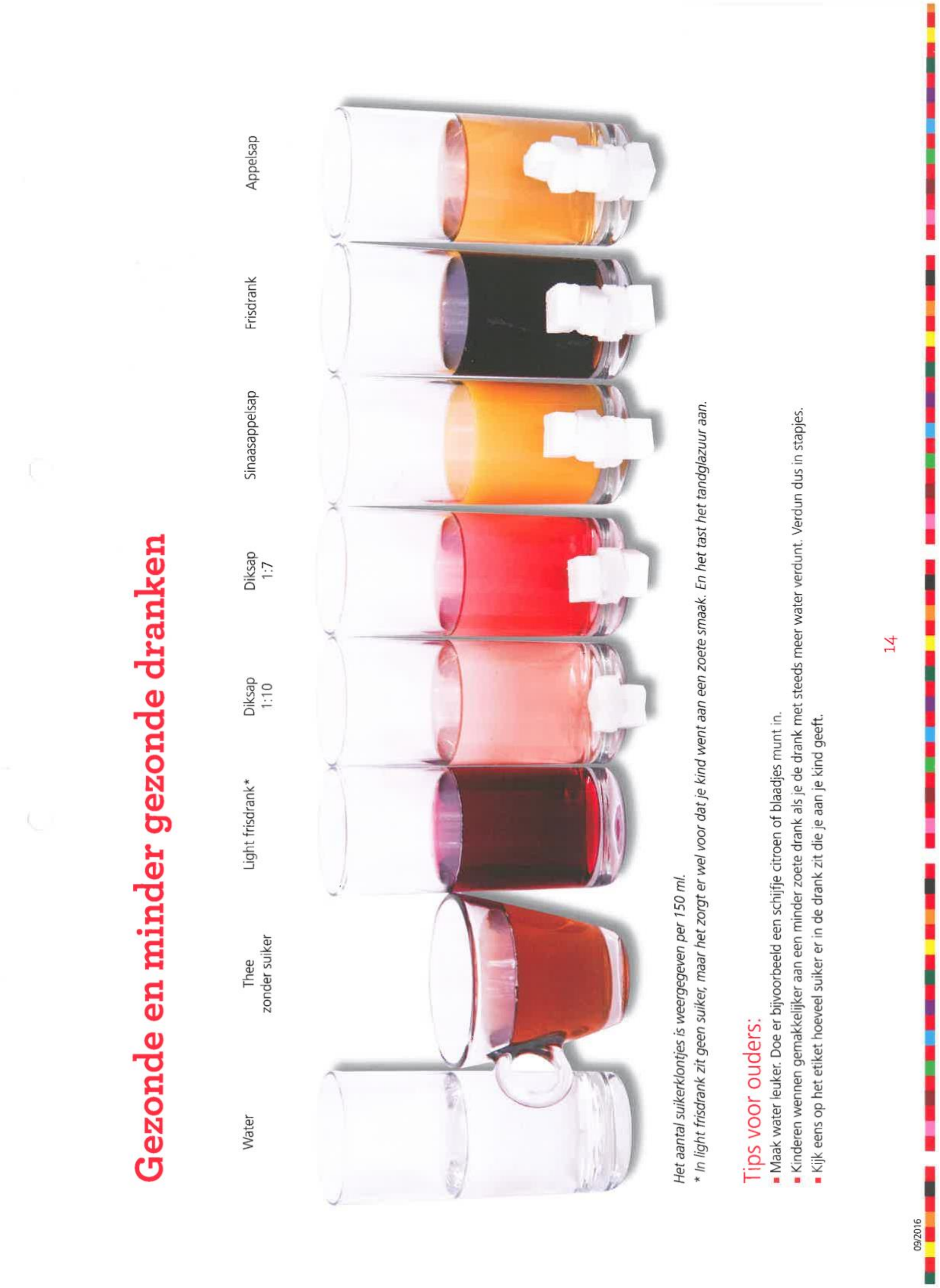

Gezonde en minder gezonde dranken

Water | Thee zonder suiker | Light frisdrank* | Diksap 1:10 | Diksap 1:7 | Sinaasappelsap | Frisdrank | Appelsap

Het aantal suikerklontjes is weergegeven per 150 ml.

** In light frisdrank zit geen suiker, maar het zorgt er wel voor dat je kind went aan een zoete smaak. En het tast het tandglazuur aan.*

Tips voor ouders:

- Maak water leuker. Doe er bijvoorbeeld een schijfje citroen of blaadjes munt in.
- Kinderen wennen gemakkelijker aan een minder zoete drank als je de drank met steeds meer water verdunt. Verdun dus in stapjes.
- Kijk eens op het etiket hoeveel suiker er in de drank zit die je aan je kind geeft.

14

09/2016

Bron: Voedingscentrum

Bijlage 10 Gezonde en minder gezonde dranken (suikerkaart)

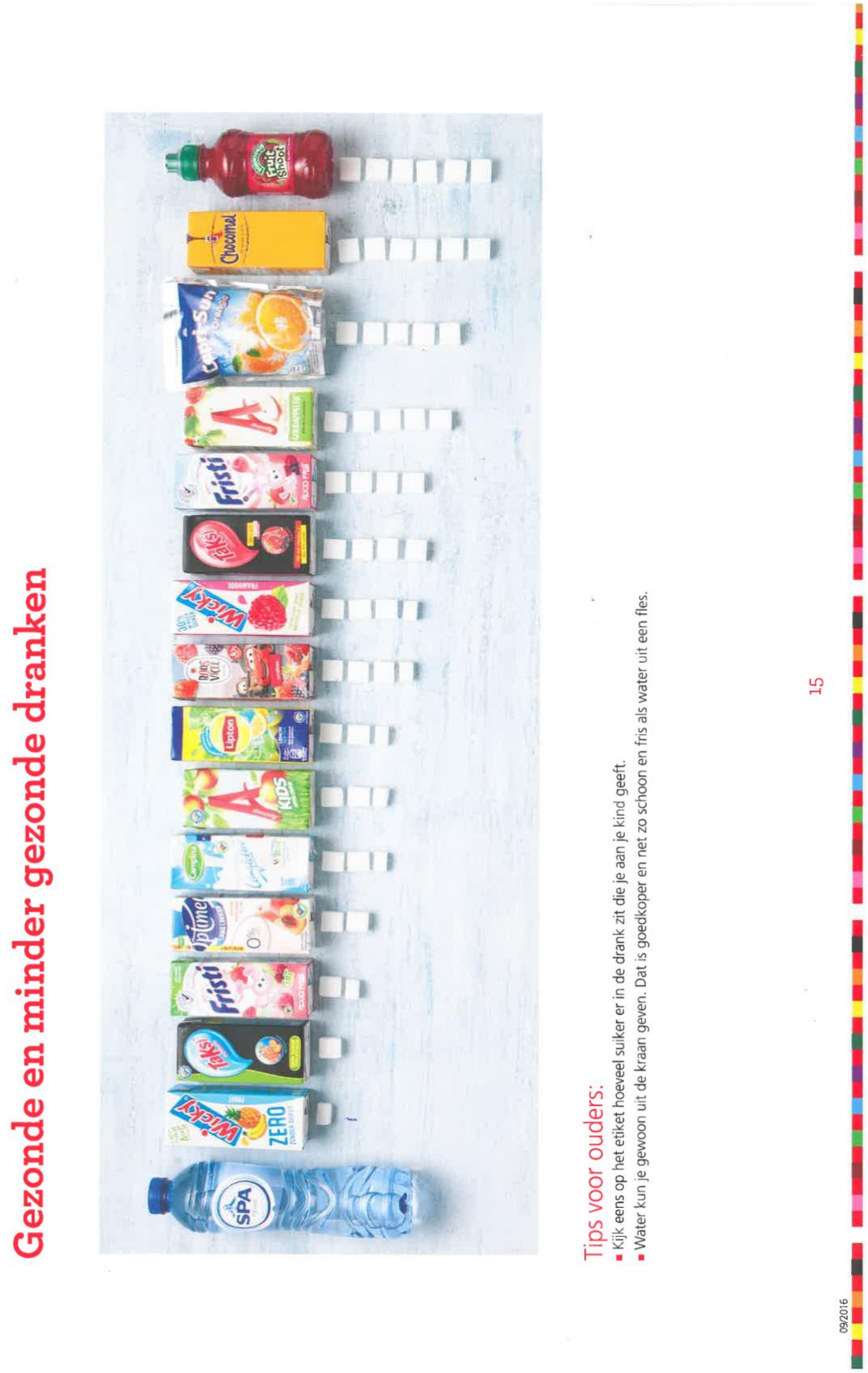

Bron: Voedingscentrum

Bijlage 11 Voorbeelden van kinderporties van niet-Schijf van Vijf producten

Voorbeelden van kinderporties van niet-Schijf van Vijf producten

Op specifieke momenten zoals in het weekend of op een verjaardag eten we allemaal wel eens iets van gebak, snoep of ijs. Een volwassen portie is snel al veel te veel voor een kind. Bij een kinderleeftijd past dus ook een kinderportie. Het advies is om onderstaande verhoudingen aan te houden:

Kind 1-4 jaar<br>max. ¼ portie van volwassen portie	Kind 4-8 jaar<br>½ portie van volwassen portie	Volwassene<br>1 portie
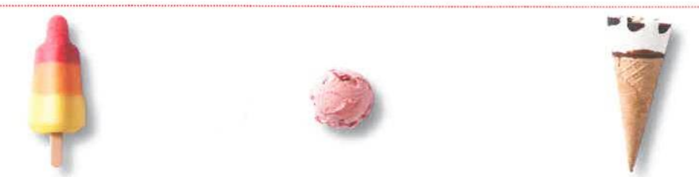		
1 waterijsje	1 bolletje ijs	1 ijshoorn
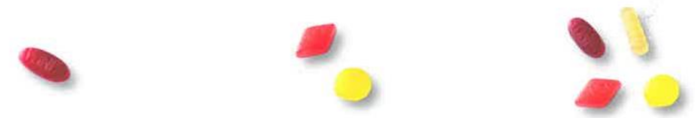		
1 snoepje	2 snoepjes	4 snoepjes
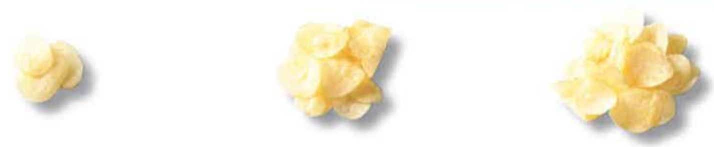		
2-3 chips	1 handje chips	2 handjes chips
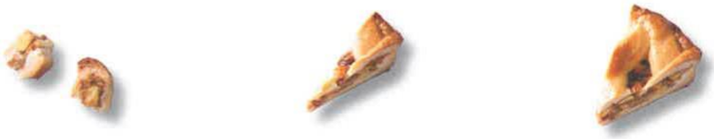		
2 happen appeltaart	½ punt appeltaart	Punt appeltaart
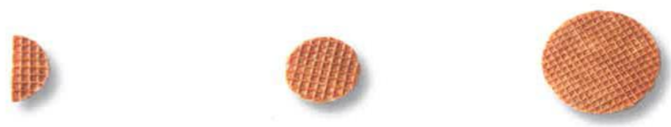		
½ mini-stroopwafel	1 mini-stroopwafel	1 stroopwafel

17

09/2016

Bron: Voedingscentrum

GO! GEZOND ONDERWEG

Bijlage 12 Beweegplanner

Beweegplanner

Bewegen is gezond en doet je goed. Het houdt je fit en het verkleint het risico op chronische ziekten. Deze Beweegplanner biedt ondersteuning bij de wens om meer te bewegen. Het geeft inzicht hoeveel je nu al beweegt en helpt om meer bewegen slim in te passen in het dagelijkse leefpatroon.

Bedenk op een vast moment in de week, voor alle dagen van de week wat je aan beweging en sport wilt gaan doen en schrijf dit op in de tweede kolom. Geef aan wat je gaat doen en hoe lang je denkt te gaan bewegen. Vink elke dag af wat je hebt gedaan. Zo zie je eenvoudig of je doel bereikt is.

Doel van deze week ____________________ dagen bewegen.
Per dag gemiddeld ____________________ minuten bewegen.

Week 1	Wat ga ik doen?	Wat heb ik gedaan?	Hoe lang? ○ = 10 minuten
**Maandag**			○○○ ○○○ ○○○ ○○○
	-<br>-<br>-	-<br>-<br>-	
**Dinsdag**			○○○ ○○○ ○○○ ○○○
	-<br>-<br>-	-<br>-<br>-	
**Woensdag**			○○○ ○○○ ○○○ ○○○
	-<br>-<br>-	-<br>-<br>-	
**Donderdag**			○○○ ○○○ ○○○ ○○○
	-<br>-<br>-	-<br>-<br>-	
**Vrijdag**			○○○ ○○○ ○○○ ○○○
	-<br>-<br>-	-<br>-<br>-	
**Zaterdag**			○○○ ○○○ ○○○ ○○○
	-<br>-<br>-	-<br>-<br>-	
**Zondag**			○○○ ○○○ ○○○ ○○○
	-<br>-<br>-	-<br>-<br>-	
**Aantal dagen bewogen ______ dagen**		**Per dag gemiddeld ______ minuten**	**Doel behaald ______ ja/nee**

Heb je het gestelde doel bereikt? **Goed gedaan!** Beloon jezelf door te luisteren naar mooie muziek, lees een leuk boek of een mooi tijdschrift of ga iets anders doen wat je leuk vindt.

Bron: Friesland Campina

Bijlage 13 Beweegkaart

In deze beweegkaart kan je samen met het kind de sportactiviteiten die het kind/jongere in de buurt kan doen invullen.

Naam kind….

Sportactiviteit	Welke dag	Waar

Bijlage 14 Schijf van vijf

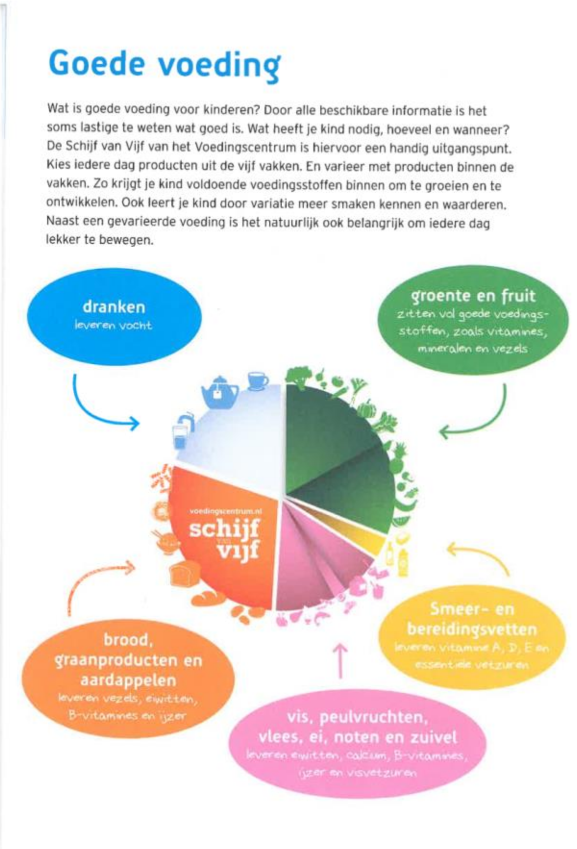

Goede voeding

Wat is goede voeding voor kinderen? Door alle beschikbare informatie is het soms lastige te weten wat goed is. Wat heeft je kind nodig, hoeveel en wanneer? De Schijf van Vijf van het Voedingscentrum is hiervoor een handig uitgangspunt. Kies iedere dag producten uit de vijf vakken. En varieer met producten binnen de vakken. Zo krijgt je kind voldoende voedingsstoffen binnen om te groeien en te ontwikkelen. Ook leert je kind door variatie meer smaken kennen en waarderen. Naast een gevarieerde voeding is het natuurlijk ook belangrijk om iedere dag lekker te bewegen.

Bron: Voedingscentrum

Bijlage 15 BMI-tabel jongens 2 tot 18 jaar

Leeftijd	*Jongens*								
(jaren)	*BMI*		*BMI*		*BMI*		*BMI*		*BMI*
	Gezond gewicht		*Overgewicht*		*Obesitas Graad 1*		*Ernstige obesitas Graad 2*		*Morbide obesitas Graad 3*
	Van	*tot*	*Van*	*tot*	*Van*	*tot*	*Van*	*tot*	*Vanaf*
2		18,4	18,4	20,1	20,1	22,5	22,5	23,6	23,6 en hoger
3		17,9		19,6		21,2		22,2	
4		17,6		19,3		20,7		21,7	
5		17,4		19,3		20,6		21,7	
6		14,04-17,6		19,8		21,0		22,2	
7		14,02-17,9		20,6		21,7		23,2	
8		14,10-18,4		21,6		23,0		24,9	
9		14,29-19,1		22,8		24,6		27,0	
10		14,53-19,8		24,0		26,4		29,5	
11		14,84-20,6		25,1		28,3		32,2	
12		15,23-21,2		26,0		30,2		34,8	
13		15,70-21,9		26,8		31,8		36,9	
14		16,25-22,6		27,6		32,9		38,4	
15		16,84-23,3		28,3		33,7		39,1	

16	17,42-23,9	28,9	34,2	39,5	
17	17,98-24,5	29,4	34,6	39,8	
18	18,50-25	30	35	40	

Bijlage 16 BMI-tabel meisjes 2 tot 18 jaar

Leeftijd	Meisjes								
(Jaren)	BMI		BMI		BMI		BMI		BMI
	Gezond gewicht		*Overgewicht*		*Obesitas Graad 1*		*Ernstige obesitas Graad 2*		*Morbide obesitas graad 3*
	Van	tot	Van	tot	Van	tot	Van	tot	Vanaf
2		18,0	18,0	19,8	19,8	21,9	21,9	23,4	23,4 en hoger
3		17,6		19,4		21,5		23,2	
4		17,3		19,2		21,6		23,5	
5		17,2		19,2		22,0		24,2	
6		13,90-17,3		19,7		22,8		25,5	
7		13,94-17,8		20,5		24,0		27,4	
8		14,06-18,4		21,6		25,6		29,8	
9		14,26-19,1		22,8		27,2		32,3	
10		14,57-19,9		24,1		28,8		34,6	
11		14,99-20,7		25,4		30,3		36,5	
12		15,52-21,7		26,7		31,6		38,0	
13		16,13-22,6		27,8		32,6		38,9	

14	16,73-23,3	28,6	33,3	39,4	
15	17,28-23,9	29,1	33,9	39,7	
16	17,75-24,4	29,4	34,3	39,9	
17	18,16-24,7	29,7	34,7	39,9	
18	18,50-25	30	35	40	

Bijlage 17 Voorbeeld groeidiagram jongens 1-21 NL

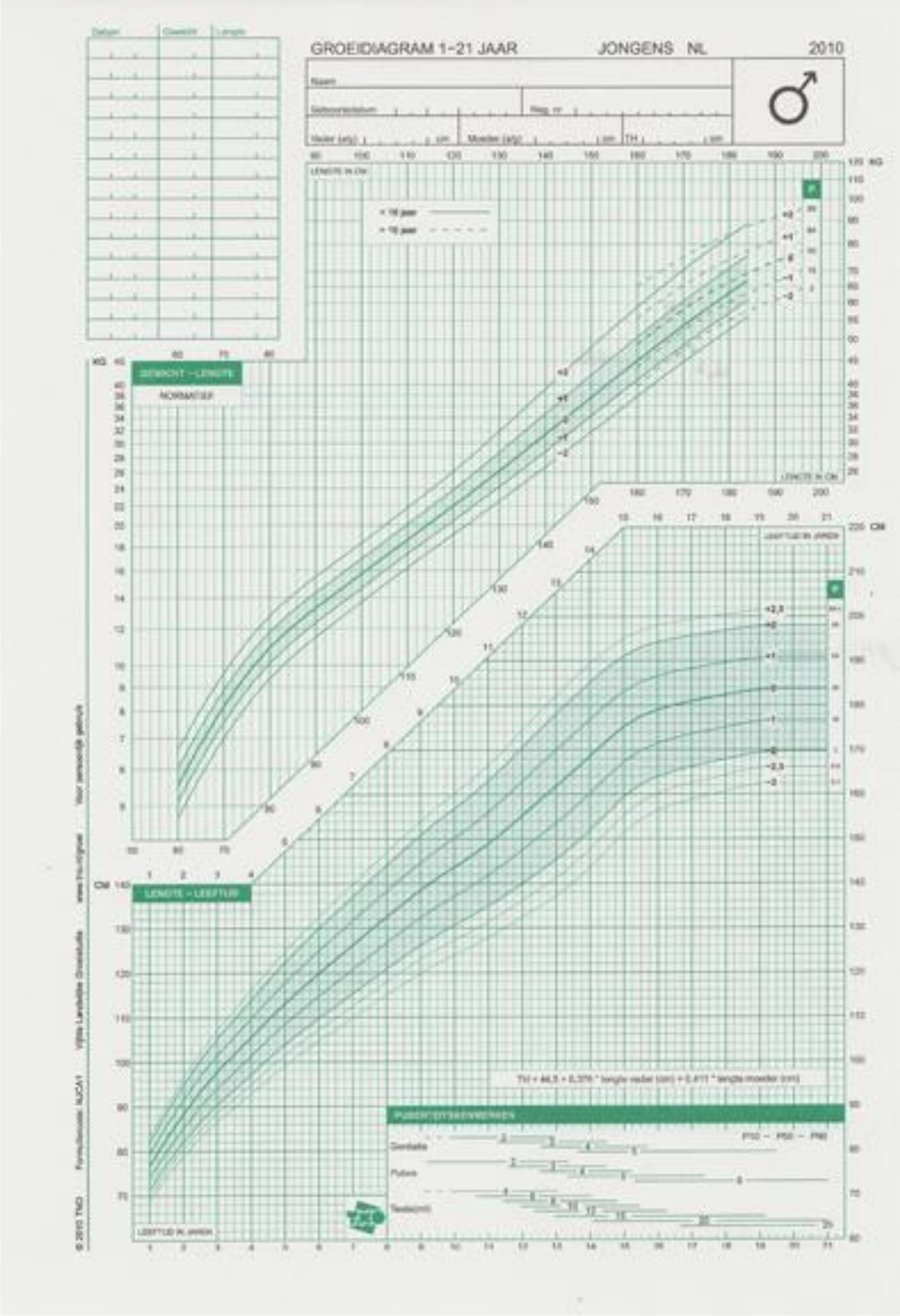

Bijlage 18 Uitleg Pedsql vragenlijsten

Wanneer de eerste afspraak (telefonisch) gepland wordt, informeer je de ouder/verzorger dat het programma GO! Gezond Onderweg meedoet aan een onderzoek naar kwaliteit van leven, genaamd Pedsql. Je legt uit wat dit onderzoek behelst en vraagt of ze daaraan mee willen doen. Bij deelname stuur je de Pedsql vragenlijst T=0 mee met het verzoek om hem ingevuld mee te terug te nemen naar de eerste afspraak. Zodoende geef je ouder de wettelijke bedenktijd om deel te nemen.
Bij de eerste afspraak geef je ouders/verzorgers een informatiebrief over dit onderzoek.

De Pedsql vragenlijst is een onderzoek naar de kwaliteit van leven, voor, tijdens en na de begeleiding van GO! De Pedsql vragenlijst is verdeeld in 5 leeftijdscategorieën.
Namelijk: 2-4 jaar, 5-7 jaar, 8-12 jaar, 13 tot 18 jaar en 18 jaar en ouder.
De lijst van 2-4 jaar is alleen voor de ouder/verzorger. De lijst voor 5-7, 8-12 jaar en 13 tot 18 jaar is voor ouder en kind. De lijst voor 18 jaar en ouder moet de jongere zelf invullen.

De meetmomenten zijn T=0 maanden, T=3 maanden, T=6 maanden, T=12 maanden en T=24 maanden. Vanaf de meetmomenten T=3; T=6; T=12 en T=24 maanden neem je de lijsten af aan het begin van je consult. Is er rond die tijd geen afspraak gepland (of deze is geannuleerd) met het kind dan stuur je de Pedsql vragenlijst per post naar het kind en de ouder/verzorger met het verzoek ingevuld te retourneren. Let op dat je het volgende noteert op de pedsql vragenlijst: studienummer, T= meetmoment, welke wijk en welke kindergezondheidscoach. Er is een marge in de meetmomenten. Zie hieronder:
Marges metingen
T=3 tussen 2 - 4,5 maanden
T=6 tussen 5 - 8 maanden
T=12 tussen 10 - 15 maanden
T=24 tussen 20 - 28 maanden

De ingevulde Pedsql lijst stuur je naar het ziekenhuis Rijnstate Arnhem. Poli kindergeneeskunde GO! team ter attentie van mw. dr. P. van Setten Interne Post 1316 Antwoordnummer 111 6800 WC Arnhem.

Bijlage 19 Terugkoppeling voortgang kind/jongere

Terugkoppeling voortgang kind/jongere naar zijn/haar huisarts, iedere 6 maanden. Terug te vinden in de dropbox van jouw kern.

Format terugkoppelingsformulier KGC naar huisarts en jeugdarts

Datum: …………………………………
Studienummer kind GO!: …………………………………
Geboortedatum kind GO!: …………………………………
Startdatum bij GO!: …………………………………
Status kind GO!:

……………………………………………………………………………………………………

……………………………………………………………………………………………………..

……………………………………………………………………………………………………..

Aandachtspunt kind GO!:

……………………………………………………………………………………………………..

……………………………………………………………………………………………………..

Toekomst/Conclusie:

……………………………………………………………………………………………………..

……………………………………………………………………………………………………..

……………………………………………………………………………………………………..

Namens Kindergezondheidscoach:

…………………………………………………

Bijlage 20 Eetdagboek voor een week

Het eetdagboek helpt jou om een beter inzicht te geven in jouw eetgewoonten. Vul elke dag in wat je eet. Van je coach krijg je 7 invulbladen mee.

Eetdagboek van een week

**Eetdagboek van ……………….**	**Dag/Datum:...................**
**Ontbijt:**	
**Tussendoor:**	
**Lunch:**	
**Tussendoor:**	
**Avondeten:**	
**Tussendoor:**	
