# Supplementary material for: Two-year effects of the community-based overweight and obesity intervention program Gezond Onderweg! (GO!) in children and adolescents living in a low socioeconomic status and multi-ethnic district on Body Mass Index-Standard Deviation Score and quality of life
Source: eClinicalMedicine. 2021 Nov 30;42:101217. doi: 10.1016/j.eclinm.2021.101217 (PMC8640234; doi:10.1016/j.eclinm.2021.101217)
Supplement: Supplementary file 2 [file mmc2.pdf]

GO! Gezond Onderweg
Interventiebeschrijving

Naam/titel interventie: GO! Gezond Onderweg
Eigenaar/licentiehouder: Rijnstate, Arnhem
Ontwikkelaars: GEZ Malburgen en Rijnstate
Consortium: VGGM, Gemeente Arnhem, Menzis, GEZ Malburgen
Auteurs: Karin Ruiter-Smit, Marjolein Postma, Marc Rinkes, Nina Meels en Petra van Setten
E-mail: info@go-nl.nl
Versie: V3

Contactpersoon
Programmamanager: Dr. Marc Rinkes
E-mail: marcrinkes@go-nl.nl
Telefoon: +31 6 52 02 71 36

Informatie interventie
Website: https://go-nl.nl/
ISBN: 9789090316093
Databank (erkenning): Interventiedatabase Gezond en Actief Leven

Inhoudsopgave

Aanleiding 3

Wat? Aanpak 4

Missie 5

Visie 5

Kernwaarden 5

Hoofddoelstellingen 6

Doelgroep 6

Wie? 7

Netwerkorganisatie 7

GO! Centraal 8

GO! Lokaal 10

Hoe? 12

1. Inventarisatie 12
2. Implementatie 12
3. Programma 13

Ketenzorg 13

Behandelprogramma kindergezondheidscoach (gezonde leefstijl coaching) 16

Multidisciplinair overleg (MDO) 18

Programmagroep 18

Multiproblematiek 19

4. Netwerkcoördinatie & kwaliteitsbewaking & wetenschap 20

Netwerkcoördinatie 20

Kwaliteitsbewaking 20

Wetenschap 20

5. GO! Community 21

Stuurgroep 21

Randvoorwaarden 22

Financiering 22

(Door) Ontwikkeling 22

Wetenschap 23

Bijlagen 24

Literatuur 25

Aanleiding

De toename van het aantal kinderen met overgewicht en obesitas in combinatie met de ernstige gevolgen voor de fysieke en psychische gezondheid, vraagt dringend om een effectieve (preventieve) interventie.

Professionals staan letterlijk en figuurlijk met de rug tegen de muur. Interventies waarbij:

I. de zorg gekenmerkt wordt door samenwerkende partijen in het zorg- en sociale domein rondom kind en gezin,
II. met een eindverantwoordelijke die het begeleidingstraject langdurig bewaakt en
III. die effectief zijn,

zijn schaars en komen veelal te laat. Dit reflecteert de sterke behoefte aan een netwerksamenwerking met zorg- en hulpverleners rondom kind en gezin met een eindverantwoordelijke die regie voert in de eigen context van kind en gezin.

Professionals in de (jeugd)gezondheidzorg worden in toenemende mate geconfronteerd met kinderen en adolescenten met overgewicht en obesitas (Lucht et al., 2010). In 2018[1] had 11.7 % van de kinderen van 4 tot en met 17 jaar oud overgewicht, waarvan 9% matig overgewicht en 2,7% ernstig overgewicht (obesitas) (Centraal Bureau voor de Statistiek, 2018)[2]. Minder bekend, maar des te schrijnender, is dat kinderartsen in toenemende mate bij kinderen geconfronteerd worden met de gevolgen van overgewicht zoals bijvoorbeeld diabetes type 2, hoge bloeddruk, hart- en vaatziekten, klachten aan het bewegingsapparaat en psychische klachten. In bepaalde gevallen blijkt kinderobesitas zelf het symptoom te zijn waarvan de achterliggende oorzaken dienen te worden aangepakt. Onderzoek wijst uit dat overgewicht en obesitas op jonge leeftijd respectievelijk gerelateerd zijn aan overgewicht en obesitas op volwassen leeftijd (Geserick et al., 2018). Ook zijn overgewicht en obesitas geassocieerd met een verhoogd risico op morbiditeit en mortaliteit bij volwassenen, onafhankelijk van hun gewicht op volwassen leeftijd (Freemark, 2018; Magarey et al., 2003; Freedman et al., 2001). Deze langdurige impact en samenhang met tal van ziekten brengt tevens enorme zorgkosten en maatschappelijke kosten met zich mee. Dit maakt overgewicht één van de grootste gezondheidsproblemen bij kinderen op dit moment (Kent et al.,2017, Neovius et al.,2012).

---

[1] Overgewicht (2018): 4-12 jr 11.9%; 12-16 jr 11.6%; 16-20 jr 15.9%
Ernstig overgewicht (2018): 4-12 jr 3.4%; 12-16 jr 2.6%; 16-20 jr 3.4%

[2] Onder bevolkingsgroepen met lage SES en niet westerse etniciteit liggen deze percentages beduidend hoger

Wat? Aanpak

GO! Gezond Onderweg[3] is een aanpak om kinderen en jongeren met ernstig overgewicht op te sporen en in hun eigen omgeving te coachen naar een gezondere leefstijl. De aanpak kan beschreven worden aan de hand van de volgende drie assen:

I. Netwerksamenwerking met alle zorg- en hulpverleners in de wijk[4] rondom kind en gezin.

Vanuit een multidisciplinair team van zorg- en hulpverleners in de wijk, uit zowel het sociale als het zorgdomein, wordt met een op maat gemaakt behandelplan toegewerkt naar een gezondere leefstijl door middel van duurzame gedragsverandering.

II. De kindergezondheidscoach/centrale zorgverlener (KGC/CZV)[5] is spin in het web.

Kenmerkend is de inzet van een KGC wiens kerntaak is:

a. Centrale zorgverlener, hetgeen betekent dat hij/zij dmv een brede anamnese op zoek gaat naar onderliggende (multi)problematiek en wanneer deze aanwezig is passende zorg daarvoor inzet
b. Het coachen en begeleiden is van het kind en/of de jongere en zijn/haar gezin gedurende minimaal twee jaar is. Coaching vindt plaats op 3 domeinen: voeding, beweging, rust & ontspanning
   De KGC positioneert zich zo in het bestaande lokale netwerk, dat hij/zij wordt opgenomen in het professionele en het privé netwerk. Op deze manier bouwt de KGC een samenwerking op met de betrokken professionals en sociaal-maatschappelijke partners in de wijk, waarbij de bestaande samenwerking en verbindingen worden gerespecteerd. Vanuit deze schakelpositie is het de taak van de KGC om het onderliggende primaire probleem te achterhalen en daarbij gezamenlijk met professionals in het multidisciplinair team een passende en integrale aanpak voor het kind/de jongere (en zijn/ haar omgeving) uit te voeren. Ouders worden meegenomen in het behandeltraject zodat zij support en het goede voorbeeld geven wat betreft gezonde voeding, gezond bewegen en gezond gedrag.

Samenvattend: de KGC gaat aan de slag met kind en gezin in een netwerk, bestaande uit ketenzorg én in samenwerking met sociaal maatschappelijke partners.

III. Dichtbij huis, lokaal

Het coachen van kind (en gezin) vindt altijd in de wijk plaats, dichtbij huis en in hun eigen context.

[3] Voor de leesbaarheid in dit document zal de interventie/GLI GO! Gezond Onderweg afgekort worden tot GO!

[4] Voor de leesbaarheid in dit document wordt de term 'wijk' gebruikt als het over het gebied gaat waar GO! actief is of geïmplementeerd wordt. Hiervoor kan dus ook kern, stad, dorp of gemeente gelezen worden

[5] GO! is conform het landelijk model, ketenaanpak voor overgewicht en obesitas. Voor kindergezondheidscoach kan ook centrale zorgverlener gelezen worden maar voor de leesbaarheid zal in dit document de afkorting van kindergezondheidscoach worden gebruikt: KGC

Missie

GO! zorgt ervoor dat kinderen vanuit hun eigen kracht gezonde keuzes kunnen maken - een leven lang. Hiermee creëert GO! wijken met gelukkige en gezonde kinderen.

Visie

GO! start in de directe leefomgeving van het kind en gezin en zorgt voor de verbinding tussen zorgprofessionals en sociale partners, waarin de kindergezondheidscoach het kind met obesitas en zijn/haar gezin coacht naar een gezonde leefstijl.

Kernwaarden

GO! staat voor **betrokkenheid** bij kind en gezin in hun eigen omgeving, waarbij de hulpvraag voorop staat. Door **het slaan van bruggen** tussen zorg en hulpverleners en het meenemen van de omgeving creëert GO! **gedeelde verantwoordelijkheid** in de wijk en ondersteunt daarmee kind en gezin om vanuit hun eigen kracht de gezonde keuzes te (gaan) maken.

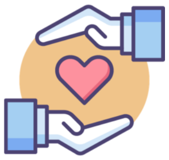

Betrokken

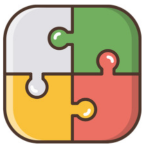

Verbinden

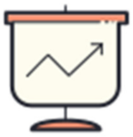

Validiteit

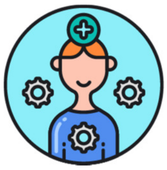

Gedeelde verantwoordelijkheid

Hoofddoelstellingen

1. Het bevorderen van de positieve gezondheid[6] door middel van de zes dimensies[7] van gezondheid
2. Het opzetten van een netwerk bestaande uit ketenzorg met zorgprofessionals en samenwerking met sociaal maatschappelijke partijen
3. Het signaleren van multiproblematiek en het zoeken van passende ondersteuning door professionals met oog voor kind en gezin
4. Teweegbrengen van een gezondere leefstijl door middel van langdurige coaching op basis van 3 pijlers: Voeding, Beweging en Rust & Ontspanning

Doelgroep

De doelgroep bestaat uit kinderen tussen de 0 - 19 jaar in de regio Midden- Gelderland met ernstig overgewicht/ obesitas graad I, II en III (met of zonder co-morbiditeit en/of risicofactoren), inclusief gezinsleden.

Naast de primaire doelgroep (kinderen met ernstig overgewicht en hun gezin) zijn ook andere stakeholders in beeld. De belangrijkste hiervan zijn:

- Professionals in de wijk (huisartsen, jeugdartsen en jeugdverpleegkundigen, kindergezondheidscoaches, fysiotherapeuten, diëtistes, psychologen, kinderwerkers en maatschappelijk werkers, welzijnswerkers) en de organisaties die zij vertegenwoordigen (geïntegreerde gezondheidscentra/GEZ, gezondheidscentra, wijkteams, GGD, ziekenhuis, etc.)
- Sociaal-maatschappelijke partijen (vb. scholen, sport bedrijven)
- Gemeenten en zorgverzekeraars (met name Menzis) in de regio Midden-Gelderland
- Landelijke coalitie: JOGG, C4O/VU, RIVM, NJi + NCJ

[6] GO! sluit hierbij aan bij het gedachtegoed van Machteld Huber

[7] Zes dimensies: Lichaamsfuncties, mentaal welbevinden, zingeving, kwaliteit van leven, sociaal maatschappelijke participatie en dagelijks functioneren

Wie?

Netwerkorganisatie

GO! is een netwerkorganisatie. Dit netwerk wordt centraal (GO! Centraal) door een projectteam gefaciliteerd vanuit Rijnstate en richt zich gezamenlijk met regionale en/of landelijke partners op de doorontwikkeling en opschaling van GO!. Lokaal (GO! Lokaal) zijn (de wijken van) gemeenten de locatie waar GO! wordt geïmplementeerd en uitgevoerd. Er is regionaal en landelijk een nauwe samenwerking met gemeenten, zorgverzekeraar, JOGG, JGZ en VWS. De netwerkorganisatie ziet er als volgt uit:

Figuur 1. Netwerkorganisatie GO! Gezond Onderweg van centraal tot lokaal

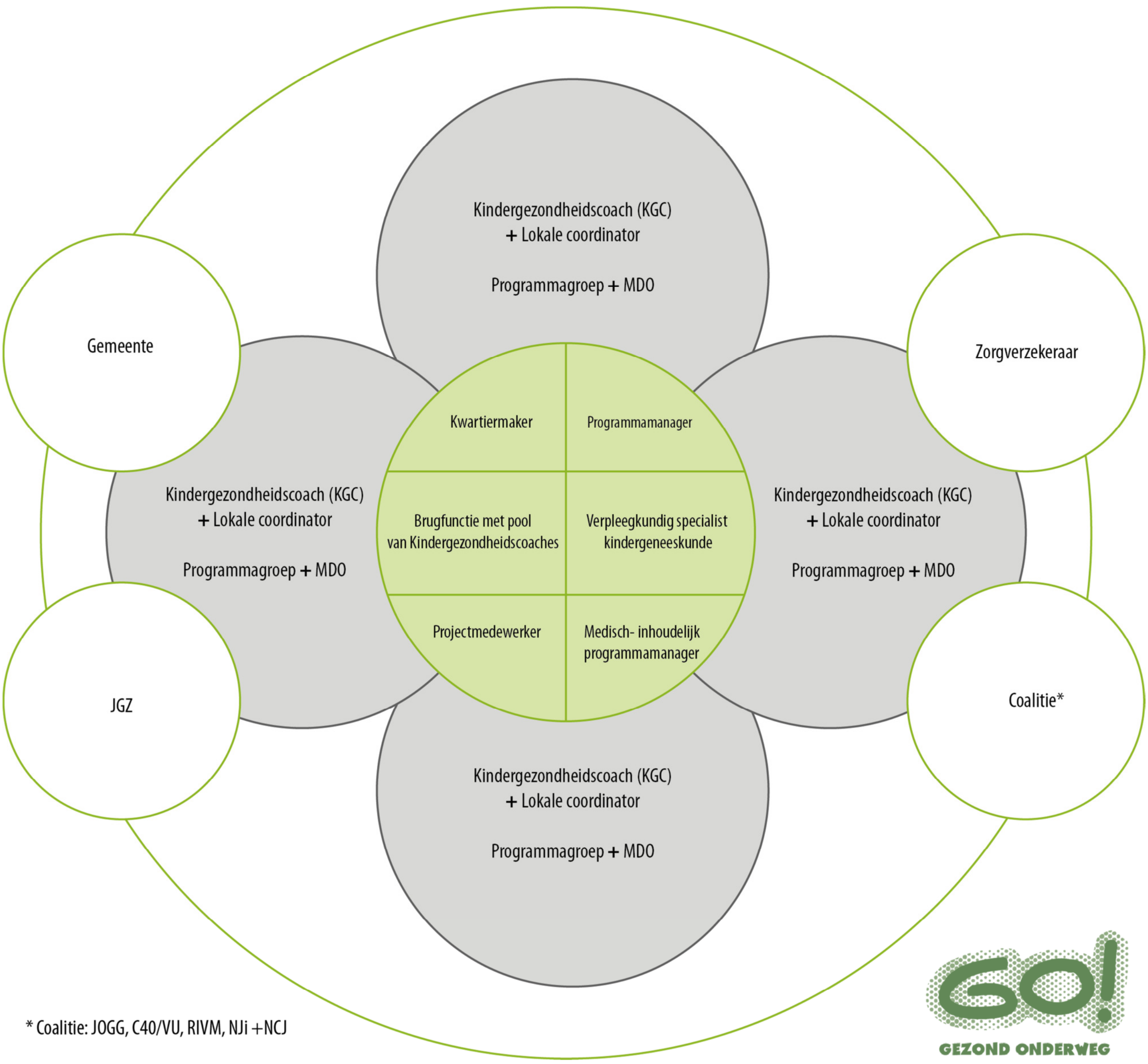

In Figuur 1 is GO! Centraal afgebeeld met het middelste groene rondje waarin de volgende functies worden vervuld:

1. Medisch- inhoudelijk programma manager
2. Programma manager
3. Verpleegkundig specialist kindergeneeskunde
4. Brugfunctie met pool van kindergezondheidscoaches
5. Projectmedewerker
6. Kwartiermaker

In geval van GO! Lokaal gaat het om de samenwerking tussen de kindergezondheidscoach, de lokale coördinator en alle professionals uit het sociale -en zorgdomein. Deze samenwerking kenmerkt zich door twee overlegvormen: het multidisciplinair overleg (MDO) en de programmagroep. In de volgende hoofdstukken wordt verder ingegaan op deze twee overlegvormen en op de verschillende functies met bijbehorende taken en verantwoordelijkheden.

In de buitenste cirkel is de samenwerking op regionaal en landelijk niveau afgebeeld met gemeenten, zorgverzekeraars, JGZ en de landelijke Coalitie ketenaanpak kinderobesitas.

GO! Centraal

GO! Centraal bestaat uit de volgende kernspelers:

Programmamanager

De programmamanager is verantwoordelijk voor de dagelijkse leiding en bedrijfsvoering, alsmede het aansturen van de doorontwikkeling van (programma) GO! in brede zin. De verantwoordelijkheid van de programmamanager is ervoor te zorgen dat GO! geïmplementeerd wordt in de wijken conform opgestelde specificaties en binnen de overeengekomen randvoorwaarden, waarmee GO! in staat is de voorziene baten, zoals gedefinieerd in het bekostigingsmodel, te realiseren. De programmamanager dient mogelijkheden te creëren voor continue verbetering en opschaling van GO! (professionalisering, interventie uitbreiding, innovatie, inbedding in netwerk, financiering etc). De taken van de programmamanager bestaan met name uit:

- Beleid en bestuur (visie, strategie, planning, regie voering van programmaonderdelen)
- Werving en acquisitie
- Leidinggeven en motiveren
- Communicatie, PR en netwerken

Kinderarts / medisch-inhoudelijk programmamanager

De kinderarts is de medisch-inhoudelijk programmamanager. Samen met de programmamanager is zij eindverantwoordelijk voor de kwaliteit van het programma GO!. De focus van de kinderarts ligt met name op het medische en wetenschappelijke vlak. Daarbij ziet zij dan ook specifiek toe op de inhoudelijke medische kwaliteit van de ketenzorg, inclusie van patiënten, behandeling door de coach, etcetera, alsmede de opzet en uitvoering van wetenschappelijke evaluaties.

Verpleegkundig specialist kindergeneeskunde

De verpleegkundig specialist brengt de sociale pediatrische en medische inbreng vanuit de 2^{e} lijn in de MDO's en Programmagroep bijeenkomsten. Tevens wordt een belangrijke bijdrage geleverd in het beheer en de ontwikkeling van nieuwe en bestaande protocollen. De verpleegkundig specialist staat onder supervisie van een kinderarts. Tevens vormt zij een brug functie richting het expertise centrum obesitas 2^{e} lijn kindergneeskunde.

Brugfunctie met pool van kindergezondheidscoaches

De pool van van kindergezondheidscoaches (zie hoofdstuk lokale uitvoerders en partners) vervullen gezamenlijk de volgende taken:

1. Intervisie van de KGC's die onderdeel uitmaken van GO!
2. Toezien op de naleving van de kwaliteitsstandaarden en signaleren van (mogelijke) problematiek of kansen
3. Vormen van de schakel tussen de coaches en het projectteam GO! en deelnemen aan project team meetings
4. Opleiden en inwerken van nieuwe KGC's tijdens de opschalingsfase en intervisie/terugkom activiteiten
5. De schakel tussen de lokale coördinator en het MDO
6. Werving, selectie en inwerken van nieuwe KGC's tijdens het kwartiermaken/implementatiefase.
7. Bijdrage leveren in de ontwikkeling en onderhoud van nieuwe en bestaande protocollen.
8. Bijdrage leveren in de doorontwikkeling van de methodiek GO!

Projectmedewerker

De projectmedewerker steunt de GO! organsiatie daar waar nodig. Naast het faciliteren op centraal niveau (administratie, media, financien) wordt tevens ondersteuning geboden aan specifieke vragen vanuit de lokale coordinatoren.

Kwartiermaker

De kwartiermaker is verantwoordelijk voor de implementatie van GO! in een nieuwe wijk. De belangrijkste taken zijn het uitvoeren van de scan om de wijk in kaart te brengen (doelgroep en netwerk), het implementeren van de ketenzorg, opzetten of verstevigen van het gehele netwerk (zorg- en hulpverleners, sociaal-maatschappelijk netwerk) en het ondersteunen van de lokale coördinator om zijn/haar rol te pakken. Hierbij wordt gebruik gemaakt van de door GO! ontwikkelde implementatie strategie en stappenplan. Na 10 weken moet de wijk gereed staan om de eerste kinderen in GO! te includeren. Op dit moment zal de kwartiermaker naar de achtergrond treden.

GO! Lokaal

GO! Lokaal bestaat uit de volgende lokale uitvoerders en partners:

Kind en Gezin

Het kind met overgewicht/obesitas en zijn of haar gezin zijn de belangrijkste spelers binnen GO!. Dit is waar het hele GO! programma om draait: het coachen naar een gezonde leefstijl met deze doelgroep en daarmee het verhogen van de kwaliteit van leven op korte en lange termijn.
Het coachingsprogramma richt zich op het individuele kind (met gezin) en zal daarom altijd maatwerk zijn. Op deze manier wordt voor iedereen een passend programma geboden. In het begin van het coachingstraject start de KGC met afnemen van de brede anamnese om onder andere te achterhalen of er sprake is van multiproblematiek. Verder voert de KGC voornamelijk de regie over de leefstijlverandering. Essentieel onderdeel van het traject is dat gedurende het programma deze regievoering juist aan kind en ouders wordt overgedragen. Dit is een absolute voorwaarde om uiteindelijk tot een succesvolle duurzame gedragsverandering in leefstijl te kunnen komen. Als die kanteling nog niet lukt omdat kind en ouders nog onvoldoende in staat zijn om deze rol op zich te nemen, dan staat vergroten van zelfredzaamheid en motivatie voorop.

Kindergezondheidscoach

De KGC is, zoals eerder beschreven, spin in het web binnen de uitvoering van GO! in een wijk. Dit betekent dat de KGC GO! op locatie uitvoert volgens het stroomschema en protocollen. Zij zullen altijd starten met het afnemen van de brede anamnese om te evalueren of er belemmerende factoren zijn om te te starten met gezonde leefstijl coaching.
Wanneer de tijd rijp is voor gecombineerde leefstijl coaching zullen zij kind en gezin minimaal 2 jaar begeleiden op de volgende drie pijlers: beweging, voeding en rust & ontspanning. De KGC's zijn geselecteerd op de aanwezige intrinsieke motivatie en beschikken over een breed pallet van vaardigheden. Zij zijn in staat om gezamenlijk met de lokale coördinator en projectmedewerkers van GO! de kwaliteit van GO! te bewaken en het netwerk te verbeteren rondom het kind. Zij zijn bekend met het gegeven dat multiproblematiek bij obese kinderen en hun gezin in hoog percentage voorkomen en zijn zij opgeleid om dit te signaleren en hebben tools om dit zichtbaar te krijgen. Wanneer er sprake blijkt van multiproblematiek zullen zij passende hulp inschakelen]. De opleiding vindt plaats aan de hand van een standaard hand- en werkboek GO! en helpen elkaar te verbeteren door regelmatige intervisie. Het functieprofiel van de KGC is opgenomen in appendix A.

Lokale coördinator

De lokale coördinator geeft vanuit de filosofie van GO! sturing aan de organisatie van GO! vanuit de wijk. Tijdens de implementatiefase wordt deze coördinator door de kwartiermaker ondersteund om zijn/haar rol te kunnen nemen in het lokale netwerk. De lokale coördinator is een sleutelfiguur in het lokale netwerk en kan goed schakelen met zowel zorg- en hulpverleners, als andere sociaal-maatschappelijk betrokken partijen. Tevens is hij/zij met de KGC de schakel tussen GO! Lokaal en Centraal is hij/zij medeverantwoordelijk voor het bewaken van de kwaliteit. Zie appendix B voor het functieprofiel van de lokale coördinator.

Zorgprofessionals & professionals sociale domein

De zorgprofessionals en professionals uit het sociale domein in een wijk spelen een belangrijke rol voor GO! De zorgprofessionals hebben een belangrijke verantwoordelijkheid in de ketenzorg voor kinderen met overgewicht en obesitas. Aan de hand van een stroomschema en protocollen hebben zij hun eigen taken en verantwoordelijkheden binnen GO!. Met de volgende professionals uit het sociale en zorgdomein zal intensief worden samengewerkt en tevens werken zij onderling nauw samen:

- Jeugdarts/jeugdverpleegkundige
- Huisarts
- Verpleegkundig specialist kindergeneeskunde
- Diëtist
- Fysiotherapeut
- Psycholoog
- Wijkcoach/jeugdconsulent
- Kinderwerker / maatschappelijk werker (vanuit sociale wijkteam)
- Interne begeleiders (IB'er)
- Zorgcoördinatoren

Sociaal-maatschappelijke partijen

Lokale sociaal-maatschappelijke partijen spelen ook een belangrijke rol binnen GO!. Deze partijen beschikken vaak over kennis van het lokale netwerk en kinderen en gezinnen. Daarnaast hebben zij vaak invloed, of kunnen zij invloed hebben, op de leefstijl van kind en gezin. Denk bijvoorbeeld aan organisaties als scholen, kerken en moskeeën, sportaanbieders en JOGG. GO! maakt gebruik van bestaande partijen in een wijk en probeert aan te sluiten en waar nodig het lokale aanbod te verstevigen. Dit moet leiden tot een gesloten cirkel, waardoor kind & gezin van alle lokale partijen en organisaties dezelfde boodschap ontvangen met betrekking tot een gezonde leefstijl.

Hoe?

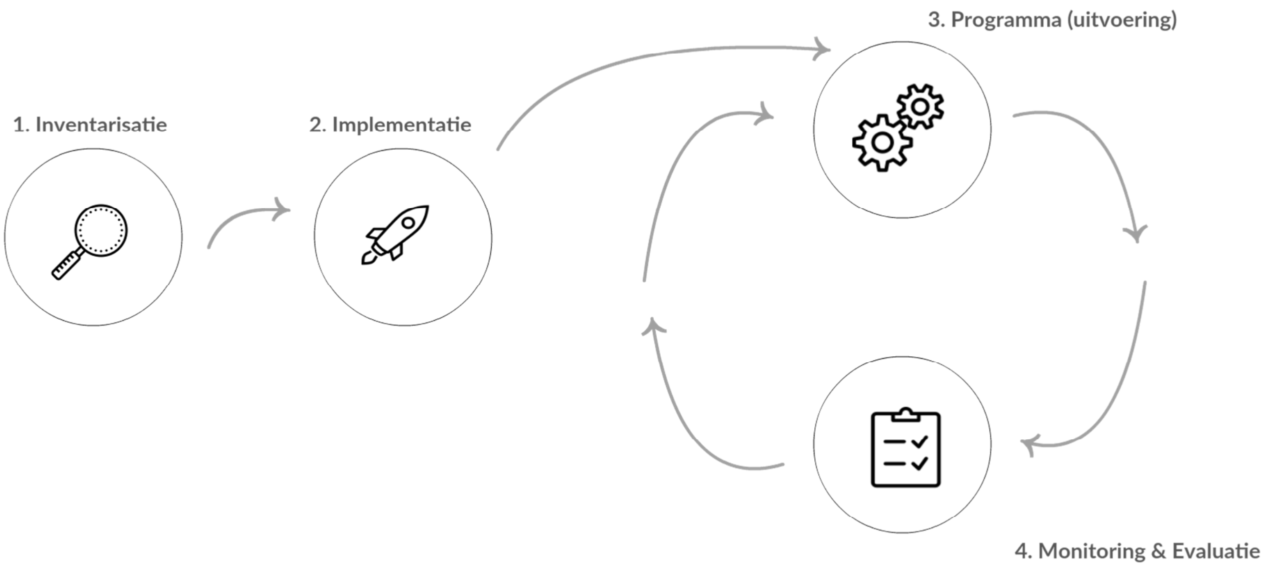

1. Inventarisatie

De inventarisatie heeft tot doel om informatie te verzamelen waarmee het mogelijk is om te bepalen of en hoe met de aanpak van GO! in een wijk aan de slag te gaan en om in beeld te brengen wat de mogelijkheden en beperkingen zijn. Deze informatie wordt verzameld door middel van gesprekken met de gemeente en andere belanghebbende en door het uitzetten van de door GO! ontwikkelde wijkscan. De scan geeft antwoord op vragen rondom de urgentie, potentie en randvoorwaarden binnen de wijk, zie appendix C.

2. Implementatie

Wanneer GO! van start gaat in een nieuwe wijk wordt gestart met het implementatietraject. Deze wordt uitgevoerd middels de implementatiestrategie en het implementatie stappenplan (zie appendix D en E).

Het hoofddoel is om binnen 10 weken te komen tot de implementatie van GO! in de wijk, zodat daarna gestart wordt met de inclusie van kinderen in het programma.
Subdoelen die bij de implementatie van belang zijn, zijn de volgende:

- Zicht krijgen op de benodigde zorg- en hulpverleners, hen enthousiasmeren, betrekken en begeleiden de juiste rol te pakken door middel van 3 sessies:
  - Sessie 1: informeren over GO! als interventie en het implementatieproces en het peilen van interesse voor verdere betrokkenheid onder de aanwezigen
  - Sessie 2: eigenaarschap creëren door gezamenlijk te brainstormen over de belangrijkste vragen in de vorm van de World Café Method.
  - Sessie 3: overdracht van de protocollen en verwijs schema's
- Inbedding van GO! in bestaande wijk, aanhaken op wat er al is qua ketenzorg en netwerk en samenwerkingsverbanden, inclusief de drie pijlers: beweging, voeding en rust & ontspanning
- Zicht krijgen op potentiële lokale coördinatoren en kindergezondheidscoaches
- Selecteren en trainen/begeleiden van de nieuwe lokale coördinator en kindergezondheidscoach
- Zorgdragen voor juiste mogelijkheden en faciliteiten om GO! ook daadwerkelijk te starten
- Creëren van bekendheid en zichtbaarheid van GO!

De resultaten aan het eind van het implementatietraject zijn de volgende:

- Uitgevoerde scan
- Opgebouwd ketenzorg verband
- Opgebouwd netwerk met sociaal/maatschappelijke partners (inclusief aansluitende programma's op het gebied van voeding, beweging en rust & ontspanning)
- Lokale coördinator en kindergezondheidscoach[8] die van start zullen gaan
- Ingeregelde randvoorwaarden rondom facilitatie GO!
- Kick-off bijeenkomst (zie appendix L)
- (Klaar voor de) start met werving van kinderen (en gezin)

Dit ziet er als volgt uit:

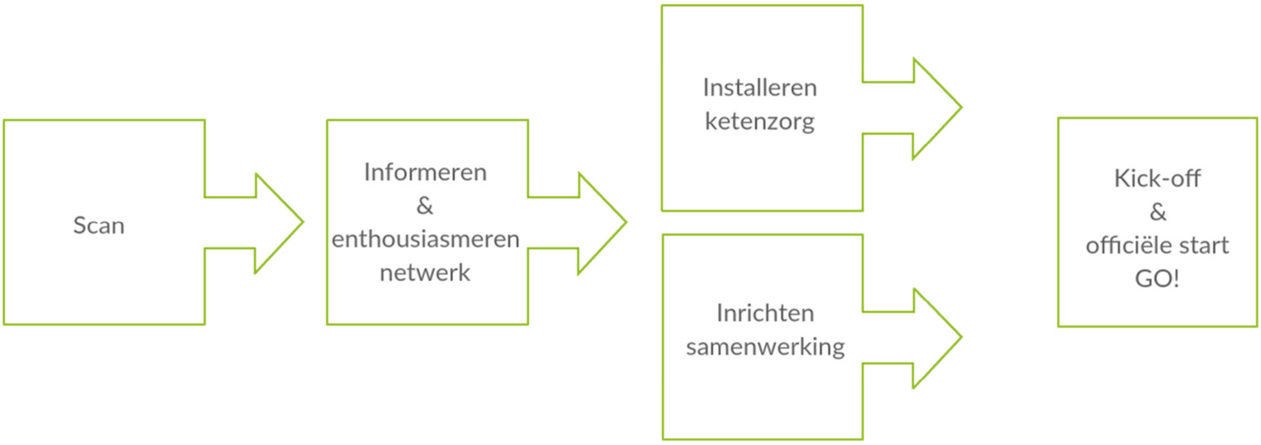

3. Programma

Ketenzorg

Dit hoofdstuk beschrijft hoe de interventie de zorg rondom kinderen met overgewicht en obesitas organiseert. De beschreven organisatiestructuur rondom betrokken zorgverleners wordt doorgaans aangeduid als ketenzorg. Onderstaand stroomschema (figuur 2.) geeft de ketenzorg van GO! schematisch weer. Het geeft inzicht in de verschillende wegen en met welke achterliggende (medische) gedachte wordt verwezen tussen zorgverleners en de KGC's van GO!.

[8] Het inhuren van een KGC is optioneel voor de wijk. Wanneer een wijk zelf een competente KGC aan kan stellen (die voldoet aan het functieprofiel en conform de kwaliteitsnorm van GO! is ingewerkt), heeft dit de voorkeur..

Stroomschema ketensamenwerking

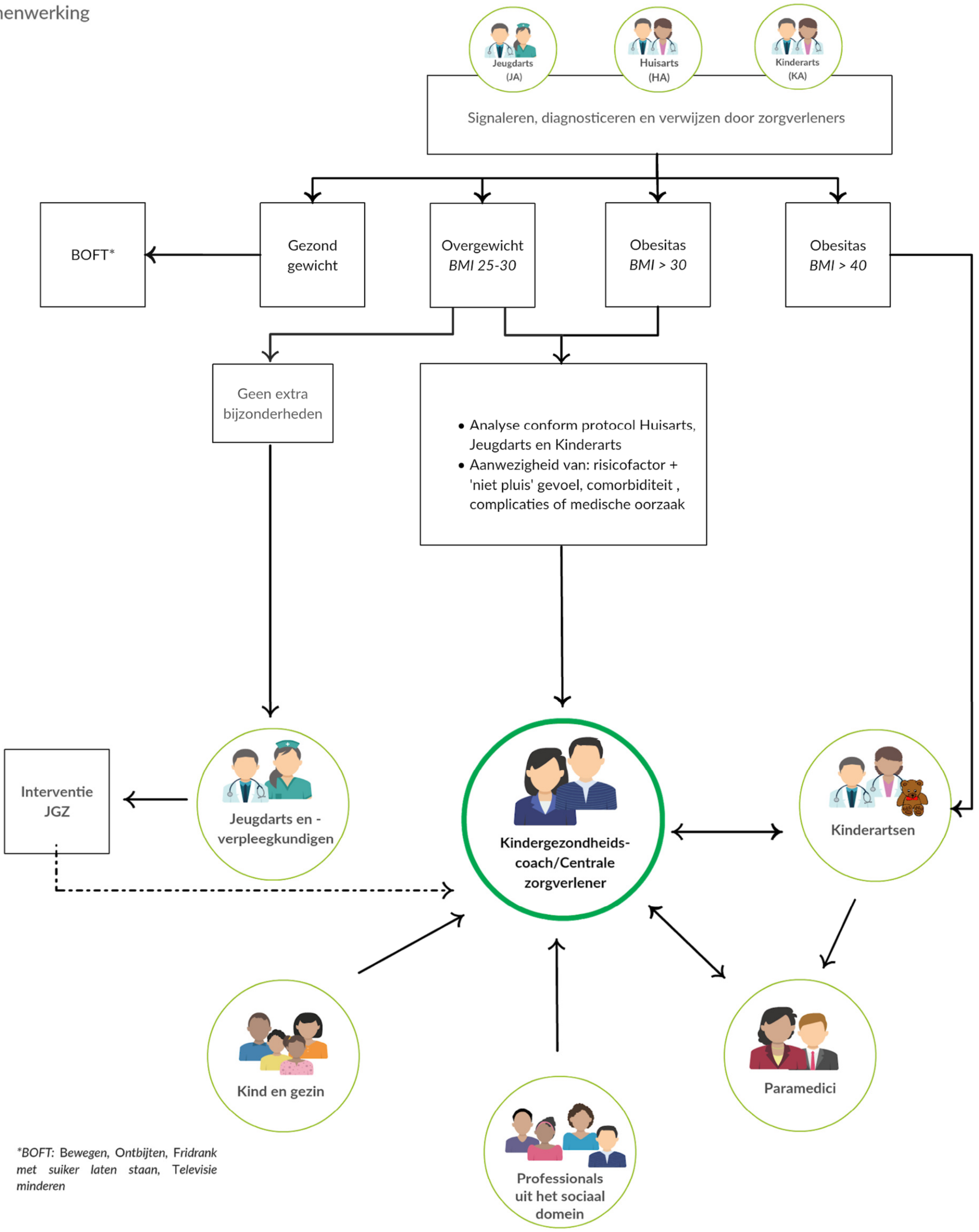

Dit stroomschema is conform het landelijk model, ketenaanpak voor overgewicht en obesitas

Signaleren en Diagnosticeren

Zoals in het stroomschema is weergegeven, begint het zorgproces met de signalering door verschillende zorgverleners. Daarna wordt geëvalueerd van welke mate van overgewicht/obesitas sprake is door de lengte en het gewicht van de patiënt te meten, en de BMI te berekenen[9]. Hierbij is het belangrijk te realiseren dat er voor kinderen geslachtsafhankelijke afkappunten zijn, zie onderstaande piramide en tabel met hierin de niveaus van gewichtsgerelateerd gezondheidsrisico (GGR) bij kinderen. Vervolgens wordt GGR bepaald aan de hand van de aan- of afwezigheid van risicofactoren en comorbiditeit.

Naast de signalering door huisarts, kinderarts, jeugdarts en jeugdverpleegkundigen, kan er ook signalering plaatsvinden door andere betrokkenen zoals bijvoorbeeld leraren en fysiotherapeuten. Dit hoofdstuk bespreekt alleen het signaleren van overgewicht en obesitas, het signaleren van multiproblematiek wordt in meer detail en met een eigen verwijsschema besproken in het hoofdstuk 'Multiproblematiek' (zie blz. 19).

Piramide en tabel met niveaus van GGR bij kinderen (PON, 2010)

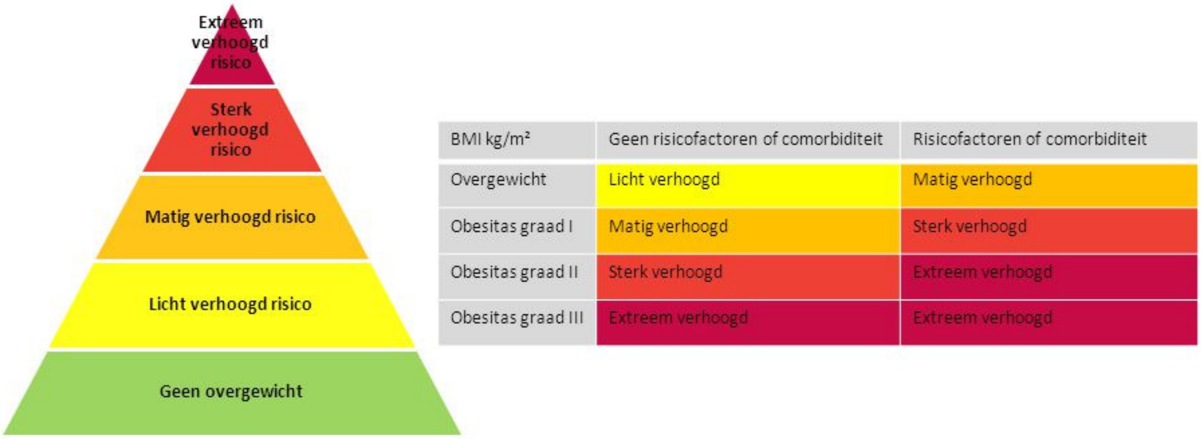

BMI kg/m²	Geen risicofactoren of comorbiditeit	Risicofactoren of comorbiditeit
Overgewicht	Licht verhoogd	Matig verhoogd
Obesitas graad I	Matig verhoogd	Sterk verhoogd
Obesitas graad II	Sterk verhoogd	Extreem verhoogd
Obesitas graad III	Extreem verhoogd	Extreem verhoogd

Niveaus van gewichtsgerelateerd gezondheidsrisico (GGR) bij kinderen (bron: Zorgstandaard Obesitas, Partnerschap Overgewicht Nederland, 2010). De afkappunten in de tabel aangeduid met overgewicht dan wel obesitas graad I, zijn gebaseerd op internationale percentielen van BMI naar leeftijd en geslacht die op volwassen leeftijd overeenkomen met een BMI van 25 resp. 30 kg/m². De afkappunten aangeduid met obesitas graad II en III zijn gebaseerd op percentielen afkomstig van de nationale groeistudie uit 1980 van BMI naar leeftijd en geslacht die op volwassen leeftijd overeenkomen met een BMI van 35 resp. 40 kg/m².

8 mei 2014

[9] Indien de desbetreffende zorgverlener geen mogelijkheid ziet voor het meten van de lengte en het gewicht, wordt er geadviseerd een folder van GO! aan de kinderen en/ of hun ouders te geven, waarop de directe contactgegevens van de JGZ en kindergezondheidscoach staan.

Verwijzen

Op basis van graad van obesitas en de aanwezigheid van risicofactoren, comorbiditeit of complicaties (zie stroomschema ketensamenwerking) wordt er verwezen waarbij de volgende categorisatie leidend zal zijn:

1. Kinderen met gezond gewicht: geen verwijzing, wel wordt aangeraden BOFT[10] - gedragingen te volgen om overgewicht te voorkomen.

2. Kinderen met overgewicht, zonder risicofactoren, complicaties en comorbiditeit worden verwezen naar de JGZ (jeugdverpleegkundigen en jeugdarts)[11]. Begeleiding door jeugdverpleegkundigen vindt plaats. Wanneer deze hulp niet wil baten kan er alsnog worden verwezen naar de KGC.

3. Kinderen met overgewicht met risicofactoren, comorbiditeit en/of complicaties worden verwezen naar de huisarts of kinderarts. De huisarts en kinderarts kunnen vervolgens naar KGC van GO! verwijzen voor verdere inventarisatie van onderliggende multiproblematiek en gecombineerde leefstijlinterventie

4. Kinderen met obesitas graad I, II zonder comorbiditeit, complicaties en risicofactoren worden verwezen naar de huisarts. De huisarts zal voor verdere inventarisatie van onderliggende multiproblematiek en de gecombineerde leefstijlinterventie doorverwijzen naar de KGC van GO!.

5. Kinderen met comorbiditeit/complicaties/ risicofactoren of obesitas graad III worden via de huisarts verwezen naar de kinderarts om de ernst te bepalen en behandeling in te zetten. De kinderarts verwijst naar GO!. Op indicatie worden door de KGC andere zorgverleners uit 1e of 2e lijn ingeschakeld.

Niet opgenomen in de categorisatie maar wel zichtbaar in de schematische weergaven in het stroomschema ketensamenwerking is dat kind en gezin ook zichzelf kunnen 'verwijzen' door direct contact op te nemen met GO! Belangrijk hierbij is dat voor de start een screening plaats vindt door een arts uit de 0e, 1ste, of 2de lijn om onderliggende oorzaak, risicofactoren of complicaties vast te stellen. In het stroomschema is dit zichtbaar met een pijl van de KGC's richting de huisartsen. Wanneer de samenwerking met een kinderarts gewenst is zal deze verwijzing altijd lopen via de huisarts, de KGC's kunnen namelijk nimmer rechtstreeks verwijzen naar de kinderarts.

De rode draad binnen de aanpak van GO! is een goed afgestemde ketenzorg. Terugrapportage naar de oorspronkelijke verwijzer en communicatie tussen de ketenpartners is hierbij essentieel om de kwaliteit van zorg voor de kinderen met overgewicht en obesitas te optimaliseren. Ook is terugrapportage van belang om de verschillende zorgtrajecten op elkaar te laten aansluiten zodat er geen belangrijke informatie verloren gaat en om uitval in het zorgtraject te minimaliseren. Om terugrapportage te waarborgen is er een terugkoppeling protocol opgesteld, zie appendix F. Ook zijn er voor de jeugdarts, huisarts en kinderarts standaard verwijsbrieven opgesteld, zie appendix O.

Behandelprogramma kindergezondheidscoach (gezonde leefstijl coaching)

Na de signalering en verwijzing zal er een intake gesprek worden gepland met kind en gezin. Wanneer er tijdens brede anamnese geen belemmerende factoren worden vastgesteld, wordt er na het intakegesprek gestart met het eerste consult. Binnen de interventie wordt het kind en gezin twee jaar begeleidt waarbij intensief wordt gestart, en vervolgens wordt afgebouwd zodat kind (en gezin) zelf in de lead gaat met als doel gedragsverandering eigen te maken. Het behandelprogramma vindt plaats op basis van 3 pijlers: Voeding, Beweging en Rust & Ontspanning. Zie hieronder een korte beschrijving per pijler. Hoe de consulten precies zijn ingericht aan de hand van deze drie pijlers en

---

[10] BOFT-gedragingen: stimuleren van Bewegen en Buiten spelen, dagelijks Ontbijten, reductie van Frisdranken en andere gezoete dranken en Fastfood en reductie van (energierijke) Tussendoortjes, Tv-kijken en computeren.

[11] Wanneer de effectiviteit uitblijft bij de behandeling door de JGZ , wordt het kind na 6-12 maanden verwezen naar de huisarts voor nader onderzoek en (her)evaluatie.

hoe de kindergezondheidscoach vanaf het moment van de intake te werk gaat, zowel op inhoudelijk vlak als protocollair gezien, staat omschreven in het handboek voor de kindergezondheidscoaches (zie appendix G).

- *Pijler 'Voeding'*
  Door middel van consulten waarin gecoacht wordt naar het leren keuzes maken in 'gezondere eetgewoontes' en het aanbieden van laagdrempelige & leerzame kookworkshop wordt er gestreefd naar het creëren van bewustwording en het teweegbrengen van gedragsverandering ten opzichte van voeding en drank. In het intakegesprek wordt er gevraagd naar voedingsgewoonten en digitaal genoteerd als een voedingsdagboek. Tijdens de consulten wordt met deze kennis geprobeerd het bestaande voedings-/eetschema van kind/ jongere aan te passen naar de richtlijnen van het voedingscentrum dmv het creëren van inzicht. Dit zal altijd gebeuren binnen de eigen leefstijl mogelijkheden van het gezin zoals bijvoorbeeld de vegetarische leefstijl. Kennis van kind (en gezin) over voeding, drank en de werking hiervan in het menselijk lichaam zal gaandeweg vergroten, waarbij in de uitleg van de KGC geredeneerd wordt vanuit de 'waarom' vraag: waarom is iets gezond? Hierdoor is het mogelijk om een 'keuze' te kunnen maken.

- *Pijler 'Beweging'*
  Door middel van consulten, waarin het bewegen in en rondom thuis benadrukt wordt en het aanbieden van laagdrempelige en attractieve beweegactiviteiten in de wijk, wordt er gestreefd naar het creëren van bewustwording en structurele verandering in het beweegpatroon met als eind streven te voldoen aan de beweegnorm. Tijdens de consulten wordt hiernaar toe gewerkt door de drempels te achterhalen, te bespreken en aan te pakken. Wanneer deze drempels fysiek blijken te zijn zal de KGC in overleg gaan met fysiotherapeut binnen het netwerk en wanneer nodig hiernaar verwijzen. Ook wordt tijdens de consulten de kennis rondom bewegen vergroot door onder andere de interactie tussen bewegen en voeding, en de invloed van bewegen op het menselijk lichaam te bespreken

- *Pijler 'Rust & Ontspanning'*
  Tijdens de consulten wordt tevens het belang van lichamelijke en mentale ontspanning uitgelegd. Waaronder uitleg over het belang van een goede nachtrust en de wisselwerking tussen ontspanning, sporten/bewegen en voeding. Maar ook het gebruik van beeldscherm, social media, sociale verwachtingen en prestatiedruk

Door te coachen op bewustwording en het leren keuzes te maken m.b.v. van kennis en inzicht, loopt het teweegbrengen van gedragsverandering als een rode draad door deze 3 pijlers. Aanvullend op de consulten over de 3 pijlers zijn consulten die de volgende thema's bespreken ter ondersteuning: motivatie, visualisatie van een leefstijl doel, bewustwording, bespreken en voorkomen van lastige momenten en 'nee' leren zeggen. Wanneer de KGC merkt dat er alsnog onderliggende, implementatie van gezonde leefstijl belemmerende problemen spelen zal overleg gepleegd worden met cq doorverwezen worden naar een andere zorg- /hulpverlener enof wordt casus ingebracht in MDO. Zulke keuzes gaan altijd in overleg met kind en gezin.

Als aanvulling op de begeleiding van de KGC op de bovengenoemde onderwerpen, stimuleert GO! initiatieven en activiteiten in de wijk rondom deze onderwerpen zoals bijvoorbeeld kookworkshops (zie appendix N) en de supermarktsafari (zie appendix M).

Multidisciplinair overleg (MDO)

Multidisciplinair overleg (MDO) is een vergadering 4 keer per jaar geinitieerd door de KGC over geïncludeerde patiënten vanwege 2 redenen:

I. Doelen niet gehaald
   A. Wanneer er na 6 maanden een een toename in gewicht is van 10 %
   B. Er gewicht is bijgekomen zonder aanwijsbare reden
   C. Er gewicht is bijgekomen door een ongezonde leefstijl (= er heeft geen gedragsverandering plaatsgevonden)
II. Vermoeden op complexe(re) c.q. zorgelijke problematiek waarbij kindergezondheidscoach en verwijzer niet verder komen

De volgende partijen zullen hierbij aanwezig zijn: kindergezondheidscoach, een arts (bij voorkeur eigen huisarts en indien dit niet mogelijk is vindt overdracht plaats van informatie naar aanwezig huisarts, zo mogelijk of zo nodig verpleegkundig specialist kindergeneeskunde of jeugdarts) en wijkteam/jeugdconsulent. Op indicatie zullen (kinder)psycholoog, diëtist, fysiotherapeut, kinderwerker, andere zorg/hulpverlener aanwezig zijn. Ook worden de ouders gevraagd of ze willen deelnemen in het MDO. Het MDO vindt plaats aan de hand van een vast protocol en format, zie appendix H.

Programmagroep

De programmagroep zal 4 worden georganiseerd door de lokale coördinator (voorzitter) met als doel de voortgang te bespreken, de verbinding binnen het netwerk te blijven stimuleren en behouden en de omgeving van kind/gezin zo optimaal mogelijk in te richten tav gezonde leefstijl. Tijdens deze bijeenkomst worden de resultaten in de wijk besproken (inclusie en wetenschappelijke resultaten) maar ook protocollen afgestemd, afspraken gemaakt, overlegd over mogelijke kansen en knelpunten en verbeter mogelijkheden besproken. Ook de programmagroep vindt plaats aan de hand van een vast protocol, zie appendix I voor het programmagroep protocol.
De programmagroep bestaat uit de kernpartners maar wie hierbij precies aanwezig is vanuit het netwerk en hoe hier precies invulling aan wordt gegeven is wijk afhankelijk. Uit voorgaande jaren is gebleken dat vaak de volgende partijen onderdeel zijn van de programmagroep, naast de kindergezondheidscoach en de lokale coördinator: huisarts, verpleegkundig specialist kindergeneeskunde, jeugdarts, diëtist , fysiotherapeut, maatschappelijk werker, wijkteam/jeugd consulent/centrum voor jeugd en gezin, (kinder)psycholoog, kinderwerker, buurtsportcoaches, welzijnswerkers en JOGG. Vanuit GO! wordt de aanwezigheid van zoveel mogelijk partijen binnen het netwerk gestimuleerd.

Voor de volgende professionals is er een protocol ontwikkeld door GO! (zie appendix J voor de protocollen per professional):

1. Huisarts
2. Kinderarts
3. Jeugdarts
4. Diëtist
5. Fysiotherapeut
6. Psycholoog
7. Kinderwerker
8. Wijkteam

Multiproblematiek

Bij kinderen met overgewicht en obesitas is er in vaak sprake van multiproblematiek. Overgewicht is dan slechts een symptoom van onderliggende problematiek zoals armoede, gezondheidsproblemen, onstabiele gezinssituatie, laaggeletterdheid, schulden, et cetera.
In achterstandswijken loopt het percentage van kinderen met overgewicht/obesitas én multiproblematiek al gauw op naar zo'n 80%. Dit vergt een goede afstemming tussen de verschillende zorg- en hulpverleners die zich op dit snijvlak bezighouden. GO! geeft hier samen met de betrokken professionals invulling aan. Dit betekent onder andere dat de jeugdgezondheidszorg zijn rol pakt en specifiek bij kinderen met obesitas én multiproblematiek goed signaleert, gericht verwijst (met belangrijke informatie die voorhanden is) en warm overdraagt naar GO!. Daarnaast moet de centrale hulpverlener in de wijk (vaak een wijkcoach/jeugdconsulent of soortgelijk) nauw contact en goede afstemming hebben met de KGC van GO! voor deze doelgroep. Na het constateren van multiproblematiek is het van belang om na te gaan of de multiproblematiek de boventoon voert. Op basis van deze informatie zijn twee paden te bewandelen die ook zijn uitgewerkt in onderstaand stroomschema :

I. Wanneer multiproblematiek de boventoon voert is de wijkcoach/jeugdconsulent in de lead en wordt er niet direct verwezen naar de kindergezondheidscoach. Er wordt eerst bekeken of het het juiste moment is voor leefstijlverandering. Wanneer multiproblematiek de boventoon voert maar het gezin echter niet openstaat voor de wijkcoach/jeugdconsulent kan het kind wel geïncludeerd worden bij GO! en zal de KGC contact met de wijkcoach/jeugdconsulent op gang proberen te brengen.
II. Wanneer multiproblematiek niet de boventoon voert zal de KGC parallel aan het hulpverleningstraject, ook GO! in gang zetten. Wanneer een kind in het GO! traject vastloopt vanwege onderliggende problematiek, zal de KGC dit bespreekbaar proberen te maken met kind en ouder waarna er terugkoppeling plaatsvindt naar de verwijzer indien dit JGZ en kinderarts zijn en anders naar de huisarts. De KGC overlegt dan samen of er direct stappen worden ondernomen kunnen worden of dat het kind en gezin ingebracht wordt in een MDO. In het MDO zal gezamenlijk worden bepaald wie de regie gaat voeren. Dit kan betekenen dat het GO! programma even 'on hold' wordt gezet, tot het kind weer klaar is voor een terugverwijzing. Hierbij blijft de kindergezondheidscoach op lage frequentie het kind en gezin volgen.

Ondanks dat het stroomschema het signaleren en constateren van multiproblematiek neerzet als een lineair proces, is volgens de visie van GO! het signaleren van multiproblematiek alles behalve lineair. Het schema is er enkel ter verduidelijking van de betrokken partijen en de verwijzingen hiertussen. Binnen de aanpak van GO! wordt het alert wezen op en het constateren van multiproblematiek in het gehele behandeltraject meegenomen. De achterliggende gedachten hierbij is dat multiproblematiek niet makkelijk te achterhalen is en uit ervaring is gebleken dat dit vaak pas naar voren komt naarmate het traject vordert en de vertrouwensband tussen kind en de KGC groeit.

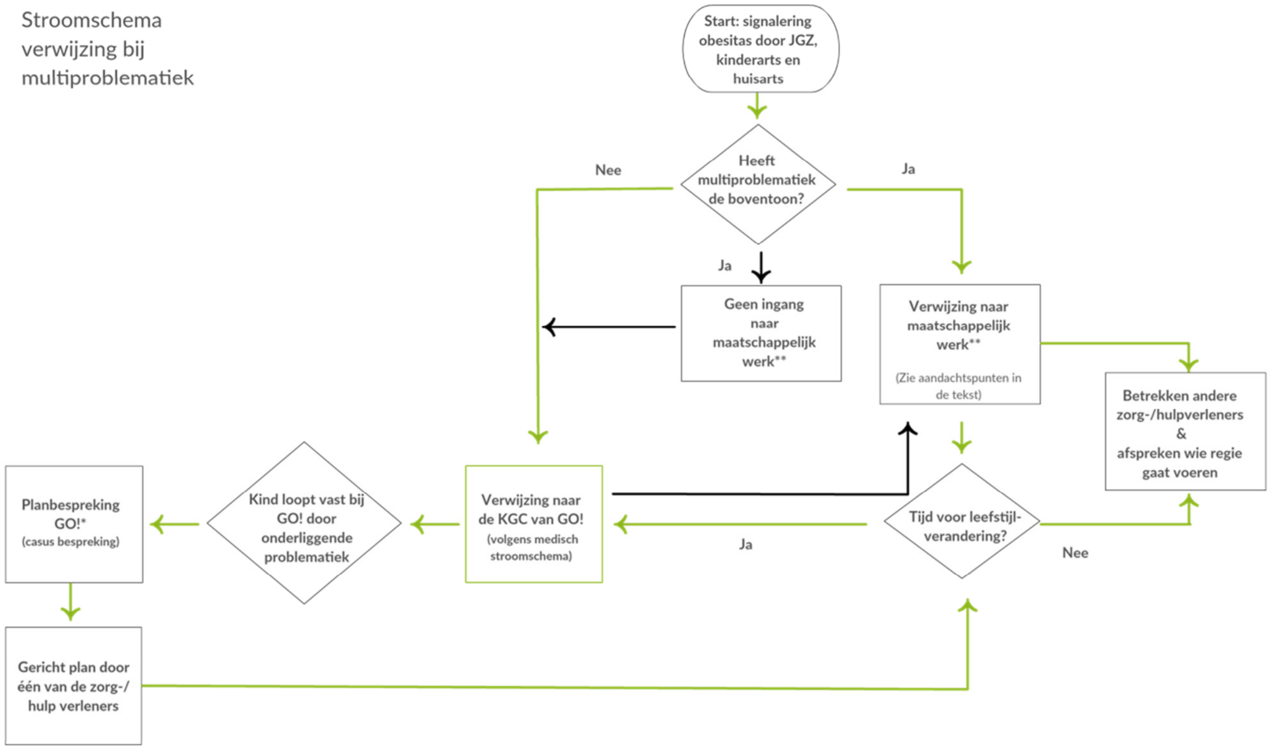

**Zie voor criteria 'planbespreking GO!' de bijlage: protocol planbespreking GO!*

***Voor maatschappelijk werk kan ook wijkcoach, regisseur, centrale zorgverlener, gezinsondersteuning, jeugdconsulent of jongerencoach gelezen worden*

4. Netwerkcoördinatie & kwaliteitsbewaking & wetenschap

Netwerkcoördinatie
Door middel van jaarlijkse netwerkbijeenkomsten en een digitaal netwerkplatform wordt de netwerkorganisatie van informatie voorzien, vindt afstemming plaats over de koers van GO!, vindt kennisdeling plaats en wordt er samengewerkt tussen centraal en lokaal.

Kwaliteitsbewaking
Netwerkbijeenkomsten: via intervisie en netwerkwerkbijeenkomsten zullen kindergezondheidscoaches en lokale coördinatoren op de hoogte gebracht worden van nieuwe richtlijnen en protocollen, zal afstemming plaatsvinden over laatste ontwikkelingen en zal kennis uitgewisseld worden. Ook kunnen er op deze manier de signalen over problemen lokaal worden opgepikt. Dit borgt de juiste kwalitatieve uitvoering van het programma in elke wijk. Het gaat hierbij om de lokale kwaliteit van de uitvoer van het programma GO! te bewaken: staat het netwerk nog zoals opgezet in de implementatiefase?, en werkt het zoals beoogd?, zijn alle partners nog betrokken en enthousiast?, zijn er zaken waar specifiek in deze wijk tegenaan wordt gelopen of worden er juist verbeterpunten gesignaleerd?. Naast het elkaar op de hoogte brengen van nieuwe ontwikkelingen en gezamenlijk oppakken en bespreken van lokale problemen zal ook aandacht worden besteed aan de opleiding component.

Wetenschap
Het gaat hierbij om de wetenschappelijke effectiviteit van het programma. Belangrijke quantitative uitkomstdata (zoals gedefinieerd in het wetenschappelijk protocol), worden gedurende de uitvoer van het programma gemeten en geanalyseerd. Dmv digitale monitoring wordt hierop toegezien en worden mogelijke problemen gesignaleerd zoals tegenvallende inclusie, hoge mate van drop out, verminderd resultaat, etcetera. In nieuwe GO! wijken zullen deze gegevens de eerste twee jaar worden verzameld.

In aanvulling op de effectiviteit data vinden er lokaal ook reguliere evaluatiemomenten plaats en heeft er in januari en februari 2019 een grotere procesevaluatie plaatsgevonden. Met behulp van kwalitatieve onderzoeksmethoden zijn op een constructieve en transparante manier de sterktes, zwaktes, mogelijkheden en barrières besproken met lokale partijen in de wijken waar GO! realiteit is. Door op deze manier te evalueren maakt GO! Centraal de lokale variatie en daarmee ook de lokale leermomenten inzichtelijk en neemt deze kennis mee naar nieuwe wijken. Zie het volledige evaluatierapport in de Appendix K

5. GO! Community

Het vormgeven van de cross over tussen zorg- en sociaal domein, het samenbrengen en daarmee verbinden van zorgprofessionals en professionals uit het sociaal-maatschappelijke domein, om daarmee de zorg rondom kinderen met overgewicht en obesitas te optimaliseren, is essentieel voor GO!. Naast het opzetten van een ketenzorg met uitsluitend zorgprofessionals speelt de interactie met lokale sociaal-maatschappelijke partijen ook een belangrijke rol binnen de aanpak. GO! werkt continu aan deze verbreding met de sociale partners in de wijk door o.a. aan wijkactiviteiten te participeren en via social media de interactie op te zoeken. GO! probeert op deze wijze alle sociaal-maatschappelijke partijen die invloed hebben op de leefstijl van kind en gezin en met kennis van het lokale netwerk aan te spreken en betrokken te krijgen, en daarmee de hele community in zijn kracht te zetten. Voorbeelden hiervan zijn organisaties zoals scholen, kerken, moskeeën, sportaanbieders en andere interventies in de wijk zoals JOGG of voedingsinterventies. Doel is om met en/of vanuit het netwerk op zowel de drie pijlers programma's te verzorgen alsmede in de gehele wijk een gezonde leefstijl te promoten. Hoe deze samenwerking lokaal invulling krijgt zal verschillen per gemeente en per wijk en zal in afstemming zijn met de lokale coördinator en de partijen zelf.

Stuurgroep
Naast het eerder genoemde jaarlijkse contact met de uitvoerende professionals om de voortgang te bespreken en eventuele knelpunten op te lossen, vindt afstemming plaats in een overkoepelende stuurgroep. Deze stuurgroep bestaat uit vertegenwoordigers van betrokken zorgverleners, JGZ, gemeente, Rijnstate en zorgverzekeraar. Hierin wordt de voortgang van het project besproken en afstemming gezocht met betrekking tot de richting waarin de doorontwikkeling vorm kan worden gegeven. Naarmate de opschaling vorm krijgt zal per gemeente of in geval van enkele kleinere gemeenten gezamenlijk een lokale afvaardiging in het leven worden geroepen die met GO! centraal nauw in contact staat. Ook hier zullen de belangrijkste stakeholders in vertegenwoordigd zijn.

Randvoorwaarden

Financiering

GO! is gestart in 2012 en vanaf 2014 gefinancierd door middel van tijdelijke financieringsbronnen (subsidies).

De 3 pilots in Malburgen en Presikhaaf (gemeente Arnhem) en Zetten-Herveld (gemeente Overbetuwe) werden grotendeels bekostigd uit het Regionaal Innovatiefonds, een tijdelijk innovatiefonds, beschikbaar gesteld door de 11 regiogemeenten rondom Arnhem. Daarnaast investeert Gemeente Arnhem zelf ook evenals zorgverzekeraar Menzis. Tevens hebben lokale stichtingen en eerder ook de Provincie Gelderland financieel bijgedragen.

Voor de opschaling naar nieuwe wijken en/of continuering van GO! dient, tot structurele financiering vanuit oa de basisverzekering een rol gaat spelen, nog naar tijdelijke geldstromen zoals gemeentelijke subsidies gezocht te worden. Andere opties worden ook verkend, zoals een Health Impact Bond of alternatieve subsidiestromen.
Bijdragen voor de opschaling worden geheel of gedeeltelijk gehaald bij de betreffende gemeente en de zorgverzekeraar. Wanneer dit niet mogelijk is, zoekt GO! mee naar mogelijke lokale subsidies of financiers om het programma alsnog te kunnen starten.

GO! Centraal zal vanuit Rijnstate de penvoerder zijn, het programma management verzorgen en het budget beheren.

GO! is daarnaast continu op zoek naar duurzame financieringsstromen om GO! te kunnen continueren en bestendigen. Een mogelijkheid is dat in 2020, op initiatief van de eerder genoemde landelijke coalitie, een landelijke innovatie beleidsregel beschikbaar komt waarmee de KGC en de interventie als GLI voor kinderen met overgewicht/obesitas gefinancierd zou kunnen worden. Andere opties waar op ingezet wordt: een met de zorgverzekeraar Menzis gezamenlijke innovatie beleidsregel aanvraag voor GO!, het meedoen binnen de innovatie beleidsregel van Maastricht/Limburg, en het vinden van structurele bekostiging vanuit het publieke/sociale domein met gemeenten in de VGGM regio Midden Gelderland. GO! zal zich ervoor inzetten dat ook de organisatie vanuit en in de wijk (lokale coördinator, programma groep en MDO) alsmede de implementatie in een nieuwe wijk op structurele financiering kunnen rekenen. GO! voert hierover gesprekken met de Nederlandse Zorg Autoriteit (NZA), het Zorg Instituut (ZI), en gemeenten.

(Door) Ontwikkeling

GO! is in 2012 gestart, en anno 2019 geïmplementeerd in 3 gemeenten / 5 wijken, en vanaf eind 2019 in 4 gemeenten / 13 wijken. GO! is 1 van de 8 landelijke VWS proeftuinen, Care for Obesity (C4O), van waaruit in het kader van het Nationaal preventie akkoord opgeschaald wordt naar 35 gemeenten. GO! heeft vanuit C4O bijgedragen aan de totstandkoming van het landelijk ketenaanpak Overgewicht kinderen. (https://www.ketenaanpakovergewichtkinderen.nl/), en voldoet zelf ook aan de eisen die daaraan gesteld worden.

GO! is inmiddels het Gelderse pareltje op het gebied van aanpak kinderobesitas (https://theeconomicboard.com/nieuws/lunch-tweede-kamerleden-prinsjesfestival/).

In 2017 en 2018 is een Maatschappelijke Kosten Baten Analyse uitgevoerd om de kosten en baten van het programma te bepalen. Deze MKBA is uitgevoerd door Ecorys in samenwerking met Society Impact. De conclusie vanuit de MKBA is dat bij de huidige inzet en effectiviteit van GO! de te verwachten maatschappelijke baten ongeveer 4 tot 8 keer zo hoog zijn als de maatschappelijke kosten. Uitgaande van een groep van 100 kinderen zijn de totale toekomstige maatschappelijke baten geraamd op € 1,3 tot 2,1 miljoen. Hier staat een investering van € 0,3 mln. tegenover. De gehele MKBA is openbaar gemaakt en te vinden op: https://www.societyimpact.nl/maatschappelijke-effectenanalyse-go/.

De uitbreiding in de regio brengt met zich dat GO! op veel verschillende plekken landt, met elk zijn eigen kenmerken. GO! is op zoek naar een continue kwaliteitsverbetering van het programma en innovatie. Zij zoekt die innovatie en doorontwikkeling in onder andere in de verdere professionalisering mbt opschaling mogelijkheden en van de belangrijkste spelers (de opleiding van de KGC en lokale coördinatoren). Daarnaast wil GO! oa de impact van het coachen vergroten door middel van serious gaming en de effectiviteit van het netwerk vergroten door middel van de opzet van een learning community.

Wetenschap

De wetenschappelijke tak van GO! vindt plaats in nauwe samenwerking met associate professor Dr. A. Haveman van de WUR, afdeling Maatschappijwetenschappen, Consumptie en Gezonde Levensstijl

De belangrijkste wetenschappelijke data voor GO! is verzameld vanuit de eerste pilot in Malburgen (Arnhem) mbv het wetenschappelijke protocol. Het gaat hierbij om de volgende data:

- Inclusie en uitstroom / drop-out en kenmerken doelgroep (leeftijd, geslacht, etniciteit ed)
- Verwijzers
- Anthropometrie (lengte en gewicht) - gemeten en vastgelegd door KGC's bij elk consult; BMI
- Gedragsverandering ten aanzien van voedings- en beweegpatroon - gemeten en geanalyseerd door Amphi (Radboudumc) middels ALIKO studie
- Fysieke en biochemische data met betrekking tot comorbiditeit en complicaties - gemeten en vastgelegd door huisarts en kinderarts
- Kwaliteit van leven (ouders en kind) - gemeten en vastgelegd door kindergezondheidscoaches middels PedsQol vragenlijsten.

Tijdstippen van evaluatie zijn bij begin, 3, 6,12, en 24 maanden na start interventie. Vragenlijsten met betrekking tot voedings- en beweegpatroon en kwaliteit van leven zullen afgenomen worden bij begin, 3, 6, 12 en 24 maanden na start interventie. De belangrijkste parameters/resultaten zullen de volgende zijn:

- Actuele gewichtsreductie
- Verandering in body mass index (BMI berekend als gewicht in kg gedeeld door lengte in meters in het kwadraat)
- BMI z-score
- Incidentie en reductie van comorbiditeit en complicaties
- Verandering in voedings- en beweegpatroon
- Kwaliteit van leven.

In de overige wijken waar GO! is geïmplementeerd, vindt ook wetenschappelijke evaluatie plaats. Deze zijn beperkter van omvang, dit om de registratielast voor zowel zorgverleners als de doelgroep te verlichten. In de andere wijken worden de volgende zaken gemeten:

- Inclusie en uitstroom / drop-out en kenmerken doelgroep (leeftijd, geslacht, etniciteit ed)
- Verwijzers
- BMI (sds)
- Kwaliteit van leven kind en ouders

In nieuwe wijken bestaat de mogelijkheid nieuwe wetenschappelijke vragen in kaart te brengen
Het streven is om in het najaar van 2019 de eerste wetenschappelijke resultaten te publiceren.

Bijlagen

Appendix A. Functieprofiel kindergezondheidscoach
Appendix B. Functieprofiel Lokale coördinator
Appendix C. Wijkscan GO!
Appendix D. Implementatie strategie
Appendix E. Implementatie stappenplan
Appendix F. Terugkoppelings- protocol (KGC naar huisarts en jeugdarts)
Appendix G. Handboek kindergezondheidscoaches
Appendix H. MDO-protocol en format
Appendix I. Programma groep protocol
Appendix J. Protocollen per professional
Appendix K. Procesevaluatie
Appendix L. Kick-off protocol
Appendix M. Kookworkshops Vitanos
Appendix N. Supermarktsafari
Appendix O. Standaard verwijsbrieven voor Jeugdarts, Huisarts, Kinderarts

Literatuur

Centraal bureau voor de Statistiek. (2018) geraadpleegd van: Gezondheidsenquête/Leefstijlmonitor CBS i.s.m. RIVM

Kent, S., Fusco, F., Gray, A., Jebb, S. A., Cairns, B. J., & Mihaylova, B. (2017). Body mass index and healthcare costs: a systematic literature review of individual participant data studies. Obesity Reviews, 18(8), 869-879.

Freedman, D. S., Khan, L. K., Dietz, W. H., Srinivasan, S. R., & Berenson, G. S. (2001). Relationship of childhood obesity to coronary heart disease risk factors in adulthood: the Bogalusa Heart Study. Pediatrics, 108(3), 712-718.

Freemark, M. (2018). Determinants of Risk for Childhood Obesity. New England Journal of Medicine

Geserick, M., Vogel, M., Gausche, R., Lipek, T., Spielau, U., Keller, E., ... & Körner, A. (2018). Acceleration of BMI in early childhood and risk of sustained obesity. *New England Journal of Medicine*, *379*(14), 1303-1312.

Magarey, A. M., Daniels, L. A., Boulton, T. J., & Cockington, R. A. (2003). Predicting obesity in early adulthood from childhood and parental obesity. International journal of obesity, 27(4), 505.

Must, A., Spadano, J., Coakley, E. H., Field, A. E., Colditz, G., & Dietz, W. H. (1999). The disease burden associated with overweight and obesity. Jama, 282(16), 1523-1529.

Neovius, K., Rehnberg, C., Rasmussen, F., & Neovius, M. (2012). Lifetime productivity losses associated with obesity status in early adulthood. Applied health economics and health policy, 10(5), 309-317.

Van der Lucht, F., & Polder, J. J. (2010). Van gezond naar beter. Volksgezondheid Toekomst Verkenning 2010. Rijksinstituut voor Volksgezondheid en Milieu RIVM.
